# Copper-catalyzed intermolecular formal (5 + 1) annulation of 1,5-diynes with 1,2,5-oxadiazoles

**DOI:** 10.1038/s42004-023-00999-y

**Published:** 2023-09-12

**Authors:** Can-Ming Chen, Ye-Nan Yang, Yin-Zhu Kong, Bo-Han Zhu, Peng-Cheng Qian, Bo Zhou, Long-Wu Ye

**Affiliations:** 1 State Key Laboratory of Physical Chemistry of Solid Surfaces, Key Laboratory of Chemical Biology of Fujian Province, and College of Chemistry and Chemical Engineering, Xiamen University, Xiamen, China; 2 College of Chemistry & Materials Engineering, Wenzhou University, Wenzhou, China; 3 Wenzhou Key Laboratory of Technology and Application of Environmental Functional Materials, Institute of New Materials & Industry Technology, Wenzhou University, Wenzhou, China; 4 State Key Laboratory of Organometallic Chemistry, Shanghai Institute of Organic Chemistry, Chinese Academy of Sciences, Shanghai, China

**Keywords:** Synthetic chemistry methodology, Stereochemistry, Asymmetric synthesis

## Abstract

One-carbon homologation reactions based on one-carbon insertion into the N−O bond of heterocycles have received tremendous interest over the past decades. However, these protocols have to rely on the use of hazardous and not easily accessible diazo compounds as precursors, and examples of the relevant asymmetric catalysis have not been reported. Here we show that a copper-catalyzed intermolecular formal (5 + 1) annulation of 1,5-diynes with 1,2,5-oxadiazoles involving one-carbon insertion into the heterocyclic N−O bond via non-diazo approach. This method enables practical and atom-economic synthesis of valuable pyrrole-substituted oxadiazines in generally moderate to good yields under mild reaction conditions. In addition, the possibility of such an asymmetric formal (5 + 1) annulation also emerges.

## Abstract (web summary)

One-carbon insertion into N–O bonds is widely exploited in organic synthesis, but synthetic protocols for this rely on hazardous diazo precursors that are difficult to access. Here, copper-catalyzed intermolecular formal (5 + 1) annulation of 1,5-diynes with 1,2,5-oxadiazoles is shown to facilitate one-carbon insertion into heterocyclic N–O bonds without the need for diazo precursors.

## Introduction

Oxadiazines are a class of important heterocyclic compounds, which are widely present in drug molecules and show good activities in anticancer, antiviral, antibacterial and weed control^1–14^. As a result, the development of efficient methods for synthesis of oxadiazines continues to draw a great deal of interest from the synthetic community. Although a range of methods have been developed for the construction of oxadiazines^15–24^, only a few reports involved the synthesis of 1,2,5-oxadiazines, which are still challenging to be accessed due to the lack of efficient method.

**Fig. 1:**
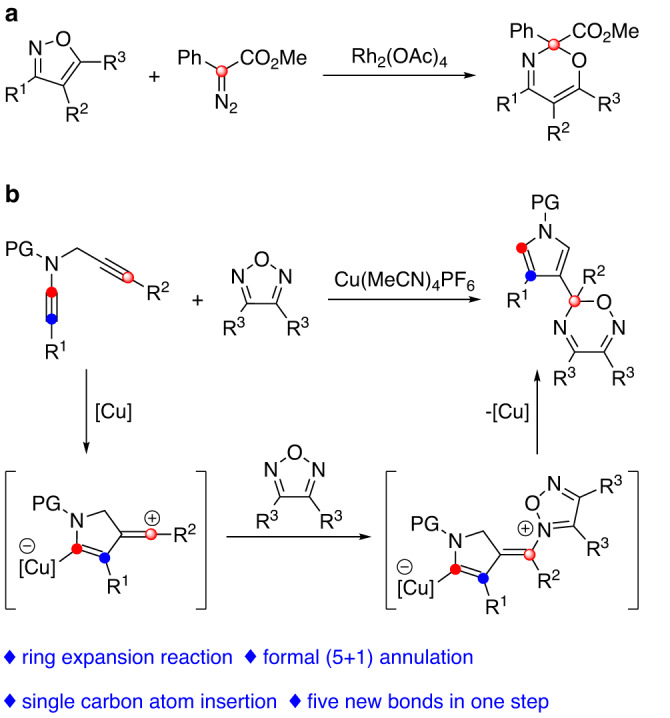
Single carbon atom insertion into N–O bond of heterocycles. **a** Rh-catalyzed formal (5 + 1) annulation of diazo compounds with isoxazoles. **b** Cu-catalyzed formal (5 + 1) annulation of *N*-propargyl ynamides with 1,2,5-oxadiazoles.

One-carbon homologation reactions, in which a carbon chain or carbon ring is expanded by a one-carbon unit, have been widely used in complex molecule synthesis^25–27^. Among various types of ring-expansion reactions, the coupling of cyclic ketones with diazoalkanes, namely the Büchner-Curtius-Schlotterbeck reaction, has been intensively investigated^28–30^. In 2008, an important breakthrough was achieved in this regard by Davies, who demonstrated an elegant protocol on the rhodium-catalyzed ring expansion of isoxazoles via rhodium carbene intermediates insertion into the N−O bond of isoxazoles (Fig. 1a)^31^. Since then, a variety of reactions in which single carbon atom is inserted into the N–O bond of heterocyclic compounds have been reported^32–35^. However, these protocols have to rely on the use of hazardous and not easily accessible diazo compounds as precursors, which severely limit their further synthetic applications and the molecular flexibility. Moreover, to our knowledge, examples of the relevant asymmetric catalysis have not been reported. Thus, it is highly desirable to develop new methods for one-carbon ring expansion, especially those with high flexibility, efficiency, and stereoselectivity.

In the past decade, the chemistry of vinyl cations has received particular attention because of their unique carbene-like reactivity^36,37^. Recently, our group reported a copper-catalyzed diyne cyclization via vinyl cations as key intermediates, providing a variety of polycyclic pyrrole derivatives^38–47^. In particular, the related catalytic asymmetric transformations were established via a remote control of enantioselectivity. Inspired by these results and by our recent study of the development of ynamide chemistry for heterocycle synthesis^48–65^, we envisioned that the vinyl cations generated from copper-catalyzed diyne cyclization might be trapped by 1,2,5-oxadiazoles, eventually leading to valuable 1,2,5-oxadiazines via one-carbon homologation (Fig. 1b). Herein, we describe such a copper-catalyzed one-carbon ring expansion of 1,2,5-oxadiazoles through vinyl cation intermediates generated from *N*-propargyl ynamides, thus constituting an intermolecular formal (5 + 1) annulation of 1,5-diynes with 1,2,5-oxadiazoles.

## Results and discussion

### Screening of reaction conditions

To prohibit the background C–H insertion reaction^38^, 2,6-dimethylphenyl-substituted *N*-propargyl ynamide **1a** was first chosen as the model substrate to react with 1,2,5-oxadiazole **2a** under our previous related reaction conditions^38–47^, and selected results are listed in Table 1. To our delight, the expected 1,2,5-oxadiazine **3a** was indeed formed in 37% yield in the presence of 10 mol % of CuOTf as catalyst (Table 1, entry 1). Subsequent screening of other copper catalysts (Table 1, entries 2–3) revealed that Cu(MeCN)_4_PF_6_ was the best catalyst to deliver the desired product **3a** in 53% yield (Table 1, entry 3). In addition, the use of other typical solvents such as DCM, CHCl_3_, PhMe and PhCl led to decreased yields (Table 1, entries 4–7). Of note, the formation of byproducts decreased with the increase of the equiv of **2a**, and 5 equiv of **2a** was found to be the most appropriate (Table 1, entries 8–9). Gratifyingly, the reaction could be significantly promoted by the addition of 12 mol % of NaBAr^F^_4_ (Table 1, entry 10), and the yield of the reaction could be further increased to 72% under nitrogen atmosphere (Table 1, entry 11). Of note, it was found that the temperature had very slight impact on the yield of **3a** (Table 1, entries 12–13).

**Table 1:** Optimizations of reaction conditions^a^.


Entry	Catalyst	Reaction conditions	Yield (%)^b^
1	CuOTf	DCE, 40 °C,6 h	37
2	Cu(MeCN)_4_BF_4_	DCE, 40 °C, 6 h	41
3	Cu(MeCN)_4_PF_6_	DCE, 40 °C, 6 h	53
4	Cu(MeCN)_4_PF_6_	DCM, 40 °C, 6 h	52
5	Cu(MeCN)_4_PF_6_	CHCl_3_, 40 °C, 10 h	30
6	Cu(MeCN)_4_PF_6_	PhMe, 40 °C, 10 h	28
7	Cu(MeCN)_4_PF_6_	PhCl, 40 °C, 9 h	47
8^c^	Cu(MeCN)_4_PF_6_	DCE, 40 °C, 6 h	38
9^d^	Cu(MeCN)_4_PF_6_	DCE, 40 °C, 6 h	52
10^e^	Cu(MeCN)_4_PF_6_	DCE, 40 °C, 4 h	68
**11**^**e**^	**Cu(MeCN)**_**4**_**PF**_**6**_	**DCE, 40 °C, N**_**2**_**, 4** **h**	**72**
12^e^	Cu(MeCN)_4_PF_6_	DCE, 20 °C, N_2_, 12 h	71
13^e^	Cu(MeCN)_4_PF_6_	DCE, 60 °C, N_2_, 2 h	70

^a^Reaction conditions: **1a** (0.05 mmol), **2a** (2–10 equiv), catalyst (0.005 mmol), solvents (1 mL), 40–60 °C, 2–12 h, in vials.

^b^Measured by ^1^H NMR using 1,3,5-trimethoxybenzene as internal reference.

^c^2 equiv of **2a** was used.

^d^10 equiv of **2a** was used.

^e^12 mol % of NaBAr^F^_4_ was added.

Ns = 4-nitrobenzenesulfonyl.

### Reaction scope study

**Fig. 2:**
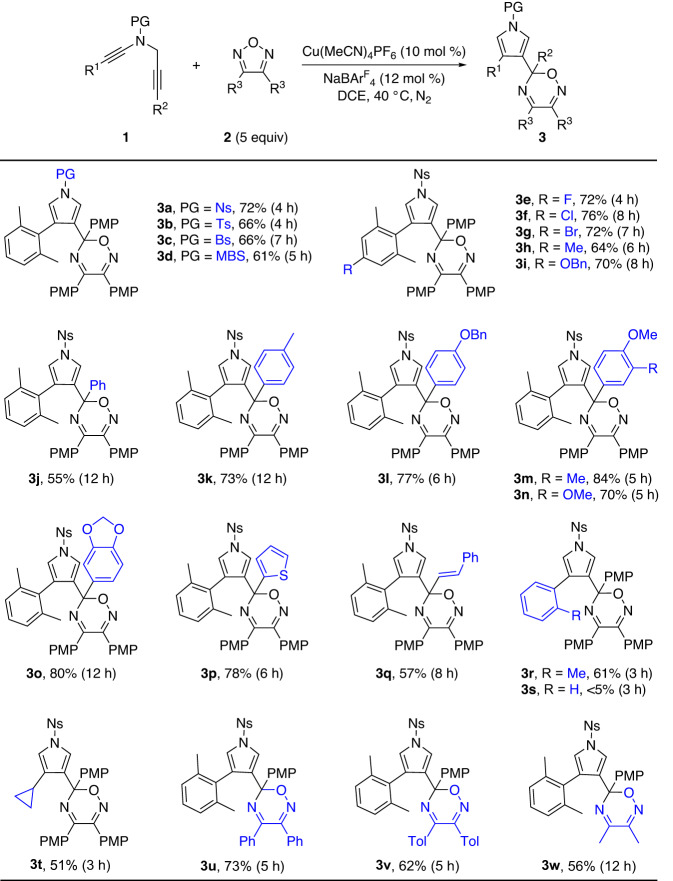
Reaction scope study. Copper-catalyzed synthesis of oxadiazines **3**. Reaction conditions: **1** (0.1 mmol), **2** (0.5 mmol), Cu(MeCN)_4_PF_6_ (10 mol %), NaBAr^F^_4_ (12 mol %), DCE (2 mL), 40 °C, N_2_, 3–12 h, in Schlenk tubes; yields are those for the isolated products.

**Fig. 3:**
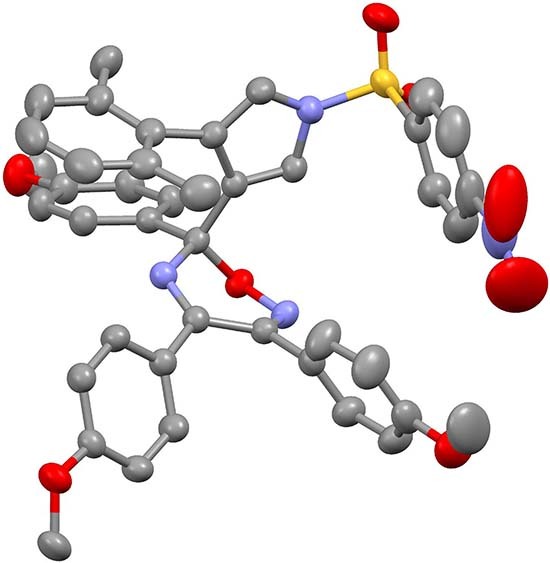
Crystal structure. Structure of compound **3a** in its crystal form. Thermal ellipsoids are shown at 50% probability.

With the optimized reaction conditions in hand (Table 1, entry 11), the scope of this copper-catalyzed formal (5 + 1) annulation was explored. As depicted in Fig. 2, in general, ynamides with different *N*-protecting groups, such as Ns, Ts, Bs and MBS groups, could proceed smoothly to provide the corresponding 1,2,5-oxadiazines **3a–3d** in 61–72% yields (see Supplementary Data 1, 2). In addition, various aryl-substituted ynamides (R^1^ = Ar) bearing both electron-donating and -withdrawing groups on the aromatic ring were tolerated for this reaction, leading to the expected products **3e**–**3i** in 64–76% yields. Moreover, the reaction occurred efficiently for a variety of aryl-substituted *N*-propargyl ynamides (R^2^ = Ar), producing the target oxadiazines **3j–3o** in 55–84% yields. Interestingly, 2-thienyl-substituted and alkenyl-substituted ynamides were also suitable substrates to deliver the desired products **3p** (78%) and **3q** (57%), respectively. Of note, 2-methylphenyl-substituted *N*-propargyl ynamide **1r** was also tolerated to afford the expected **3r** in 61% yield while the use of phenyl-substituted *N*-propargyl ynamide **1** **s** only led to complicated mixtures. Moreover, the cyclopropyl-substituted ynamide could also be smoothly converted into the expected product **3t** in 51% yield. Particularly, this formal (5 + 1) annulation was also extended to other aryl-substituted 1,2,5-oxadiazoles, allowing the formation of the corresponding products **3u–3** **v** in 62–73% yields. Finally, the methyl-substituted 1,2,5-oxadiazole was tolerated in this reaction, and the expected product **3w** was formed in 56% yield. The structure of product **3a** was further confirmed by X-ray diffraction analysis (Fig. 3). Thus, this protocol provides a unique way for rapid and efficient assembly of 1,2,5-oxadiazine derivatives, which are not readily accessible by known methods. It is notable that the use of the alkyl-substituted ynamide **1** **y** (R^2^ = alkyl) as substrate and the relevant thiadiazole and triazole as nucleophiles only led to complicated mixtures under the optimal and related conditions.

**Fig. 4:**
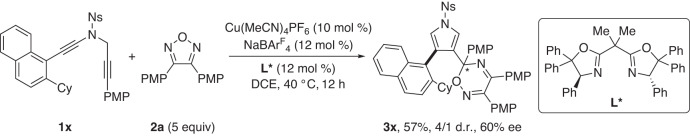
Asymmetric copper-catalyzed formal (5 + 1) annulation. Chiral oxadiazine **3x** was obtained in 57% yield with 60% ee.

Inspired by the above results, we then explored the chiral copper-catalyzed asymmetric formal (5 + 1) annulation. Although direct asymmetric catalysis based on the reaction of *N*-propargyl ynamide **1a** and 1,2,5-oxadiazole **2a** failed to give promising enantioselectivity (<20% ee), the use of the steric group-substituted *N*-propargyl ynamide **1x** as substrate could lead to moderate enantioselectivity bases on our recent study on the chiral copper-catalyzed atroposelective diyne cyclization^45^. As depicted in Fig. 4, we were pleased to find that the use of bisoxazoline (BOX) ligand L^*^ as the chiral ligand resulted in the formation of the desired chiral oxadiazine **3x** in 57% yield and 4:1 d.r. with 60% ee.

### Synthetic applications

**Fig. 5:**
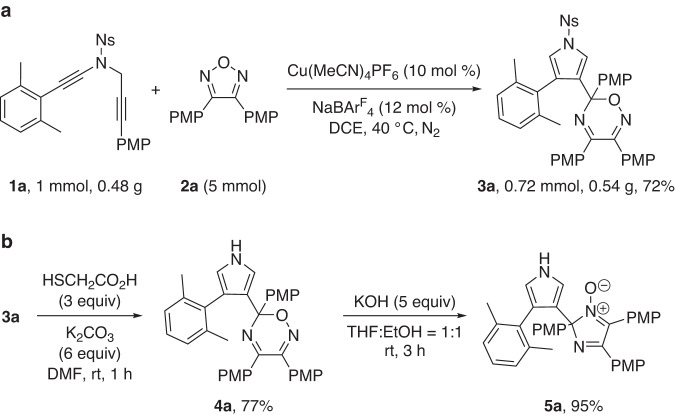
Preparative-scale reaction and product elaboration. **a** Preparative-scale reaction of **1a**. **b** Transformation of **3a** into **4a** and **5a**.

To further demonstrate the utility of this annulation reaction, we carried out several synthetic transformations of the pyrrole-substituted oxadiazine **3a**, as illustrated in Fig. 5. First, the preparative-scale reaction of ynamide **1a** was conducted under the standard conditions, and the desired product **3a** was formed in 72% yield. In addition, the Ns group of **3a** could be readily removed to deliver product **4a** in 77% yield. Interestingly, the treatment of **4a** with 5 equiv of KOH led to the formation of the corresponding *N*-oxide compound **5a** in 95% yield via a ring contraction way.

### Plausible reaction mechanism

**Fig. 6:**
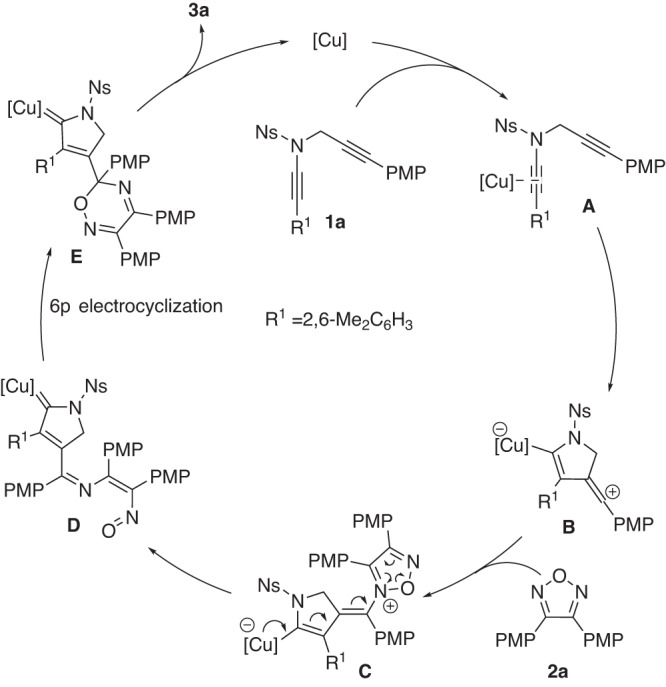
Mechanistic hypothesis. Plausible catalytic cycle.

On the basis of the above experimental results and our previous study on the copper-catalyzed cyclization of *N*-propargyl ynamides^38–47^, a plausible reaction mechanism to rationalize the formation of pyrrole-substituted oxadiazine **3a** is shown in Fig. 6. First, catalytic Cu(I) species coordinate with the electron-richer amide-tethered C–C triple bond of ynamide **1a**, generating the precursor **A**, which subsequently undergoes nucleophilic attack by another C−C triple bond to produce the key vinyl cation intermediate **B**. Then, the vinyl cation is trapped by oxadiazole **2a**, providing intermediate **C**. Next, ring-opening of oxadiazole, followed by 6π electrocyclization, leads to donor/donor copper carbene intermediate **E**. Finally, intermediate **E** undergoes [1,4]-H shift and demetallation to furnish the corresponding product **3a**. On the other hand, by using chiral copper catalyst, chiral product **3x** can be obtained via asymmetric atroposelective cyclization through a remote control of enantioselectivity.

## Conclusions

In summary, we have disclosed a copper-catalyzed intermolecular formal (5 + 1) annulation of 1,5-diynes with 1,2,5-oxadiazoles, allowing the practical and atom-economic synthesis of valuable 1,2,5-oxadiazine derivatives in generally moderate to good yields under mild reaction conditions. This reaction is realized by inserting vinyl cations formed through 1,5-diyne cyclization into the N–O bond of 1,2,5-oxadiazoles. In addition, the possibility of such an asymmetric formal (5 + 1) annulation also emerges. Importantly, the protocol achieves one-carbon ring expansion of 1,2,5-oxadiazoles base on vinyl cations, and provides a rare approach to 1,2,5-oxadiazines. Further application of this type of copper-catalyzed one-carbon homologation will be pursued in our laboratory.

## Methods

### Materials

Unless otherwise noted, materials were obtained commercially and used without further purification. All the solvents were treated according to general methods. Flash column chromatography was performed over silica gel (300–400 mesh). See Supplementary Methods for experimental details.

### General methods

^1^H NMR spectra and ^13^C NMR spectra were recorded on a Bruker AV-400 spectrometer in chloroform-d_3_. For ^1^H NMR spectra, chemical shifts are reported in ppm with the internal TMS signal at 0.0 ppm as a standard. For ^13^C NMR spectra, chemical shifts are reported in ppm with the internal chloroform signal at 77.0 ppm as a standard. Infrared spectra were recorded on a Nicolet AVATER FTIR330 spectrometer as thin film and are reported in reciprocal centimeter (cm^−1^). Mass spectra were recorded with Micromass QTOF2 Quadrupole/Time-of-Flight Tandem mass spectrometer using electron spray ionization. ^1^H NMR, ^13^C NMR spectra and HPLC spectra are supplied for all compounds: see Supplementary Information. See Supplementary Methods for the characterization data of compounds not listed in this part.

### General procedure for the synthesis of 1,2,5-oxadiazines 3

1,2,5-oxadiazole **2** (0.5 mmol), NaBAr^F^_4_ (0.012 mmol, 2.7 mg), and Cu(MeCN)_4_PF_6_ (0.01 mmol, 3.7 mg) were added in this order to the *N*-propargyl ynamide **1** (0.1 mmol) in DCE (2 mL) at room temperature. The reaction mixture was stirred at 40 °C and the progress of the reaction was monitored by TLC. Upon completion, the mixture was concentrated under reduced pressure and the residue was purified by column chromatography on silica gel (dichloromethane/hexane) to afford the desired product **3**.

### Supplementary information

Supporting Information

Description of additional supplementary file

Supplementary Data 1

Supplementary Data 2

## Data Availability

Data for the crystal structure reported in this paper has been deposited at the Cambridge Crystallographic Data Centre (CCDC) under the deposition number CCDC 2268060 (**3a**). Copies of these data can be obtained free of charge via www.ccdc.cam.ac.uk/data_request/cif. All other data supporting the findings of this study, including experimental procedures and compound characterization, are available within the paper and its Supplementary Data 1, 2, or from the corresponding authors on request.
