# Supplementary material for: Copper-catalyzed intermolecular formal (5 + 1) annulation of 1,5-diynes with 1,2,5-oxadiazoles
Source: Commun Chem. 2023 Sep 12;6:194. doi: 10.1038/s42004-023-00999-y (PMC10497616; doi:10.1038/s42004-023-00999-y)
Supplement: Supplementary file 1 — Supporting Information [file 42004_2023_999_MOESM1_ESM.pdf]

Supporting Information

Copper-catalyzed intermolecular formal [5+1] annulation of 1,5-diynes with 1,2,5-oxadiazoles

Can-Ming Chen,[1] Ye-Nan Yang,[1] Yin-Zhu Kong,[1] Bo-Han Zhu,[2] Peng-Cheng Qian,[1,2,3*] Bo Zhou,[1] & Long-Wu Ye[1,4*]

[1]State Key Laboratory of Physical Chemistry of Solid Surfaces, Key Laboratory of Chemical Biology of Fujian Province, and College of Chemistry and Chemical Engineering, Xiamen University, Xiamen 361005, China.

[2]College of Chemistry & Materials Engineering, Wenzhou University, Wenzhou 325035, China.

[3]Wenzhou Key Laboratory of Technology and Application of Environmental Functional Materials, Institute of New Materials & Industry Technology, Wenzhou University, Wenzhou 325000, China.

[4]State Key Laboratory of Organometallic Chemistry, Shanghai Institute of Organic Chemistry, Chinese Academy of Sciences, Shanghai 200032, China.

*Emails: longwuye@xmu.edu.cn; qpc@wzu.edu.cn

Table of Contents

1 Supplementary Methods 2

1.1 General Information 2

1.2 Preparation of Starting Materials 3

1.3 General Procedure for the Synthesis of 1,2,5-Oxadiazines **3** 16

1.4 Preparative-Scale Synthesis and Synthetic Transformations 32

1.5 Crystal Data and Structure Refinement for **3a** 35

1.6 HPLC Chromatograms 36

2 Supplementary References 37

1. Supplementary Methods

1.1 General Information

Acetonitrile (ACS grade), toluene (ACS grade), ethyl acetate (ACS grade) and hexanes (ACS grade) were obtained commercially and used without further purification. Methylene chloride, tetrahydrofuran and diethyl ether were purified according to standard methods unless otherwise noted. Commercially available reagents were used without further purification. All reactions were carried out with a Titan HMS-14 digital magnetic stirrer with hot plate. Reactions were monitored by thin layer chromatography (TLC) using silicycle pre-coated silica gel plates. Flash column chromatography was performed over silica gel (300-400 mesh). Infrared spectra were recorded on a Nicolet AVATER FTIR330 spectrometer as thin film and are reported in reciprocal centimeter (cm^{-1}). Mass spectra were recorded with Agilent 6230 ESI-TOF MS using electron spray ionization. X-ray diffraction analysis was recorded on a Rigaku AFC7R X-ray single crystal diffractometer. HPLC analyses were carried out in a chromatograph equipped with a UV diode-array detector using chiral stationary columns from Daicel.

^{1}H NMR spectra and ^{13}C NMR spectra were recorded on a Bruker AV-400 spectrometer and a Bruker AV-500 spectrometer in chloroform-d_3. Chemical shifts are reported in ppm with the internal TMS signal at 0.0 ppm as a standard for ^{1}H NMR spectra and with the internal chloroform signal at 77.0 ppm as a standard for ^{13}C NMR spectra. The data is being reported as (s = singlet, d = doublet, t = triplet, m = multiplet or unresolved, brs = broad singlet, coupling constant(s) in Hz, integration).

1.2 Preparation of Starting Materials

1.2.1 Representative synthetic procedure for the preparation of ynamides 1 (1a-1v)[1,2]

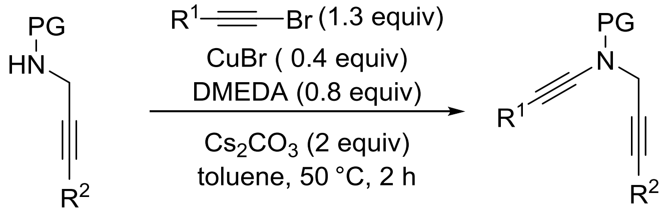

1, 50-82%

To a solution of the sulfonyl propargylamide derivative (1 mmol) in toluene (10 mL) were added copper bromide (0.4 mmol, 57.4 mg), DMEDA (0.8 mmol, 88 μL), Cs_2CO_3 (2.0 mmol, 651.6 mg) and alkynyl bromide derivative (1.3 mmol) sequentially. The reaction was stirred at 50 °C and the progress of the reaction was monitored by TLC. The reaction typically took 2 h. Upon completion, the mixture was filtered and concentrated under reduced pressure. The residue was purified by column chromatography on silica gel (PE/EA) to afford the desired diyne **1**. Ynamides **1b**, **1c** and **1d** are known and the spectroscopic data match those reported in our previous work.[3]

***N*-((2,6-dimethylphenyl)ethynyl)-*N*-(3-(4-methoxyphenyl)prop-2-yn-1-yl)-4-nitrobenzenesulfonamide (1a)**

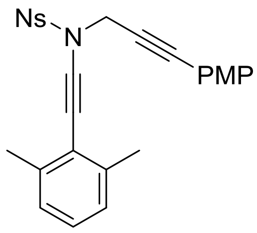

1a

Compound **1a** was prepared in 75% yield (335.9 mg) according to the general procedure. The substrate was isolated through silica gel column chromatography (PE/EA = 10/1) as a yellow solid (mp 144–145 °C). ^{1}H NMR (400 MHz, $CDCl_3$) δ 8.26 (d, *J* = 8.8 Hz, 2H), 8.21 (d, *J* = 9.2 Hz, 2H), 7.15 – 7.06 (m, 1H), 7.06 – 6.92 (m, 4H), 6.74 (d, *J* = 8.4 Hz, 2H), 4.65 (s, 2H), 3.78 (s, 3H), 2.38 (s, 6H); ^{13}C NMR (100 MHz, $CDCl_3$) δ 160.1, 150.5,

142.9, 140.2, 132.9, 129.5, 127.9, 126.7, 124.0, 121.6, 114.0, 113.2, 88.8, 87.2, 79.2, 69.3, 55.3, 43.8, 21.1; IR (neat): 3104, 2918, 2235, 1606, 1532, 1377, 1250, 1177, 765, 609 cm^{-1}; HRESIMS Calcd for $[C_{26}H_{22}N_2NaO_5S]^+$ $(M + Na^+)$ 497.1142, found 497.1148.

***N*-((4-fluoro-2,6-dimethylphenyl)ethynyl)-*N*-(3-(4-methoxyphenyl)prop-2-yn-1-yl)-4-nitrobenzenesulfonamide (1e)**

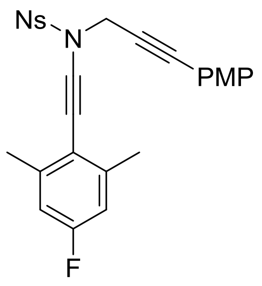

1e

Compound **1e** was prepared in 66% yield (325.1 mg) according to the general procedure. The substrate was isolated through silica gel column chromatography (PE/EA = 10/1) as a pale yellow solid (mp 138–139 °C). ^{1}H NMR (400 MHz, $CDCl_3$) δ 8.26 (d, *J* = 9.2 Hz, 2H), 8.20 (d, *J* = 8.8 Hz, 2H), 7.00 (d, *J* = 8.4 Hz, 2H), 6.77 – 6.71 (m, 4H), 4.64 (s, 2H), 3.78 (s, 3H), 2.36 (s, 6H); ^{13}C NMR (100 MHz, $CDCl_3$) δ 161.9 (d, *J* = 247.0 Hz), 160.1, 150.5, 143.0 (d, *J* = 9.0 Hz), 142.8, 132.9, 129.4, 124.0, 117.6 (d, *J* = 3.0 Hz), 114.0, 113.8 (d, *J* = 22.0 Hz), 113.2, 88.3, 87.2, 79.2, 68.3, 55.3, 43.7, 21.1; ^{19}F NMR (376 MHz, $CDCl_3$) δ -112.2; IR (neat): 3105, 2918, 2235, 1606, 1532, 1377, 1250, 1177, 765, 609 cm^{-1}; HRESIMS Calcd for $[C_{26}H_{21}FN_2NaO_5S]^+$ $(M + Na^+)$ 515.1047, found 515.1044.

***N*-((4-chloro-2,6-dimethylphenyl)ethynyl)-*N*-(3-(4-methoxyphenyl)prop-2-yn-1-yl)-4-nitrobenzenesulfonamide (1f)**

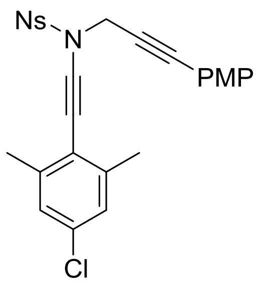

1f

Compound **1f** was prepared in 69% yield (351.2 mg) according to the general procedure. The substrate was isolated through silica gel column chromatography (PE/EA = 10/1) as a pale yellow solid (mp 141–143 °C). ^{1}H NMR (400 MHz, $CDCl_3$) δ 8.27 (d, *J* = 9.2 Hz, 2H), 8.20 (d, *J* = 8.8 Hz, 2H), 7.09 – 6.94 (m, 4H), 6.75 (d, *J* = 8.8 Hz, 2H), 4.65 (s, 2H), 3.79 (s, 3H), 2.35 (s, 6H); ^{13}C NMR (100 MHz, $CDCl_3$) δ 160.2, 150.6, 142.8, 141.8, 133.4, 132.9, 129.4, 126.8, 124.0, 120.3, 114.0, 113.1, 89.4, 87.3, 79.0, 68.6, 55.3, 43.7, 20.9; IR (neat): 3107, 2962, 2235, 1606, 1532, 1372, 1250, 1175, 752, 611 cm^{-1}; HRESIMS Calcd for $[C_{26}H_{21}ClN_2NaO_5S]^+$ (M + Na^+) 531.0752, found 531.0733.

***N*-((4-bromo-2,6-dimethylphenyl)ethynyl)-*N*-(3-(4-methoxyphenyl)prop-2-yn-1-yl)-4-nitrobenzenesulfonamide (1g)**

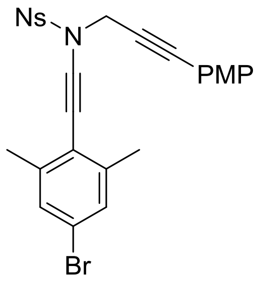

1g

Compound **1g** was prepared in 70% yield (387.4 mg) according to the general procedure. The substrate was isolated through silica gel column chromatography (PE/EA = 10/1) as a pale yellow solid (mp 133–134 °C). ^{1}H NMR (400 MHz, $CDCl_3$) δ 8.26 (d, *J* = 9.2 Hz, 2H), 8.18 (d, *J* = 9.2 Hz, 2H), 7.17 (s, 2H), 7.00 (d, *J* = 8.8 Hz, 2H), 6.74 (d, *J* = 8.8 Hz, 2H), 4.64 (s, 2H), 3.78 (s, 3H), 2.34 (s, 6H); ^{13}C NMR (100 MHz, $CDCl_3$) δ 160.1, 150.5, 142.7, 141.9, 132.9, 129.6, 129.3, 124.0, 121.7, 120.7, 114.0, 113.1, 89.7, 87.2, 79.0, 68.6, 55.2, 43.6, 20.8; IR (neat): 3107, 2953, 2236, 1606, 1532, 1378, 1250, 1176, 752, 611 cm^{-1}; HRESIMS Calcd for $[C_{26}H_{21}BrN_2NaO_5S]^+$ (M + Na^+) 575.0247, found 575.0238.

***N*-(mesitylethynyl)-*N*-(3-(4-methoxyphenyl)prop-2-yn-1-yl)-4 nitrobenzenesulfonamide (1h)**

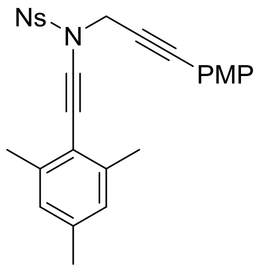

1h

Compound **1h** was prepared in 67% yield (327.3 mg) according to the general procedure. The substrate was isolated through silica gel column chromatography (PE/EA = 10/1) as a pale yellow solid (mp 116–118 ^oC). ^1H NMR (400 MHz, $CDCl_3$) δ 8.29 – 8.09 (m, 4H), 6.99 (d, *J* = 8.8 Hz, 2H), 6.84 (s, 2H), 6.73 (d, *J* = 8.4 Hz, 2H), 4.63 (s, 2H), 3.76 (s, 3H), 2.33 (s, 6H), 2.26 (s, 3H); ^{13}C NMR (100 MHz, $CDCl_3$) δ 160.0, 150.4, 142.7, 140.2, 138.0, 132.8, 129.4, 127.6, 123.8, 118.5, 113.9, 113.2, 88.0, 87.0, 79.3, 69.2, 55.2, 43.7, 21.2, 20.9; IR (neat): 3104, 2921, 2234, 1607, 1532, 1377, 1250, 1176, 757, 611 cm^{-1}; HRESIMS Calcd for $[C_{27}H_{24}N_2NaO_5S]^+$ (M + Na^+) 511.1298, found 511.1300.

***N*-((4-(benzyloxy)-2,6-dimethylphenyl)ethynyl)-*N*-(3-(4-methoxyphenyl)prop-2-yn-1-yl)-4-nitrobenzenesulfonamide (1i)**

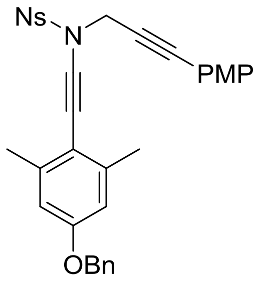

1i

Compound **1i** was prepared in 72% yield (418.1 mg) according to the general procedure. The substrate was isolated through silica gel column chromatography (PE/EA = 10/1) as a pale yellow solid (mp 119–121 ^oC). ^1H NMR (400 MHz, $CDCl_3$) δ 8.25 (d, *J* = 8.8 Hz, 2H), 8.20 (d, *J* = 8.8 Hz, 2H), 7.47 – 7.28 (m, 5H), 7.00 (d, *J* = 8.8 Hz, 2H), 6.74 (d, *J* = 8.8 Hz, 2H), 6.67 (s, 2H), 5.04 (s, 2H), 4.63 (s, 2H), 3.79 (s, 3H), 2.34 (s, 6H); ^{13}C NMR (100 MHz, $CDCl_3$) δ 160.1, 158.5, 150.5, 142.9, 142.6, 136.7, 132.9, 129.5, 128.6, 128.0, 127.4, 123.9, 114.1, 114.0, 113.4, 113.3, 87.3, 87.1, 79.3, 69.8, 69.0, 55.3, 43.9, 21.4; IR

(neat): 3105, 2920, 2238, 1605, 1532, 1375, 1276, 1174, 750, 611 cm^{-1}; HRESIMS Calcd for $[C_{33}H_{28}N_2NaO_6S]^+$ $(M + Na^+)$ 603.1560, found 603.1554.

***N*-((2,6-dimethylphenyl)ethynyl)-4-nitro-*N*-(3-phenylprop-2-yn-1-yl)benzenesulfonamide (1j)**

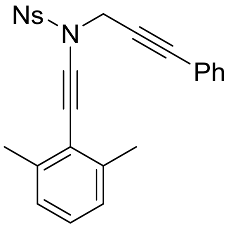

1j

Compound **1j** was prepared in 77% yield (342.3 mg) according to the general procedure. The substrate was isolated through silica gel column chromatography (PE/EA = 10/1) as a pale yellow solid (mp 129–130 °C). ^{1}H NMR (400 MHz, $CDCl_3$) δ 8.25 (d, *J* = 8.8 Hz, 2H), 8.21 (d, *J* = 8.8 Hz, 2H), 7.33 – 7.26 (m, 1H), 7.25 – 7.20 (m, 2H), 7.14 – 7.00 (m, 5H), 4.67 (s, 2H), 2.38 (s, 6H); ^{13}C NMR (100 MHz, $CDCl_3$) δ 150.5, 142.7, 140.2, 131.3, 129.4, 129.1, 128.3, 127.9, 126.7, 124.0, 121.6, 121.2, 88.7, 87.1, 80.5, 69.4, 43.6, 21.0; IR (neat): 3107, 2919, 2232, 1633, 1532, 1378, 1312, 1178, 692, 572 cm^{-1}; HRESIMS Calcd for $[C_{25}H_{20}N_2NaO_4S]^+$ $(M + Na^+)$ 467.1036, found 467.1046.

***N*-((2,6-dimethylphenyl)ethynyl)-4-nitro-*N*-(3-(*p*-tolyl)prop-2-yn-1-yl)benzenesulfonamide (1k)**

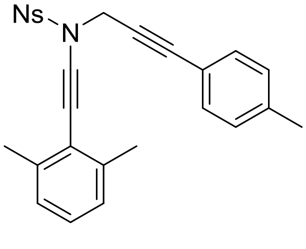

1k

Compound **1k** was prepared in 82% yield (376.0 mg) according to the general procedure. The substrate was isolated through silica gel column chromatography (PE/EA = 10/1) as a pale yellow solid (mp 132–134 °C). ^{1}H NMR (400 MHz, $CDCl_3$) δ 8.28 – 8.14 (m, 4H), 7.13 – 7.06 (m, 1H), 7.01 (d, *J* = 8.0 Hz, 4H), 6.94 (d, *J* = 8.0 Hz, 2H), 4.65 (s, 2H), 2.38

(s, 6H), 2.30 (s, 3H); ^{13}C NMR (100 MHz, $CDCl_3$) δ 150.5, 142.7, 140.2, 139.4, 131.2, 129.4, 129.1, 127.8, 126.7, 123.9, 121.6, 118.1, 88.8, 87.2, 79.8, 69.3, 43.6, 21.4, 21.0; IR (neat): 3109, 2922, 2232, 1634, 1532, 1378, 1348, 1177, 740, 609 cm^{-1}; HRESIMS Calcd for $[C_{26}H_{22}N_2NaO_4S]^+$ (M + Na^+) 481.1192, found 481.1194.

***N*-(3-(4-(benzyloxy)phenyl)prop-2-yn-1-yl)-*N*-((2,6-dimethylphenyl)ethynyl)-4-nitrobenzenesulfonamide (1l)**

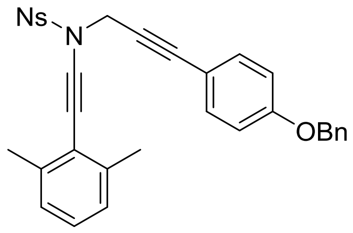

1l

Compound **1l** was prepared in 73% yield (402.0 mg) according to the general procedure. The substrate was isolated through silica gel column chromatography (PE/EA = 10/1) as a pale yellow solid (mp 148–150 ^oC). ^1H NMR (400 MHz, $CDCl_3$) δ 8.26 (d, *J* = 8.8 Hz, 2H), 8.21 (d, *J* = 8.8 Hz, 2H), 7.51 – 7.29 (m, 5H), 7.17 – 7.06 (m, 1H), 7.08 – 6.94 (m, 4H), 6.82 (d, *J* = 8.8 Hz, 2H), 5.04 (s, 2H), 4.65 (s, 2H), 2.38 (s, 6H); ^{13}C NMR (100 MHz, $CDCl_3$) δ 159.3, 150.5, 142.9, 140.2, 136.3, 133.0, 129.5, 128.6, 128.1, 127.9, 127.5, 126.7, 124.0, 121.6, 114.9, 113.5, 88.8, 87.2, 79.3, 70.1, 69.4, 43.8, 21.1; IR (neat): 3107, 2920, 2231, 1634, 1532, 1376, 1347, 1175, 749, 565 cm^{-1}; HRESIMS Calcd for $[C_{32}H_{26}N_2NaO_5S]^+$ (M + Na^+) 573.1455, found 473.1449.

***N*-((2,6-dimethylphenyl)ethynyl)-*N*-(3-(4-methoxy-3-methylphenyl)prop-2-yn-1-yl)-4-nitrobenzenesulfonamide (1m)**

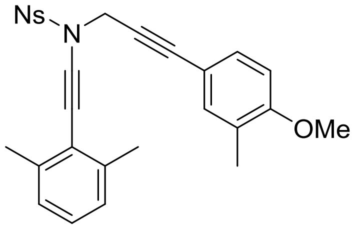

1m

Compound **1m** was prepared in 65% yield (317.5 mg) according to the general procedure. The substrate was isolated through silica gel column chromatography (PE/EA = 10/1) as a pale yellow solid (mp 105–107 °C). ^{1}H NMR (400 MHz, $CDCl_3$) δ 8.26 (d, *J* = 8.8 Hz, 2H), 8.21 (d, *J* = 8.8 Hz, 2H), 7.17 – 7.06 (m, 1H), 7.03 (d, *J* = 7.2 Hz, 2H), 6.89 (d, *J* = 8.0 Hz, 1H), 6.81 (s, 1H), 6.64 (d, *J* = 8.4 Hz, 1H), 4.64 (s, 2H), 3.80 (s, 3H), 2.39 (s, 6H), 2.11 (s, 3H); ^{13}C NMR (100 MHz, $CDCl_3$) δ 158.4, 150.4, 142.9, 140.2, 133.5, 130.5, 129.5, 127.9, 127.0, 126.7, 124.0, 121.7, 112.7, 109.7, 88.9, 87.5, 78.8, 69.2, 55.3, 43.8, 21.1, 15.9; IR (neat): 3105, 2923, 2230, 1605, 1532, 1378, 1347, 1177, 749, 568 cm^{-1}; HRESIMS Calcd for $[C_{27}H_{24}N_2NaO_5S]^+$ (M + Na$^+$) 511.1298, found 511.1296.

***N*-(3-(3,4-dimethoxyphenyl)prop-2-yn-1-yl)-*N*-((2,6-dimethylphenyl)ethynyl)-4-nitrobenzenesulfonamide (1n)**

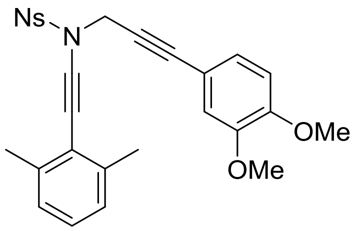

1n

Compound **1n** was prepared in 74% yield (371.4 mg) according to the general procedure. The substrate was isolated through silica gel column chromatography (PE/EA = 10/1) as a pale yellow solid (mp 140–142 °C). ^{1}H NMR (400 MHz, $CDCl_3$) δ 8.26 (d, *J* = 8.8 Hz, 2H), 8.21 (d, *J* = 8.8 Hz, 2H), 7.15 – 7.06 (m, 1H), 7.01 (d, *J* = 7.6 Hz, 2H), 6.75 – 6.59 (m, 3H), 4.65 (s, 2H), 3.85 (s, 3H), 3.78 (s, 3H), 2.38 (s, 6H); ^{13}C NMR (100 MHz, $CDCl_3$) δ 150.4, 150.0, 148.6, 142.7, 140.1, 129.3, 127.8, 126.7, 124.9, 123.9, 121.5, 113.9, 113.3, 110.9, 88.7, 87.2, 79.0, 69.3, 55.8, 55.7, 43.6, 21.0; IR (neat): 3106, 2937, 2231, 1600, 1532, 1377, 1348, 1177, 748, 612 cm^{-1}; HRESIMS Calcd for $[C_{27}H_{24}N_2NaO_6S]^+$ (M + Na$^+$) 527.1247, found 527.1229.

***N*-(3-(benzo[*d*][1,3]dioxol-5-yl)prop-2-yn-1-yl)-*N*-((2,6-dimethylphenyl)ethynyl)-4-nitrobenzenesulfonamide (1o)**

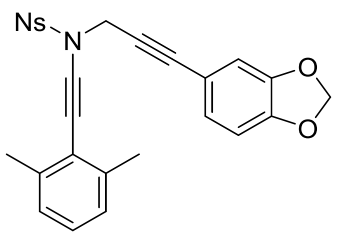

1o

Compound **1o** was prepared in 65% yield (316.6 mg) according to the general procedure. The substrate was isolated through silica gel column chromatography (PE/EA = 10/1) as a pale yellow solid (mp 142–144 °C). ^{1}H NMR (400 MHz, $CDCl_3$) δ 8.26 (d, *J* = 9.2 Hz, 2H), 8.20 (d, *J* = 9.2 Hz, 2H), 7.14 – 7.07 (m, 1H), 7.03 (d, *J* = 7.6 Hz, 2H), 6.71 – 6.55 (m, 2H), 6.49 (d, *J* = 0.8 Hz, 1H), 5.95 (s, 2H), 4.63 (s, 2H), 2.38 (s, 6H); ^{13}C NMR (100 MHz, $CDCl_3$) δ 150.5, 148.5, 147.4, 142.8, 140.2, 129.4, 127.9, 126.7, 126.2, 124.0, 121.6, 114.4, 111.2, 108.4, 101.5, 88.8, 87.1, 78.9, 69.4, 43.7, 21.0; IR (neat): 3107, 2921, 2231, 1635, 1532, 1377, 1347, 1177, 749, 569 cm^{-1}; HRESIMS Calcd for $[C_{26}H_{20}N_2NaO_6S]^+$ (M + Na$^+$) 511.0934, found 511.0948.

***N*-((2,6-dimethylphenyl)ethynyl)-4-nitro-*N*-(3-(thiophen-2-yl)prop-2-yn-1-yl)benzenesulfonamide (1p)**

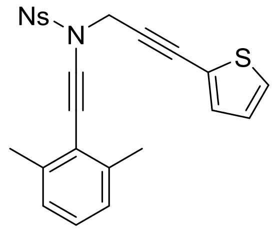

1p

Compound **1p** was prepared in 55% yield (246.4 mg) according to the general procedure. The substrate was isolated through silica gel column chromatography (PE/EA = 10/1) as a pale yellow solid (mp 118–120 °C). ^{1}H NMR (400 MHz, $CDCl_3$) δ 8.29 (d, *J* = 7.6 Hz, 2H), 8.20 (d, *J* = 7.6 Hz, 2H), 7.24 – 7.17 (m, 1H), 7.15 – 6.99 (m, 3H), 6.98 – 6.87 (m, 2H), 4.67 (s, 2H), 2.38 (s, 6H); ^{13}C NMR (100 MHz, $CDCl_3$) δ 150.6, 142.5, 140.3, 132.8, 129.4, 128.1, 127.9, 127.0, 126.7, 124.1, 121.5, 120.9, 88.6, 84.4, 80.4, 69.4, 43.7, 21.1; IR (neat): 3108, 2920, 2231, 1636, 1532, 1378, 1347, 1178, 764, 615 cm^{-1}; HRESIMS Calcd for $[C_{23}H_{18}N_2NaO_4S_2]^+$ (M + Na$^+$) 473.0600, found 473.0599.

(E)-*N*-((2,6-dimethylphenyl)ethynyl)-4-nitro-*N*-(5-phenylpent-4-en-2-yn-1-yl)benzenesulfonamide (1q)

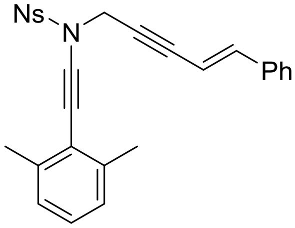

1q

Compound **1q** was prepared in 61% yield (286.9 mg) according to the general procedure. The substrate was isolated through silica gel column chromatography (PE/EA = 10/1) as a pale yellow solid (mp 149–151 °C). ^{1}H NMR (400 MHz, $CDCl_3$) δ 8.34 (d, *J* = 6.4 Hz, 2H), 8.20 (d, *J* = 6.0 Hz, 2H), 7.39 – 7.19 (m, 5H), 7.15 – 6.95 (m, 3H), 6.62 (d, *J* = 16.0 Hz, 1H), 5.86 (d, *J* = 16.0 Hz, 1H), 4.61 (s, 2H), 2.38 (s, 6H); ^{13}C NMR (100 MHz, $CDCl_3$) δ 150.6, 142.9, 142.6, 140.1, 135.2, 129.4, 129.1, 128.7, 127.8, 126.7, 126.2, 124.0, 121.5, 106.0, 88.6, 86.2, 82.4, 69.4, 43.6, 21.0; IR (neat): 3106, 2935, 2245, 1607, 1532, 1374, 1349, 1173, 767, 612 cm^{-1}; HRESIMS Calcd for $[C_{27}H_{22}N_2NaO_4S]^+$ (M + Na^+) 493.1192, found 493.1196.

***N*-(3-(4-methoxyphenyl)prop-2-yn-1-yl)-4-nitro-*N*-(o-tolylethynyl)benzenesulfonamide (1r)**

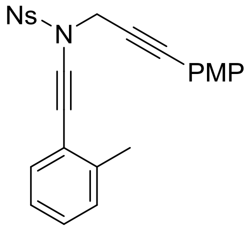

1r

Compound **1r** was prepared in 84% yield (385.0 mg) according to the general procedure. The substrate was isolated through silica gel column chromatography (PE:EA = 10:1) as a pale yellow solid (mp 140–142 °C). ^{1}H NMR (500 MHz, $CDCl_3$) δ 8.25 (d, *J* = 7.2 Hz, 2H), 8.20 (d, *J* = 7.2 Hz, 2H), 7.40 – 7.35 (m, 1H), 7.24 – 7.16 (m, 2H), 7.16 – 7.10 (m, 1H), 7.02 (d, *J* = 7.2 Hz, 2H), 6.74 (d, *J* = 6.8 Hz, 2H), 4.63 (s, 2H), 3.77 (s, 3H), 2.36 (s,

3H); ^{13}C NMR (125 MHz, $CDCl_3$) δ 160.1, 150.4, 142.7, 140.2, 132.9, 131.9, 129.4, 128.5, 125.6, 123.9, 121.6, 114.0, 113.2, 87.2, 84.6, 79.2, 70.4, 55.2, 43.7, 20.6; IR (neat): 3106, 2927, 2236, 1606, 1532, 1376, 1348, 1177, 756, 610 cm^{-1}; HRESIMS Calcd for $[C_{25}H_{20}N_2NaO_5S]^+$ (M + Na^+) 483.0985, found 483.0988.

***N*-(3-(4-methoxyphenyl)prop-2-yn-1-yl)-4-nitro-*N*-(phenylethynyl)benzenesulfonamide (1s)**

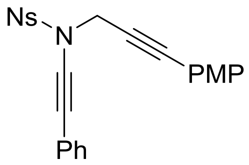

1s

Compound **1s** was prepared in 79% yield (354.5 mg) according to the general procedure. The substrate was isolated through silica gel column chromatography (PE/EA = 10/1) as a pale yellow oli. ^{1}H NMR (400 MHz, $CDCl_3$) δ 8.25 (d, *J* = 9.2 Hz, 2H), 8.19 (d, *J* = 9.2 Hz, 2H), 7.44 – 7.36 (m, 2H), 7.35 – 7.26 (m, 3H), 7.04 (d, *J* = 8.8 Hz, 2H), 6.74 (d, *J* = 8.8 Hz, 2H), 4.61 (s, 2H), 3.77 (s, 3H); ^{13}C NMR (100 MHz, $CDCl_3$) δ 160.0, 150.4, 142.6, 132.9, 131.7, 129.4, 128.4, 128.3, 123.9, 121.7, 113.9, 113.2, 87.2, 80.9, 79.1, 71.5, 55.2, 43.6; IR (neat): 3104, 2927, 2239, 1606, 1532, 1377, 1349, 1177, 762, 611 cm^{-1}; HRESIMS Calcd for $[C_{24}H_{18}N_2NaO_5S]^+$ (M + Na^+) 469.0829, found 469.0835.

***N*-(cyclopropylethynyl)-*N*-(3-(4-methoxyphenyl)prop-2-yn-1-yl)-4-nitrobenzenesulfonamide (1t)**

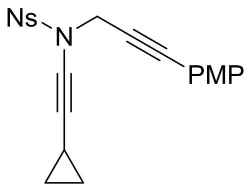

1t

Compound **1t** was prepared in 57% yield (234.2 mg) according to the general procedure. The substrate was isolated through silica gel column chromatography (PE/EA = 10/1) as a yellow solid (mp 118–120 °C). ^{1}H NMR (400 MHz, $CDCl_3$) δ 8.26 (d, *J* = 8.8 Hz, 2H), 8.15 (d, *J* = 8.8 Hz, 2H), 7.03 (d, *J* = 8.4 Hz, 2H), 6.76 (d, *J* = 8.8 Hz, 2H), 4.47 (s, 2H),

3.79 (s, 3H), 1.39 – 1.30 (m, 1H), 0.93 – 0.75 (m, 2H), 0.73 – 0.57 (m, 2H); ^{13}C NMR (100 MHz, $CDCl_3$) δ 160.0, 150.3, 142.7, 132.9, 129.4, 123.8, 113.9, 113.4, 86.9, 79.4, 76.1, 67.5, 55.3, 43.5, 9.0, -0.9; IR (neat): 3106, 2935, 2245, 1607, 1532, 1374, 1349, 1173, 767, 612 cm^{-1}; HRESIMS Calcd for $[C_{21}H_{18}N_2NaO_5S]^+$ (M + Na$^+$) 433.0829, found 433.0831.

***N*-((2-cyclohexylnaphthalen-1-yl)ethynyl)-*N*-(3-(4-methoxyphenyl)prop-2-yn-1-yl)-4-nitrobenzenesulfonamide (1x)**

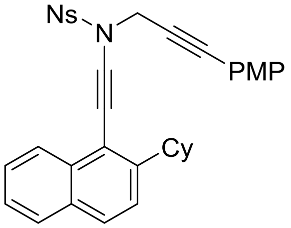

1x

Compound **1x** was prepared in 50% yield (287.6 mg) according to the general procedure. The substrate was isolated through silica gel column chromatography (PE/EA = 10/1) as a yellow solid (mp 130–131 ºC). ^{1}H NMR (400 MHz, $CDCl_3$) δ 8.30 – 8.16 (m, 5H), 7.77 (d, *J* = 8.8 Hz, 2H), 7.45 – 7.38 (m, 2H), 7.37 – 7.31 (m, 1H), 7.04 (d, *J* = 8.8 Hz, 2H), 6.73 (d, *J* = 8.8 Hz, 2H), 4.74 (s, 2H), 3.77 (s, 3H), 3.36 – 3.26 (m, 1H), 1.94 – 1.76 (m, 4H), 1.76 – 1.65 (m, 1H), 1.61 – 1.35 (m, 5H); ^{13}C NMR (100 MHz, $CDCl_3$) δ 160.2, 150.5, 148.4, 142.9, 133.5, 133.0, 131.6, 129.5, 128.6, 128.0, 126.9, 125.9, 125.6, 124.0, 123.9, 116.8, 114.0, 113.2, 89.4, 87.4, 79.3, 68.7, 55.3, 43.7, 42.9, 33.5, 26.8, 26.1; IR (neat): 3108, 2926, 2233, 1637, 1532, 1378, 1347, 1176, 751, 611 cm^{-1}; HRESIMS Calcd for $[C_{34}H_{30}N_2NaO_5S]^+$ (M + Na$^+$) 601.1768, found 601.1764.

***N*-(but-2-yn-1-yl)-*N*-((2,6-dimethylphenyl)ethynyl)-4-nitrobenzenesulfonamide (1y)**

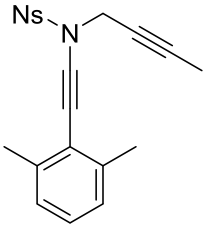

1y

Compound **1y** was prepared in 79% yield (302.2 mg) according to the general procedure. The substrate was isolated through silica gel column chromatography (PE/EA = 20/1) as a yellow oli. ^{1}H NMR (400 MHz, $CDCl_3$) δ 8.37 (d, *J* = 8.8 Hz, 2H), 8.20 (d, *J* = 8.8 Hz, 2H), 7.14 – 7.07 (m, 1H), 7.03 (d, *J* = 7.6 Hz, 2H), 4.40 (d, *J* = 2.4 Hz, 2H), 2.37 (s, 6H), 1.60 (t, *J* = 2.4 Hz, 3H); ^{13}C NMR (100 MHz, $CDCl_3$) δ 150.6, 142.9, 140.0, 129.5, 127.8, 126.7, 123.9, 121.7, 88.7, 83.5, 70.8, 69.4, 43.2, 20.9, 3.3; IR (neat): 3105, 2921, 2233, 1607, 1532, 1373, 1348, 1178, 740, 610 cm^{-1}; HRESIMS Calcd for $[C_{20}H_{18}N_2NaO_4S]^+$ (M + Na^+) 405.0879, found 405.0888.

1.2.2 General procedure for the synthesis of 1,2,5-oxadiazoles 2 (2a-2c)[4,5]

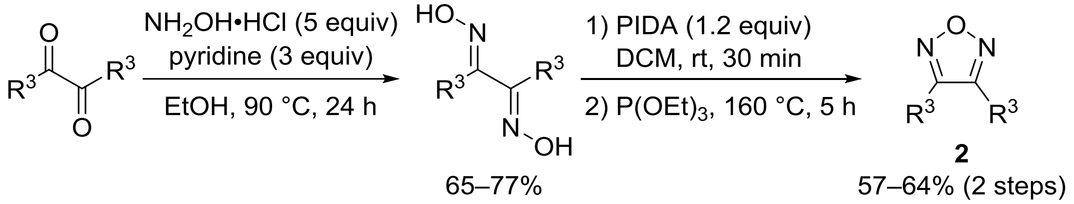

To a solution of 1,2-diketone (5.0 mmol) in EtOH (20 mL) were added pyridine (15 mmol, 1.2 mL) and NH_2OH·HCl (25 mmol, 1.737 g) at room temperature, and the mixture was stirred at 90 ℃ for 24 h. The progress of the reaction was monitored by TLC. Upon completion, the reaction was cooled to room temperature, quenched with 1 N aqueous HCl and extracted with ethyl acetate. Then combined organic layers was dried over Mg_2SO_4, filtered and concentrated under reduced pressure. The residue was purified by column chromatography on silica gel (PE/EA = 2/1) to give the desired oxime in 65–77% yield.

To a solution of above oxime (2.0 mmol) in DCM (5 mL) was added PIDA (2.4 mmol, 773 mg). The reaction mixture was stirred at room temperature and the progress of the reaction was monitored by TLC. The reaction typically took 30 min. Upon completion, the reaction was quenched with saturated $NaHCO_3$ and extracted with DCM. The combined organic layers was dried over Mg_2SO_4, filtered and concentrated under reduced pressure. The residue was purified by column chromatography on silica gel (PE/EA = 10/1) to afford the desired intermediate. The solution of above intermediate in $P(OEt)_3$ (3 mL) was heated at 160 ℃ for 5 h. Upon completion, the mixture was cooled to room temperature, and the resulting mixture was directly purified by column chromatography

on silica gel (PE/EA = 20/1) to afford the desired 1,2,5-oxadiazole **2** (57–64% yield, 2 steps).

3,4-bis(4-methoxyphenyl)-1,2,5-oxadiazole (2a)

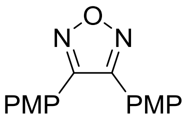

2a

Compound **2a** was prepared in 37% yield for 3 steps (522.1 mg) according to the general procedure. The substrate was isolated through silica gel column chromatography (PE/EA = 20/1) as a white solid (mp 128–130 °C). ^{1}H NMR (400 MHz, $CDCl_3$) δ 7.45 (d, *J* = 8.8 Hz, 4H), 6.92 (d, *J* = 8.8 Hz, 4H), 3.82 (s, 6H); ^{13}C NMR (100 MHz, $CDCl_3$) δ 161.1, 152.5, 130.1, 117.9, 114.2, 55.2; IR (neat): 2973, 2844, 2050, 1615, 1508, 1456, 1258, 1027, 764, 558 cm^{-1}; HRESIMS Calcd for $[C_{16}H_{15}N_2O_3]^+$ (M + H$^+$) 283.1077, found 283.1081.

3,4-diphenyl-1,2,5-oxadiazole (2b)

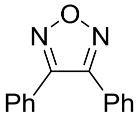

2b

Compound **2b** was prepared in 49% yield for 3 steps (544.1 mg) according to the general procedure. The substrate was isolated through silica gel column chromatography (PE/EA = 20/1) as a white solid (mp 94–96 °C). ^{1}H NMR (400 MHz, $CDCl_3$) δ 7.62 – 7.50 (m, 4H), 7.50 – 7.45 (m, 2H), 7.44 – 7.34 (m, 4H); ^{13}C NMR (100 MHz, $CDCl_3$) δ 153.0, 130.4, 128.9, 128.8, 125.7; IR (neat): 2983, 2823, 2068, 1635, 1444, 1368, 992, 893, 766, 698 cm^{-1}; HRESIMS Calcd for $[C_{14}H_{11}N_2O]^+$ (M + H$^+$) 223.0866, found 223.0870.

3,4-di-*p*-tolyl-1,2,5-oxadiazole (2c)

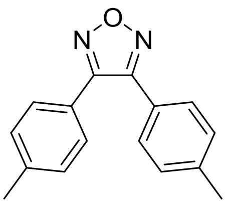

2c

Compound **2c** was prepared in 45% yield for 3 steps (563.1 mg) according to the general procedure. The substrate was isolated through silica gel column chromatography (PE/EA = 20/1) as a white solid (mp 89–91 °C). ^{1}H NMR (400 MHz, $CDCl_3$) δ 7.42 (d, *J* = 8.0 Hz, 4H), 7.23 (d, *J* = 8.0 Hz, 4H), 2.40 (s, 6H); ^{13}C NMR (100 MHz, $CDCl_3$) δ 153.0, 140.6, 129.6, 128.7, 122.9, 21.4; IR (neat): 3036, 2922, 1913, 1613, 1506, 1295, 989, 828, 758, 583 cm^{-1}; HRESIMS Calcd for $[C_{16}H_{15}N_2O]^+$ (M + H$^+$) 251.1179, found 251.1184.

1.3 General Procedure for the Synthesis of 1,2,5-Oxadiazines 3

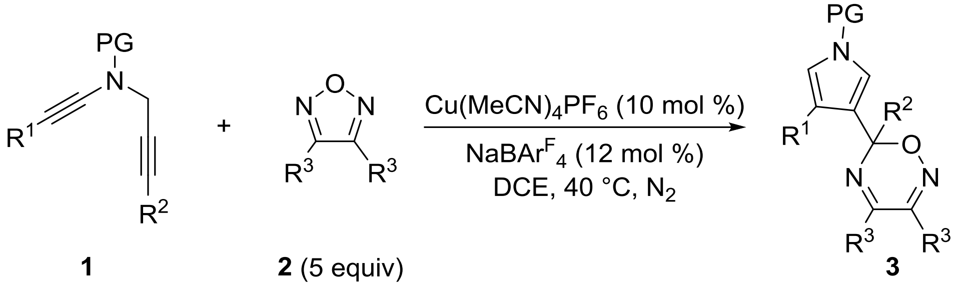

1,2,5-oxadiazole **2** (0.5 mmol), $NaBAr^F_4$ (0.012 mmol, 2.7 mg) and $Cu(MeCN)_4PF_6$ (0.01 mmol, 3.7 mg) were added in this order to the *N*-propargyl ynamide **1** (0.1 mmol) in DCE (4 mL) at room temperature. The reaction mixture was stirred at 40 ℃ and the progress of the reaction was monitored by TLC. Upon completion, the mixture was concentrated under reduced pressure and the residue was purified by column chromatography on silica gel (dichloromethane/hexane) to afford the desired product **3**.

6-(4-(2,6-dimethylphenyl)-1-((4-nitrophenyl)sulfonyl)-1*H*-pyrrol-3-yl)-3,4,6-tris(4-methoxyphenyl)-6*H*-1,2,5-oxadiazine (3a)

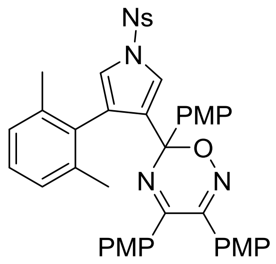

3a

Compound **3a** was prepared in 72% yield (54.5 mg) according to the general procedure. The product was isolated through silica gel column chromatography (DCM/PE = 2/1) as a pale yellow solid (mp 175–177 ^{o}C). ^{1}H NMR (400 MHz, $CDCl_3$) δ 8.09 (d, *J* = 8.8 Hz, 2H), 7.88 (d, *J* = 8.8 Hz, 2H), 7.55 (d, *J* = 2.4 Hz, 1H), 7.21 – 7.02 (m, 7H), 6.95 (d, *J* = 7.6 Hz, 1H), 6.88 – 6.82 (m, 2H), 6.75 – 6.67 (m, 4H), 6.65 (d, *J* = 8.8 Hz, 2H), 3.78 (s, 3H), 3.76 (s, 3H), 3.73 (s, 3H), 1.60 (s, 3H), 1.47 (s, 3H); ^{13}C NMR (100 MHz, $CDCl_3$) δ 161.3, 160.7, 159.4, 155.0, 153.4, 150.4, 144.2, 138.3, 137.9, 132.3, 131.5, 131.1, 130.6, 129.1, 128.8, 128.4, 127.8, 127.7, 127.6, 126.8, 126.3, 124.9, 124.3, 120.7, 120.0, 113.7, 113.2, 113.0, 89.6, 55.2(3), 55.2(1), 55.2(0), 20.9, 20.0; IR (neat): 2935, 2839, 1607, 1510, 1253, 1181, 1070, 834, 740, 571 cm^{-1}; HRESIMS Calcd for $[C_{42}H_{37}N_4O_8S]^+$ (M + H^+) 757.2327, found 757.2333.

6-(4-(2,6-dimethylphenyl)-1-tosyl-1*H*-pyrrol-3-yl)-3,4,6-tris(4-methoxyphenyl)-6*H*-1,2,5-oxadiazine (3b)

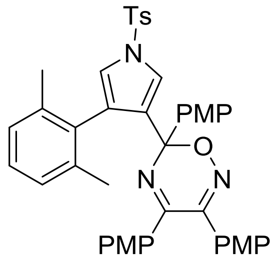

3b

Compound **3b** was prepared in 66% yield (48.1 mg) according to the general procedure. The product was isolated through silica gel column chromatography (DCM/PE = 2/1) as a pale yellow solid (mp 146–148 ^{o}C). ^{1}H NMR (400 MHz, $CDCl_3$) δ 7.63 (d, *J* = 8.4 Hz, 2H), 7.50 (d, *J* = 2.4 Hz, 1H), 7.20 – 7.10 (m, 6H), 7.09 – 7.03 (m, 3H), 6.91 (d, *J* = 7.6 Hz, 1H), 6.86 – 6.78 (m, 2H), 6.73 – 6.68 (m, 4H), 6.62 (d, *J* = 8.8 Hz, 2H), 3.78 (s, 3H),

3.74 (s, 3H), 3.73 (s, 3H), 2.36 (s, 3H), 1.61 (s, 3H), 1.54 (s, 3H); ^{13}C NMR (100 MHz, $CDCl_3$) δ 161.2, 160.5, 159.2, 155.0, 153.3, 144.7, 138.6, 138.4, 136.1, 132.9, 131.7, 130.6, 129.9, 129.7, 129.2, 128.8, 128.0, 127.3, 127.0, 126.6, 126.5, 126.2, 125.2, 120.5, 119.8, 113.6, 113.1, 112.8, 89.8, 55.3, 55.2, 21.5, 20.8, 20.2; IR (neat): 2923, 2850, 1606, 1510, 1252, 1173, 1068, 833, 674, 566 cm^{-1}; HRESIMS Calcd for $[C_{43}H_{40}N_3O_6S]^+$ (M + H^+) 726.2632, found 726.2642.

6-(1-((4-bromophenyl)sulfonyl)-4-(2,6-dimethylphenyl)-1*H*-pyrrol-3-yl)-3,4,6-tris(4-methoxyphenyl)-6*H*-1,2,5-oxadiazine (3c)

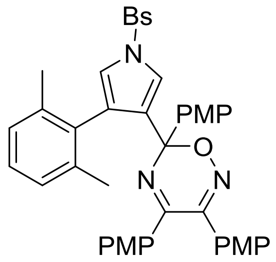

3c

Compound **3c** was prepared in 66% yield (52.1 mg) according to the general procedure. The product was isolated through silica gel column chromatography (DCM/PE = 2/1) as a pale yellow solid (mp 158–160 ºC). ^{1}H NMR (400 MHz, $CDCl_3$) δ 7.58 (d, *J* = 8.8 Hz, 2H), 7.51 (d, *J* = 2.8 Hz, 1H), 7.44 (d, *J* = 8.8 Hz, 2H), 7.20 – 7.12 (m, 4H), 7.11 – 7.04 (m, 3H), 6.93 (d, *J* = 7.6 Hz, 1H), 6.84 (d, *J* = 7.6 Hz, 1H), 6.81 (d, *J* = 2.4 Hz, 1H), 6.75 – 6.68 (m, 4H), 6.67 – 6.61 (m, 2H), 3.78 (s, 3H), 3.76 (s, 3H), 3.73 (s, 3H), 1.61 (s, 3H), 1.50 (s, 3H); ^{13}C NMR (100 MHz, $CDCl_3$) δ 161.2, 160.6, 159.3, 155.0, 153.3, 138.5, 138.2, 137.9, 132.6, 132.5, 131.6, 130.6, 130.5, 129.2, 128.8, 128.0, 127.9, 127.7, 127.4, 126.7, 126.3, 125.1, 120.6, 119.9, 113.7, 113.1, 112.9, 89.7, 55.2(4), 55.2(2), 20.8, 20.1; IR (neat): 2933, 2838, 1607, 1510, 1253, 1175, 1069, 834, 745, 639 cm^{-1}; HRESIMS Calcd for $[C_{42}H_{37}BrN_3O_6S]^+$ (M + H^+) 790.1581, found 790.1583.

6-(4-(2,6-dimethylphenyl)-1-((4-methoxyphenyl)sulfonyl)-1*H*-pyrrol-3-yl)-3,4,6-tris(4-methoxyphenyl)-6*H*-1,2,5-oxadiazine (3d)

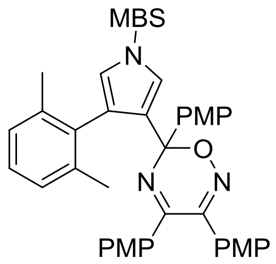

3d

Compound **3d** was prepared in 61% yield (45 mg) according to the general procedure. The product was isolated through silica gel column chromatography (DCM/PE = 3/1) as a pale yellow solid (mp 143–145 °C). ^{1}H NMR (400 MHz, $CDCl_3$) δ 7.69 (d, *J* = 8.8 Hz, 2H), 7.48 (d, *J* = 2.8 Hz, 1H), 7.18 – 7.12 (m, 4H), 7.09 – 7.01 (m, 3H), 6.92 (d, *J* = 7.6 Hz, 1H), 6.86 – 6.76 (m, 4H), 6.74 – 6.67 (m, 4H), 6.62 (d, *J* = 8.8 Hz, 2H), 3.81 (s, 3H), 3.79 (s, 3H), 3.74 (s, 3H), 3.73 (s, 3H), 1.62 (s, 3H), 1.54 (s, 3H); ^{13}C NMR (100 MHz, $CDCl_3$) δ 163.6, 161.1, 160.5, 159.1, 155.0, 153.3, 138.6, 138.4, 133.0, 131.7, 130.6, 130.4, 129.7, 129.2, 128.8, 128.0, 127.3, 126.9, 126.6, 126.2, 125.2, 120.4, 119.7, 114.3, 113.6, 113.1, 112.8, 89.7, 55.6, 55.3, 55.2(0), 55.1(8), 20.8, 20.2; IR (neat): 2931, 2839, 1607, 1510, 1253, 1168, 1070, 833, 681, 577 cm^{-1}; HRESIMS Calcd for $[C_{43}H_{40}N_3O_7S]^+$ (M + H$^+$) 742.2581, found 742.2604.

6-(4-(4-fluoro-2,6-dimethylphenyl)-1-((4-nitrophenyl)sulfonyl)-1*H*-pyrrol-3-yl)-3,4,6-tris(4-methoxyphenyl)-6*H*-1,2,5-oxadiazine (3e)

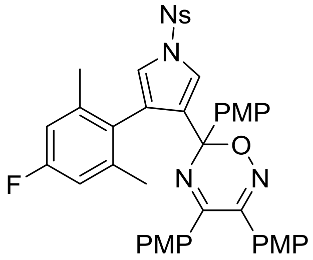

3e

Compound **3e** was prepared in 72% yield (55.7 mg) according to the general procedure. The product was isolated through silica gel column chromatography (DCM/PE = 2/1) as a pale yellow solid (mp 163–165 °C). ^{1}H NMR (400 MHz, $CDCl_3$) δ 8.15 (d, *J* = 8.8 Hz, 2H), 7.91 (d, *J* = 8.8 Hz, 2H), 7.50 (d, *J* = 2.4 Hz, 1H), 7.23 – 7.11 (m, 4H), 7.05 (d, *J* = 8.8 Hz, 2H), 6.85 (d, *J* = 2.4 Hz, 1H), 6.79 – 6.61 (m, 7H), 6.58 – 6.49 (m, 1H), 3.79 (s,

3H), 3.76 (s, 3H), 3.74 (s, 3H), 1.61 (s, 3H), 1.50 (s, 3H); ^{13}C NMR (100 MHz, $CDCl_3$) δ 162.1 (d, *J* = 244.0 Hz), 161.4, 160.8, 159.4, 155.1, 153.5, 150.4, 144.2, 140.8 (d, *J* = 12.0 Hz), 140.4 (d, *J* = 8.0 Hz), 131.4, 131.2, 130.5, 129.1, 128.7, 128.1 (d, *J* = 2.0 Hz), 127.8, 127.7, 127.5, 124.8, 124.4, 120.7, 120.2, 113.7, 113.3, 113.2 (d, *J* = 21.0 Hz), 113.0, 112.8, 89.5, 55.3, 55.2(4), 55.2(3), 21.0, 20.3; ^{19}F NMR (376 MHz, C_6D_6) δ -116.2; IR (neat): 2922, 2851, 1607, 1511, 1253, 1180, 1092, 834, 741, 579 cm^{-1}; HRESIMS Calcd for $[C_{42}H_{36}FN_4O_8S]^+$ (M + H$^+$) 775.2232, found 775.2244.

6-(4-(4-chloro-2,6-dimethylphenyl)-1-((4-nitrophenyl)sulfonyl)-1*H*-pyrrol-3-yl)-3,4,6-tris(4-methoxyphenyl)-6*H*-1,2,5-oxadiazine (3f)

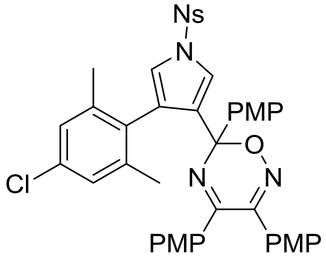

3f

Compound **3f** was prepared in 76% yield (60.4 mg) according to the general procedure. The product was isolated through silica gel column chromatography (DCM/PE = 2/1) as a pale yellow solid (mp 160–162 ºC). ^{1}H NMR (400 MHz, $CDCl_3$) δ 8.16 (d, *J* = 8.8 Hz, 2H), 7.91 (d, *J* = 8.8 Hz, 2H), 7.47 (d, *J* = 2.4 Hz, 1H), 7.20 (d, *J* = 8.8 Hz, 2H), 7.13 (d, *J* = 8.4 Hz, 2H), 7.04 (d, *J* = 8.8 Hz, 2H), 6.92 (s, 1H), 6.85 (d, *J* = 2.4 Hz, 1H), 6.79 (s, 1H), 6.77 – 6.61 (m, 6H), 3.80 (s, 3H), 3.76 (s, 3H), 3.75 (s, 3H), 1.61 (s, 3H), 1.53 (s, 3H); ^{13}C NMR (100 MHz, $CDCl_3$) δ 161.3, 160.8, 159.5, 155.0, 153.5, 150.5, 144.2, 140.3, 139.9, 132.9, 131.4, 131.1, 130.9, 130.5, 129.2, 128.7, 127.8, 127.3, 126.6, 126.2, 124.8, 124.5, 120.8, 119.9, 113.7, 113.3, 113.1, 89.5, 55.3, 55.2(4), 55.2(2), 20.7, 20.2; IR (neat): 2924, 2850, 1607, 1510, 1253, 1181, 1072, 834, 740, 574 cm^{-1}; HRESIMS Calcd for $[C_{42}H_{36}ClN_4O_8S]^+$ (M + H$^+$) 791.1937, found 791.1945.

6-(4-(4-bromo-2,6-dimethylphenyl)-1-((4-nitrophenyl)sulfonyl)-1*H*-pyrrol-3-yl)-3,4,6-tris(4-methoxyphenyl)-6*H*-1,2,5-oxadiazine (3g)

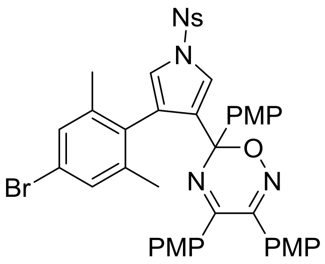

3g

Compound **3g** was prepared in 72% yield (60.3 mg) according to the general procedure. The product was isolated through silica gel column chromatography (DCM/PE = 2/1) as a pale yellow solid (mp 143–145 °C). ^{1}H NMR (400 MHz, $CDCl_3$) δ 8.17 (d, *J* = 8.4 Hz, 2H), 7.91 (d, *J* = 8.4 Hz, 2H), 7.45 (d, *J* = 2.4 Hz, 1H), 7.20 (d, *J* = 8.4 Hz, 2H), 7.12 (d, *J* = 8.4 Hz, 2H), 7.09 – 6.99 (m, 3H), 6.94 (s, 1H), 6.85 (d, *J* = 2.4 Hz, 1H), 6.74 (d, *J* = 8.8 Hz, 2H), 6.71 – 6.59 (m, 4H), 3.80 (s, 3H), 3.75 (s, 3H), 3.74 (s, 3H), 1.61 (s, 3H), 1.54 (s, 3H); ^{13}C NMR (100 MHz, $CDCl_3$) δ 161.3, 160.8, 159.5, 155.0, 153.5, 150.5, 144.2, 140.6, 140.2, 131.5, 131.4, 131.1, 130.4, 129.5, 129.2, 129.1, 128.7, 127.8, 127.7, 127.3, 124.8, 124.4, 121.3, 120.9, 119.8, 113.7, 113.3, 113.1, 89.5, 55.3, 55.2(4), 55.2(1), 20.6, 20.1; IR (neat): 2924, 2850, 1607, 1510, 1253, 1181, 1071, 834, 740, 574 cm^{-1}; HRESIMS Calcd for $[C_{42}H_{36}BrN_4O_8S]^+$ (M + H$^+$) 835.1432, found 835.1436.

6-(4-mesityl-1-((4-nitrophenyl)sulfonyl)-1*H*-pyrrol-3-yl)-3,4,6-tris(4-methoxyphenyl)-6*H*-1,2,5-oxadiazine (3h)

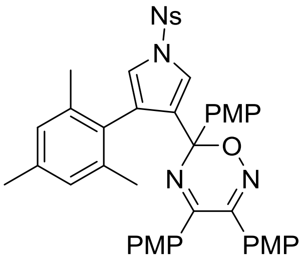

3h

Compound **3h** was prepared in 64% yield (49.0 mg) according to the general procedure. The product was isolated through silica gel column chromatography (DCM/PE = 3/1) as a pale yellow solid (mp 164–166 °C). ^{1}H NMR (400 MHz, $CDCl_3$) δ 8.09 (d, *J* = 8.0 Hz, 2H), 7.87 (d, *J* = 7.6 Hz, 2H), 7.52 (s, 1H), 7.20 (d, *J* = 8.0 Hz, 2H), 7.13 (d, *J* = 7.6 Hz, 2H), 7.06 (d, *J* = 8.0 Hz, 2H), 6.85 – 6.56 (m, 9H), 3.78 (s, 3H), 3.77 (s, 3H), 3.74 (s, 3H),

2.28 (s, 3H), 1.55 (s, 3H), 1.46 (s, 3H); ^{13}C NMR (100 MHz, $CDCl_3$) δ 161.2, 160.7, 159.3, 154.9, 153.3, 150.3, 144.2, 138.1, 137.6, 137.1, 131.5, 131.4, 130.6, 129.3, 129.1, 128.8, 128.5, 127.9, 127.6, 127.1, 124.9, 124.3, 120.8, 120.1, 113.7, 113.1, 113.0, 89.6, 55.3, 55.2, 21.1, 20.7, 20.0; IR (neat): 2924, 2852, 1607, 1511, 1253, 1181, 1092, 835, 740, 577 cm^{-1}; HRESIMS Calcd for $[C_{43}H_{39}N_4O_8S]^+$ (M + H$^+$) 771.2483, found 771.2485.

6-(4-(4-(benzyloxy)-2,6-dimethylphenyl)-1-((4-nitrophenyl)sulfonyl)-1*H*-pyrrol-3-yl)-3,4,6-tris(4-methoxyphenyl)-6*H*-1,2,5-oxadiazine (3i)

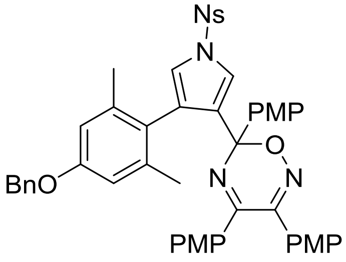

3i

Compound **3i** was prepared in 70% yield (60.2 mg) according to the general procedure. The product was isolated through silica gel column chromatography (DCM/PE = 3/1) as a pale yellow solid (mp 158–160 °C). ^{1}H NMR (400 MHz, $CDCl_3$) δ 8.10 (d, *J* = 8.4 Hz, 2H), 7.88 (d, *J* = 8.4 Hz, 2H), 7.52 – 7.32 (m, 6H), 7.20 – 7.15 (m, 4H), 7.06 (d, *J* = 8.4 Hz, 2H), 6.83 (s, 1H), 6.75 – 6.56 (m, 7H), 6.49 (s, 1H), 5.03 (s, 2H), 3.77 (s, 6H), 3.72 (s, 3H), 1.57 (s, 3H), 1.46 (s, 3H); ^{13}C NMR (100 MHz, $CDCl_3$) δ 161.3, 160.7, 159.3, 158.1, 155.0, 153.4, 150.3, 144.2, 139.8, 139.3, 137.3, 131.5(4), 131.4(7), 130.6, 129.1, 128.8, 128.6, 128.2, 127.9, 127.7, 127.4, 125.0, 124.9, 124.3, 120.6, 120.4, 113.7, 113.2, 113.1, 113.0, 112.8, 89.6, 69.7, 55.3, 55.2, 21.1, 20.4; IR (neat): 2926, 2853, 1607, 1510, 1251, 1180, 1059, 834, 739, 579 cm^{-1}; HRESIMS Calcd for $[C_{49}H_{42}N_4NaO_9S]^+$ (M + Na$^+$) 885.2565, found 885.2552.

6-(4-(2,6-dimethylphenyl)-1-((4-nitrophenyl)sulfonyl)-1*H*-pyrrol-3-yl)-3,4-bis(4-methoxyphenyl)-6-phenyl-6*H*-1,2,5-oxadiazine (3j)

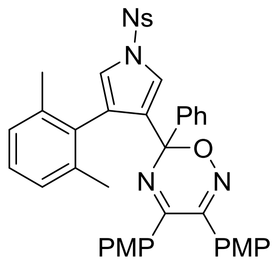

3j

Compound **3j** was prepared in 55% yield (39.8 mg) according to the general procedure. The product was isolated through silica gel column chromatography (DCM/PE = 3/1) as a pale yellow solid (mp 180–182 °C). ^{1}H NMR (400 MHz, $CDCl_3$) δ 8.11 (d, *J* = 8.4 Hz, 2H), 7.88 (d, *J* = 8.8 Hz, 2H), 7.58 (d, *J* = 2.4 Hz, 1H), 7.26 – 7.23 (m, 2H), 7.18 – 7.08 (m, 6H), 7.06 (d, *J* = 8.4 Hz, 2H), 6.93 (d, *J* = 7.6 Hz, 1H), 6.89 – 6.83 (m, 2H), 6.75 – 6.66 (m, 4H), 3.79 (s, 3H), 3.77 (s, 3H), 1.54 (s, 3H), 1.46 (s, 3H); ^{13}C NMR (100 MHz, $CDCl_3$) δ 161.3, 160.8, 155.1, 153.5, 150.4, 144.2, 139.2, 138.4, 137.9, 132.1, 130.9, 130.6, 129.1, 128.4, 128.1, 127.8, 127.7, 127.6, 127.4, 126.8, 126.3, 124.8, 124.4, 120.9, 120.0, 113.7, 113.2, 89.7, 55.3, 55.2, 20.8, 20.0; IR (neat): 2924, 2853, 1607, 1533, 1254, 1181, 1071, 836, 740, 571 cm^{-1}; HRESIMS Calcd for $[C_{41}H_{35}N_4O_7S]^+$ (M + H$^+$) 727.2221, found 727.2226.

6-(4-(2,6-dimethylphenyl)-1-((4-nitrophenyl)sulfonyl)-1*H*-pyrrol-3-yl)-3,4-bis(4-methoxyphenyl)-6-(*p*-tolyl)-6*H*-1,2,5-oxadiazine (3k)

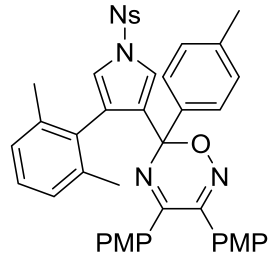

3k

Compound **3k** was prepared in 73% yield (57.2 mg) according to the general procedure. The product was isolated through silica gel column chromatography (DCM/PE = 3/1) as a pale yellow solid (mp 163–165 °C). ^{1}H NMR (400 MHz, $CDCl_3$) δ 8.11 (d, *J* = 8.4 Hz, 2H), 7.89 (d, *J* = 8.8 Hz, 2H), 7.55 (d, *J* = 2.0 Hz, 1H), 7.20 – 7.01 (m, 7H), 6.95 – 6.90 (m, 3H), 6.85 – 6.80 (m, 2H), 6.73 – 6.80 (m, 4H), 3.78 (s, 3H), 3.76 (s, 3H), 2.25 (s, 3H),

1.58 (s, 3H), 1.45 (s, 3H); ^{13}C NMR (100 MHz, $CDCl_3$) δ 161.3, 160.7, 155.0, 153.4, 150.4, 144.2, 138.3, 137.9, 137.8, 136.2, 132.2, 131.2, 130.6, 129.2, 128.5, 128.3, 127.9, 127.7, 127.6, 127.3, 126.7, 126.3, 124.9, 124.3, 120.7, 119.9, 113.7, 113.1, 89.7, 55.3, 55.2, 21.1, 20.8, 20.0; IR (neat): 2924, 2840, 1607, 1533, 1349, 1181, 1070, 837, 741, 573 cm^{-1}; HRESIMS Calcd for $[C_{42}H_{37}N_4O_7S]^+$ (M + H^+) 741.2377, found 741.2367.

6-(4-(benzyloxy)phenyl)-6-(4-(2,6-dimethylphenyl)-1-((4-nitrophenyl)sulfonyl)-1*H*-pyrrol-3-yl)-3,4-bis(4-methoxyphenyl)-6*H*-1,2,5-oxadiazine (3l)

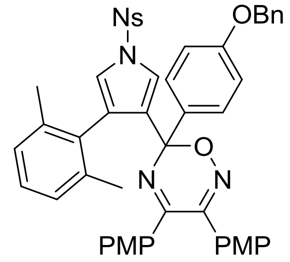

3l

Compound **3l** was prepared in 77% yield (63.9 mg) according to the general procedure. The product was isolated through silica gel column chromatography (DCM/PE = 3/1) as a pale yellow solid (mp 183–185 ^{o}C). ^{1}H NMR (400 MHz, $CDCl_3$) δ 8.08 (d, J = 8.0 Hz, 2H), 7.87 (d, J = 8.0 Hz, 2H), 7.56 (s, 1H), 7.43 – 7.27 (m, 5H), 7.21 – 6.99 (m, 7H), 6.94 – 6.79 (m, 3H), 6.77 – 6.61 (m, 6H), 5.00 (s, 2H), 3.77 (s, 6H), 1.56 (s, 3H), 1.43 (s, 3H); ^{13}C NMR (100 MHz, $CDCl_3$) δ 161.3, 160.7, 158.4, 155.1, 153.4, 150.3, 144.2, 138.4, 137.9, 136.8, 132.2, 131.7, 131.0, 130.6, 129.1, 128.8, 128.5, 128.4, 127.9, 127.8, 127.6, 127.4, 126.8, 126.3, 124.8, 124.3, 120.6, 119.9, 114.0, 113.7, 113.2, 89.6, 69.8, 55.3, 55.2, 20.8, 20.0; IR (neat): 2925, 2853, 1607, 1509, 1253, 1181, 1070, 836, 740, 571 cm^{-1}; HRESIMS Calcd for $[C_{48}H_{41}N_4O_8S]^+$ (M + H^+) 833.2640, found 833.2631.

6-(4-(2,6-dimethylphenyl)-1-((4-nitrophenyl)sulfonyl)-1*H*-pyrrol-3-yl)-6-(4-methoxy-3-methylphenyl)-3,4-bis(4-methoxyphenyl)-6*H*-1,2,5-oxadiazine (3m)

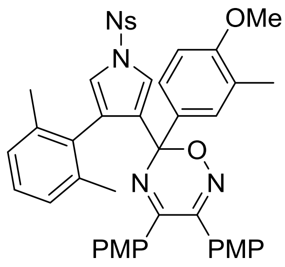

3m

Compound **3m** was prepared in 84% yield (64.7 mg) according to the general procedure. The product was isolated through silica gel column chromatography (DCM/PE = 3/1) as a pale yellow solid (mp 173–175 °C). ^{1}H NMR (400 MHz, $CDCl_3$) δ 8.11 (d, *J* = 8.8 Hz, 2H), 7.88 (d, *J* = 8.4 Hz, 2H), 7.55 (s, 1H), 7.21 – 7.01 (m, 6H), 6.95 (d, *J* = 7.2 Hz, 2H), 6.86 (d, *J* = 8.0 Hz, 2H), 6.77 – 6.64 (m, 4H), 6.55 (d, *J* = 8.4 Hz, 1H), 3.79 (s, 3H), 3.77 (s, 3H), 3.75 (s, 3H), 2.03 (s, 3H), 1.58 (s, 3H), 1.48 (s, 3H); ^{13}C NMR (100 MHz, $CDCl_3$) δ 161.3, 160.7, 157.5, 155.0, 153.3, 150.3, 144.3, 138.4, 137.9, 132.2, 131.4, 130.8, 130.6, 129.7, 129.2, 128.5, 127.9, 127.7, 127.6, 126.7, 126.3, 126.1, 125.6, 124.9, 124.3, 120.6, 119.9, 113.7, 113.2, 108.9, 89.6, 55.3, 55.2, 55.2, 20.8, 20.1, 16.2; IR (neat): 2923, 2852, 1606, 1507, 1251, 1181, 1070, 836, 739, 569 cm^{-1}; HRESIMS Calcd for $[C_{43}H_{38}N_4NaO_8S]^+$ (M + Na^+) 793.2303, found 793.2304.

6-(3,4-dimethoxyphenyl)-6-(4-(2,6-dimethylphenyl)-1-((4-nitrophenyl)sulfonyl)-1*H*-pyrrol-3-yl)-3,4-bis(4-methoxyphenyl)-6*H*-1,2,5-oxadiazine (3n)

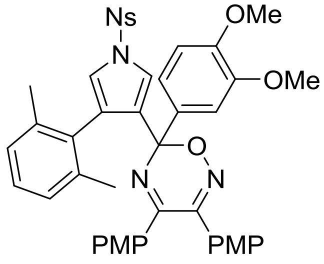

3n

Compound **3n** was prepared in 70% yield (55.1 mg) according to the general procedure. The product was isolated through silica gel column chromatography (PE/EA = 4/1) as a pale yellow solid (mp 145–147 °C). ^{1}H NMR (400 MHz, $CDCl_3$) δ 8.09 (d, *J* = 8.0 Hz, 2H), 7.87 (d, *J* = 8.4 Hz, 2H), 7.57 (s, 1H), 7.22 – 7.02 (m, 5H), 6.96 (d, *J* = 7.2 Hz, 1H), 6.89 (d, *J* = 7.6 Hz, 2H), 6.85 (s, 1H), 6.79 – 6.57 (m, 6H), 3.81 (s, 3H), 3.78 (s, 6H),

3.62 (s, 3H), 1.58 (s, 3H), 1.50 (s, 3H); ^{13}C NMR (100 MHz, $CDCl_3$) δ 161.4, 160.8, 155.1, 153.5, 150.4, 148.7, 147.8, 144.2, 138.5, 138.0, 132.3, 132.0, 131.0, 130.6, 129.1, 128.3, 127.8, 127.7, 127.6, 126.7, 126.3, 124.8, 124.3, 120.5, 120.4, 120.0, 113.7, 113.3, 110.6, 110.4, 89.5, 55.9, 55.6, 55.3, 55.2, 20.8, 20.1; IR (neat): 2923, 2851, 1606, 1513, 1256, 1182, 1072, 837, 740, 571 cm^{-1}; HRESIMS Calcd for $[C_{43}H_{39}N_4O_9S]^+$ (M + H^+) 787.2432, found 787.2416.

6-(benzo[*d*][1,3]dioxol-5-yl)-6-(4-(2,6-dimethylphenyl)-1-((4-nitrophenyl)sulfonyl)-1*H*-pyrrol-3-yl)-3,4-bis(4-methoxyphenyl)-6*H*-1,2,5-oxadiazine (3o)

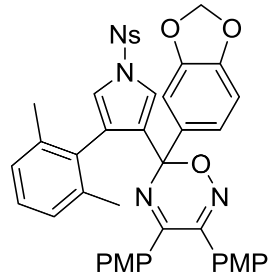

3o

Compound **3o** was prepared in 80% yield (61.8 mg) according to the general procedure. The product was isolated through silica gel column chromatography (DCM/PE = 3/1) as a pale yellow solid (mp 179–181 °C). ^{1}H NMR (400 MHz, $CDCl_3$) δ 8.12 (d, *J* = 8.0 Hz, 2H), 7.90 (d, *J* = 8.0 Hz, 2H), 7.54 (s, 1H), 7.19 – 7.02 (m, 5H), 6.97 (d, *J* = 6.8 Hz, 1H), 6.91 – 6.81 (m, 2H), 6.79 – 6.64 (m, 6H), 6.54 (d, *J* = 7.6 Hz, 1H), 5.87 (s, 2H), 3.78 (s, 3H), 3.77 (s, 3H), 1.64 (s, 3H), 1.51 (s, 3H); ^{13}C NMR (100 MHz, $CDCl_3$) δ 161.4, 160.8, 155.1, 153.4, 150.4, 147.4, 147.0, 144.2, 138.2, 137.9, 133.3, 132.2, 131.1, 130.6, 129.2, 128.4, 127.7, 126.8, 126.4, 124.8, 124.4, 121.4, 120.7, 120.1, 113.7, 113.2, 108.3, 107.2, 101.0, 89.5, 55.3, 55.2, 20.9, 20.1; IR (neat): 2921, 2849, 1607, 1533, 1252, 1182, 1031, 840, 740, 682 cm^{-1}; HRESIMS Calcd for $[C_{42}H_{35}N_4O_9S]^+$ (M + H^+) 771.2119, found 771.2091.

6-(4-(2,6-dimethylphenyl)-1-((4-nitrophenyl)sulfonyl)-1*H*-pyrrol-3-yl)-3,4-bis(4-methoxyphenyl)-6-(thiophen-2-yl)-6*H*-1,2,5-oxadiazine (3p)

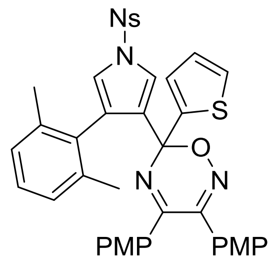

3p

Compound **3p** was prepared in 78% yield (56.9 mg) according to the general procedure. The product was isolated through silica gel column chromatography (DCM/PE = 3/1) as a pale yellow solid (mp 141–143 °C). ^{1}H NMR (400 MHz, $CDCl_3$) δ 8.09 (d, *J* = 8.8 Hz, 2H), 7.90 (d, *J* = 8.8 Hz, 2H), 7.62 (d, *J* = 2.0 Hz, 1H), 7.19 (d, *J* = 4.4 Hz, 1H), 7.16 – 7.02 (m, 5H), 6.98 (d, *J* = 7.2 Hz, 1H), 6.92 – 6.83 (m, 2H), 6.83 – 6.63 (m, 6H), 3.78 (s, 6H), 1.66 (s, 3H), 1.51 (s, 3H); ^{13}C NMR (100 MHz, $CDCl_3$) δ 161.5, 160.9, 154.8, 153.8, 150.4, 144.2, 143.2, 138.4, 137.8, 132.2, 130.8, 130.7, 129.3, 128.4, 127.7(0), 127.6(5), 127.4, 126.8, 126.5, 126.4, 124.7, 124.3, 120.7, 120.1, 113.7, 113.2, 88.1, 55.3, 55.2, 21.0, 19.8; IR (neat): 2923, 2852, 1607, 1533, 1256, 1182, 1069, 837, 740, 570 cm^{-1}; HRESIMS Calcd for $[C_{39}H_{32}N_4NaO_7S_2]^+$ (M + Na^+) 755.1605, found 755.1600.

(*E*)-6-(4-(2,6-dimethylphenyl)-1-((4-nitrophenyl)sulfonyl)-1*H*-pyrrol-3-yl)-3,4-bis(4-methoxyphenyl)-6-styryl-6*H*-1,2,5-oxadiazine (3q)

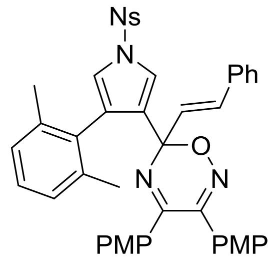

3q

Compound **3q** was prepared in 57% yield (43.1 mg) according to the general procedure. The product was isolated through silica gel column chromatography (DCM/PE = 3/1) as a pale yellow solid (mp 155–157 °C). ^{1}H NMR (400 MHz, $CDCl_3$) δ 8.13 (d, *J* = 8.4 Hz, 2H), 7.88 (d, *J* = 8.4 Hz, 2H), 7.49 (s, 1H), 7.25 – 7.07 (m, 10H), 7.05 – 6.91 (m, 3H), 6.81 – 6.66 (m, 4H), 6.46 (d, *J* = 16.0 Hz, 1H), 6.07 (d, *J* = 16.0 Hz, 1H), 3.81 (s, 3H), 3.77 (s, 3H), 1.99 (s, 3H), 1.81 (s, 3H); ^{13}C NMR (100 MHz, $CDCl_3$) δ 161.4, 160.8,

155.5, 154.2, 150.4, 144.3, 138.3, 137.6, 135.7, 133.0, 132.6, 130.6, 129.2, 129.1, 128.4, 128.3, 128.1, 127.8(1), 127.7(8), 127.7, 127.3, 126.8, 126.1, 125.0, 124.4, 121.5, 120.0, 113.8, 113.4, 88.6, 55.3, 55.2, 21.1, 21.0; IR (neat): 2918, 2854, 1646, 1533, 1252, 1182, 1068, 854, 638, 557 cm^{-1}; HRESIMS Calcd for $[C_{43}H_{37}N_4O_7S]^+$ (M + H^+) 753.2377, found 753.2358.

3,4,6-tris(4-methoxyphenyl)-6-(1-((4-nitrophenyl)sulfonyl)-4-(o-tolyl)-1*H*-pyrrol-3-yl)-6*H*-1,2,5-oxadiazine (3r)

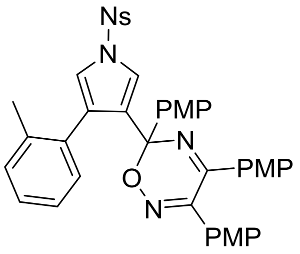

3r

Compound **3r** was prepared in 61% yield (45.0 mg) according to the general procedure. The substrate was isolated through silica gel column chromatography (DCM:PE = 3:1) as a pale yellow solid (mp 168–170 °C). ^{1}H NMR (500 MHz, $CDCl_3$) δ 8.05 (d, *J* = 7.2 Hz, 2H), 7.85 (d, *J* = 6.8 Hz, 2H), 7.49 (d, *J* = 1.6 Hz, 1H), 7.26 – 7.23 (m, 2H), 7.21 – 7.16 (m, 1H), 7.09 – 7.00 (m, 6H), 6.91 (d, *J* = 1.6 Hz, 1H), 6.83 (d, *J* = 6.0 Hz, 1H), 6.74 – 6.64 (m, 6H), 3.79 (s, 3H), 3.77 (s, 3H), 3.76 (s, 3H), 1.66 (s, 3H); ^{13}C NMR (125 MHz, $CDCl_3$) δ 161.3, 160.8, 159.4, 155.1, 153.6, 150.4, 144.1, 137.2, 132.5, 132.0, 131.6, 130.6, 130.5, 129.4, 129.2, 129.1, 128.8, 127.9, 127.7(3), 127.6(7), 124.9, 124.6, 124.4, 120.5, 120.3, 113.7, 113.2, 113.0, 89.8, 55.3, 55.2, 20.0; IR (neat): 3435, 2924, 2851, 1607, 1533, 1348, 1305, 1254, 741, 637 cm^{-1}; HRESIMS Calcd for $[C_{41}H_{34}N_4NaO_8S]^+$ (M + Na^+) 765.1990, found 765.1982.

6-(4-cyclopropyl-1-((4-nitrophenyl)sulfonyl)-1*H*-pyrrol-3-yl)-3,4,6-tris(4-methoxyphenyl)-6*H*-1,2,5-oxadiazine (3t)

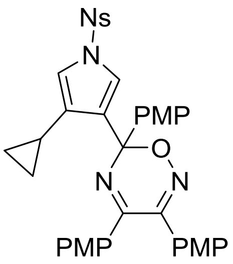

3t

Compound **3t** was prepared in 51% yield (35.4 mg) according to the general procedure. The product was isolated through silica gel column chromatography (DCM/PE = 2/1) as a pale yellow solid (mp 146–148 ^oC). ^1H NMR (400 MHz, $CDCl_3$) δ 8.02 (d, *J* = 8.4 Hz, 2H), 7.76 (d, *J* = 8.4 Hz, 2H), 7.53 (d, *J* = 8.4 Hz, 2H), 7.37 (d, *J* = 8.4 Hz, 2H), 7.24 (s, 1H), 7.11 (d, *J* = 8.0 Hz, 2H), 6.85 (d, *J* = 8.4 Hz, 2H), 6.81 – 6.67 (m, 4H), 6.63 (s, 1H), 3.80 (s, 3H), 3.79 (s, 3H), 3.78 (s, 3H), 1.78 – 1.63 (m, 1H), 0.75 – 0.46 (m, 2H), 0.42 – 0.12 (m, 2H); ^{13}C NMR (100 MHz, $CDCl_3$) δ 161.4, 160.8, 159.6, 155.2, 154.1, 150.3, 144.1, 132.8, 132.3, 130.5, 129.2, 128.9, 128.1, 127.7, 124.9, 124.4, 120.9, 117.0, 113.7, 113.5, 113.3, 90.3, 55.3, 55.2, 8.0, 7.8, 7.3; IR (neat): 2926, 2840, 1607, 1510, 1253, 1177, 1092, 834, 741, 576 cm^{-1}; HRESIMS Calcd for $[C_{37}H_{33}N_4O_8S]^+$ (M + H^+) 693.2014, found 693.2007.

6-(4-(2,6-dimethylphenyl)-1-((4-nitrophenyl)sulfonyl)-1*H*-pyrrol-3-yl)-6-(4-methoxyphenyl)-3,4-diphenyl-6*H*-1,2,5-oxadiazine (3u)

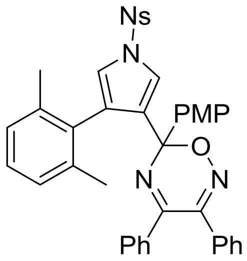

3u

Compound **3u** was prepared in 73% yield (51.2 mg) according to the general procedure. The product was isolated through silica gel column chromatography (DCM/PE = 3/1) as a pale yellow solid (mp 180–182 ^oC). ^1H NMR (400 MHz, $CDCl_3$) δ 8.14 (d, *J* = 8.4 Hz, 2H), 7.91 (d, *J* = 8.8 Hz, 2H), 7.52 (d, *J* = 2.0 Hz, 1H), 7.38 – 7.27 (m, 2H), 7.23 – 7.13 (m, 8H), 7.13 – 7.03 (m, 3H), 6.95 (d, *J* = 7.6 Hz, 1H), 6.91 – 6.80 (m, 2H), 6.66 (d, *J* =

8.8 Hz, 2H), 3.75 (s, 3H), 1.61 (s, 3H), 1.52 (s, 3H); ^{13}C NMR (100 MHz, $CDCl_3$) δ 159.4, 155.4, 154.2, 150.4, 144.2, 138.4, 138.0, 135.1, 132.3, 132.1, 131.0, 130.9, 130.4, 129.8, 128.8, 128.3(3), 128.2(9), 127.8, 127.7(4), 127.6(8), 127.6, 126.8, 126.4, 124.4, 120.7, 120.0, 113.0, 89.9, 55.3, 20.9, 20.1; IR (neat): 2923, 2851, 1608, 1533, 1251, 1183, 1070, 740, 682, 571 cm^{-1} HRESIMS Calcd for $[C_{40}H_{32}N_4NaO_6S]^+$ (M + Na^+) 719.1935, found 719.1927.

6-(4-(2,6-dimethylphenyl)-1-((4-nitrophenyl)sulfonyl)-1*H*-pyrrol-3-yl)-6-(4-methoxyphenyl)-3,4-di-*p*-tolyl-6*H*-1,2,5-oxadiazine (3v)

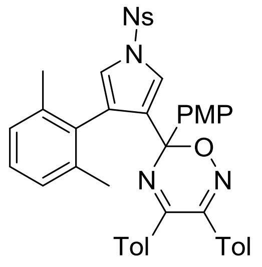

3v

Compound **3v** was prepared in 62% yield (53.7 mg) according to the general procedure. The product was isolated through silica gel column chromatography (DCM/PE = 3/1) as a pale yellow solid (mp 178–180 °C). ^{1}H NMR (400 MHz, $CDCl_3$) δ 8.08 (d, *J* = 8.8 Hz, 2H), 7.88 (d, *J* = 8.8 Hz, 2H), 7.56 (d, *J* = 2.4 Hz, 1H), 7.17 (d, *J* = 8.8 Hz, 2H), 7.13 – 7.04 (m, 3H), 7.03 – 6.92 (m, 7H), 6.89 – 6.82 (m, 2H), 6.65 (d, *J* = 8.8 Hz, 2H), 3.74 (s, 3H), 2.31 (s, 3H), 2.30 (s, 3H), 1.58 (s, 3H), 1.47 (s, 3H); ^{13}C NMR (100 MHz, $CDCl_3$) δ 159.4, 155.4, 154.1, 150.4, 144.2, 140.7, 140.1, 138.4, 137.9, 132.5, 132.2, 131.3, 131.0, 129.5, 129.0, 128.8, 128.5, 128.4, 127.7, 127.5, 126.8, 126.4, 124.4, 120.7, 120.0, 113.0, 89.7, 55.3, 21.4, 21.3, 20.9, 20.0; IR (neat): 2925, 2852, 1607, 1533, 1348, 1183, 1068, 830, 740, 570 cm^{-1}; HRESIMS Calcd for $[C_{42}H_{36}N_4NaO_6S]^+$ (M + Na^+) 747.2248, found 747.2255.

6-(4-(2,6-dimethylphenyl)-1-((4-nitrophenyl)sulfonyl)-1*H*-pyrrol-3-yl)-6-(4-methoxyphenyl)-3,4-dimethyl-6*H*-1,2,5-oxadiazine (3w)

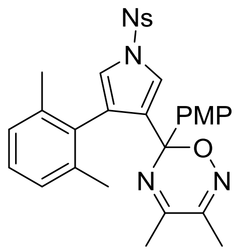

3w

Compound **3w** was prepared in 56% yield (32.0 mg) according to the general procedure. The product was isolated through silica gel column chromatography (PE/EA = 5/1) as a pale yellow solid (mp 160–164 °C). ^{1}H NMR (400 MHz, $CDCl_3$) δ 8.33 (d, *J* = 8.4 Hz, 2H), 7.99 (d, *J* = 8.4 Hz, 2H), 7.25 (s, 1H), 7.10 – 6.91 (m, 4H), 6.88 – 6.77 (m, 2H), 6.63 (d, *J* = 8.0 Hz, 2H), 3.75 (s, 3H), 1.93 (s, 3H), 1.83 (s, 3H), 1.82 (s, 3H), 1.51 (s, 3H); ^{13}C NMR (100 MHz, $CDCl_3$) δ 159.2, 153.2, 150.9, 150.6, 144.4, 138.4, 138.0, 132.5, 132.1, 131.5, 128.4(2), 128.3(5), 127.9, 127.5, 126.5, 126.4, 124.5, 120.9, 119.7, 112.8, 88.4, 55.2, 21.4, 20.7, 20.3, 16.6; IR (neat): 2923, 2840, 1609, 1533, 1382, 1183, 1066, 829, 740, 646 cm^{-1}; HRESIMS Calcd for $[C_{30}H_{29}N_4O_6S]^+$ (M + H^+) 573.1802, found 573.1793.

General procedure for the synthesis of chiral oxadiazine 3x.

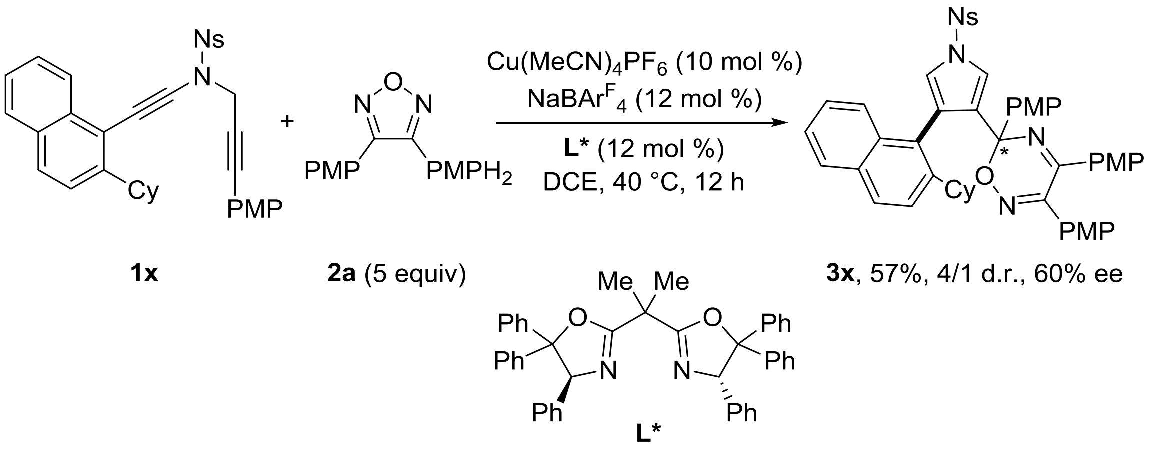

The powdered $Cu(MeCN)_4PF_6$ (0.01 mmol, 3.7 mg), **L*** (0.012 mmol, 7.7 mg), $NaBAr^F{}_4$ (0.012 mmol, 10.7 mg) were introduced into an oven-dried Schlenk tube. After addition of DCE (2 mL) into the Schlenk tube, the solution was stirred at 40 °C for 2 h. Subsequently, a solution of *N*-propargyl ynamide **1x** (0.1 mmol, 47.5 mg) and oxadiazole **2a** (0.5 mmol, 141.1 mg) in DCE (2 mL) was introduced into the system. The resulting mixture was stirred at 40 °C and the progress of the reaction was monitored by TLC.

Upon completion, the mixture was concentrated and the residue was purified by column chromatography on silica gel (DCM/PE = 3/1) to afford the desired chiral oxadiazine **3x** in 57% yield (48.8 mg) as a pale yellow solid (mp 168–170 °C). $[\alpha]_D^{20}$ = -23.149 (c = 1.0, $CHCl_3$). 60% ee (determined by HPLC: Chiralpak IC Column, *n*-hexane/2-propanol = 70/30, flow rate = 1.0 mL/min, 254 nm; TR = 14.01 min (major), 16.53 min (minor)). ^{1}H NMR (400 MHz, $CDCl_3$) δ 7.93 (d, *J* = 8.8 Hz, 2H), 7.86 (d, *J* = 8.8 Hz, 2H), 7.84 – 7.69 (m, 3H), 7.40 (d, *J* = 8.4 Hz, 1H), 7.24 – 7.09 (m, 5H), 6.94 (s, 1H), 6.86 – 6.79 (m, 1H), 6.76 (d, *J* = 8.4 Hz, 2H), 6.59 – 6.35 (m, 7H), 3.84 (s, 3H), 3.71 (s, 3H), 3.64 (s, 3H), 2.09 – 2.00 (m, 1H), 1.64 – 1.36 (m, 6H), 1.15 – 1.01 (m, 2H), 0.90 – 0.68 (m, 2H). ^{13}C NMR (100 MHz, $CDCl_3$) δ 160.9, 159.2, 154.5, 153.5, 150.4, 144.9, 143.9, 133.8, 131.6, 131.5, 130.6, 130.2, 129.3, 128.6, 128.4, 127.8, 127.7, 127.3, 126.9, 126.0, 125.5, 124.7, 124.5, 124.3, 124.2, 122.0, 121.8, 113.6, 113.1, 112.8, 89.7, 55.3, 55.2, 55.0, 41.7, 36.3, 31.1, 27.0, 26.5, 26.1; IR (neat): 2925, 2852, 1607, 1510, 1253, 1182, 1066, 834, 740, 587 cm^{-1}; HRESIMS Calcd for $[C_{50}H_{44}N_4NaO_8S]^+$ (M + Na$^+$) 883.2772, found 883.2764.

1.4 Preparative-Scale Synthesis and Synthetic Transformations

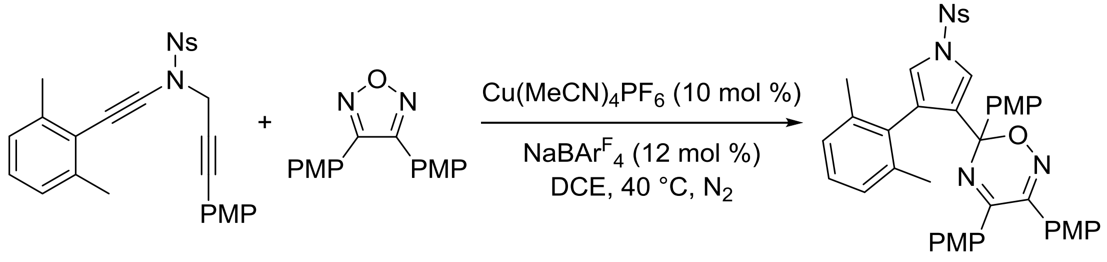

1a, 1 mmol, 0.48 g　　**2a** (5 mmol)　　**3a**, 72%, 0.54 g

1,2,5-oxadiazole **2a** (5 mmol, 1.41 g), NaBAr$^F_4$ (0.12 mmol, 106.4 mg) and $Cu(MeCN)_4PF_6$ (0.1 mmol, 37.3 mg) were added in this order to the *N*-propargyl ynamide **1a** (1 mmol, 0.48 g) in DCE (20 mL) at room temperature. The reaction mixture was stirred at 40 ℃ and the progress of the reaction was monitored by TLC. Upon completion, the mixture was concentrated under reduced pressure and the residue was purified by column chromatography on silica gel (dichloromethane/hexane) to afford the desired oxadiazine **3a** (72%, 0.54 g).

6-(4-(2,6-dimethylphenyl)-1*H*-pyrrol-3-yl)-3,4,6-tris(4-methoxyphenyl)-6*H*-1,2,5-

oxadiazine (4a)

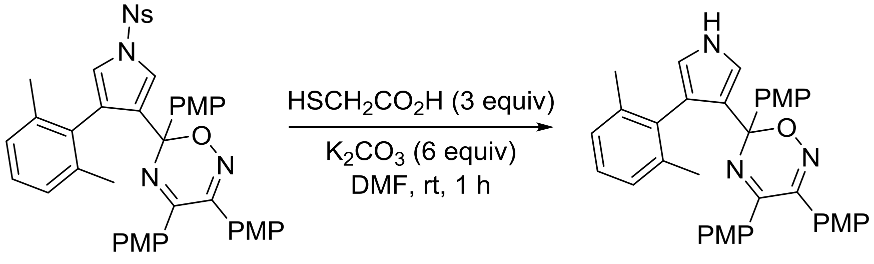

3a **4a**, 77%

Compound **4a** was prepared in 77% yield (44.0 mg) according to the known procedure (0.1 mmol scale).[6] The product was isolated through silica gel column chromatography (PE/EA = 3/1) as a pale yellow solid (mp 123–125 ºC). ^{1}H NMR (400 MHz, $CDCl_3$) δ 8.19 (s, 1H), 7.27 (d, J = 8.8 Hz, 2H), 7.18 (d, J = 8.8 Hz, 2H), 7.09 (d, J = 8.8 Hz, 2H), 7.06 – 7.01 (m, 2H), 6.93 (d, J = 7.2 Hz, 1H), 6.85 (d, J = 7.2 Hz, 1H), 6.73 – 6.66 (m, 4H), 6.63 (d, J = 8.8 Hz, 2H), 6.46 – 6.40 (m, 1H), 3.78 (s, 3H), 3.72 (s, 6H), 1.79 (s, 3H), 1.73 (s, 3H); ^{13}C NMR (100 MHz, $CDCl_3$) δ 160.9, 160.4, 158.8, 154.7, 152.8, 139.2, 139.0, 135.6, 133.5, 130.6, 129.2, 128.7, 128.5, 126.5, 126.4, 126.0, 125.7, 123.9, 121.5, 117.9, 116.9, 113.6, 113.0, 112.6, 90.7, 55.2(1), 55.2(0), 55.1(8), 21.2, 20.7; IR (neat): 3446, 2919, 1608, 1508, 1249, 1173, 1025, 978, 831, 549 cm^{-1}; HRESIMS Calcd for $[C_{36}H_{34}N_3O_4S]^+$ (M + H$^+$) 572.2543, found 572.2549.

2-(4-(2,6-dimethylphenyl)-1*H*-pyrrol-3-yl)-2,4,5-tris(4-methoxyphenyl)-2*H*-imidazole 1-oxide (5a)

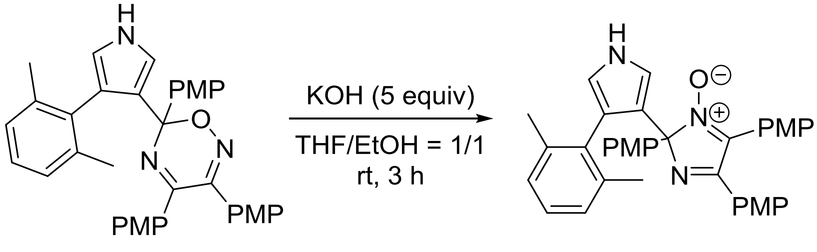

4a **5a**, 95%

To the solution of **4a** (0.1 mmol, 57.2 mg) in THF (1 mL) and EtOH (1 mL) was added KOH (0.5 mmol, 28.1 mg). The reaction was stirred at room temperature and the progress of the reaction was monitored by TLC. Upon completion, the reaction was diluted with

water, extracted with EtOAc, dried over $MgSO_4$ and concentrated. The residue was purified by column chromatography on silica gel (PE/EA = 2/1) to afford the desired chiral oxadiazine **5a** in 95% yield (54.2 mg) as a pale yellow solid (mp 263–265 ºC). ^{1}H NMR (400 MHz, $CDCl_3$) δ 8.67 (s, 1H), 7.85 (d, *J* = 8.8 Hz, 2H), 7.32 (d, *J* = 9.2 Hz, 2H), 7.08 (d, *J* = 8.8 Hz, 2H), 6.90 – 6.81 (m, 6H), 6.80 – 6.75 (m, 1H), 6.75 – 6.69 (m, 2H), 6.57 – 6.52 (m, 1H), 6.43 – 6.36 (m, 1H), 3.81 (s, 3H), 3.80 (s, 3H), 3.79 (s, 3H), 2.09 (s, 3H), 1.96 (s, 3H); ^{13}C NMR (100 MHz, $CDCl_3$) δ 165.2, 161.2, 160.3, 159.5, 139.0, 138.7, 135.2, 135.1, 131.0, 130.9, 130.6, 129.1, 127.0, 126.5, 125.0, 122.5, 121.2, 119.0, 118.2, 116.8, 113.2, 113.1, 112.9, 103.7, 55.3, 55.2, 21.3, 21.0; IR (neat): 3434, 2917, 1609, 1509, 1253, 1171, 1031, 835, 808, 586 cm^{-1}; HRESIMS Calcd for $[C_{36}H_{34}N_3O_4S]^+$ ($M + H^+$) 572.2543, found 572.2549.

1.5 Crystal Data and Structure Refinement for 3a. CCDC Number = 2268060

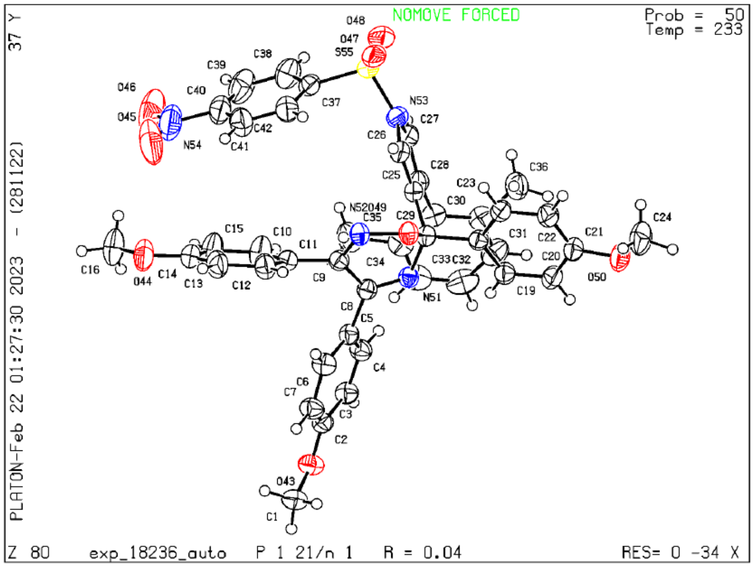

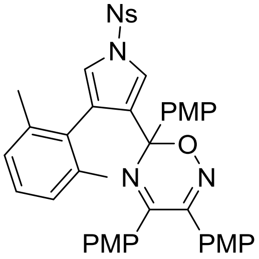

3a

Bond precision: C-C = 0.0025 A Wavelength=1.54184

Cell:	a=14.9717(1)	b=15.0636(1)	c=16.8102(1)
	alpha=90	beta=97.488(1)	gamma=90
Temperature:	233 K		

	Calculated	Reported
Volume	3758.84(4)	3758.83(4)
Space group	P 21/n	P 1 21/n 1
Hall group	-P 2yn	-P 2yn
Moiety formula	C42 H36 N4 O8 S	C42 H36 N4 O8 S
Sum formula	C42 H36 N4 O8 S	C42 H36 N4 O8 S
Mr	756.81	756.81
Dx,g cm-3	1.337	1.337
Z	4	4
Mu (mm-1)	1.266	1.266
F000	1584.0	1584.0
F000'	1590.21	
h,k,lmax	18,18,20	18,18,20
Nref	7671	7440
Tmin,Tmax	0.987,0.987	0.914,1.000
Tmin'	0.987	

Correction method= # Reported T Limits: Tmin=0.914 Tmax=1.000
AbsCorr = MULTI-SCAN

Data completeness= 0.970 Theta(max)= 74.381

R(reflections)= 0.0401(6139) wR2(reflections)= 0.1138(7440)

S = 1.030 Npar= 501

1.6 HPLC Chromatograms

3v: HPLC (IC, *n*-hexane/2-propanol = 70/30, flow rate = 1.0 mL/min, 254 nm)

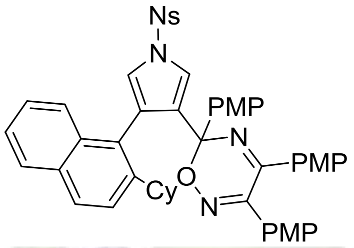

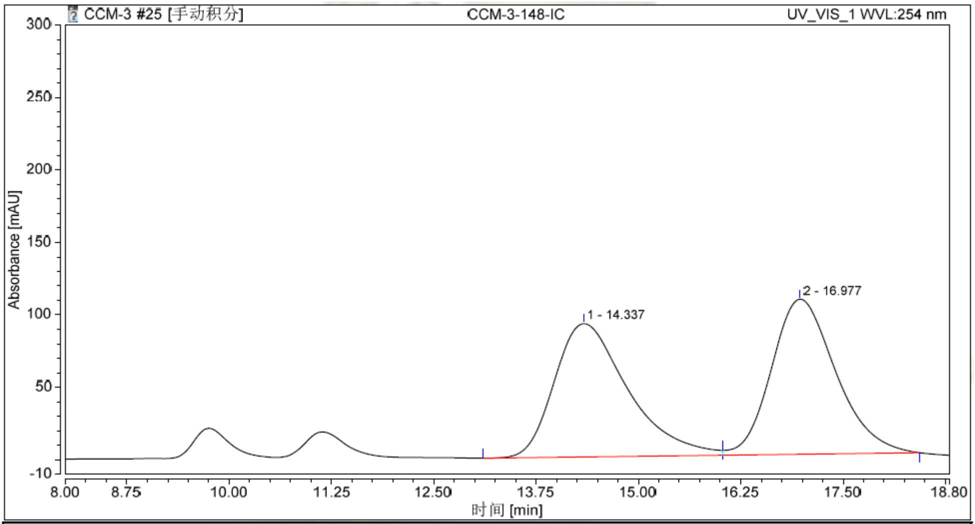

Integration Results

No.	Peak Name	Retention Time min	Area mAU*min	Height mAU	Relative Area %	Relative Height %	Amount n.a.
1		14.337	95.718	92.017	49.56	46.24	n.a.
2		16.977	97.436	106.962	50.44	53.76	n.a.
**Total:**			**193.155**	**198.979**	**100.00**	**100.00**	

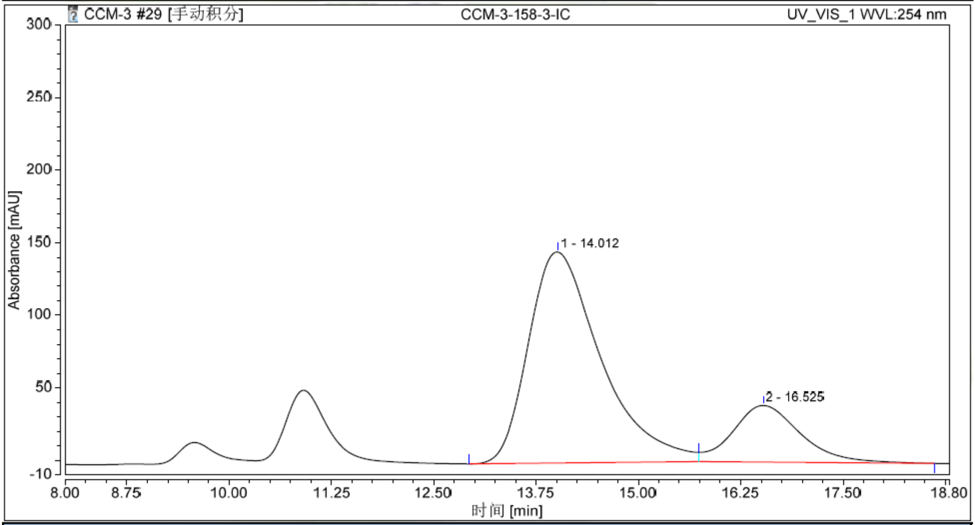

Integration Results

No.	Peak Name	Retention Time min	Area mAU*min	Height mAU	Relative Area %	Relative Height %	Amount n.a.
1		14.012	149.174	145.540	79.96	78.84	n.a.
2		16.525	37.396	39.053	20.04	21.16	n.a.
**Total:**			**186.570**	**184.593**	**100.00**	**100.00**	

2. Supplementary References:

1. Qi, L.-J. et al. Enantioselective copper-catalyzed formal [2+1] and [4+1] annulations of diynes with ketones via carbonyl ylides. *Angew. Chem. Int. Ed.* **61**, e202210637 (2022).

2. Zhu, X.-Q. et al. Copper-catalyzed asymmetric cyclization of alkenyl diynes: method development and new mechanistic insights. *Chem. Sci.* **12**, 9466–9474 (2021).

3. Huang, E.-H. et al. Copper-catalyzed Si−H bond insertion reaction of N-propargyl ynamides with hydrosilanes. *Org. Lett.* **24**, 196–201 (2022).

4. Zhang, Q., Zhao, C., Zhang, X., He, C. & Pang, S. Oxidation of *o*-dioxime by (diacetoxyiodo)benzene: green and mild access to furoxans. *New J. Chem.* **46**, 1489–1493 (2022).

5. Zhao, J.-Q. et al. Synthesis of furoxan derivatives: DABCO-mediated cascade sulfonylation/cyclization reaction of α-nitro-ketoximes. *Tetrahedron.* **71**, 1560–1565 (2015).

6. Liu, X. et al. Copper-catalyzed enantioselective Doyle–Kirmse reaction of azide-ynamides via α-imino copper carbenes. *Angew. Chem. Int. Ed.* **62**, e202216923 (2023).
