# Supplementary material for: Copper-catalyzed intermolecular formal (5 + 1) annulation of 1,5-diynes with 1,2,5-oxadiazoles
Source: Commun Chem. 2023 Sep 12;6:194. doi: 10.1038/s42004-023-00999-y (PMC10497616; doi:10.1038/s42004-023-00999-y)
Supplement: Supplementary file 2 — Description of additional supplementary file [file 42004_2023_999_MOESM2_ESM.pdf]

Description of Additional Supplementary Files

File name: Supplementary Data 1

Description: Supplementary Information for compound spectrum.

File name: Supplementary Data 2

Description: cif file of compound 3a
