# Supplementary material for: Copper-catalyzed intermolecular formal (5 + 1) annulation of 1,5-diynes with 1,2,5-oxadiazoles
Source: Commun Chem. 2023 Sep 12;6:194. doi: 10.1038/s42004-023-00999-y (PMC10497616; doi:10.1038/s42004-023-00999-y)

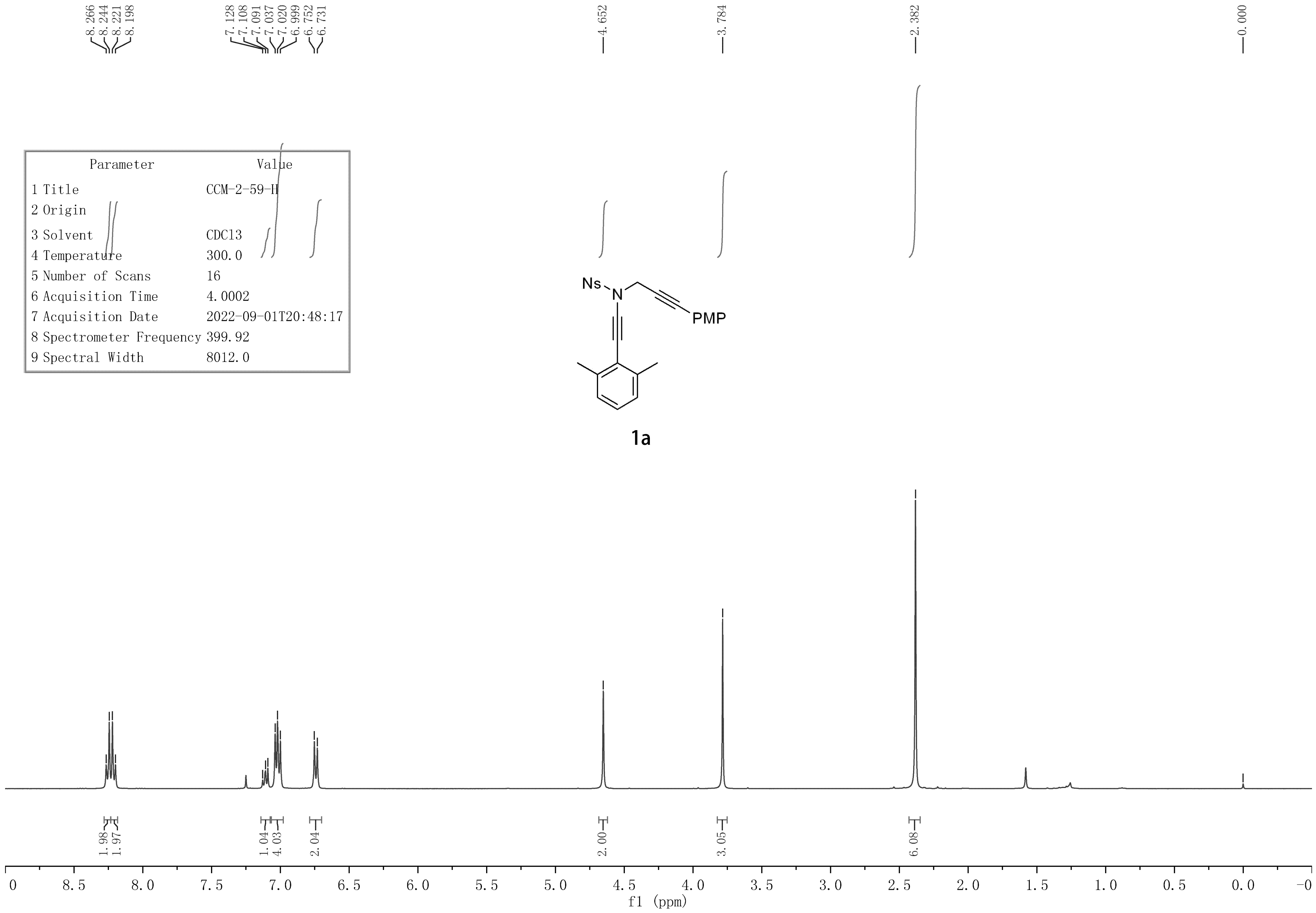
8.266
8.244
8.221
8.198
7.128
7.108
7.091
7.037
7.020
6.999
6.752
6.731
4.652
3.784
2.382
0.000
Parameter Value
1 Title CCM-2-59-H
2 Origin
3 Solvent CDCl3
4 Temperature 300.0
5 Number of Scans 16
6 Acquisition Time 4.0002
7 Acquisition Date 2022-09-01T20:48:17
8 Spectrometer Frequency 399.92
9 Spectral Width 8012.0
Ns
N
PMP
1a
1.98
1.97
1.04
4.03
2.04
2.00
3.05
6.08
f1 (ppm)

Parameter	Value
1 Title	CCM-2-59-C
2 Origin	
3 Solvent	CDCl3
4 Temperature	300.3
5 Number of Scans	200
6 Acquisition Time	1.0000
7 Acquisition Date	2022-09-01T20:57:39
8 Spectrometer Frequency	100.56
9 Spectral Width	26041.0

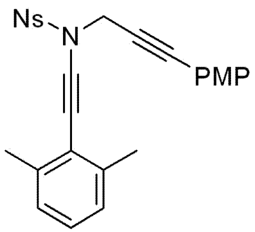

1a

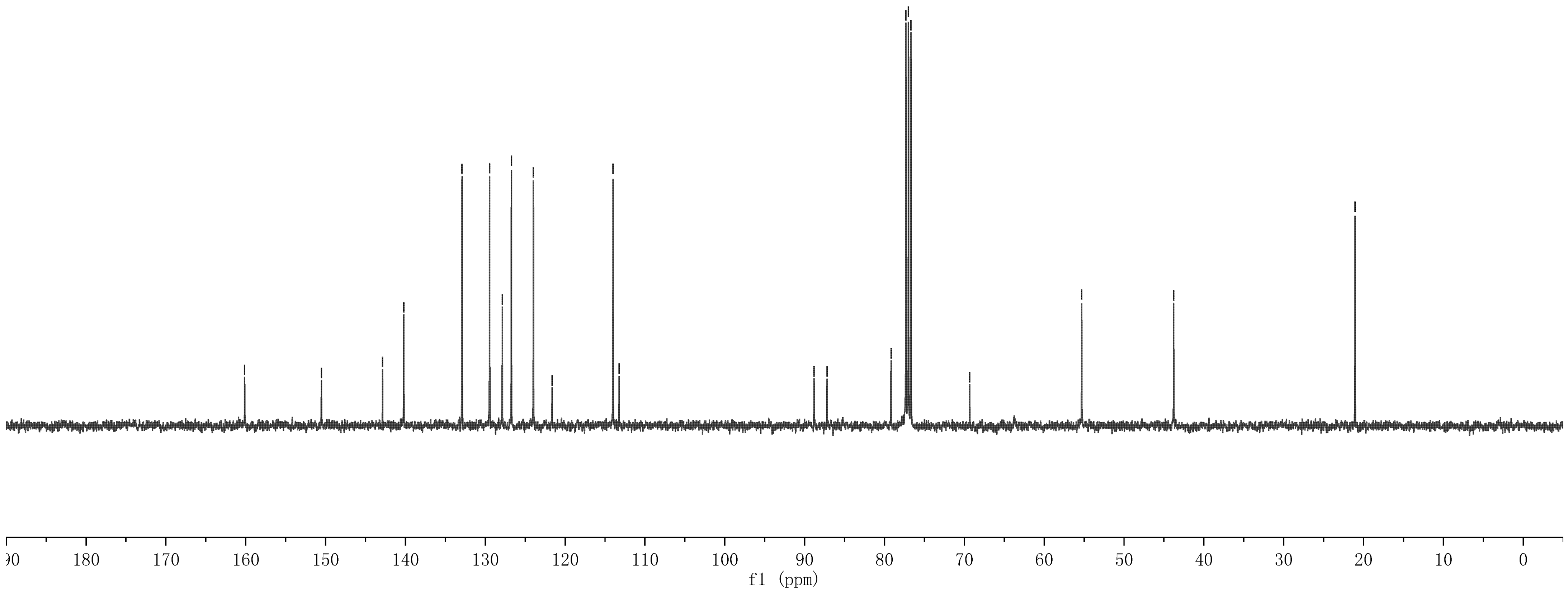

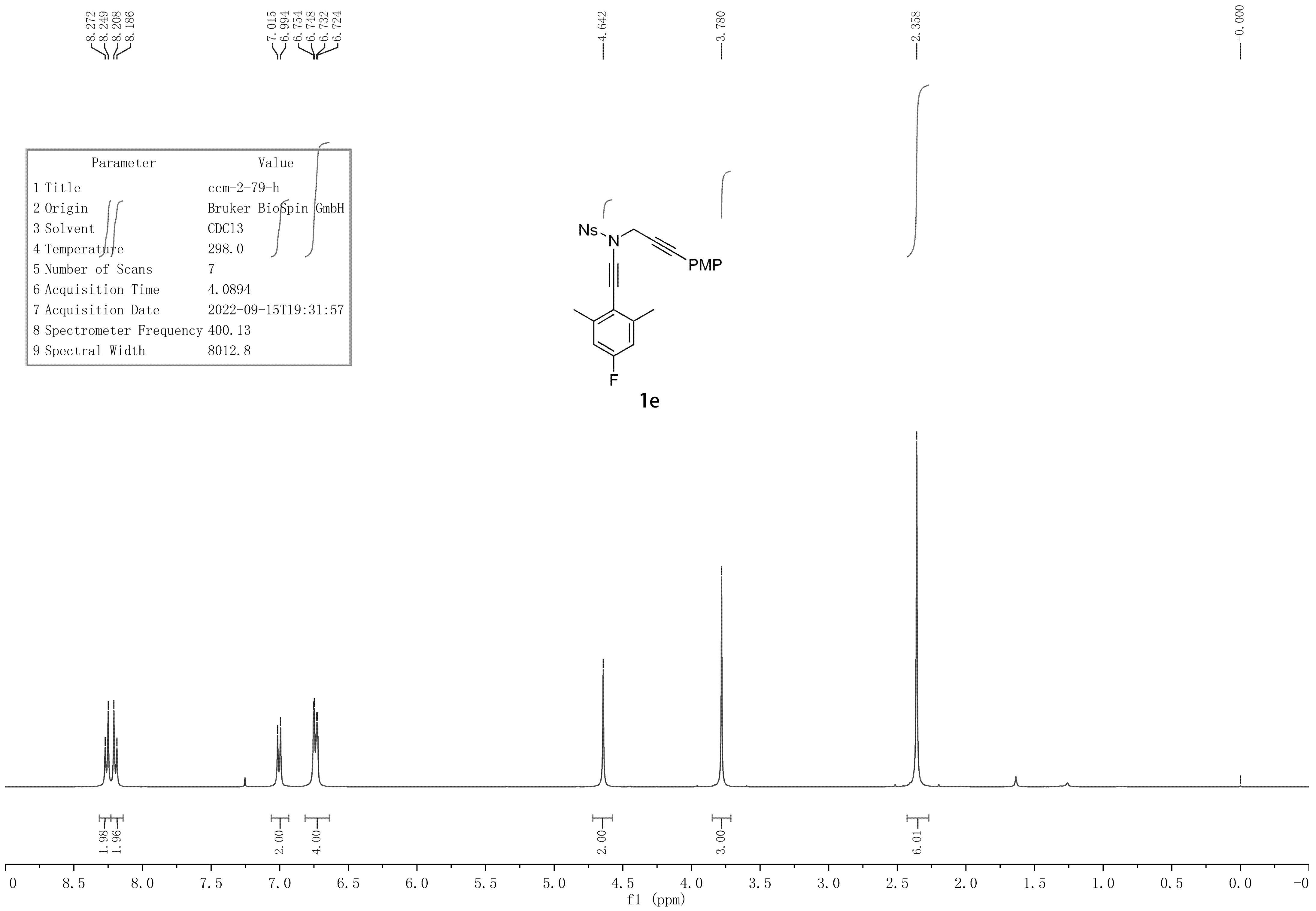
8.272
8.249
8.208
8.186
7.015
6.994
6.754
6.748
6.732
6.724
4.642
3.780
2.358
-0.000
Parameter Value
1 Title ccm-2-79-h
2 Origin Bruker BioSpin GmbH
3 Solvent CDCl3
4 Temperature 298.0
5 Number of Scans 7
6 Acquisition Time 4.0894
7 Acquisition Date 2022-09-15T19:31:57
8 Spectrometer Frequency 400.13
9 Spectral Width 8012.8
Ns
N
PMP
F
1e
1.98
1.96
2.00
4.00
2.00
3.00
6.01
0
8.5
8.0
7.5
7.0
6.5
6.0
5.5
5.0
4.5
4.0
3.5
3.0
2.5
2.0
1.5
1.0
0.5
0.0
-0
f1 (ppm)

Parameter	Value
1 Title	ccm-2-79-c
2 Origin	Bruker BioSpin GmbH
3 Solvent	CDCl3
4 Temperature	300.0
5 Number of Scans	41
6 Acquisition Time	1.3631
7 Acquisition Date	2022-09-15T19:33:26
8 Spectrometer Frequency	100.61
9 Spectral Width	24038.5

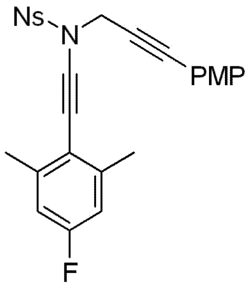

1e

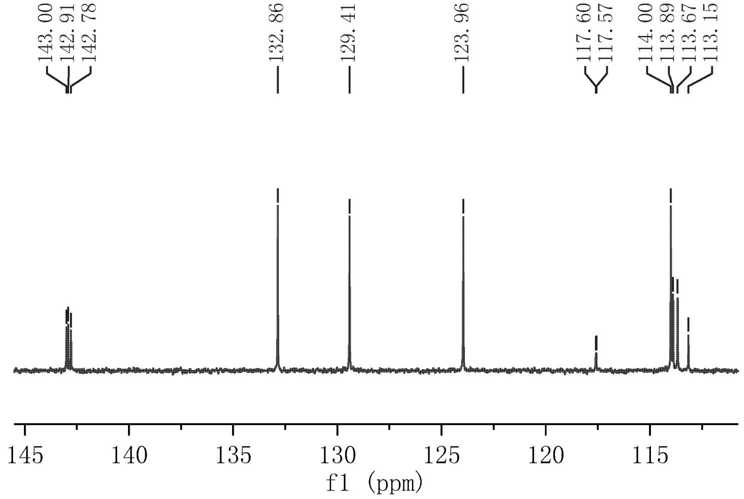

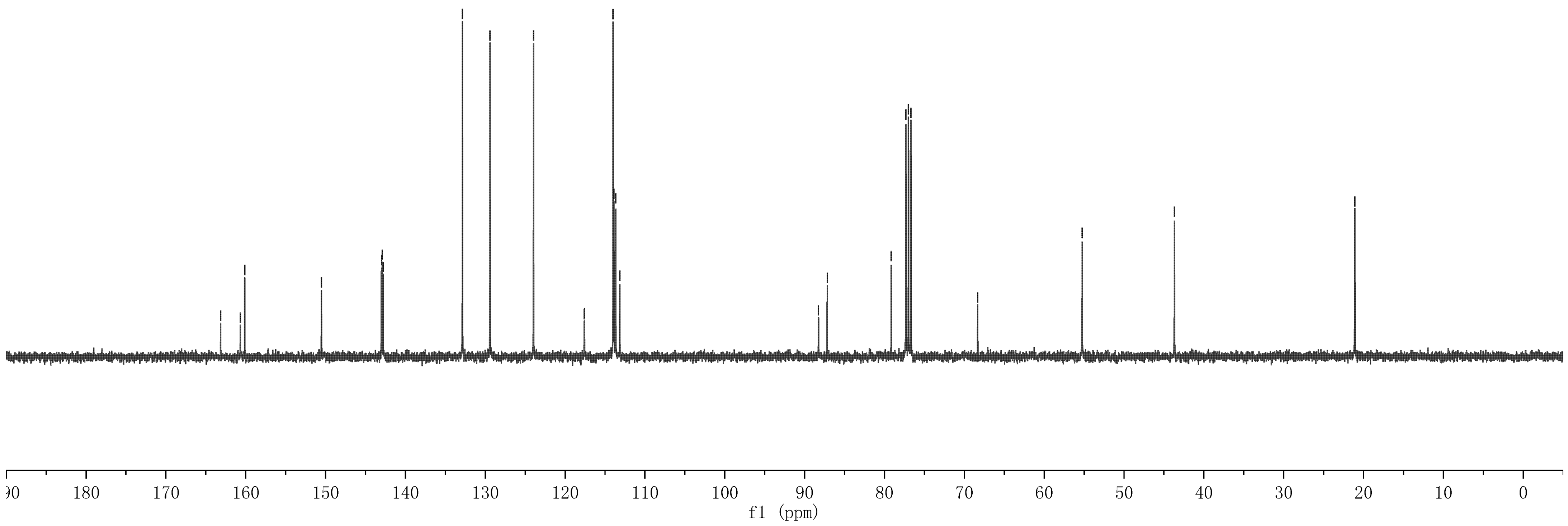

Parameter	Value
1 Title	CCM-2-79-F
2 Origin	Bruker BioSpin GmbH
3 Solvent	CDCl3
4 Temperature	297.1
5 Number of Scans	16
6 Acquisition Time	0.7340
7 Acquisition Date	2022-09-15T13:53:04
8 Spectrometer Frequency	376.31
9 Spectral Width	89285.7

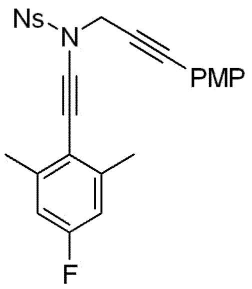

1e

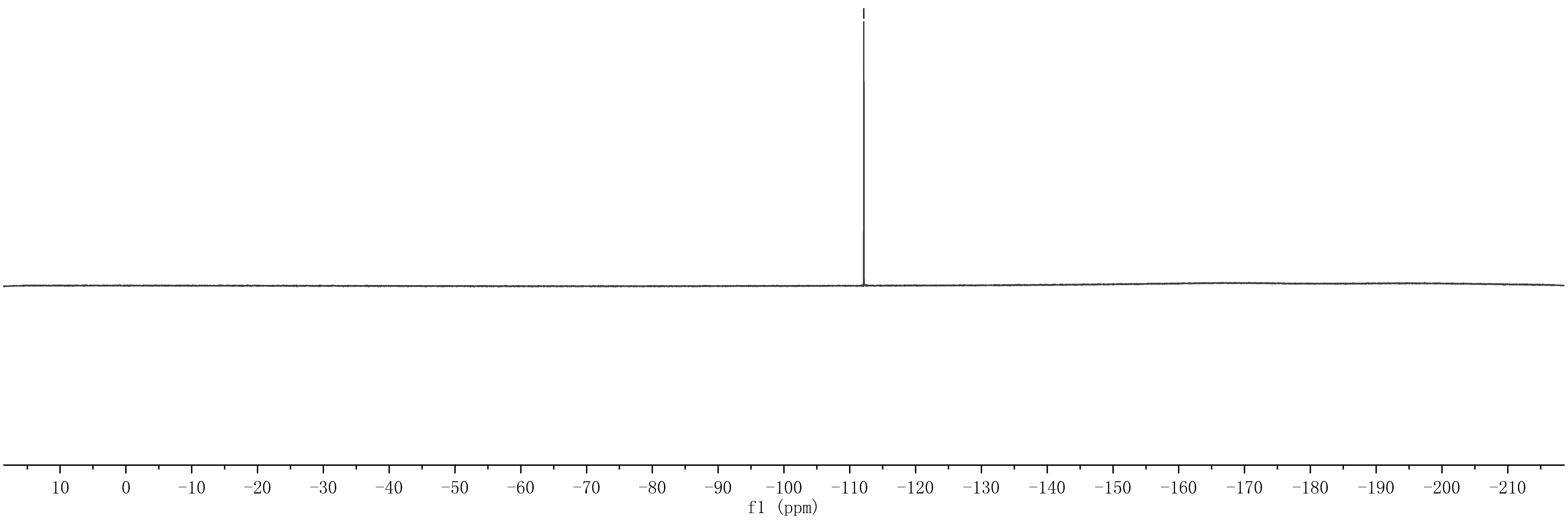

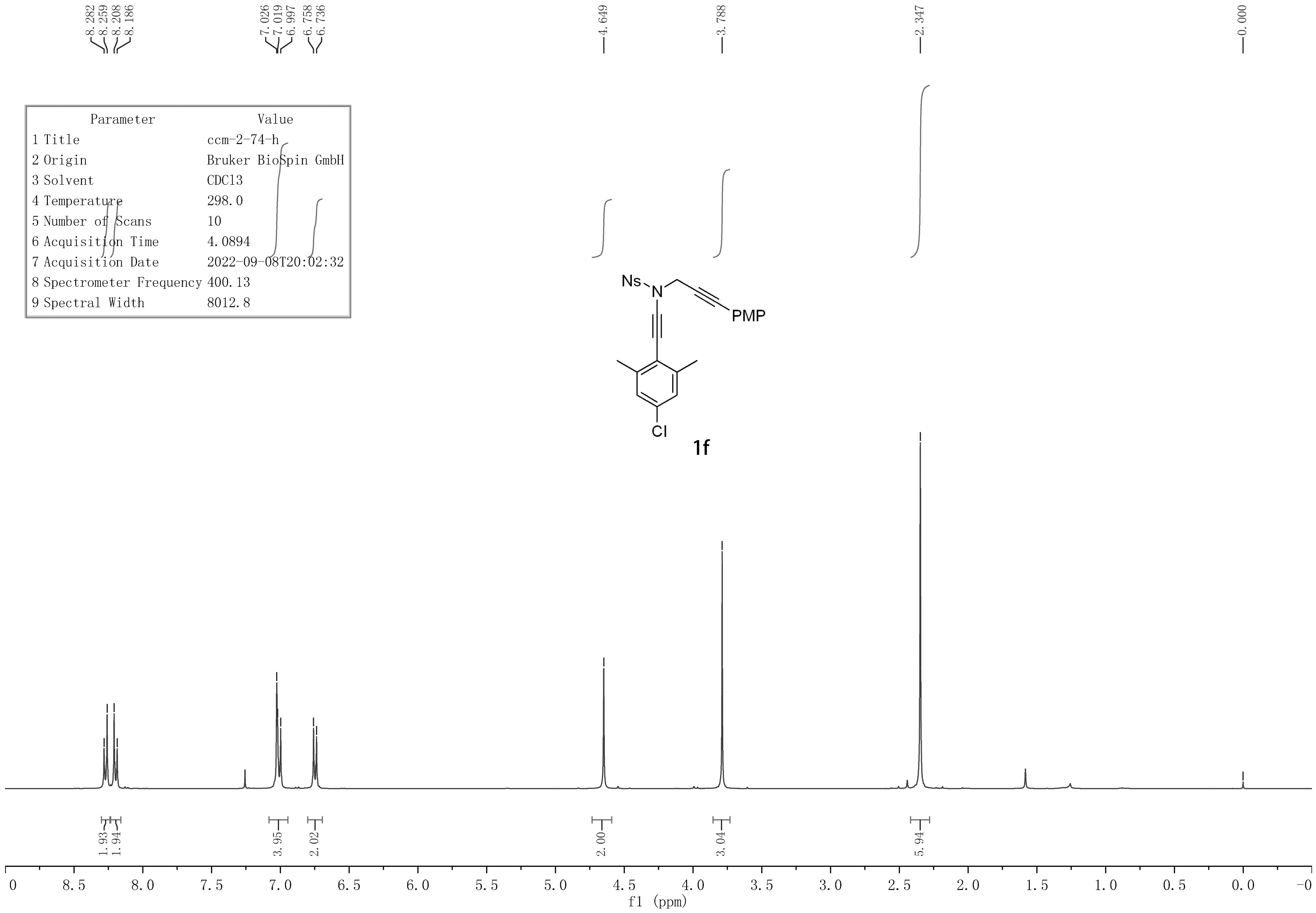

8.282
8.259
8.208
8.186
7.026
7.019
6.997
6.758
6.736
4.649
3.788
2.347
0.000
Parameter Value
1 Title ccm-2-74-h
2 Origin Bruker BioSpin GmbH
3 Solvent CDCl3
4 Temperature 298.0
5 Number of Scans 10
6 Acquisition Time 4.0894
7 Acquisition Date 2022-09-08T20:02:32
8 Spectrometer Frequency 400.13
9 Spectral Width 8012.8
Ns
N
PMP
Cl
1f
1.93
1.94
3.95
2.02
2.00
3.04
5.94
8.5 8.0 7.5 7.0 6.5 6.0 5.5 5.0 4.5 4.0 3.5 3.0 2.5 2.0 1.5 1.0 0.5 0.0
f1 (ppm)

Parameter	Value
1 Title	ccm-2-74-c
2 Origin	Bruker BioSpin GmbH
3 Solvent	CDCl3
4 Temperature	300.0
5 Number of Scans	53
6 Acquisition Time	1.3631
7 Acquisition Date	2022-09-08T20:05:38
8 Spectrometer Frequency	100.61
9 Spectral Width	24038.5

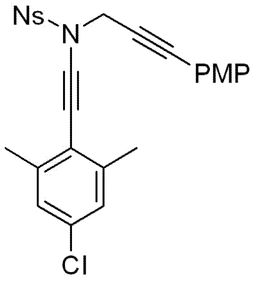

1f

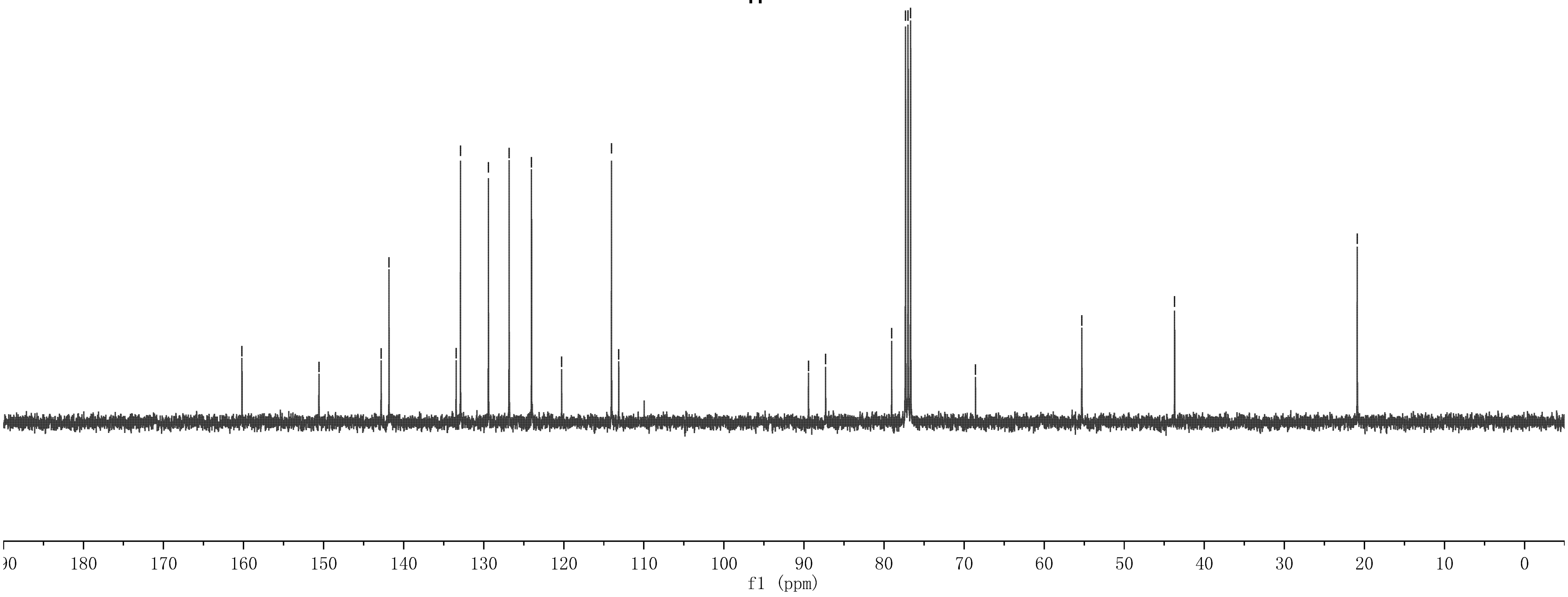

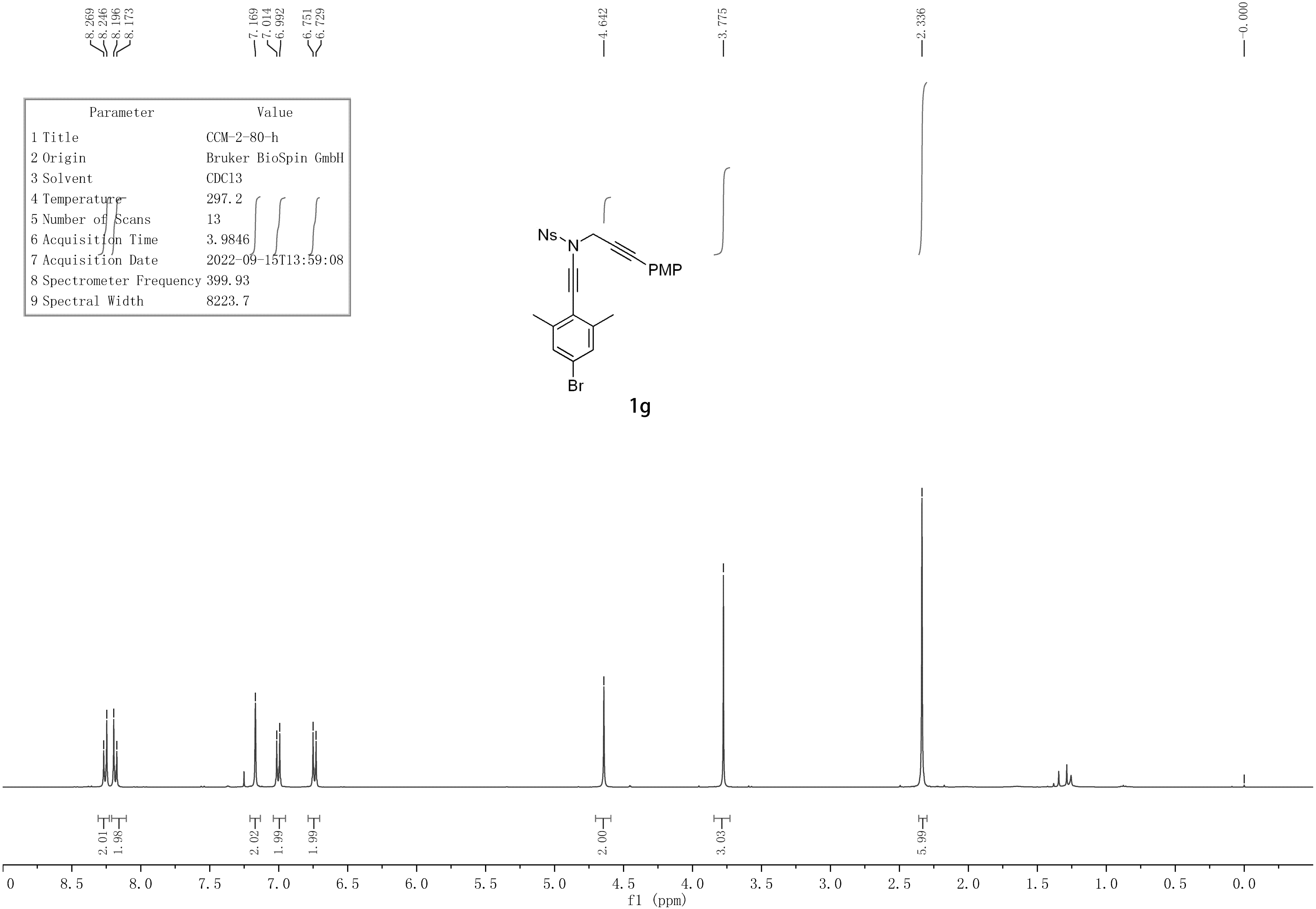
8.269
8.246
8.196
8.173
7.169
7.014
6.992
6.751
6.729
4.642
3.775
2.336
-0.000
Parameter Value
1 Title CCM-2-80-h
2 Origin Bruker BioSpin GmbH
3 Solvent CDCl3
4 Temperature 297.2
5 Number of Scans 13
6 Acquisition Time 3.9846
7 Acquisition Date 2022-09-15T13:59:08
8 Spectrometer Frequency 399.93
9 Spectral Width 8223.7
Ns
N
PMP
Br
1g
2.01
1.98
2.02
1.99
1.99
2.00
3.03
5.99
0 8.5 8.0 7.5 7.0 6.5 6.0 5.5 5.0 4.5 4.0 3.5 3.0 2.5 2.0 1.5 1.0 0.5 0.0
f1 (ppm)

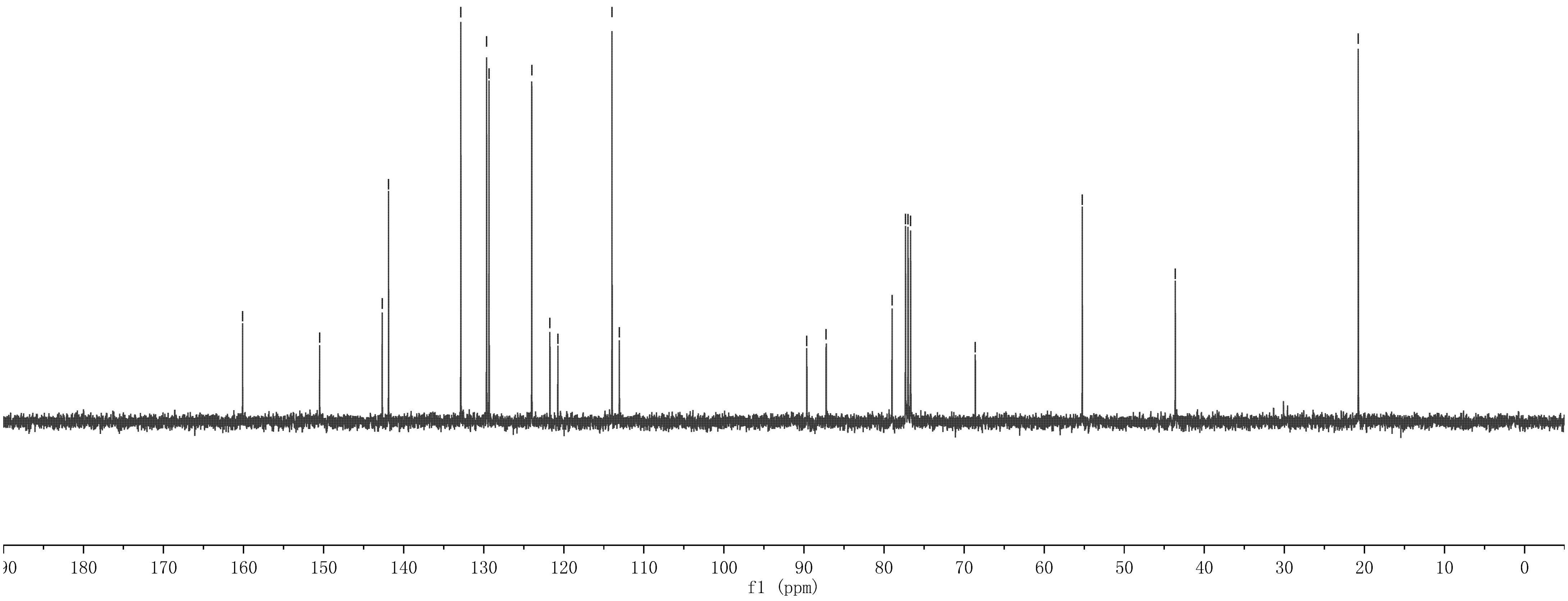

Parameter	Value
1 Title	CCM-2-80-c
2 Origin	Bruker BioSpin GmbH
3 Solvent	CDCl3
4 Temperature	297.6
5 Number of Scans	19
6 Acquisition Time	1.3631
7 Acquisition Date	2022-09-15T14:02:53
8 Spectrometer Frequency	100.56
9 Spectral Width	24038.5

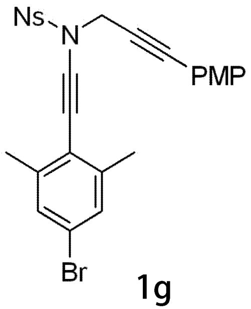

1g

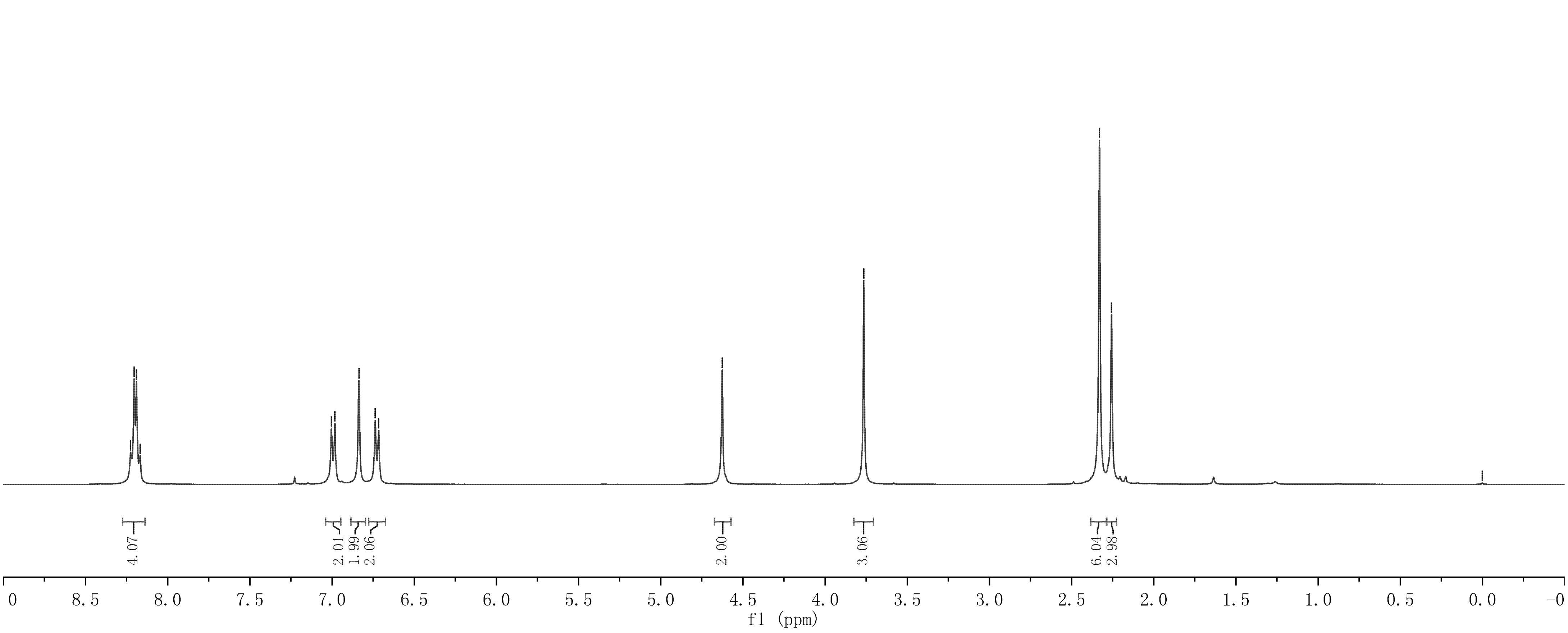

Parameter	Value
1 Title	CCM-2-172-H
2 Origin	Bruker BioSpin GmbH
3 Solvent	CDCl3
4 Temperature	298.0
5 Number of Scans	9
6 Acquisition Time	4.0894
7 Acquisition Date	2022-11-05T09:44:27
8 Spectrometer Frequency	400.13
9 Spectral Width	8012.8

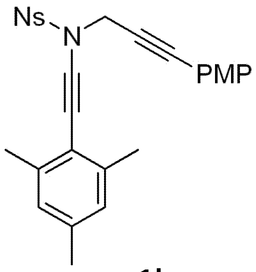

1h

Parameter	Value
1 Title	CCM-2-172-C
2 Origin	Bruker BioSpin GmbH
3 Solvent	CDCl3
4 Temperature	300.0
5 Number of Scans	23
6 Acquisition Time	1.3631
7 Acquisition Date	2022-11-05T09:47:01
8 Spectrometer Frequency	100.61
9 Spectral Width	24038.5

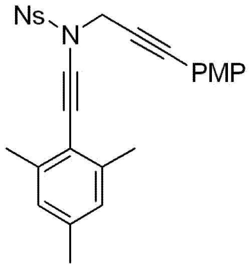

1h

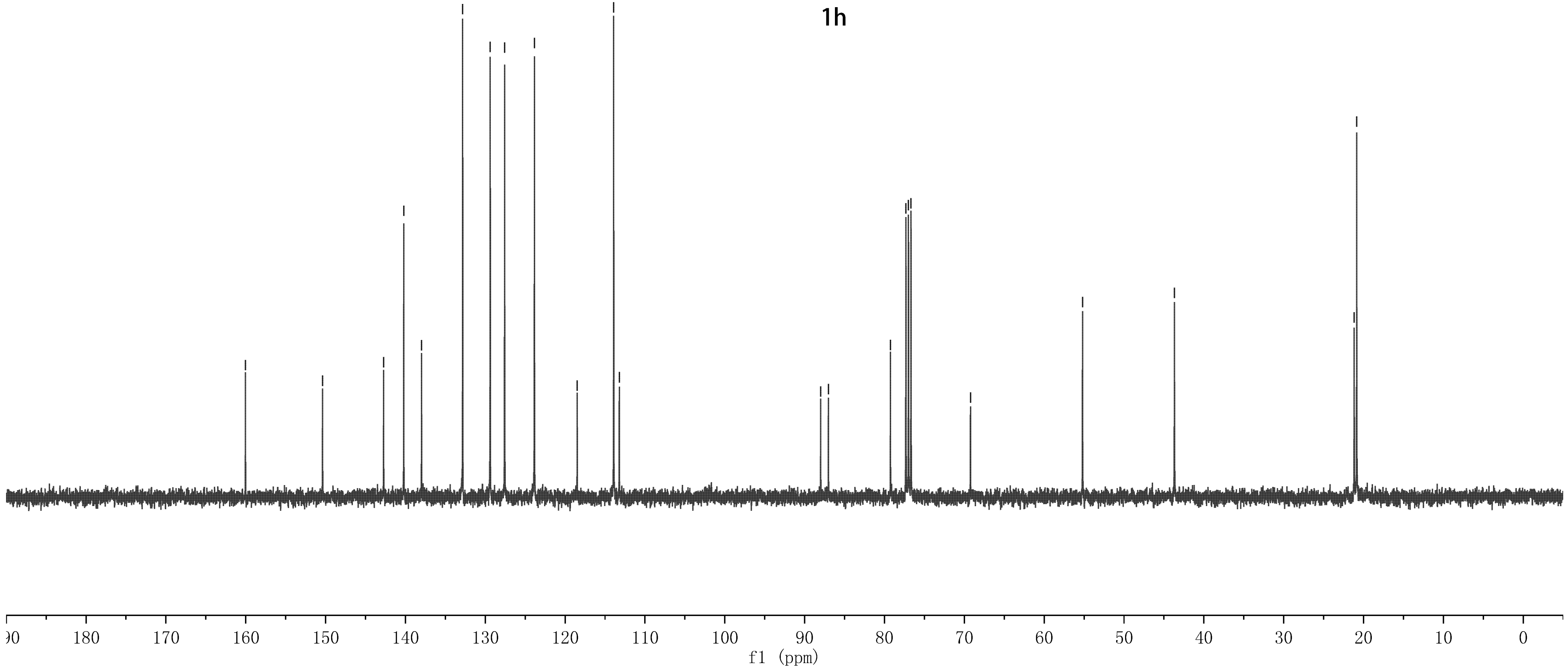

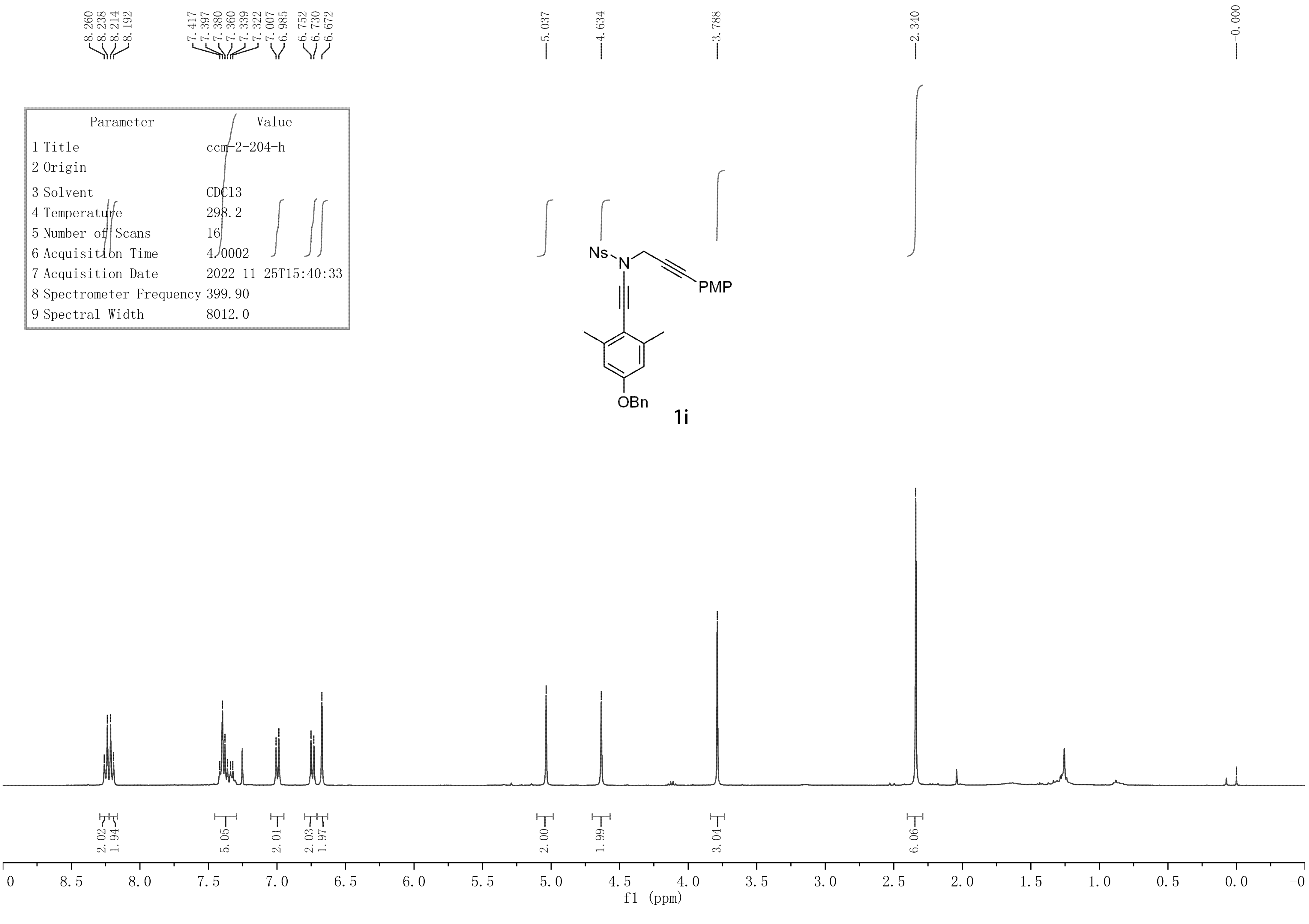

8.260
8.238
8.214
8.192
7.417
7.397
7.380
7.360
7.339
7.322
7.007
6.985
6.752
6.730
6.672
5.037
4.634
3.788
2.340
-0.000
Parameter Value
1 Title ccm-2-204-h
2 Origin
3 Solvent CDCl3
4 Temperature 298.2
5 Number of Scans 16
6 Acquisition Time 4.0002
7 Acquisition Date 2022-11-25T15:40:33
8 Spectrometer Frequency 399.90
9 Spectral Width 8012.0
Ns
N
PMP
OBn
1i
2.02
1.94
5.05
2.01
2.03
1.97
2.00
1.99
3.04
6.06
f1 (ppm)

Parameter	Value
1 Title	ccm-2-204-c
2 Origin	
3 Solvent	CDCl3
4 Temperature	298.5
5 Number of Scans	600
6 Acquisition Time	1.0000
7 Acquisition Date	2022-11-25T16:03:05
8 Spectrometer Frequency	100.56
9 Spectral Width	26041.0

1i

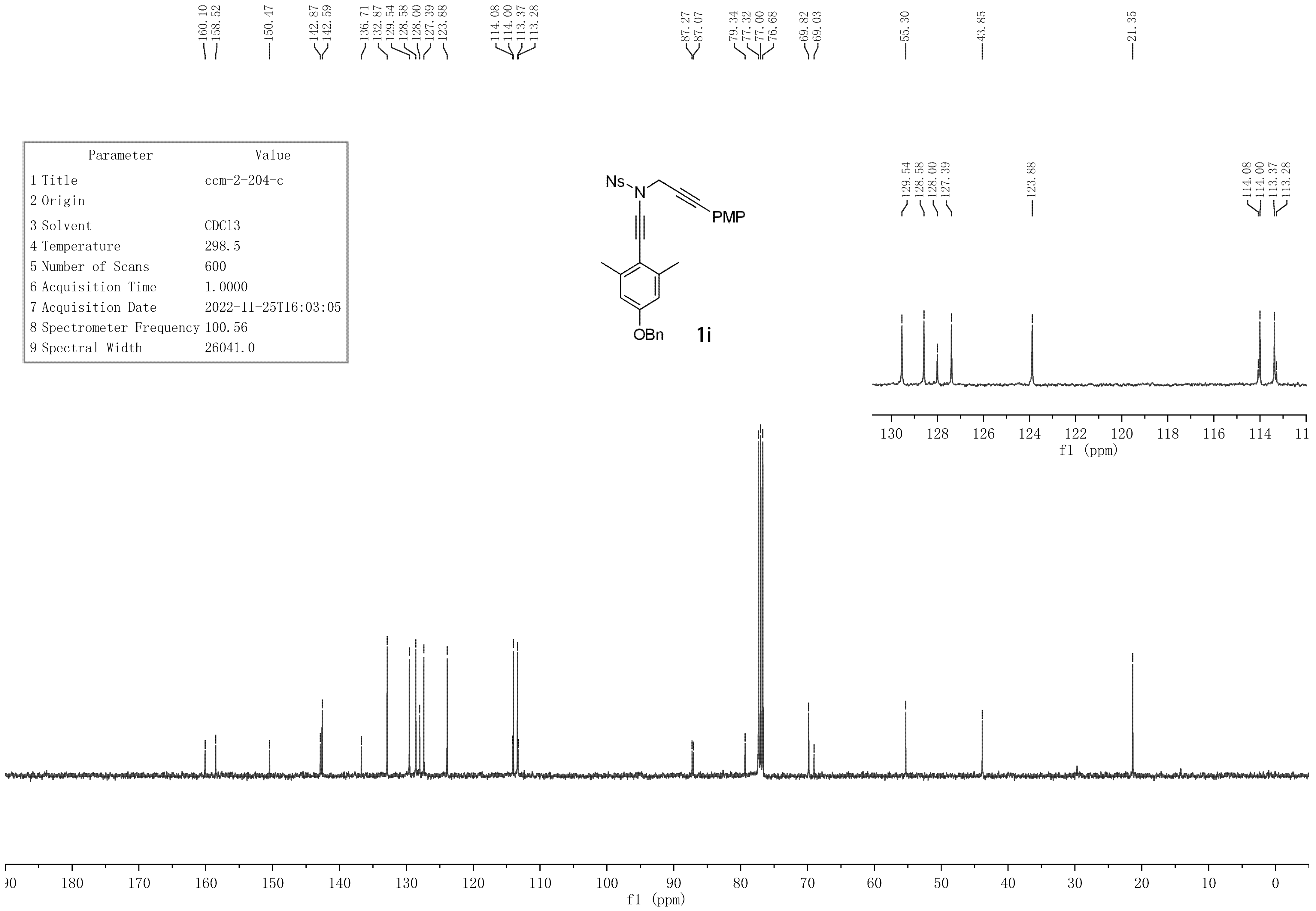

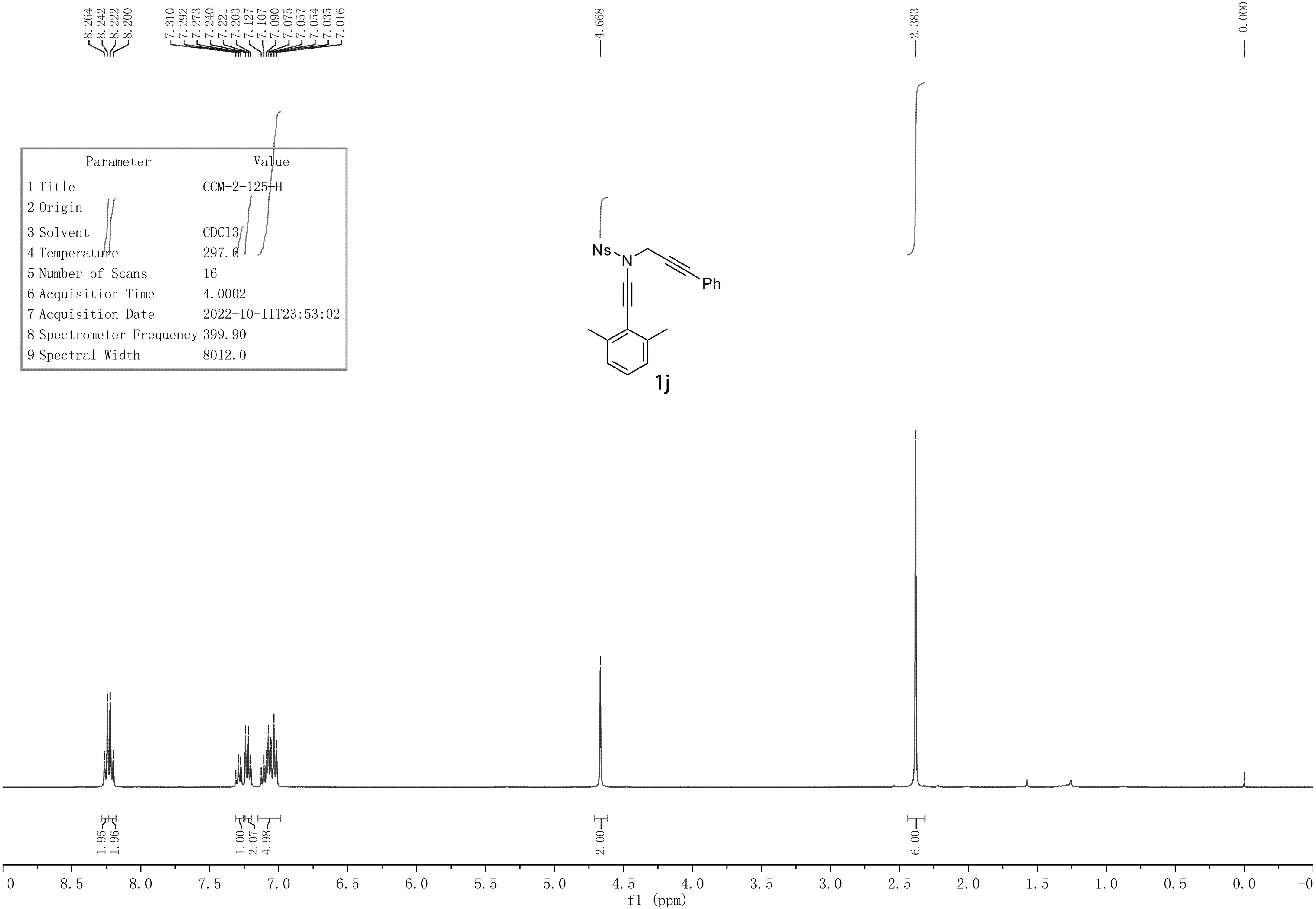

Parameter	Value
1 Title	CCM-2-125-H
2 Origin	
3 Solvent	CDCl3
4 Temperature	297.6
5 Number of Scans	16
6 Acquisition Time	4.0002
7 Acquisition Date	2022-10-11T23:53:02
8 Spectrometer Frequency	399.90
9 Spectral Width	8012.0

	Parameter	Value
1	Title	CCM-2-125-C
2	Origin	
3	Solvent	CDCl3
4	Temperature	296.8
5	Number of Scans	500
6	Acquisition Time	1.0000
7	Acquisition Date	2022-10-12T00:12:21
8	Spectrometer Frequency	100.56
9	Spectral Width	26041.0

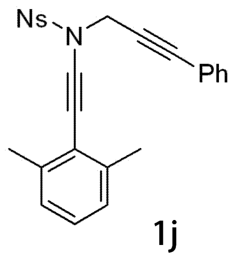

1j

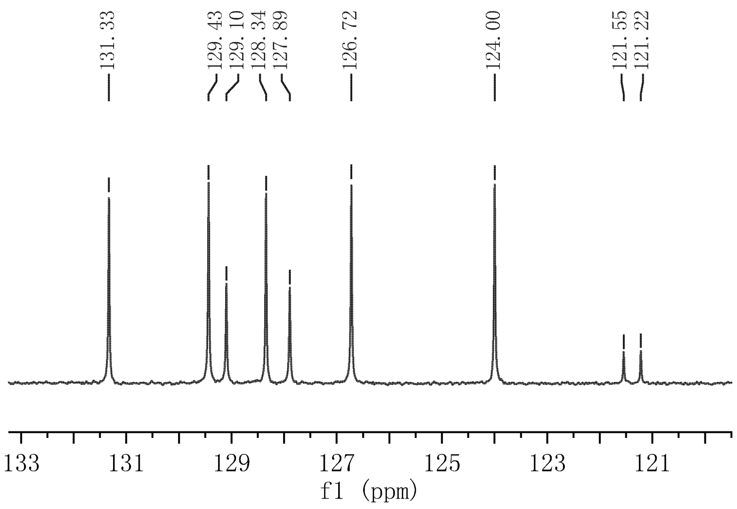

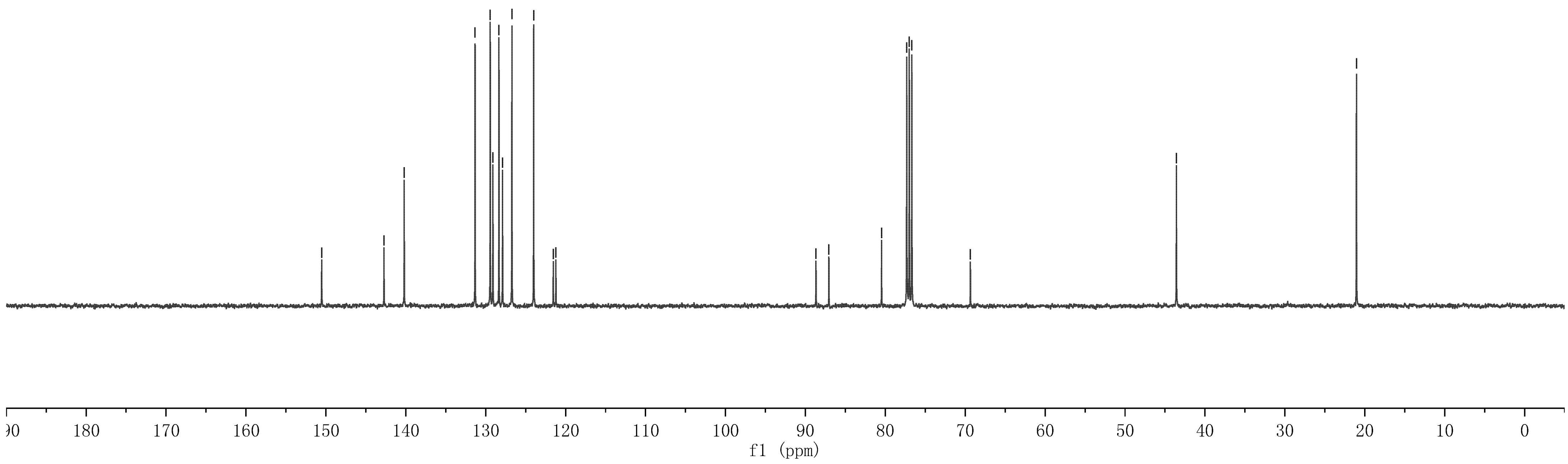

Parameter	Value
1 Title	CCM-2-128-H
2 Origin	
3 Solvent	CDCl3
4 Temperature	297.8
5 Number of Scans	16
6 Acquisition Time	4.0002
7 Acquisition Date	2022-10-12T00:17:12
8 Spectrometer Frequency	399.90
9 Spectral Width	8012.0

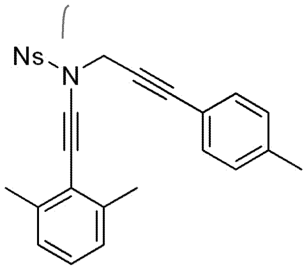

1k

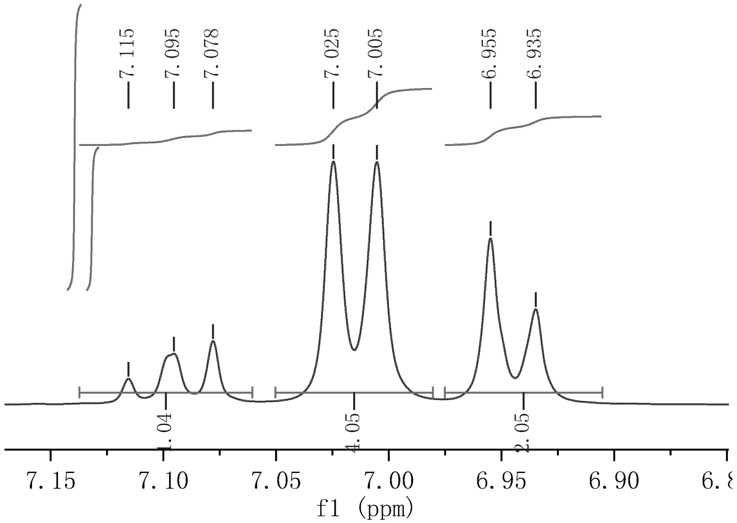

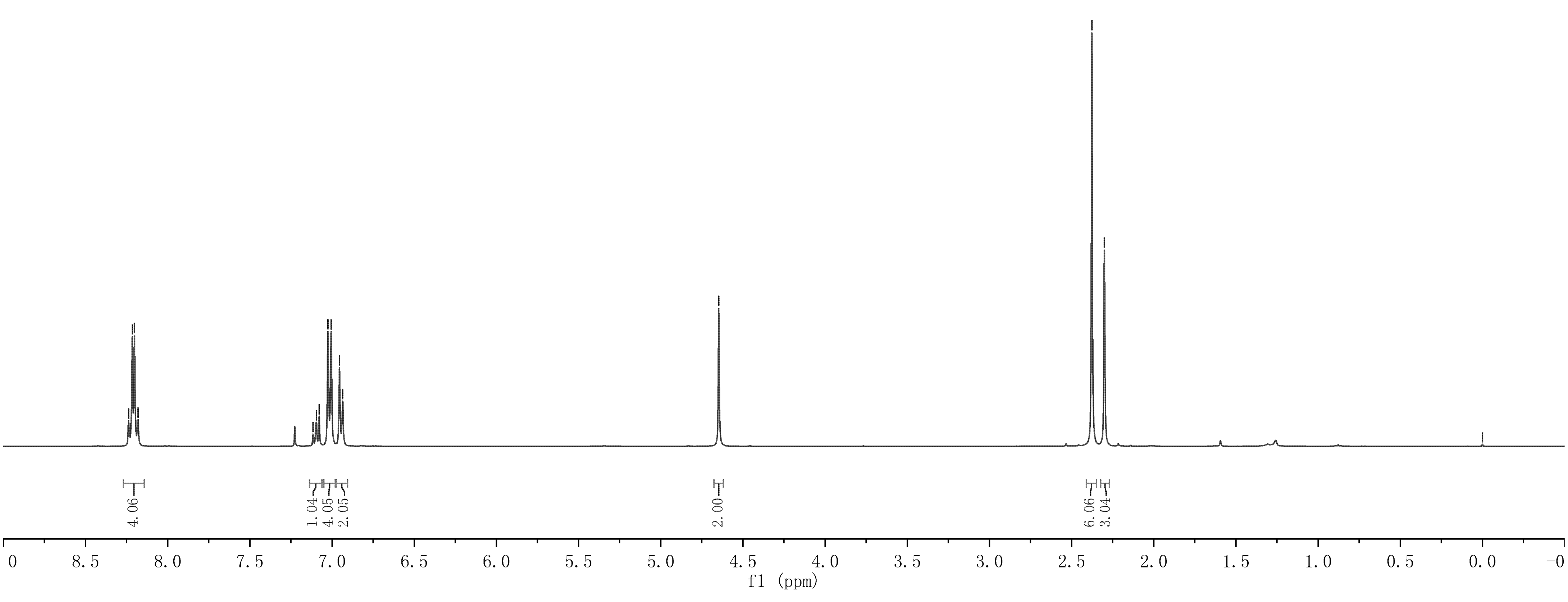

Parameter	Value
1 Title	CCM-2-128-C
2 Origin	
3 Solvent	CDCl3
4 Temperature	298.1
5 Number of Scans	500
6 Acquisition Time	1.0000
7 Acquisition Date	2022-10-12T00:36:37
8 Spectrometer Frequency	100.56
9 Spectral Width	26041.0

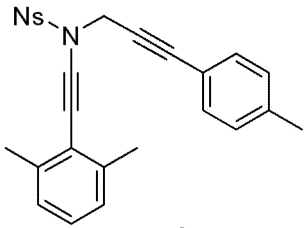

1k

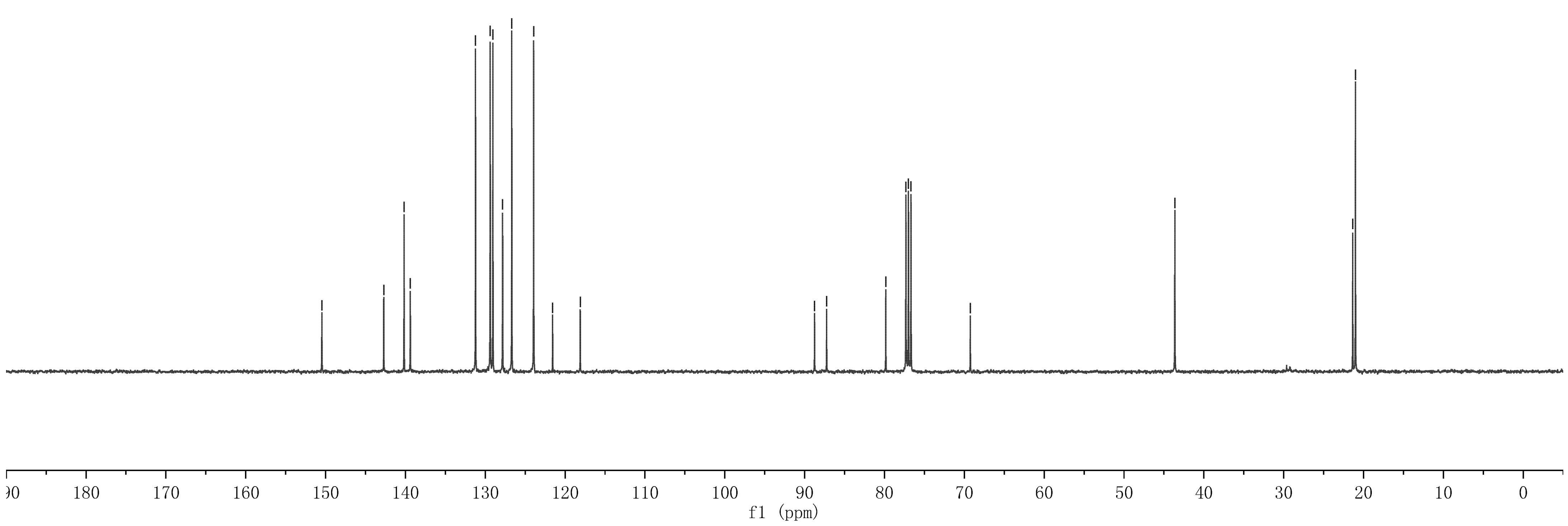

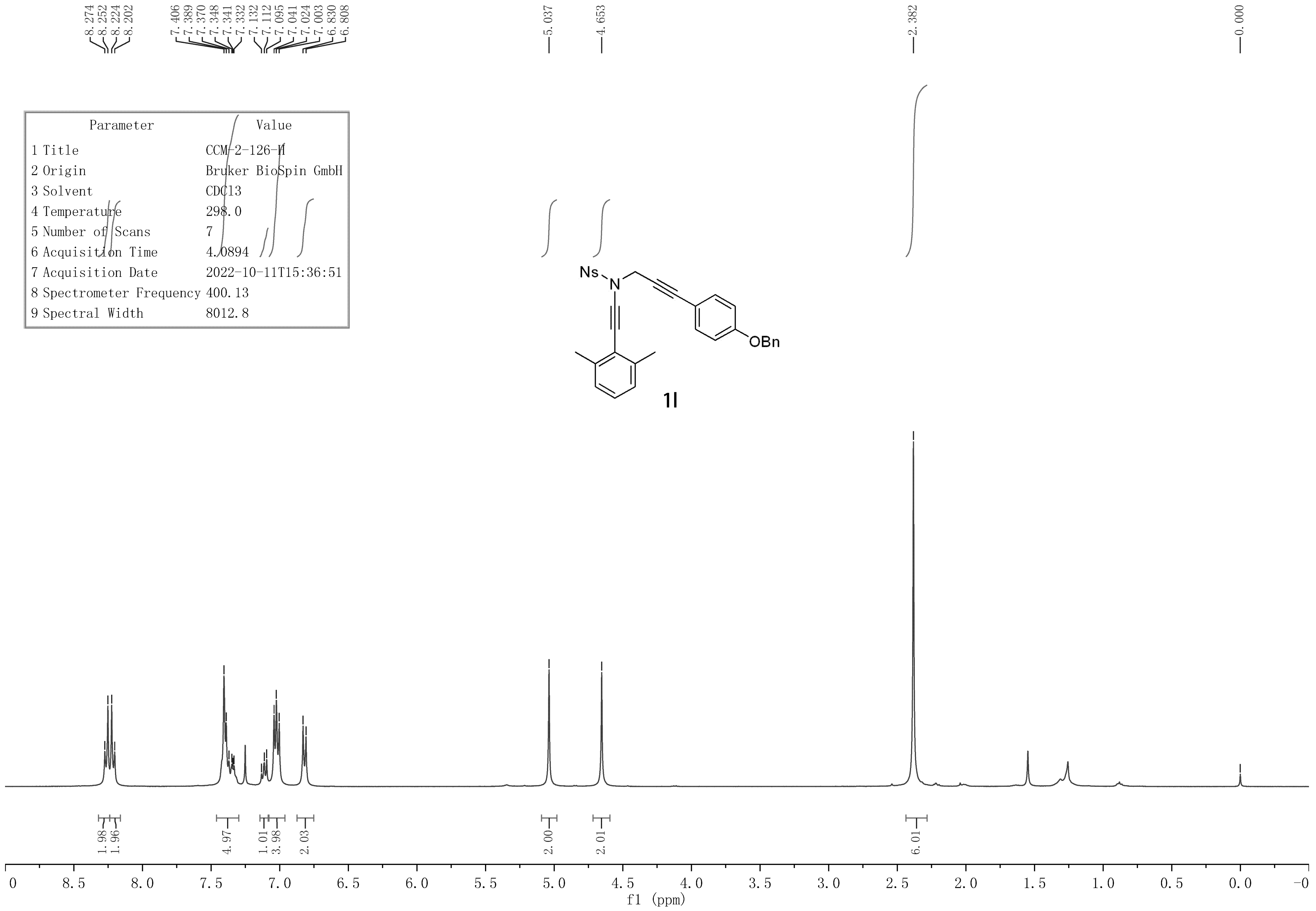
8.274
8.252
8.224
8.202
7.406
7.389
7.370
7.348
7.341
7.332
7.132
7.112
7.095
7.041
7.024
7.003
6.830
6.808
5.037
4.653
2.382
0.000
Parameter
Value
1 Title
CCM-2-126-H
2 Origin
Bruker BioSpin GmbH
3 Solvent
CDCl3
4 Temperature
298.0
5 Number of Scans
7
6 Acquisition Time
4.0894
7 Acquisition Date
2022-10-11T15:36:51
8 Spectrometer Frequency
400.13
9 Spectral Width
8012.8
Ns
N
OBn
1l
1.98
1.96
4.97
1.01
3.98
2.03
2.00
2.01
6.01
f1 (ppm)

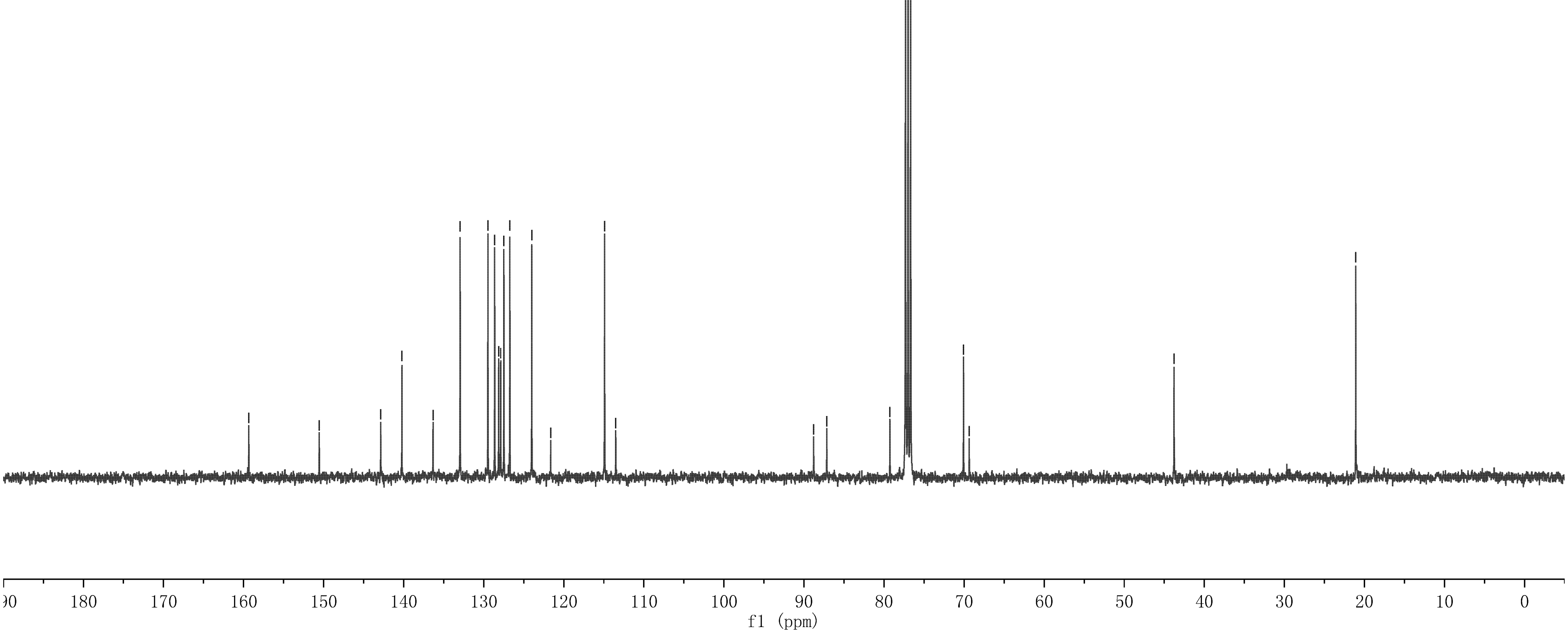

Parameter	Value
1 Title	CCM-2-126-C
2 Origin	
3 Solvent	CDCl3
4 Temperature	298.4
5 Number of Scans	770
6 Acquisition Time	1.0000
7 Acquisition Date	2022-10-11T17:02:08
8 Spectrometer Frequency	100.56
9 Spectral Width	26041.0

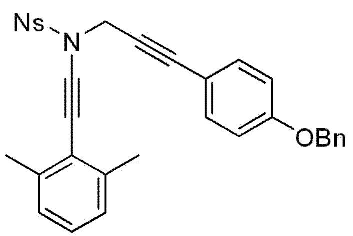

1l

Parameter	Value
1 Title	ccm-2-127-h
2 Origin	
3 Solvent	CDCl3
4 Temperature	297.5
5 Number of Scans	16
6 Acquisition Time	4.0002
7 Acquisition Date	2022-10-11T17:06:55
8 Spectrometer Frequency	399.90
9 Spectral Width	8012.0

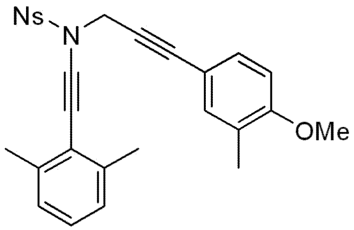

1m

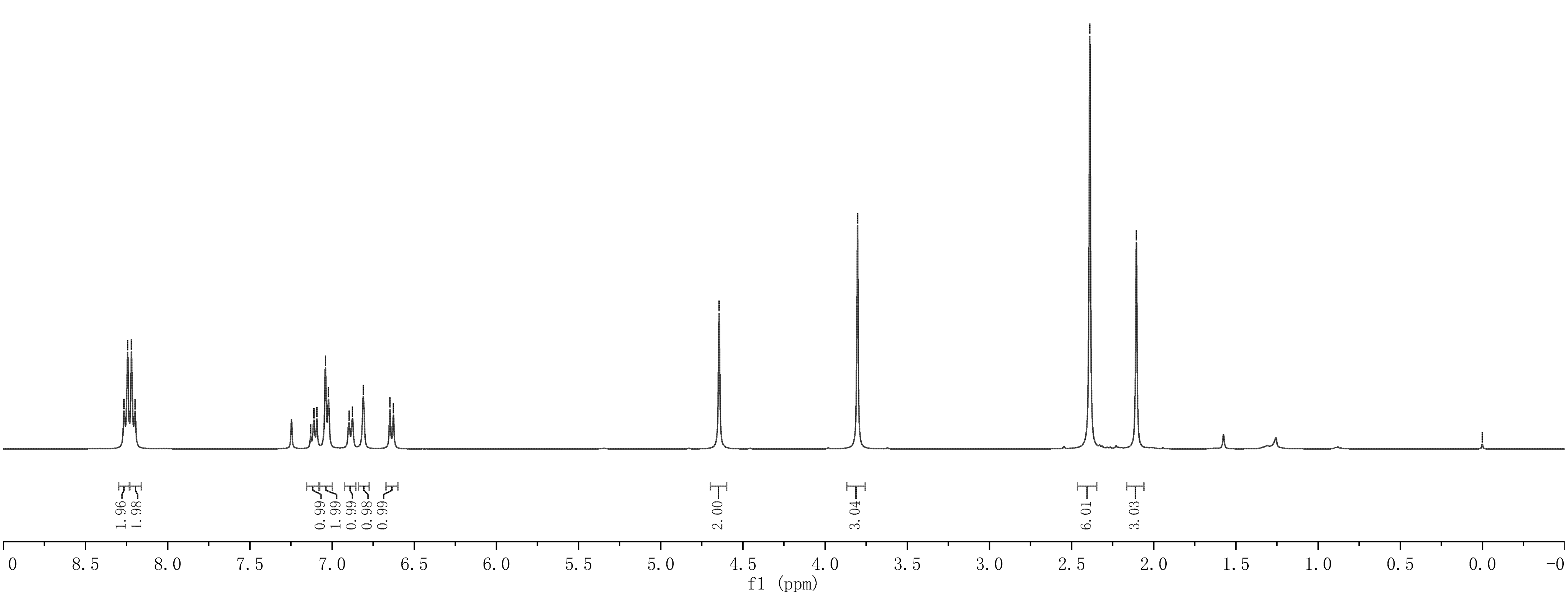

Parameter	Value
1 Title	ccm-2-127-c
2 Origin	
3 Solvent	CDCl3
4 Temperature	298.0
5 Number of Scans	700
6 Acquisition Time	1.0000
7 Acquisition Date	2022-10-11T17:32:44
8 Spectrometer Frequency	100.56
9 Spectral Width	26041.0

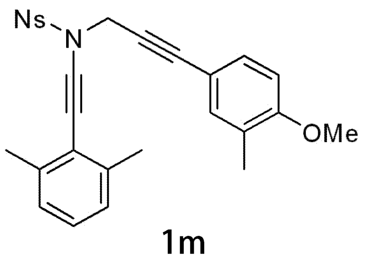

1m

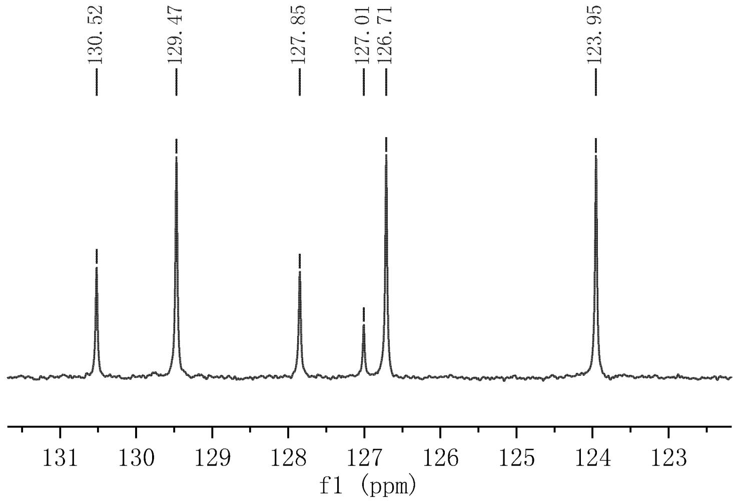

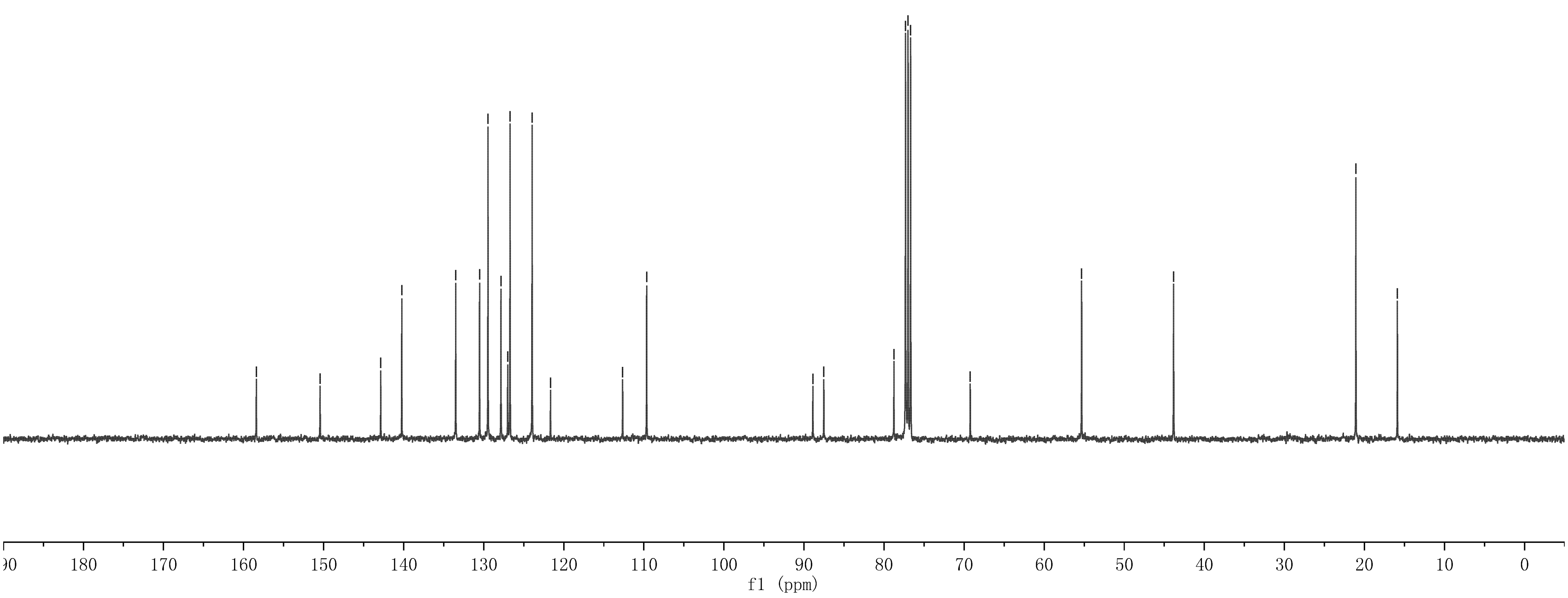

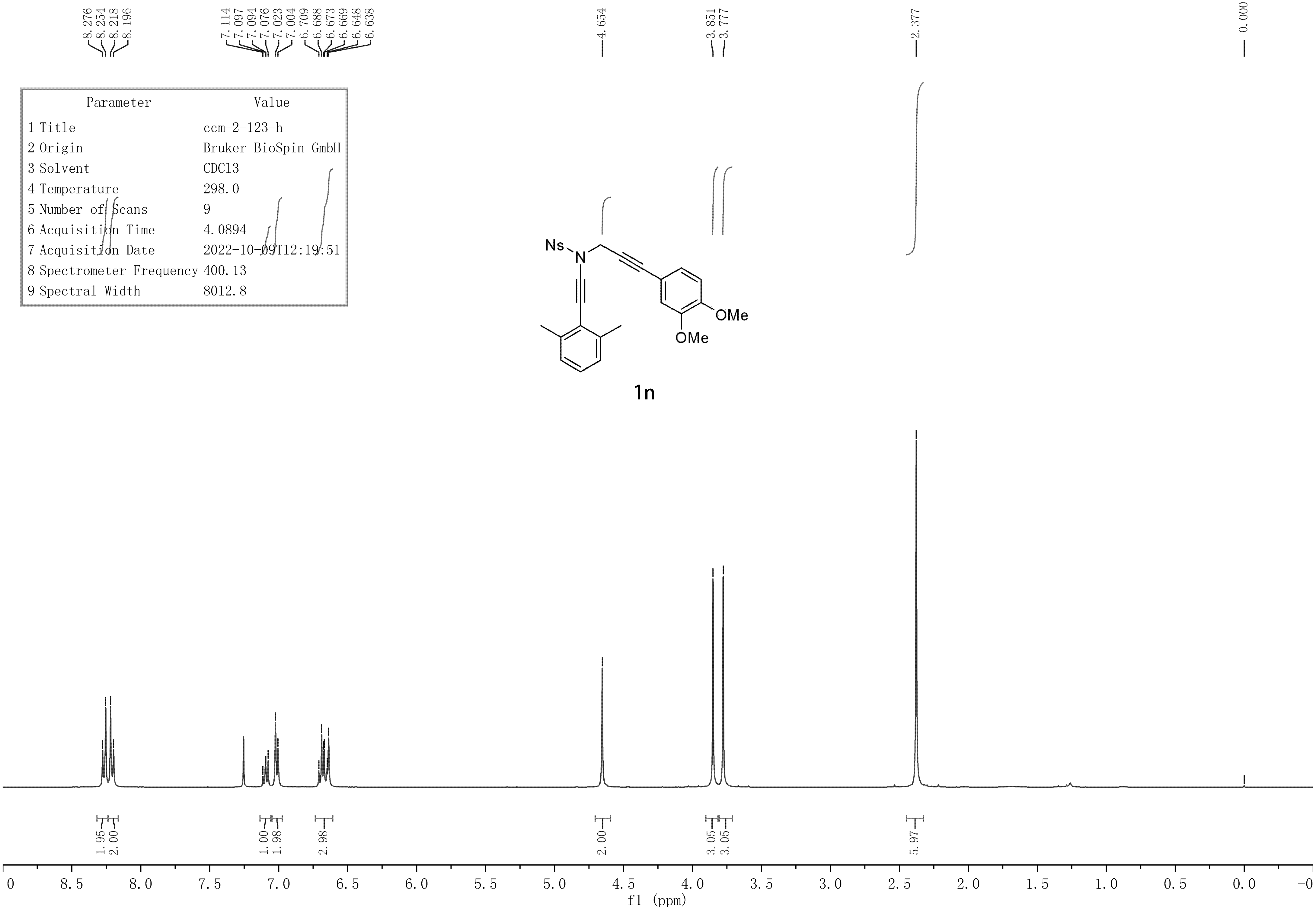

8.276
8.254
8.218
8.196
7.114
7.097
7.094
7.076
7.023
7.004
6.709
6.688
6.673
6.669
6.648
6.638
4.654
3.851
3.777
2.377
-0.000
Parameter Value
1 Title ccm-2-123-h
2 Origin Bruker BioSpin GmbH
3 Solvent CDCl3
4 Temperature 298.0
5 Number of Scans 9
6 Acquisition Time 4.0894
7 Acquisition Date 2022-10-09T12:19:51
8 Spectrometer Frequency 400.13
9 Spectral Width 8012.8
Ns
N
OMe
OMe
1n
1.95
2.00
1.00
1.98
2.98
2.00
3.05
3.05
5.97
8.5 8.0 7.5 7.0 6.5 6.0 5.5 5.0 4.5 4.0 3.5 3.0 2.5 2.0 1.5 1.0 0.5 0.0
f1 (ppm)

Parameter	Value
1 Title	ccm-2-123-c
2 Origin	Bruker BioSpin GmbH
3 Solvent	CDCl3
4 Temperature	300.0
5 Number of Scans	49
6 Acquisition Time	1.3631
7 Acquisition Date	2022-10-09T12:21:46
8 Spectrometer Frequency	100.61
9 Spectral Width	24038.5

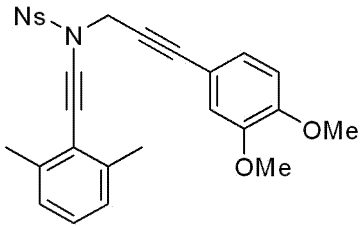

1n

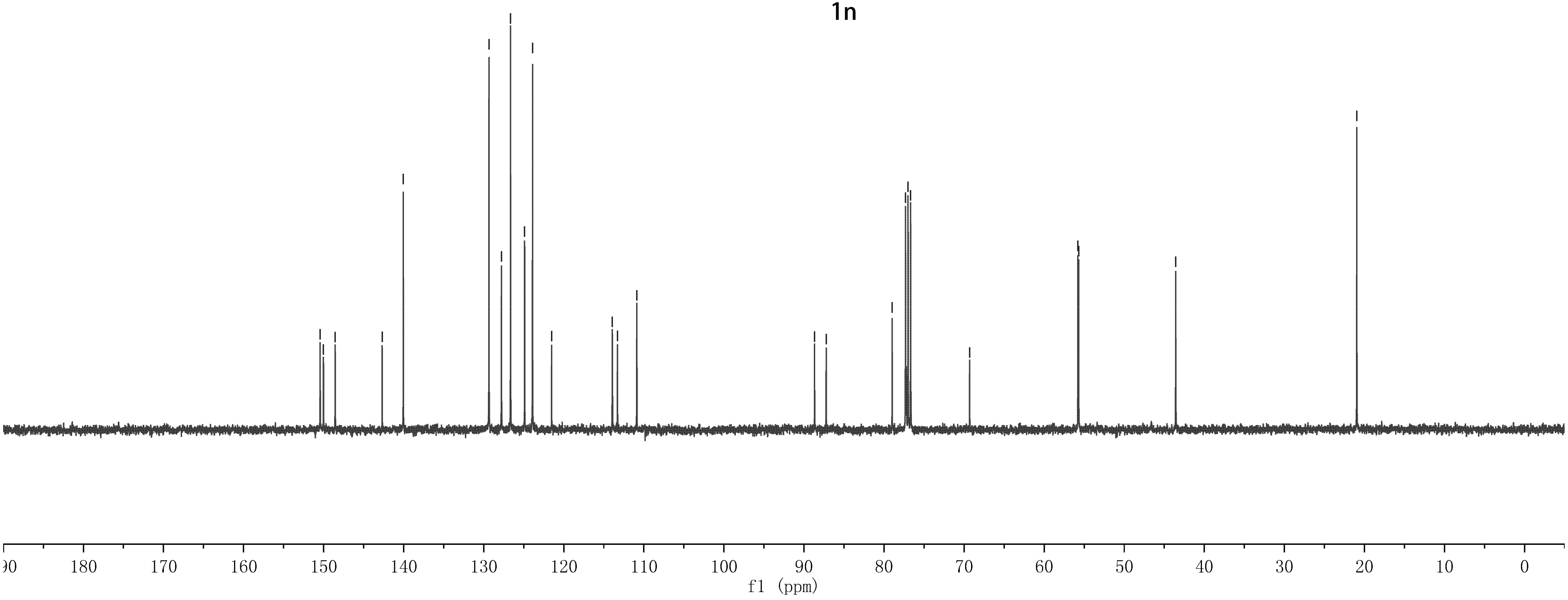

Parameter	Value
1 Title	CCM-2-101-H
2 Origin	Bruker BioSpin GmbH
3 Solvent	CDCl3
4 Temperature	298.0
5 Number of Scans	7
6 Acquisition Time	4.0894
7 Acquisition Date	2022-09-29T20:26:30
8 Spectrometer Frequency	400.13
9 Spectral Width	8012.8

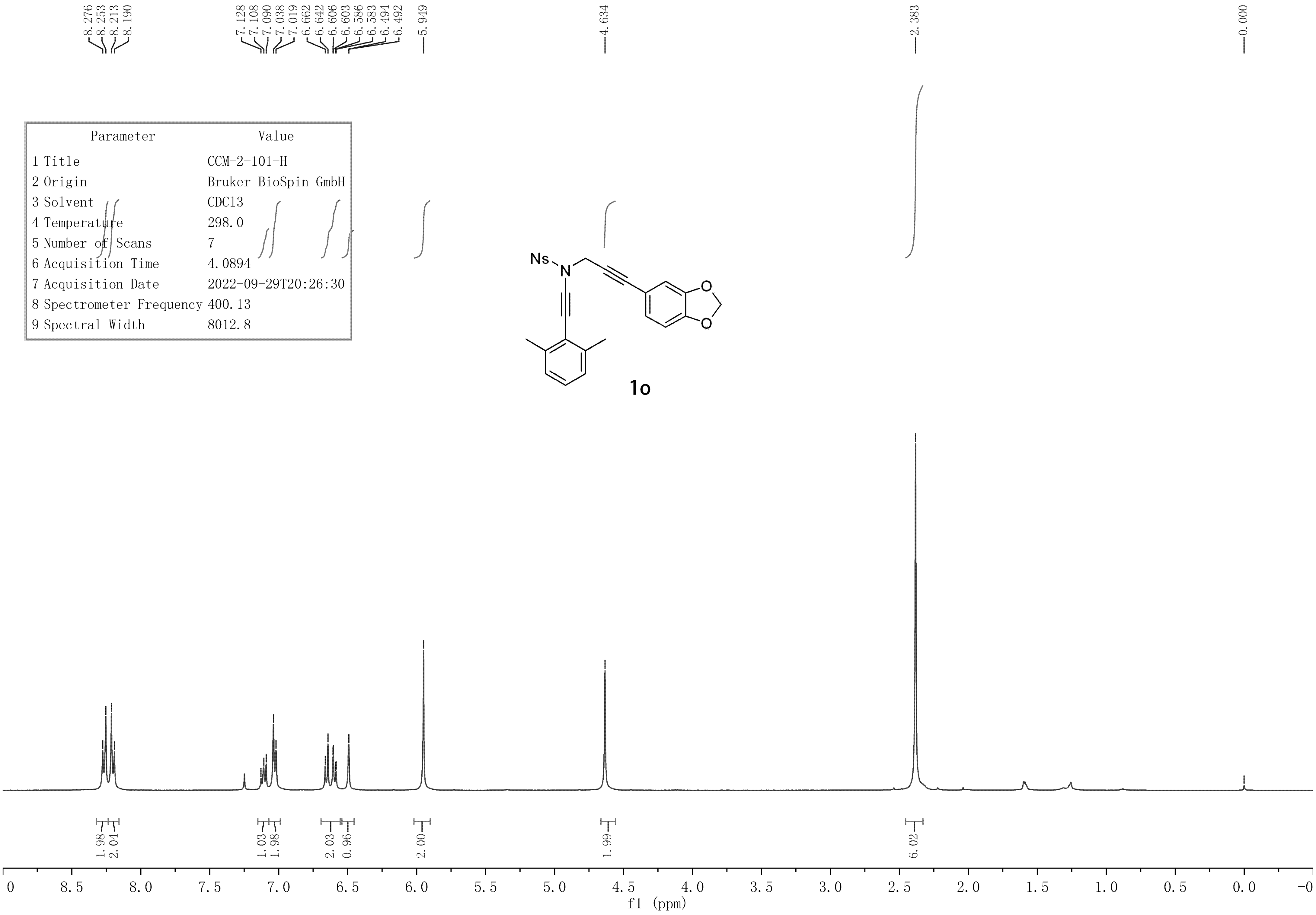

1o

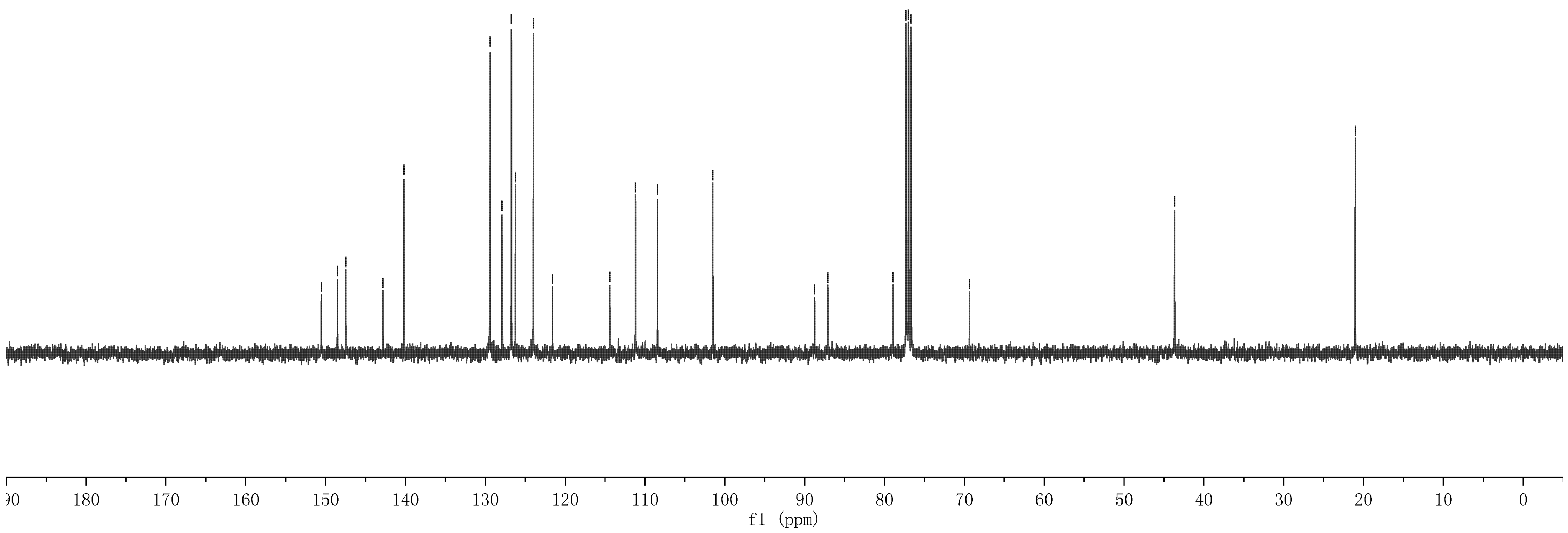

Parameter	Value
1 Title	CCM-2-101-C
2 Origin	Bruker BioSpin GmbH
3 Solvent	CDCl3
4 Temperature	300.0
5 Number of Scans	75
6 Acquisition Time	1.3631
7 Acquisition Date	2022-09-29T20:29:16
8 Spectrometer Frequency	100.61
9 Spectral Width	24038.5

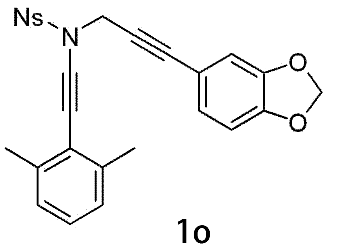

1o

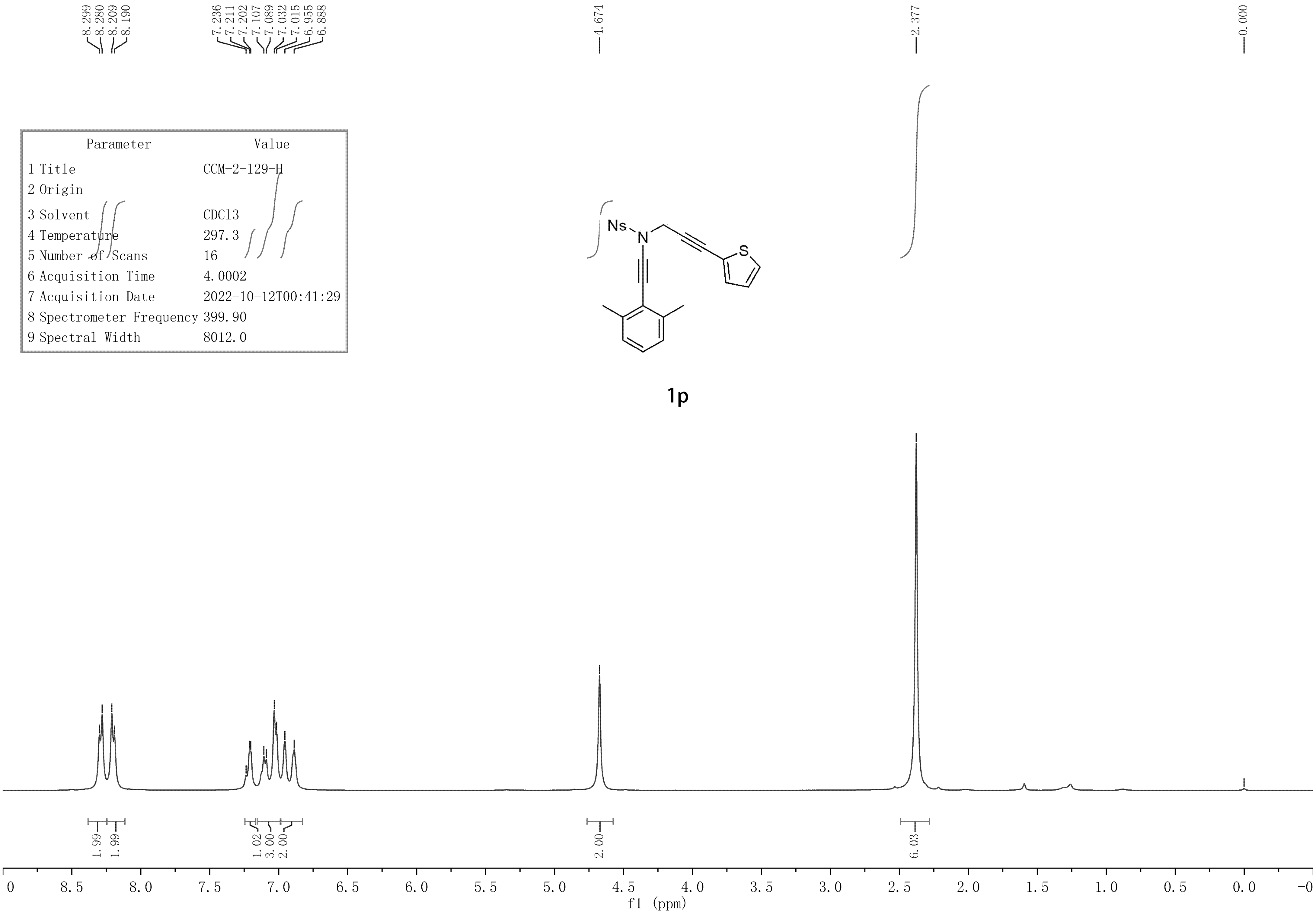

Parameter	Value
1 Title	CCM-2-129-H
2 Origin	
3 Solvent	CDCl3
4 Temperature	297.3
5 Number of Scans	16
6 Acquisition Time	4.0002
7 Acquisition Date	2022-10-12T00:41:29
8 Spectrometer Frequency	399.90
9 Spectral Width	8012.0

1p

Parameter	Value
1 Title	CCM-2-129-C
2 Origin	
3 Solvent	CDCl3
4 Temperature	297.3
5 Number of Scans	500
6 Acquisition Time	1.0000
7 Acquisition Date	2022-10-12T01:00:36
8 Spectrometer Frequency	100.56
9 Spectral Width	26041.0

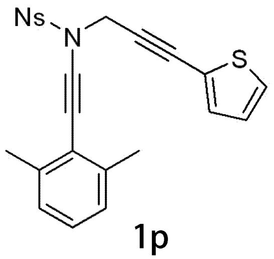

1p

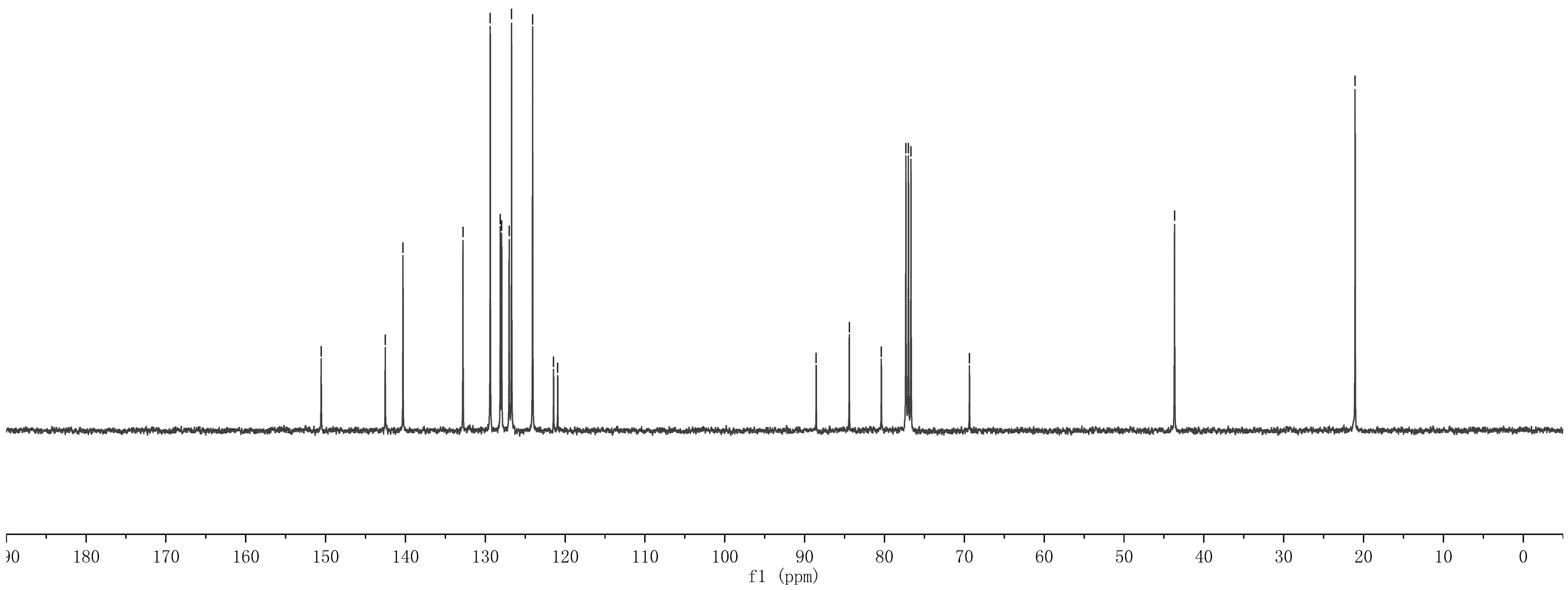

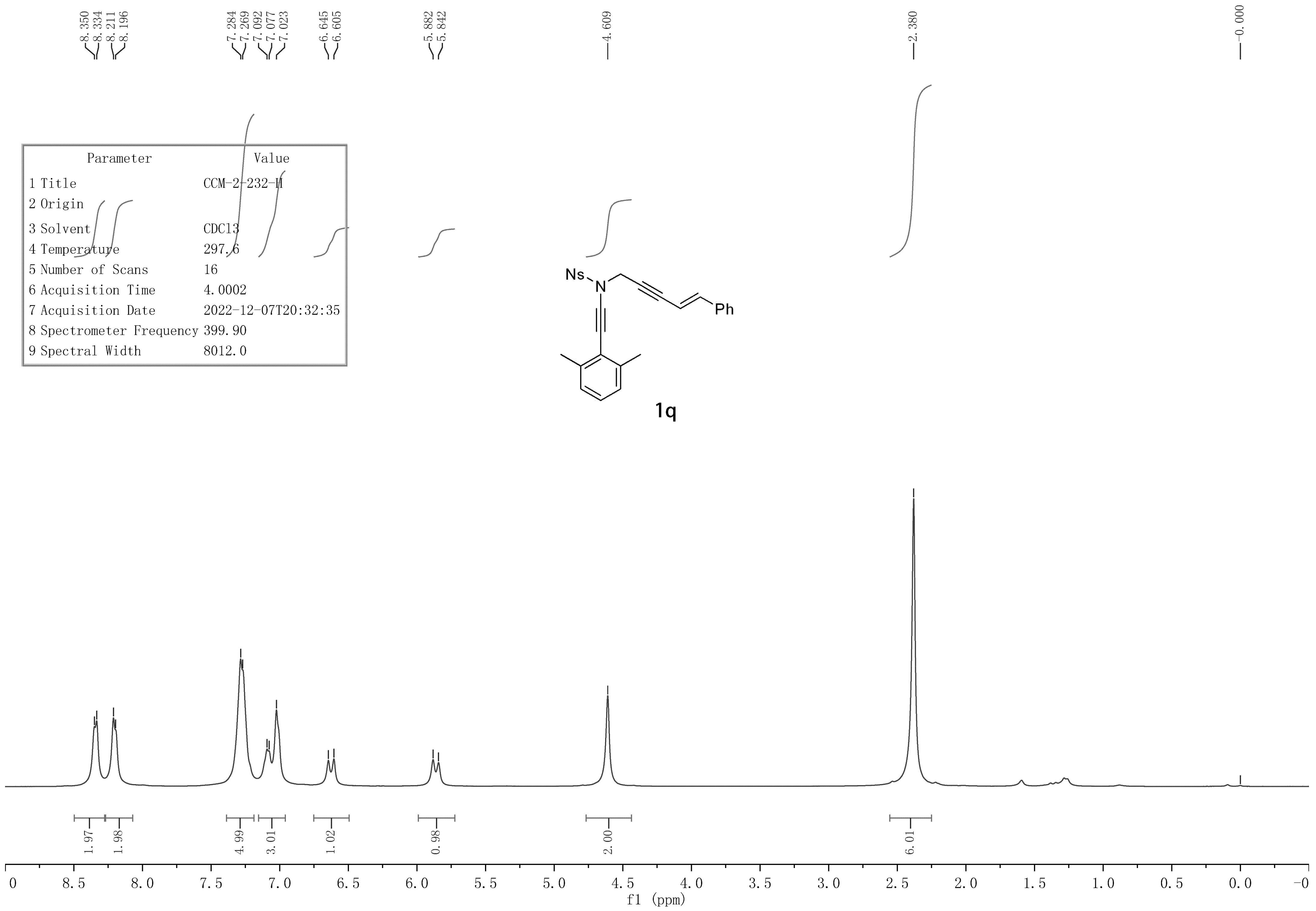
8.350
8.334
8.211
8.196
7.284
7.269
7.092
7.077
7.023
6.645
6.605
5.882
5.842
4.609
2.380
-0.000
Parameter
Value
1 Title
CCM-2-232-H
2 Origin
3 Solvent
CDCl3
4 Temperature
297.6
5 Number of Scans
16
6 Acquisition Time
4.0002
7 Acquisition Date
2022-12-07T20:32:35
8 Spectrometer Frequency
399.90
9 Spectral Width
8012.0
Ns
N
Ph
1q
1.97
1.98
4.99
3.01
1.02
0.98
2.00
6.01
f1 (ppm)

Parameter	Value
1 Title	CCM-2-232-C
2 Origin	
3 Solvent	CDCl3
4 Temperature	297.8
5 Number of Scans	500
6 Acquisition Time	1.0000
7 Acquisition Date	2022-12-07T20:51:28
8 Spectrometer Frequency	100.56
9 Spectral Width	26041.0

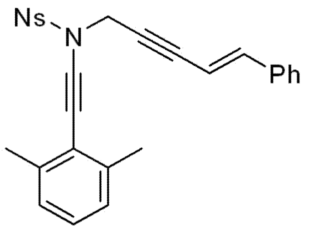

1q

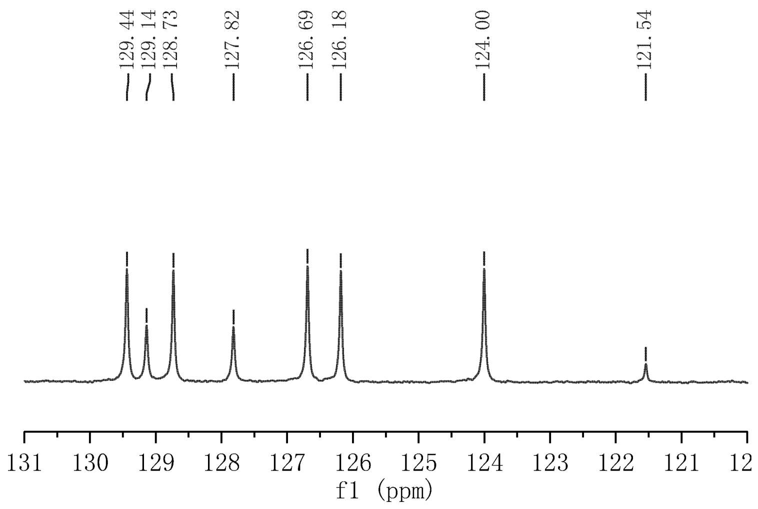

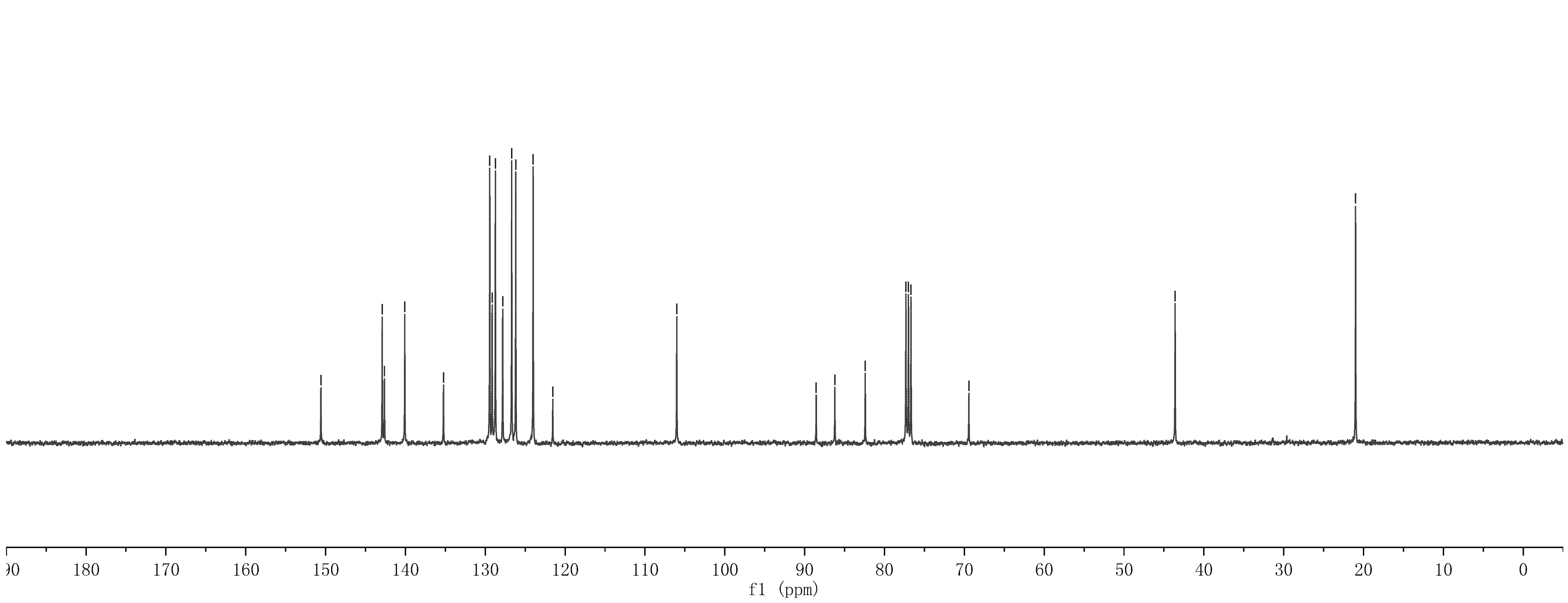

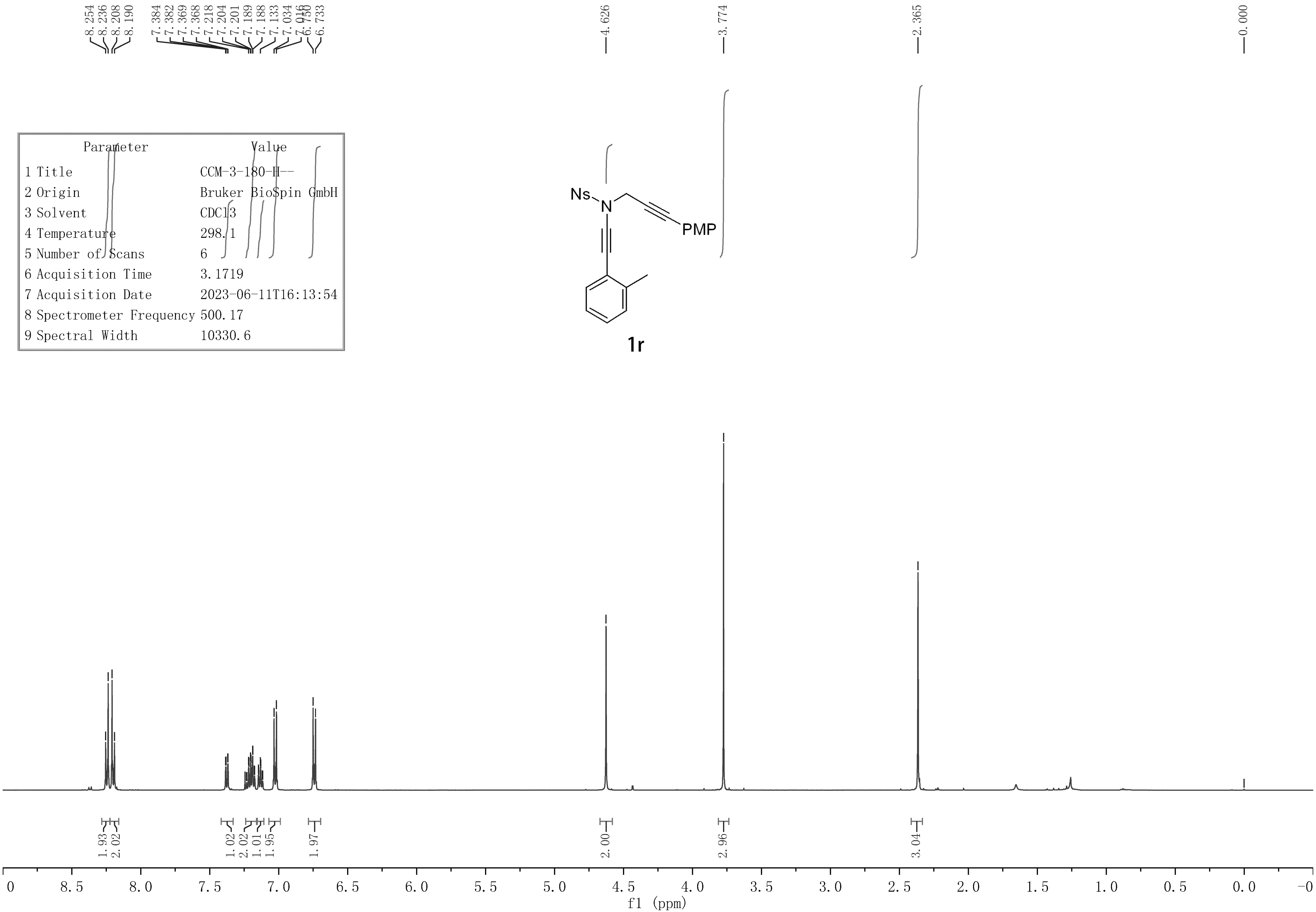

8.254
8.236
8.208
8.190
7.384
7.382
7.369
7.368
7.218
7.204
7.201
7.189
7.188
7.133
7.034
7.016
6.750
6.733
4.626
3.774
2.365
0.000
Parameter Value
1 Title CCM-3-180-H--
2 Origin Bruker BioSpin GmbH
3 Solvent CDCl3
4 Temperature 298.1
5 Number of Scans 6
6 Acquisition Time 3.1719
7 Acquisition Date 2023-06-11T16:13:54
8 Spectrometer Frequency 500.17
9 Spectral Width 10330.6
Ns
N
PMP
1r
1.93
2.02
1.02
2.02
1.01
1.95
1.97
2.00
2.96
3.04
8.5 8.0 7.5 7.0 6.5 6.0 5.5 5.0 4.5 4.0 3.5 3.0 2.5 2.0 1.5 1.0 0.5 0.0
f1 (ppm)

Parameter	Value
1 Title	CCM-3-180-C-
2 Origin	Bruker BioSpin GmbH
3 Solvent	CDCl3
4 Temperature	298.2
5 Number of Scans	21
6 Acquisition Time	1.1010
7 Acquisition Date	2023-06-11T16:17:04
8 Spectrometer Frequency	125.77
9 Spectral Width	29761.9

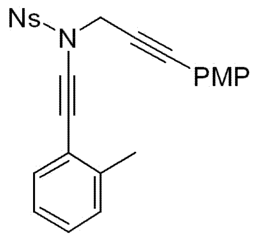

1r

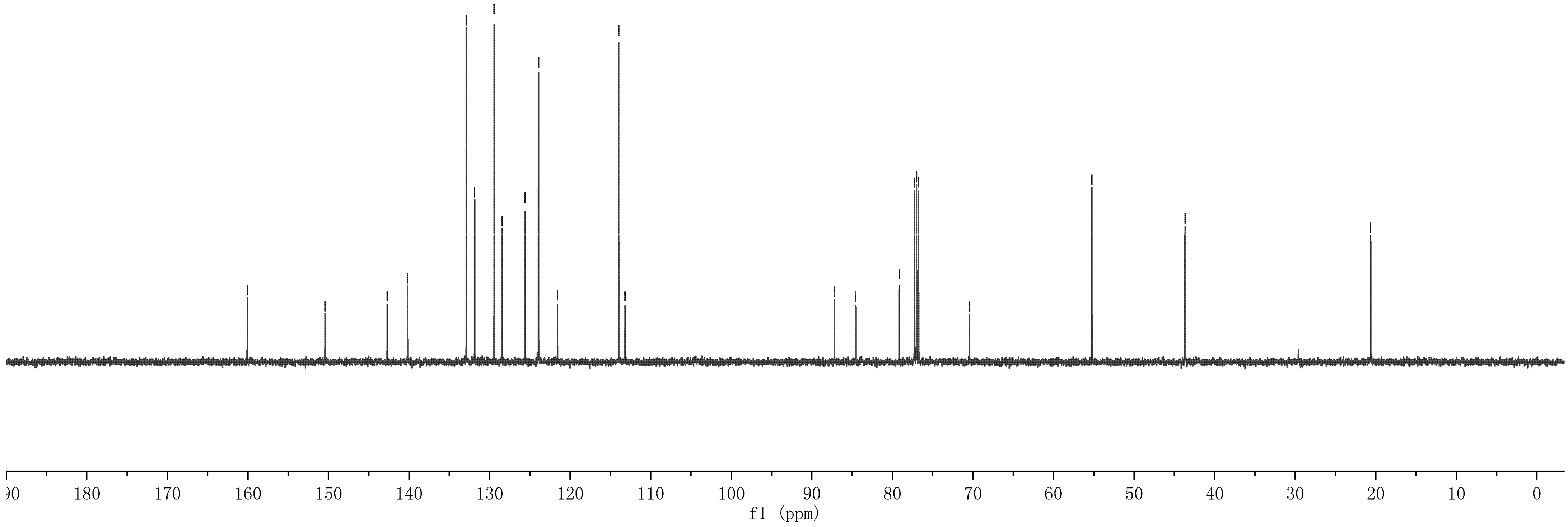

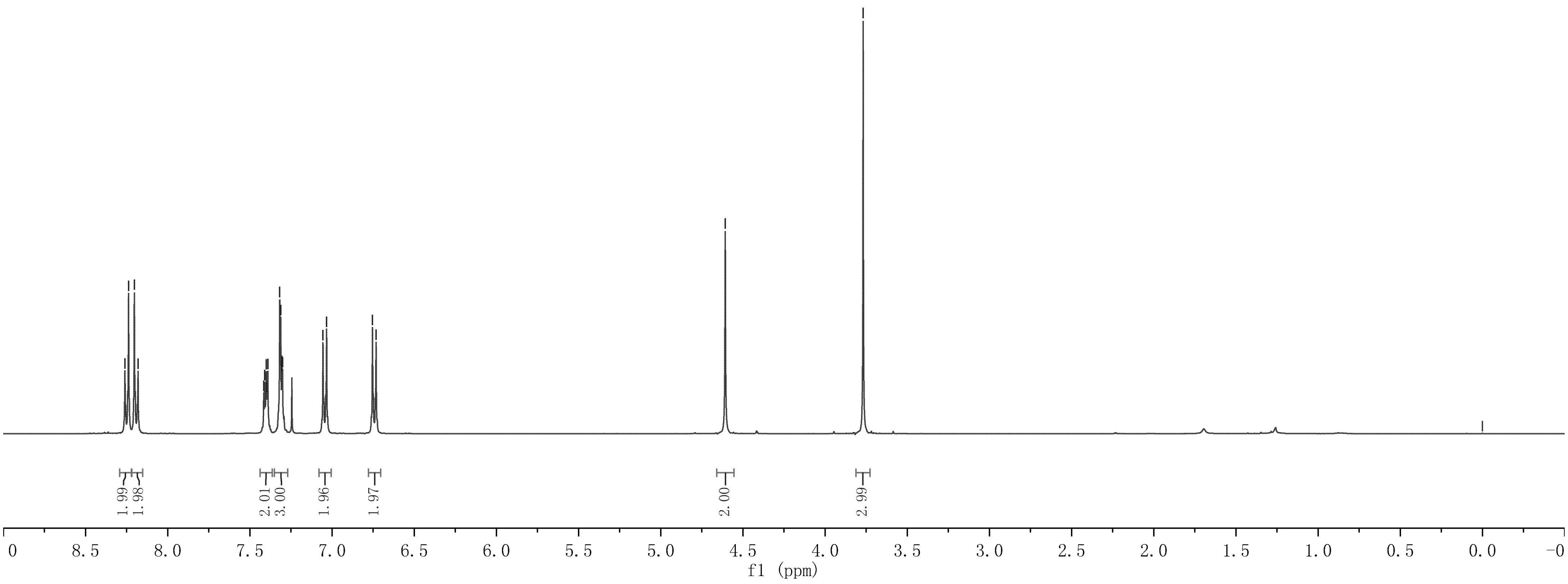

Parameter	Value
1 Title	ccm-3-191-h
2 Origin	Bruker BioSpin GmbH
3 Solvent	CDCl3
4 Temperature	294.8
5 Number of Scans	6
6 Acquisition Time	3.9846
7 Acquisition Date	2023-06-12T20:14:29
8 Spectrometer Frequency	399.93
9 Spectral Width	8223.7

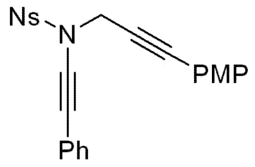

1s

Parameter	Value
1 Title	ccm-3-191-c-
2 Origin	Bruker BioSpin GmbH
3 Solvent	CDCl3
4 Temperature	295.3
5 Number of Scans	39
6 Acquisition Time	1.3631
7 Acquisition Date	2023-06-12T20:18:03
8 Spectrometer Frequency	100.56
9 Spectral Width	24038.5

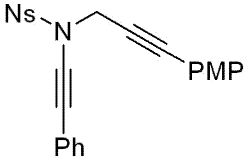

1s

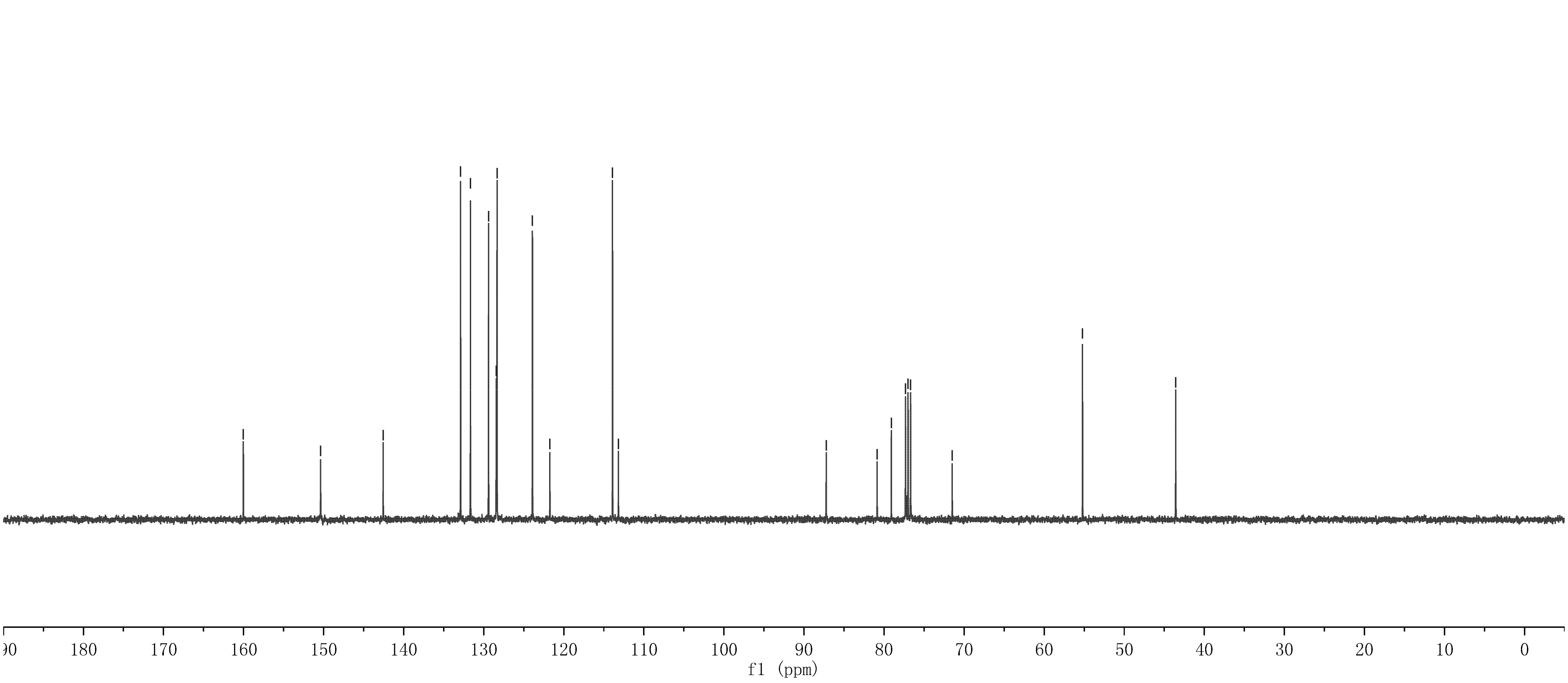

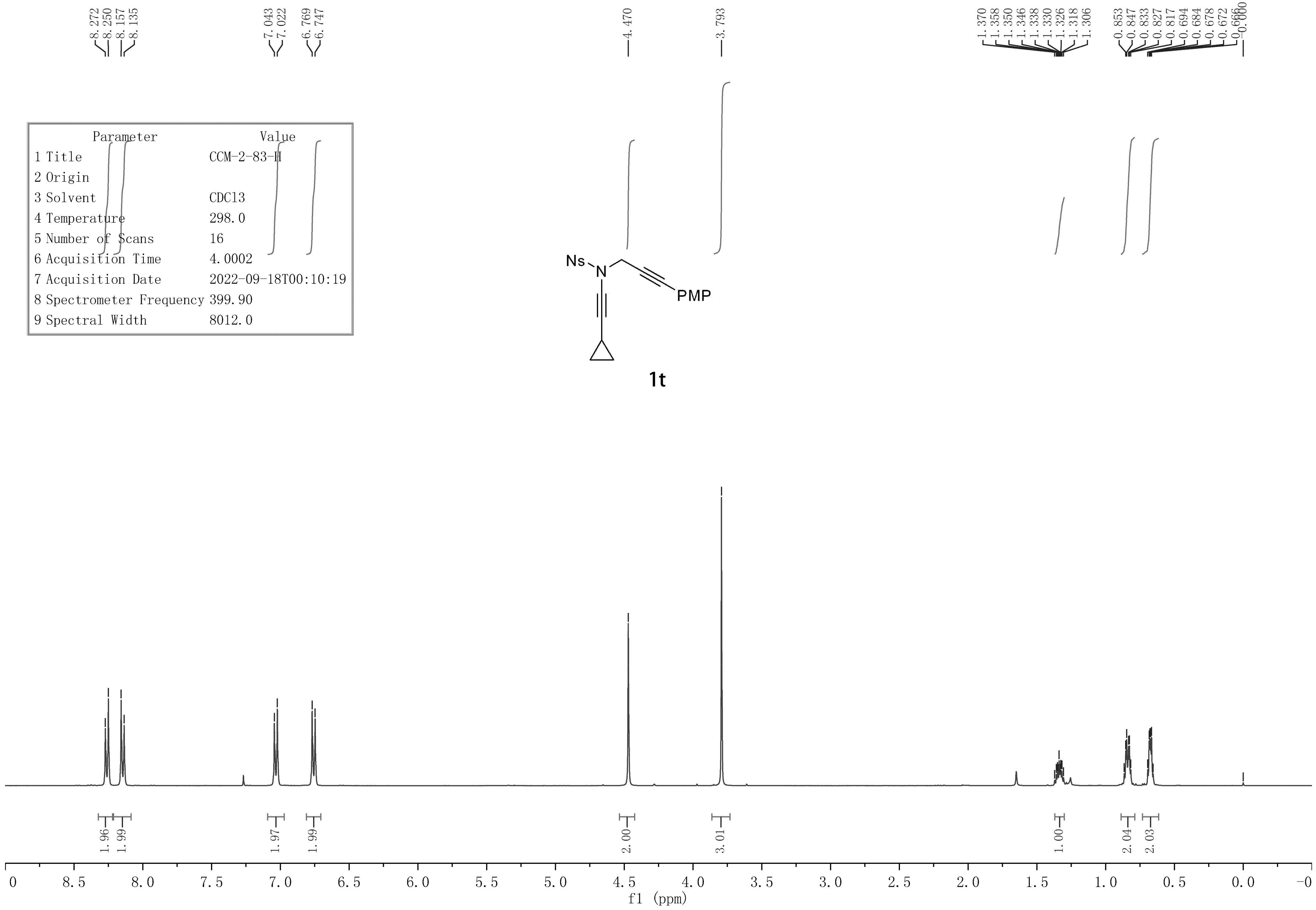
8.272
8.250
8.157
8.135
7.043
7.022
6.769
6.747
4.470
3.793
1.370
1.358
1.350
1.346
1.338
1.330
1.326
1.318
1.306
0.853
0.847
0.833
0.827
0.817
0.694
0.684
0.678
0.672
0.666
0.000
Parameter Value
1 Title CCM-2-83-II
2 Origin
3 Solvent CDCl3
4 Temperature 298.0
5 Number of Scans 16
6 Acquisition Time 4.0002
7 Acquisition Date 2022-09-18T00:10:19
8 Spectrometer Frequency 399.90
9 Spectral Width 8012.0
Ns
N
PMP
1t
1.96
1.99
1.97
1.99
2.00
3.01
1.00
2.04
2.03
f1 (ppm)

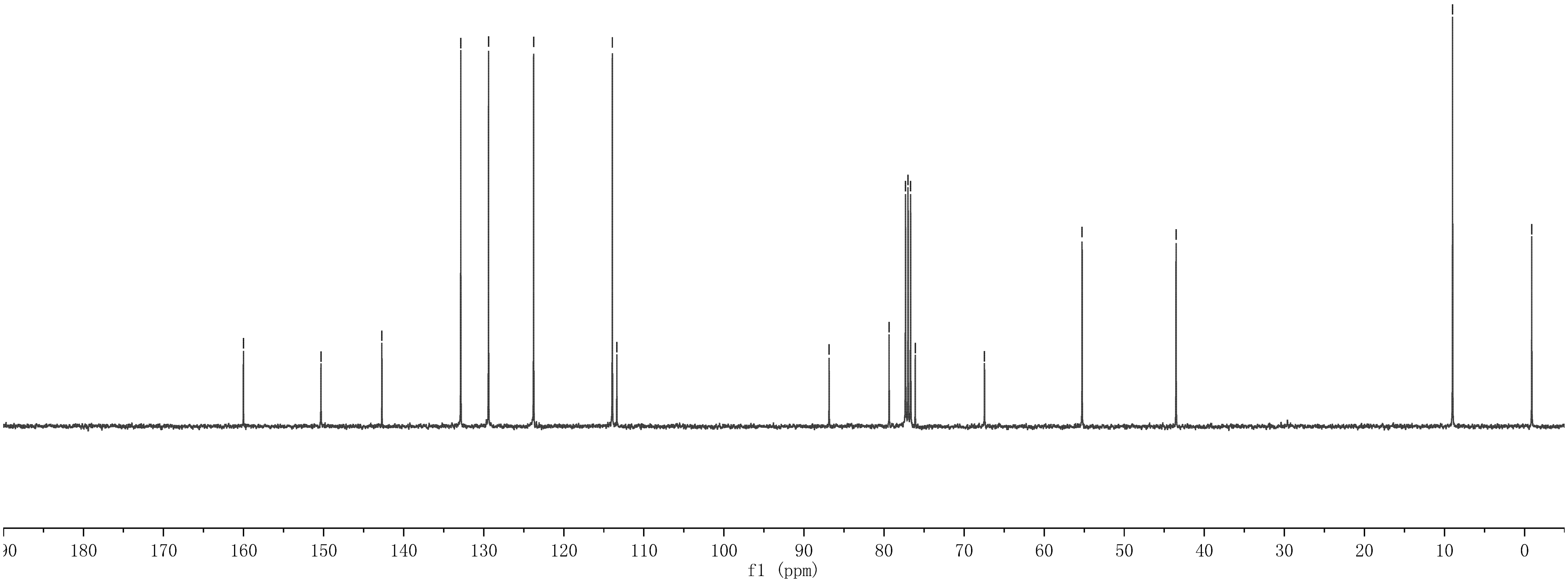

Parameter	Value
1 Title	CCM-2-83-C
2 Origin	
3 Solvent	CDCl3
4 Temperature	298.8
5 Number of Scans	400
6 Acquisition Time	1.0000
7 Acquisition Date	2022-09-18T00:26:15
8 Spectrometer Frequency	100.56
9 Spectral Width	26041.0

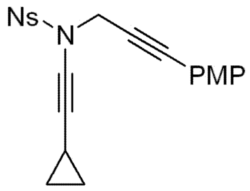

1t

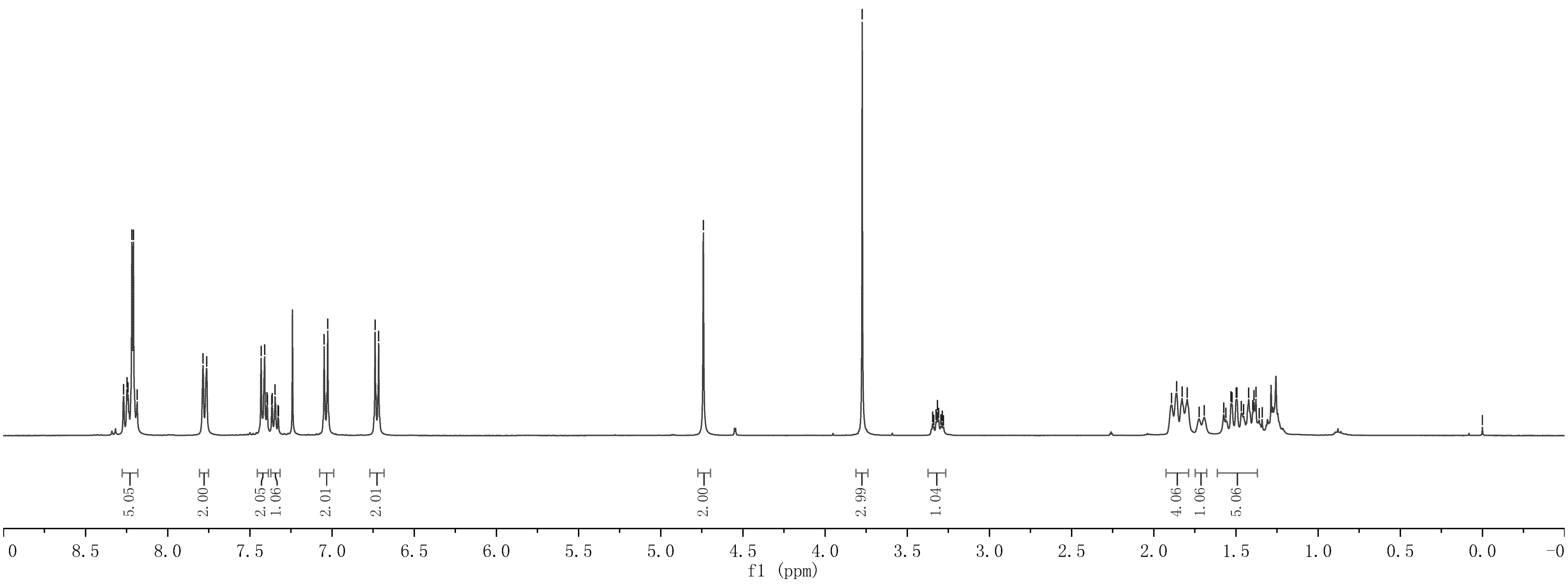

Parameter	Value
1 Title	ccm-3-143-h-
2 Origin	Bruker BioSpin GmbH
3 Solvent	CDCl3
4 Temperature	298.0
5 Number of Scans	7
6 Acquisition Time	4.0894
7 Acquisition Date	2023-05-11T20:50:11
8 Spectrometer Frequency	400.13
9 Spectral Width	8012.8

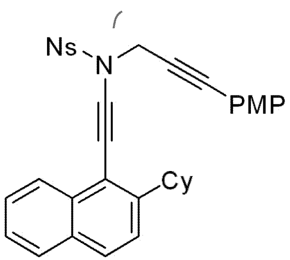

1x

Parameter	Value
1 Title	CCM-3-143-C
2 Origin	
3 Solvent	CDCl3
4 Temperature	297.4
5 Number of Scans	600
6 Acquisition Time	1.0000
7 Acquisition Date	2023-05-12T02:47:20
8 Spectrometer Frequency	100.56
9 Spectral Width	26041.0

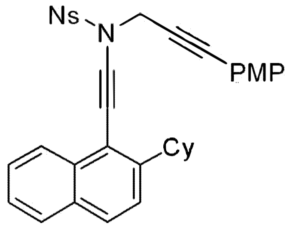

1x

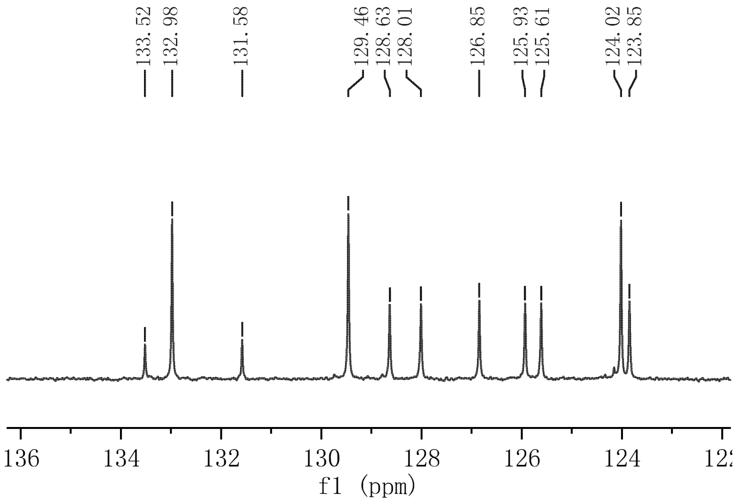

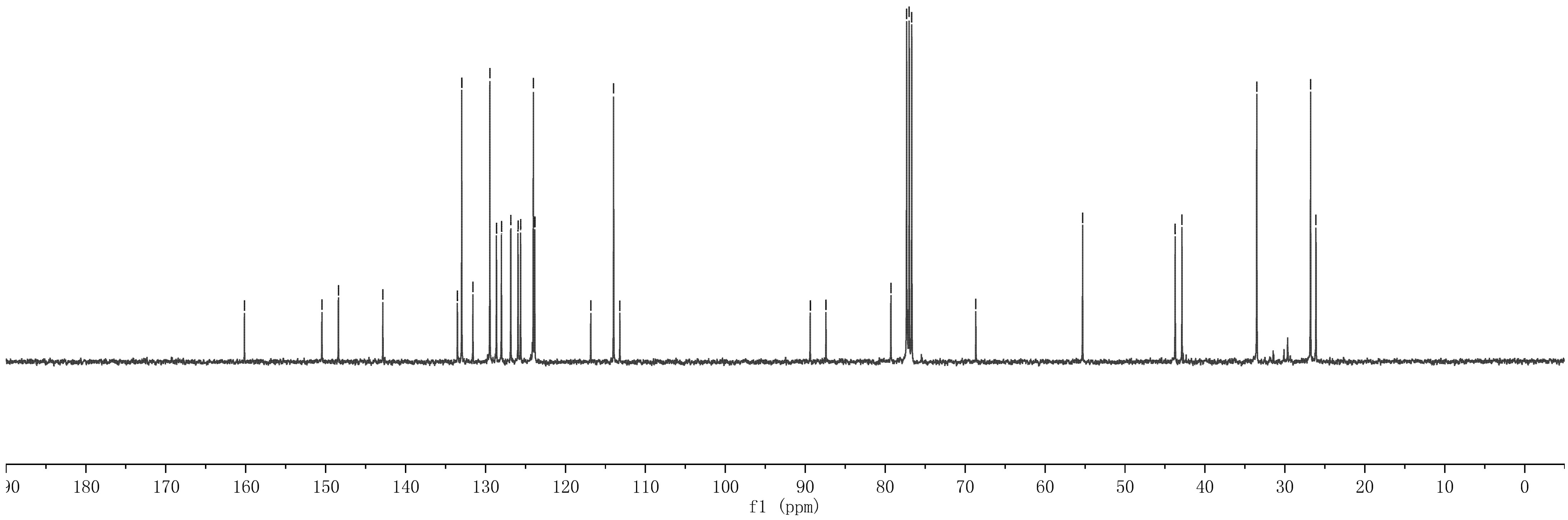

Parameter	Value
1 Title	CCM-4-7-H
2 Origin	
3 Solvent	CDCl3
4 Temperature	299.0
5 Number of Scans	16
6 Acquisition Time	4.0002
7 Acquisition Date	2023-07-25T05:58:45
8 Spectrometer Frequency	399.90
9 Spectral Width	8012.0

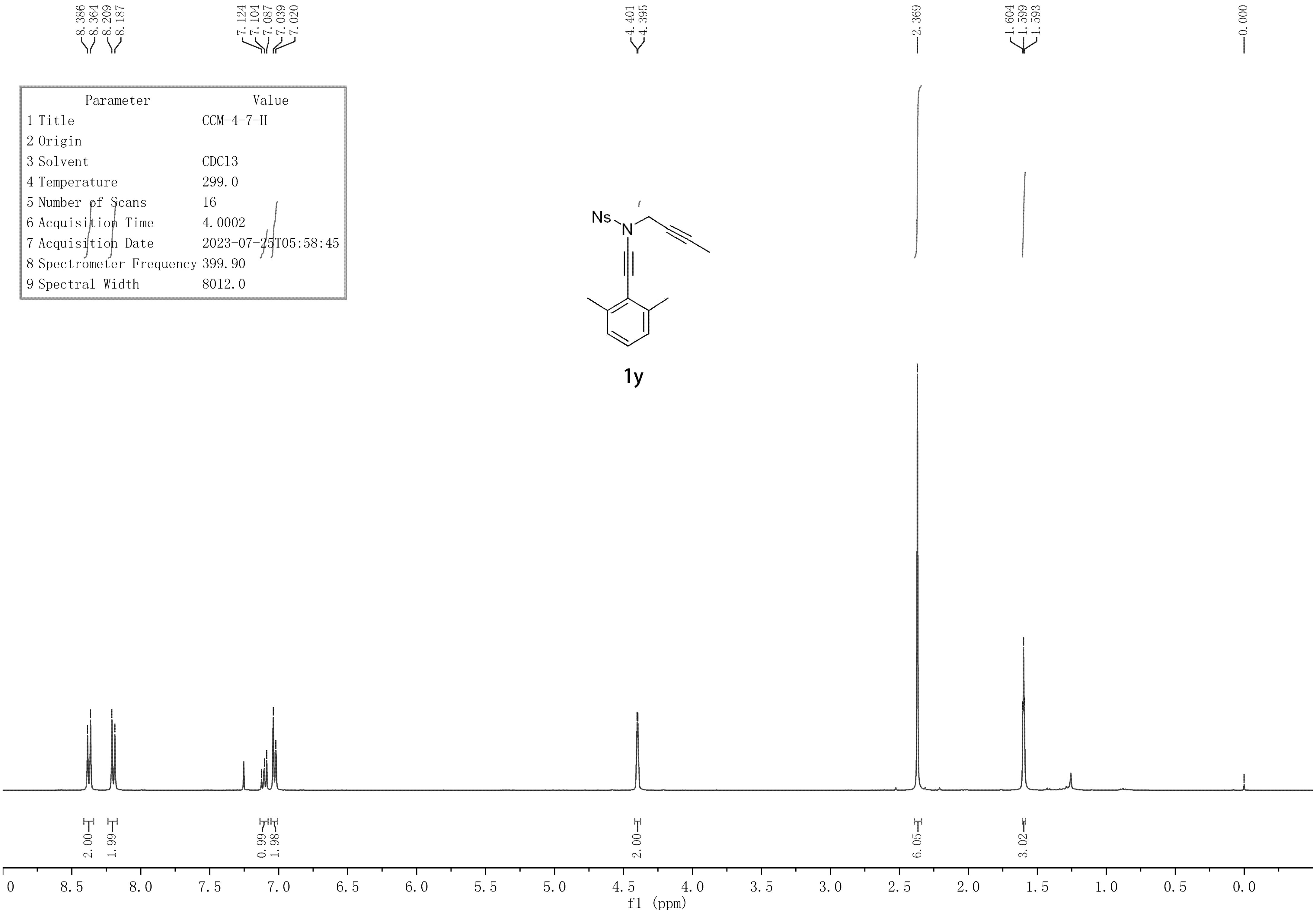

Parameter	Value
1 Title	CCM-4-7-C
2 Origin	
3 Solvent	CDCl3
4 Temperature	298.8
5 Number of Scans	500
6 Acquisition Time	1.0000
7 Acquisition Date	2023-07-25T06:17:59
8 Spectrometer Frequency	100.56
9 Spectral Width	26041.0

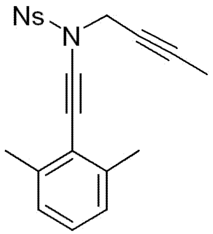

1y

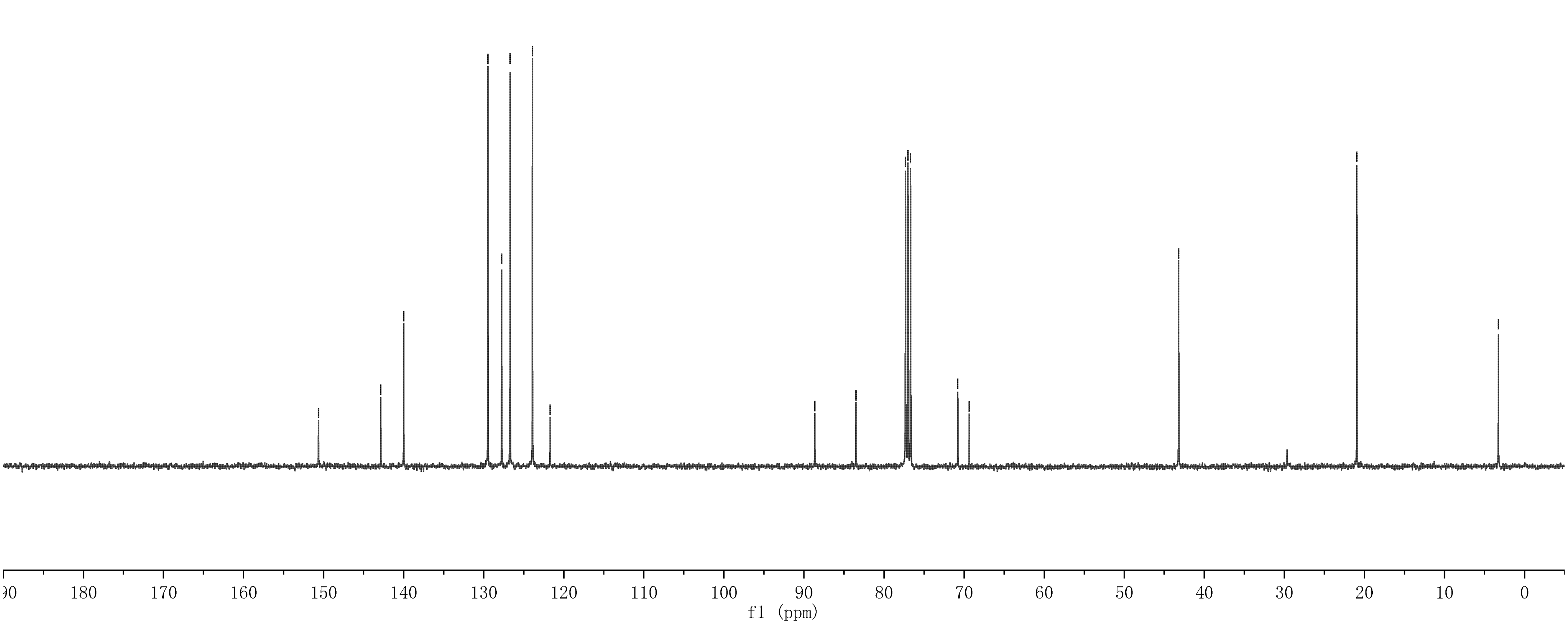

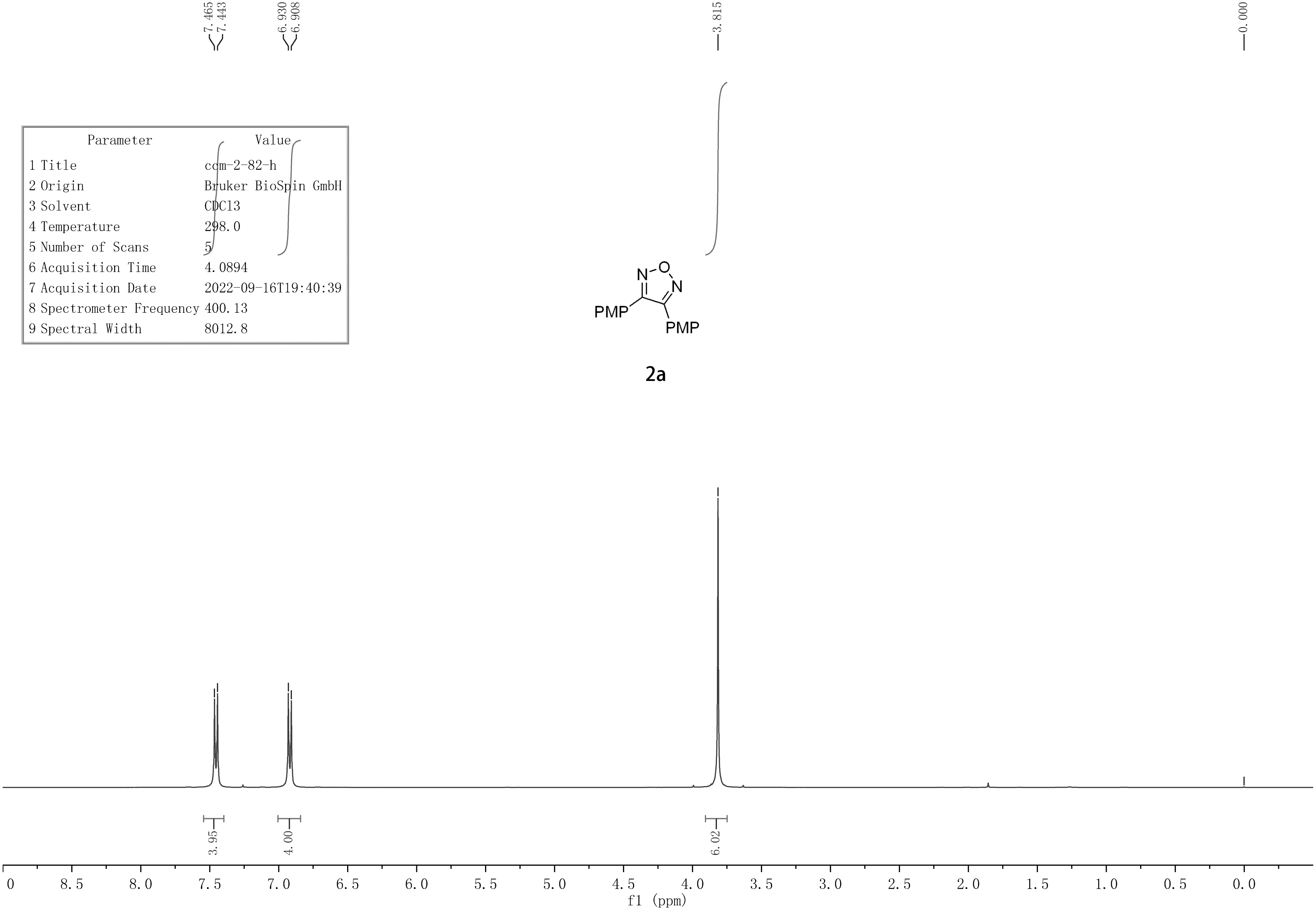

Parameter	Value
1 Title	ccm-2-82-h
2 Origin	Bruker BioSpin GmbH
3 Solvent	CDCl3
4 Temperature	298.0
5 Number of Scans	5
6 Acquisition Time	4.0894
7 Acquisition Date	2022-09-16T19:40:39
8 Spectrometer Frequency	400.13
9 Spectral Width	8012.8

2a

Parameter	Value
1 Title	ccm-2-82-c
2 Origin	Bruker BioSpin GmbH
3 Solvent	CDCl3
4 Temperature	300.0
5 Number of Scans	15
6 Acquisition Time	1.3631
7 Acquisition Date	2022-09-16T19:42:02
8 Spectrometer Frequency	100.61
9 Spectral Width	24038.5

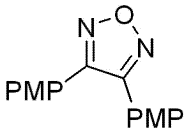

2a

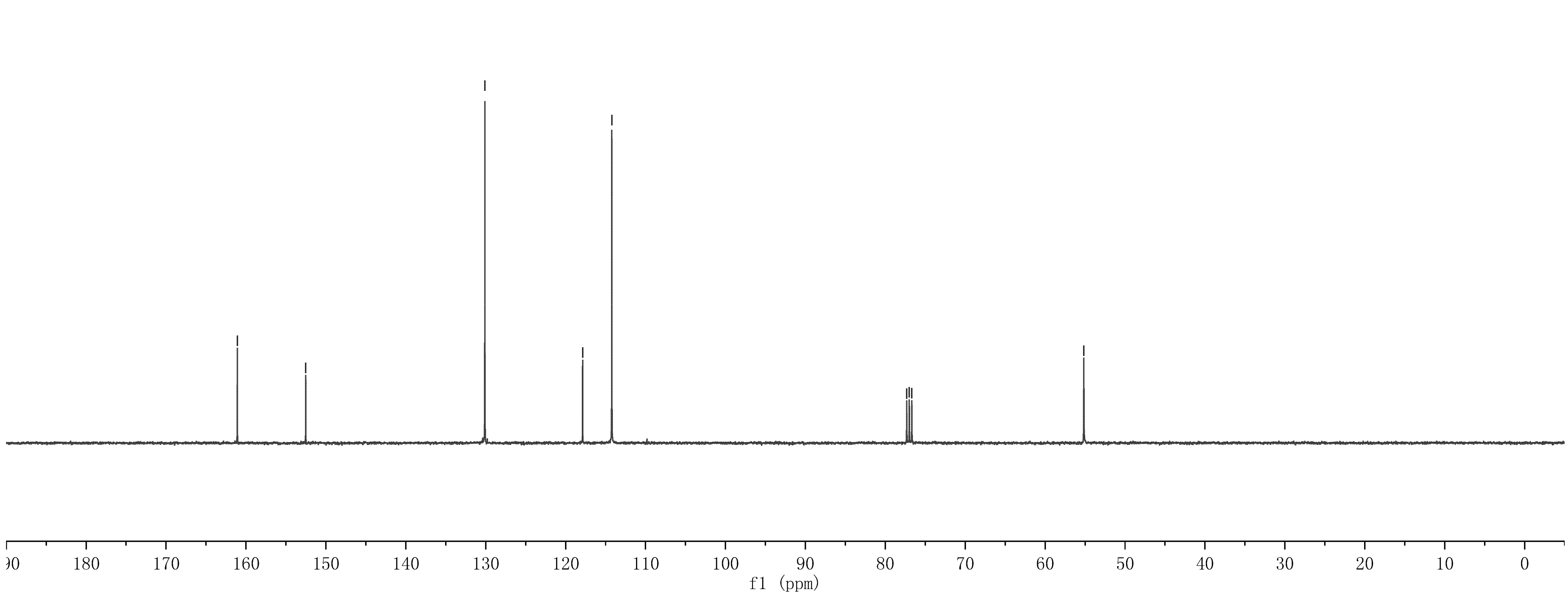

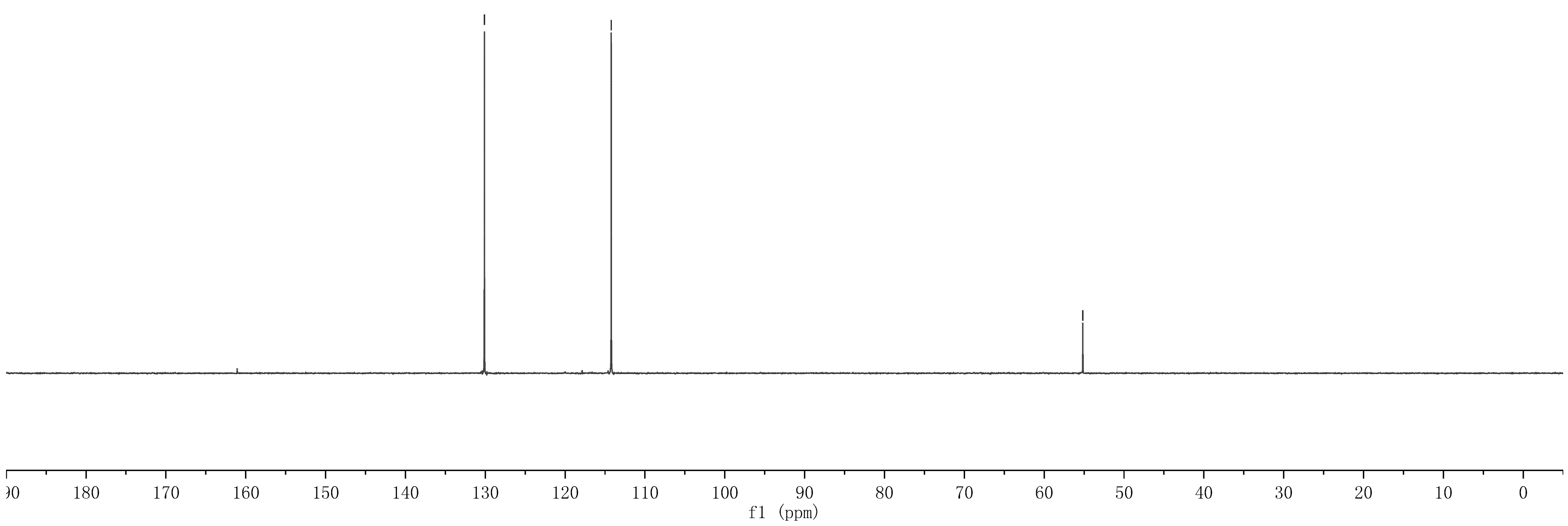

Parameter	Value
1 Title	ccm-2-82-c135
2 Origin	Bruker BioSpin GmbH
3 Solvent	CDCl3
4 Temperature	300.0
5 Number of Scans	18
6 Acquisition Time	1.3631
7 Acquisition Date	2022-09-16T19:43:30
8 Spectrometer Frequency	100.61
9 Spectral Width	24038.5

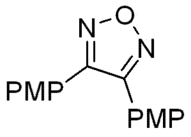

2a

Parameter	Value
1 Title	ccm-2-231-h
2 Origin	
3 Solvent	CDCl3
4 Temperature	297.1
5 Number of Scans	16
6 Acquisition Time	4.0002
7 Acquisition Date	2022-12-13T18:51:04
8 Spectrometer Frequency	399.90
9 Spectral Width	8012.0

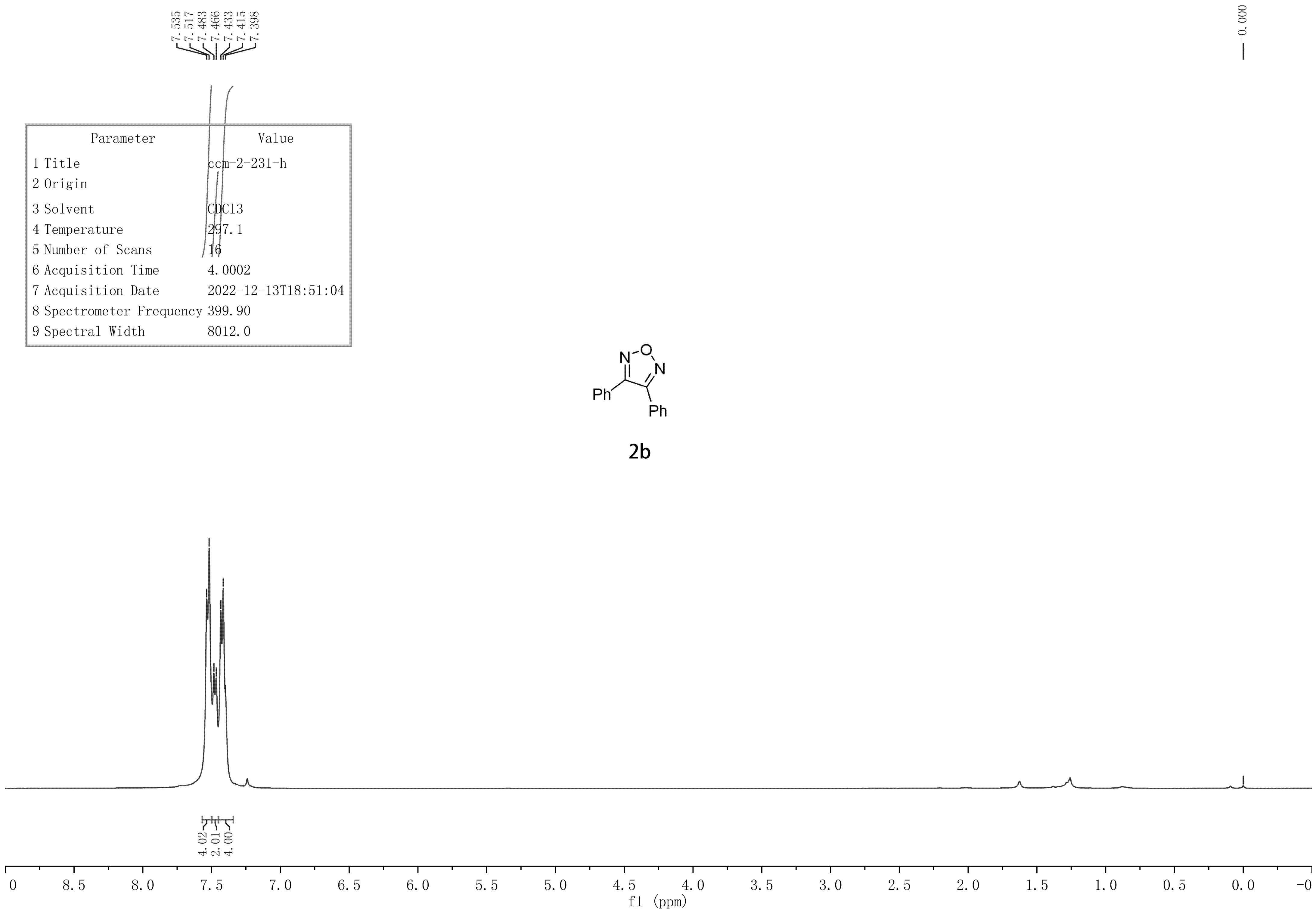

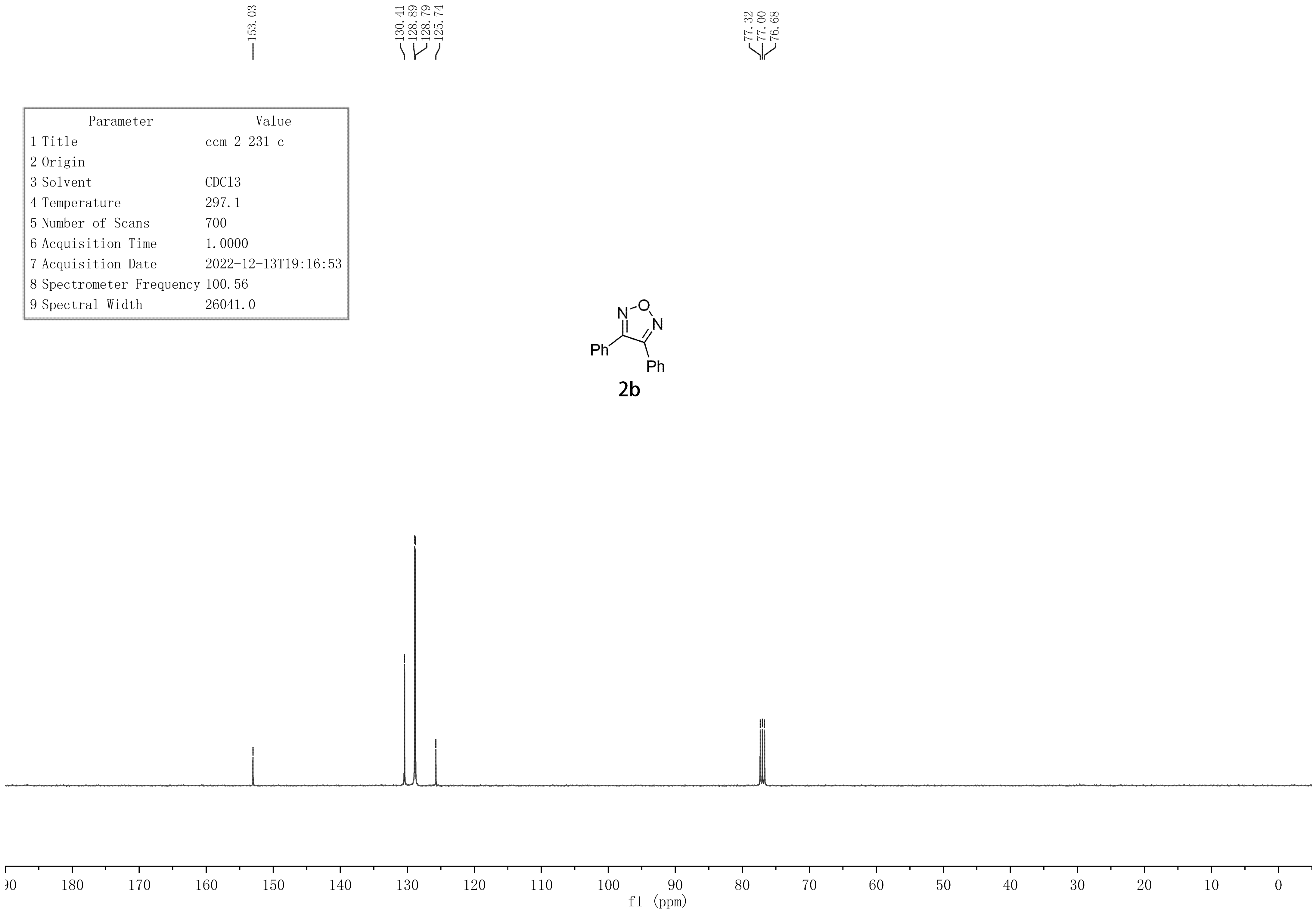

Parameter	Value
1 Title	ccm-2-231-c
2 Origin	
3 Solvent	CDCl3
4 Temperature	297.1
5 Number of Scans	700
6 Acquisition Time	1.0000
7 Acquisition Date	2022-12-13T19:16:53
8 Spectrometer Frequency	100.56
9 Spectral Width	26041.0

2b

Parameter	Value
1 Title	ccm-2-240-h
2 Origin	Bruker BioSpin GmbH
3 Solvent	CDCl3
4 Temperature	298.0
5 Number of Scans	9
6 Acquisition Time	4.0894
7 Acquisition Date	2023-02-01T09:37:29
8 Spectrometer Frequency	400.13
9 Spectral Width	8012.8

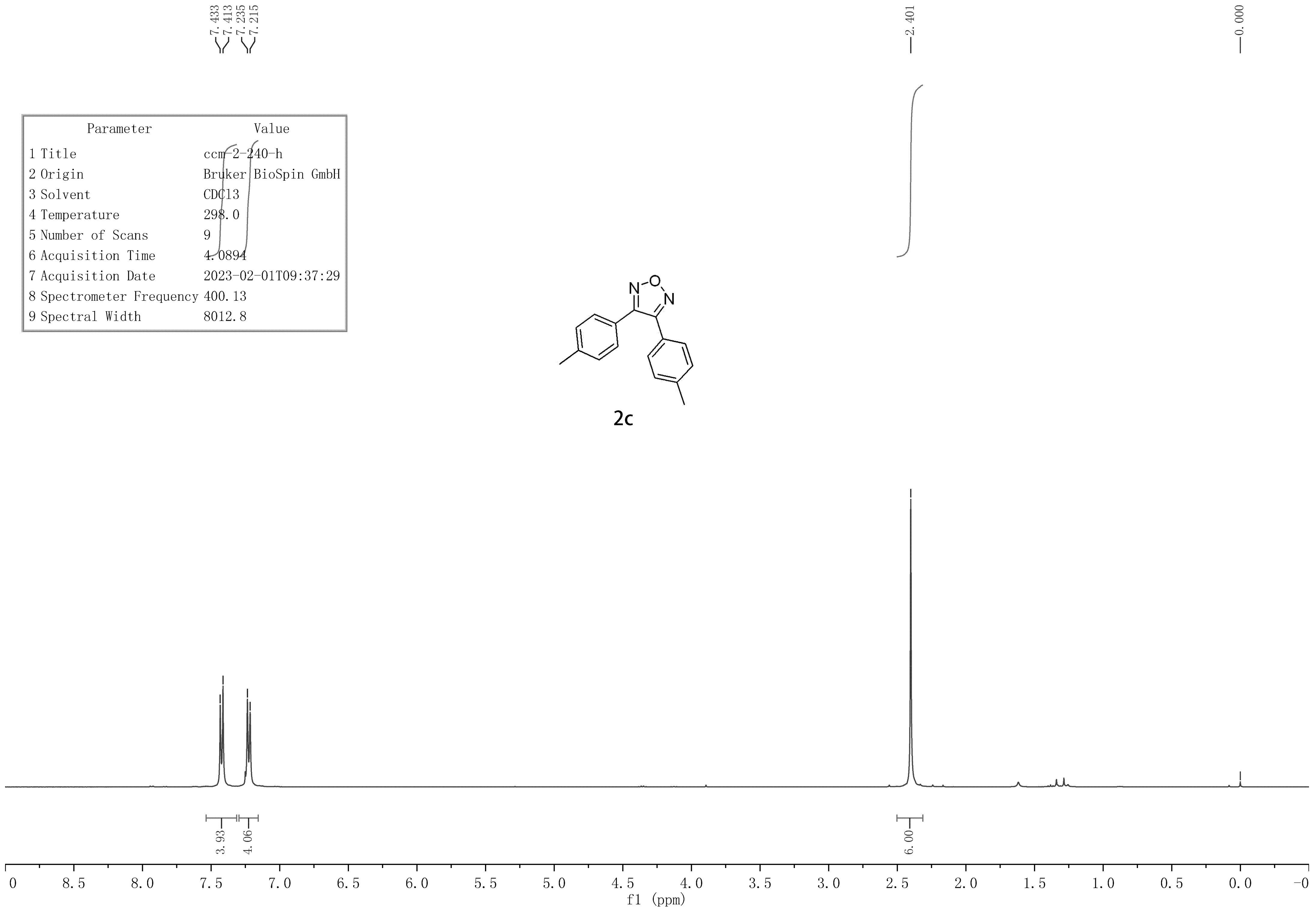

Parameter	Value
1 Title	ccm-2-240-c
2 Origin	Bruker BioSpin GmbH
3 Solvent	CDCl3
4 Temperature	300.0
5 Number of Scans	19
6 Acquisition Time	1.3631
7 Acquisition Date	2023-02-01T09:40:40
8 Spectrometer Frequency	100.61
9 Spectral Width	24038.5

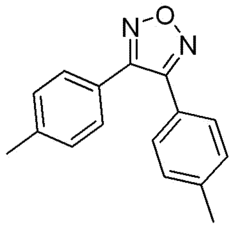

2c

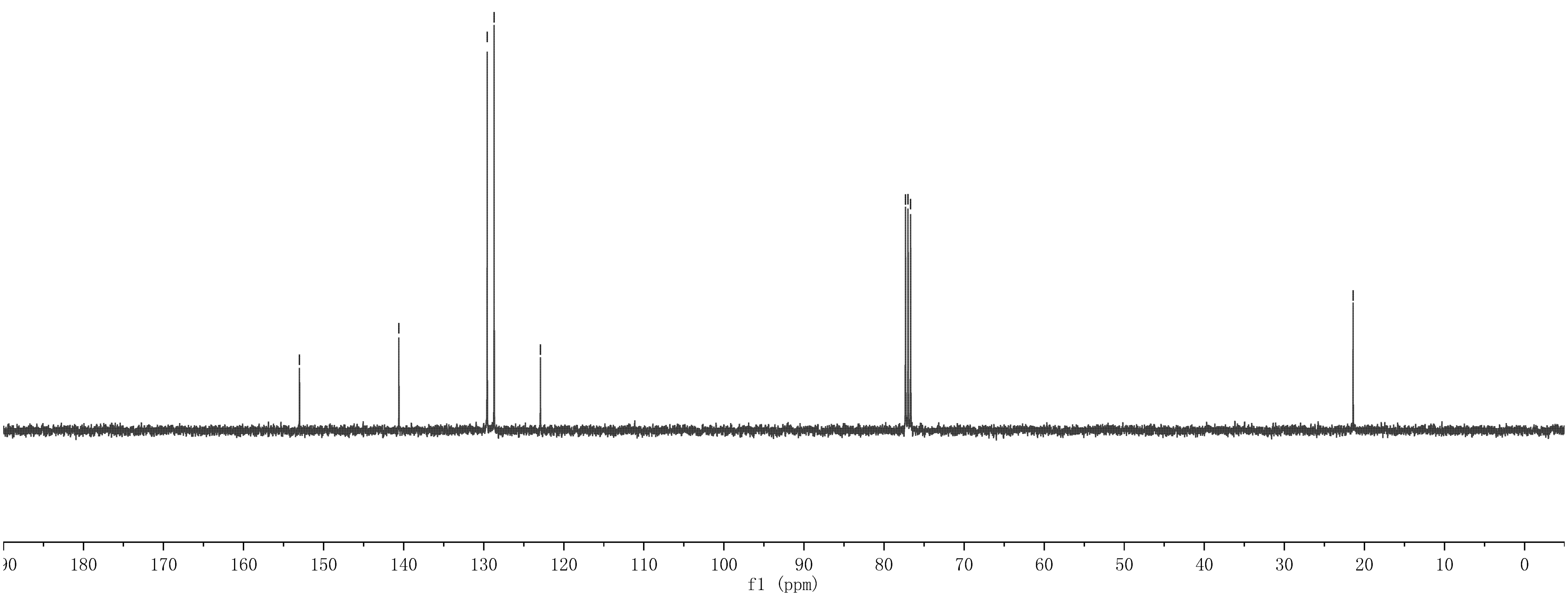

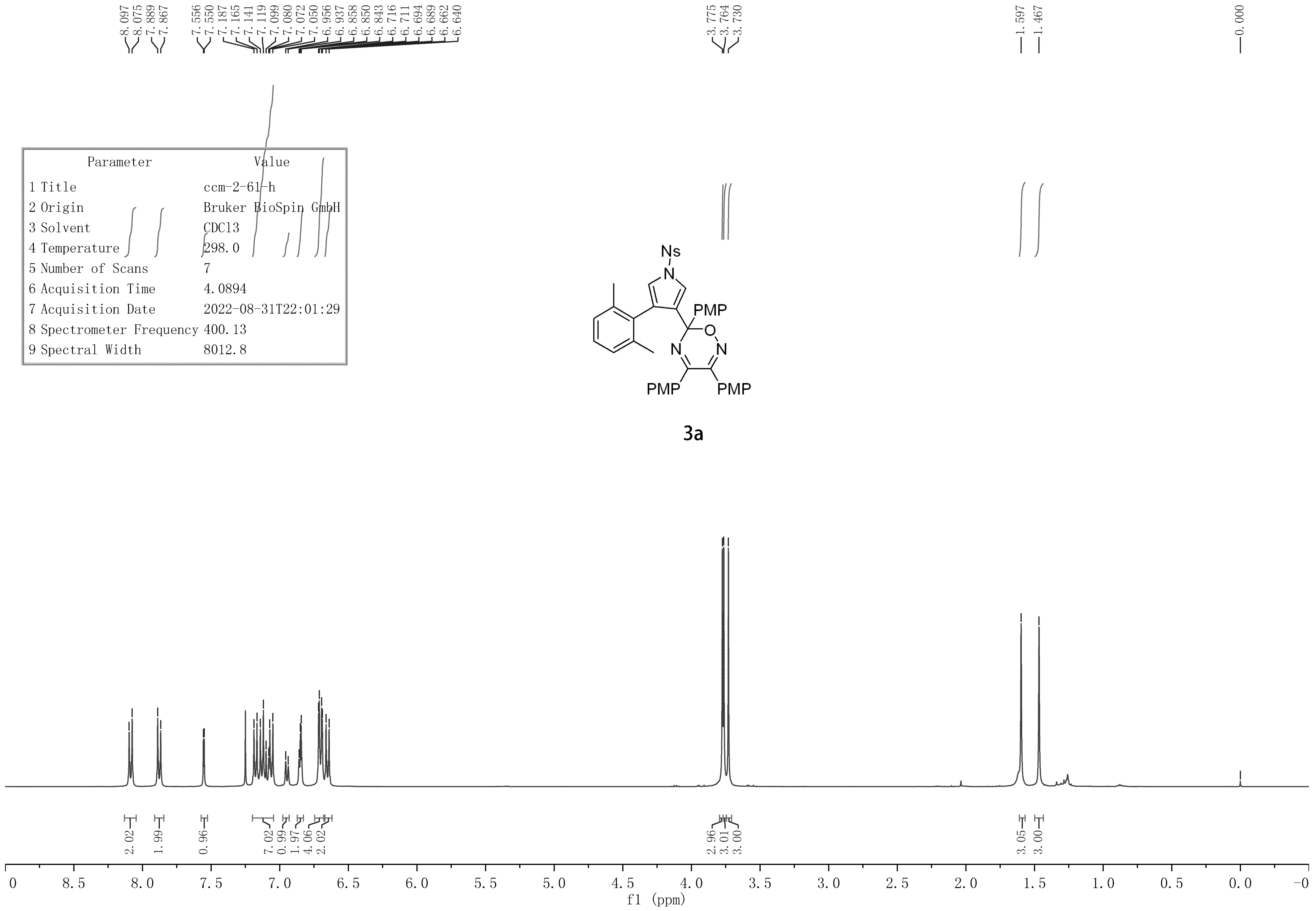
8.097
8.075
7.889
7.867
7.556
7.550
7.187
7.165
7.141
7.119
7.099
7.080
7.072
7.050
6.956
6.937
6.858
6.850
6.843
6.716
6.711
6.694
6.689
6.662
6.640
3.775
3.764
3.730
1.597
1.467
0.000
Parameter Value
1 Title ccm-2-61-h
2 Origin Bruker BioSpin GmbH
3 Solvent CDCl3
4 Temperature 298.0
5 Number of Scans 7
6 Acquisition Time 4.0894
7 Acquisition Date 2022-08-31T22:01:29
8 Spectrometer Frequency 400.13
9 Spectral Width 8012.8
Ns
N
PMP
O
N
N
PMP
PMP
3a
2.02
1.99
0.96
7.02
0.99
1.97
4.06
2.02
2.96
3.01
3.00
3.05
3.00
0 8.5 8.0 7.5 7.0 6.5 6.0 5.5 5.0 4.5 4.0 3.5 3.0 2.5 2.0 1.5 1.0 0.5 0.0 -0
f1 (ppm)

Parameter	Value
1 Title	ccm-2-61-c
2 Origin	Bruker BioSpin GmbH
3 Solvent	CDCl3
4 Temperature	300.0
5 Number of Scans	36
6 Acquisition Time	1.3631
7 Acquisition Date	2022-08-31T22:03:25
8 Spectrometer Frequency	100.61
9 Spectral Width	24038.5

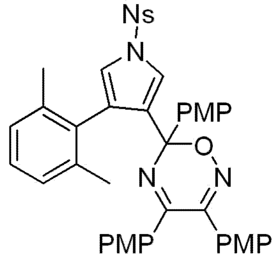

3a

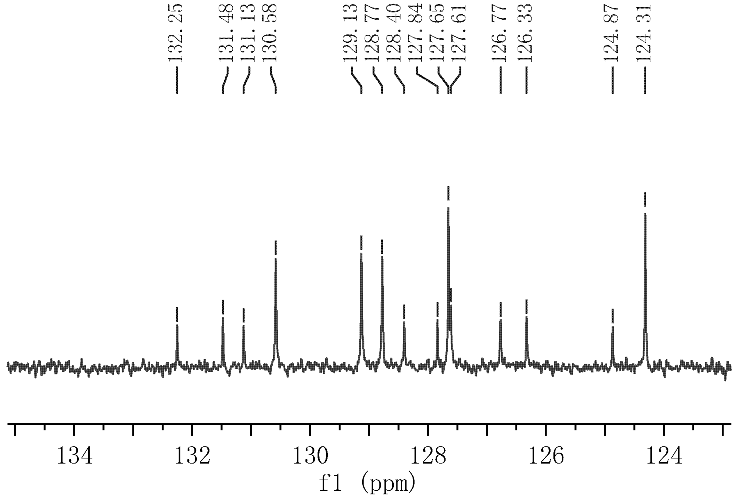

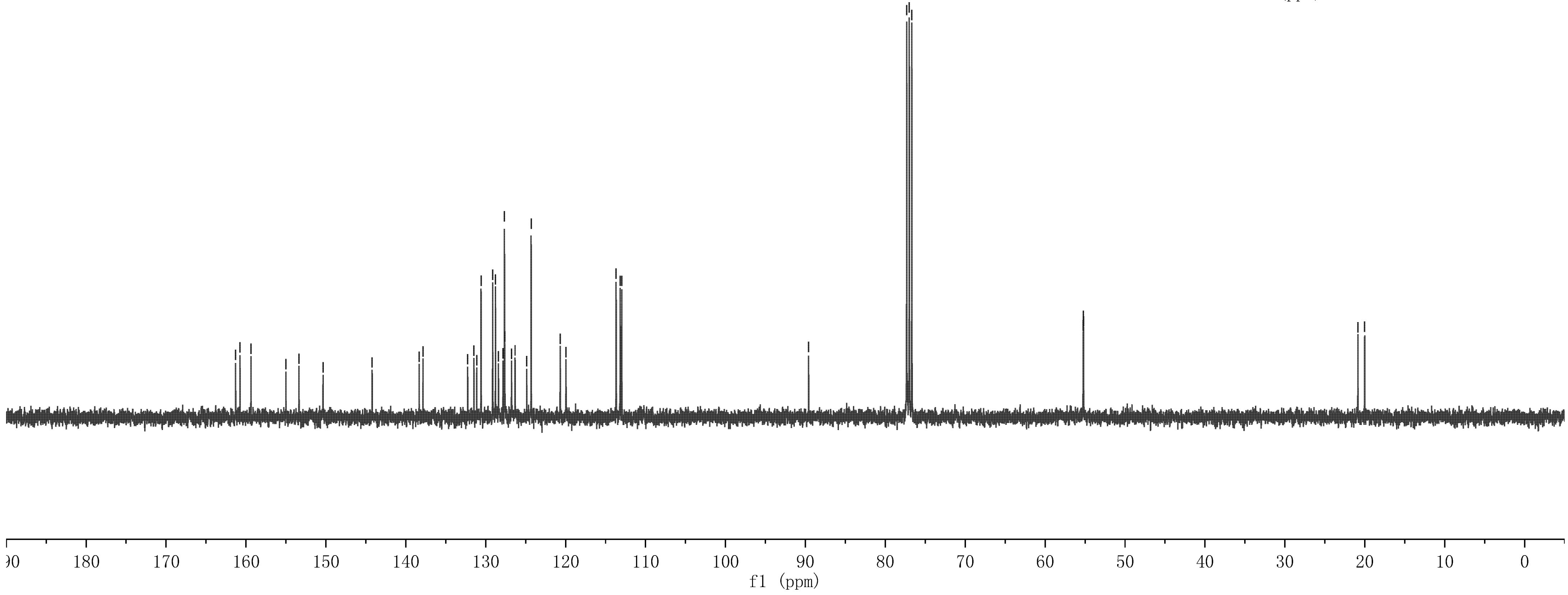

Parameter	Value
1 Title	ccm-2-61-135
2 Origin	Bruker BioSpin GmbH
3 Solvent	CDCl3
4 Temperature	300.0
5 Number of Scans	15
6 Acquisition Time	1.4855
7 Acquisition Date	2022-08-31T22:07:36
8 Spectrometer Frequency	100.61
9 Spectral Width	22058.8

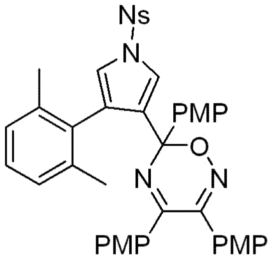

3a

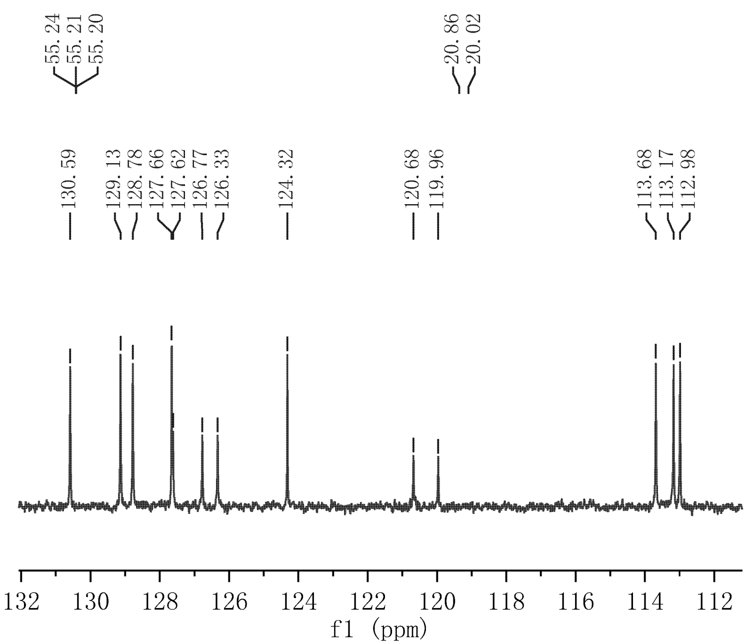

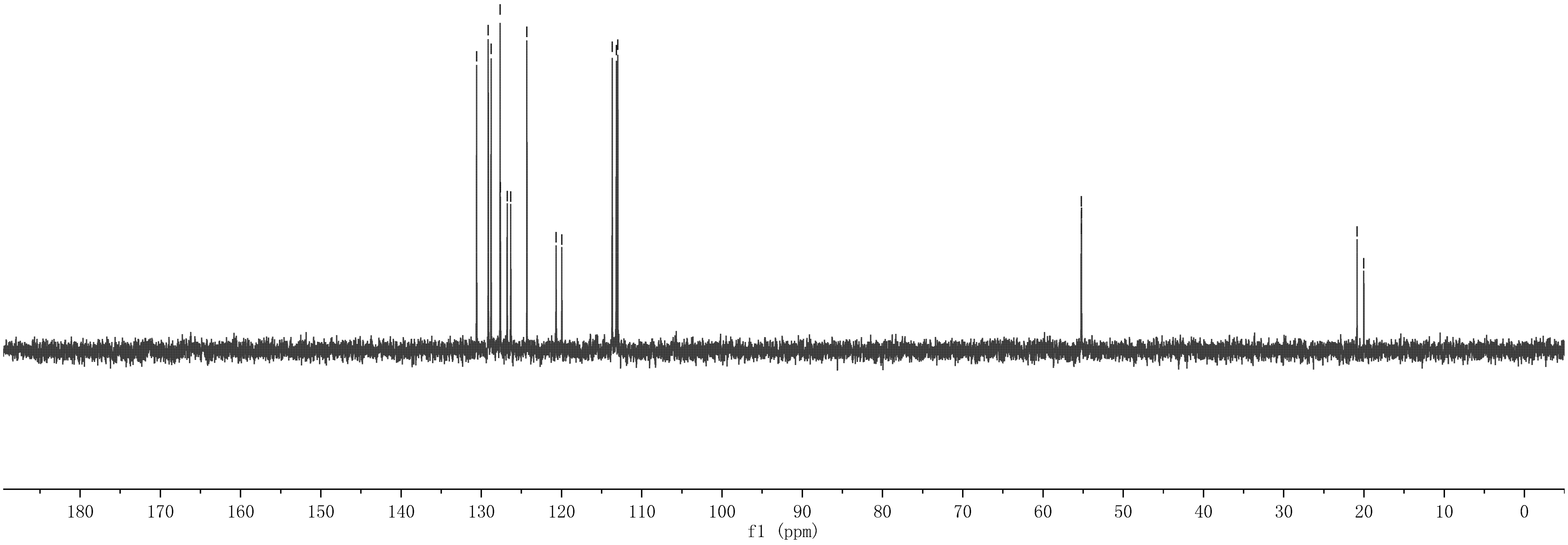

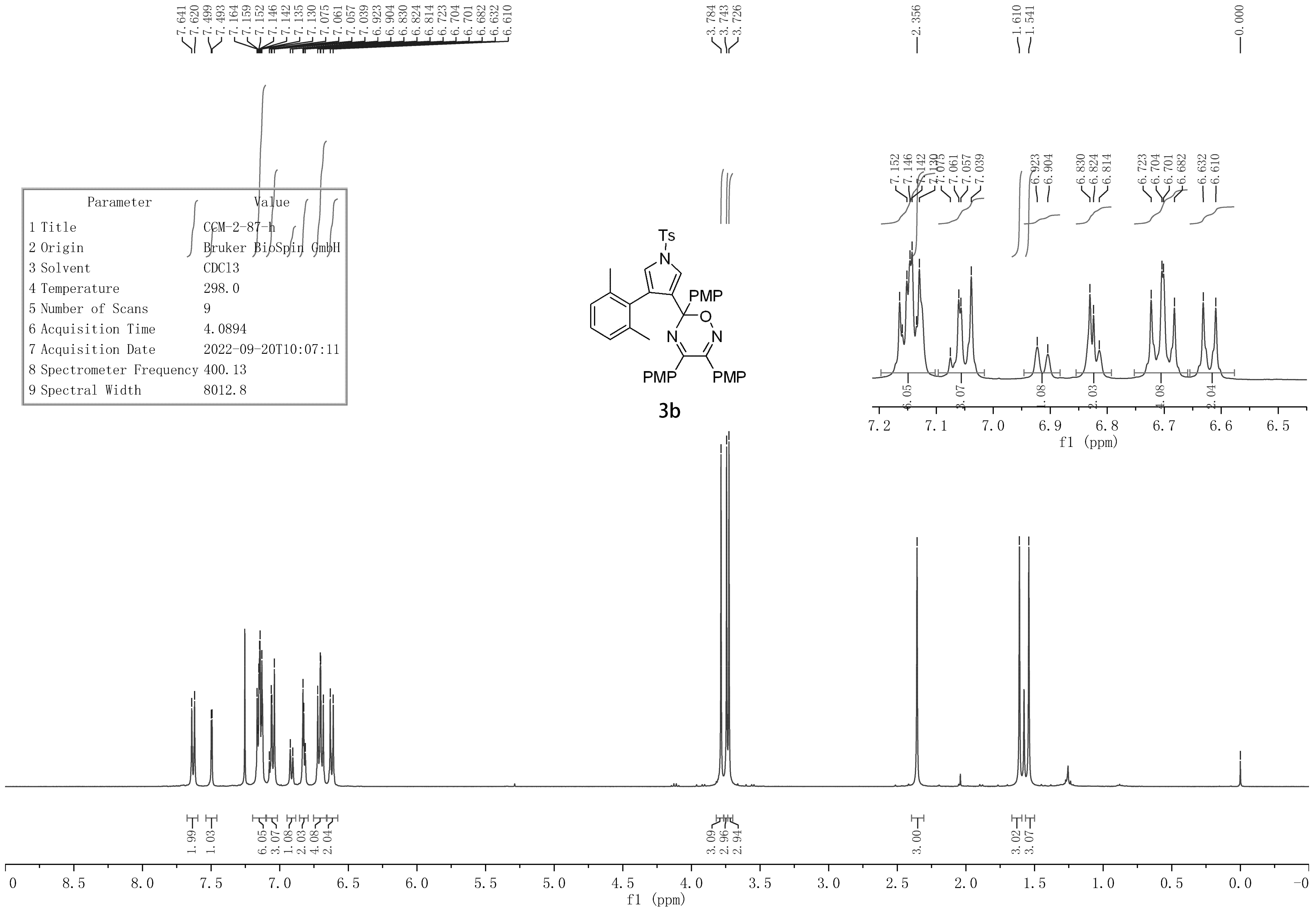

Parameter Value
1 Title CCM-2-87-h
2 Origin Bruker BioSpin GmbH
3 Solvent CDCl3
4 Temperature 298.0
5 Number of Scans 9
6 Acquisition Time 4.0894
7 Acquisition Date 2022-09-20T10:07:11
8 Spectrometer Frequency 400.13
9 Spectral Width 8012.8
Ts
N
PMP
O
N
N
PMP
PMP
3b
7.641 7.620 7.499 7.493 7.164 7.159 7.152 7.146 7.142 7.135 7.130 7.075 7.061 7.057 7.039 6.923 6.904 6.830 6.824 6.814 6.723 6.704 6.701 6.682 6.632 6.610
3.784 3.743 3.726
2.356
1.610 1.541
0.000
1.99 1.03 6.05 3.07 1.08 2.03 4.08 2.04
3.09 2.96 2.94
3.00
3.02 3.07
f1 (ppm)

Parameter	Value
1 Title	CCM-2-87-C
2 Origin	
3 Solvent	CDCl3
4 Temperature	298.5
5 Number of Scans	500
6 Acquisition Time	1.0000
7 Acquisition Date	2022-09-20T14:13:13
8 Spectrometer Frequency	100.56
9 Spectral Width	26041.0

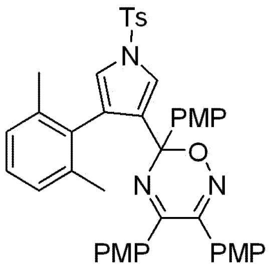

3b

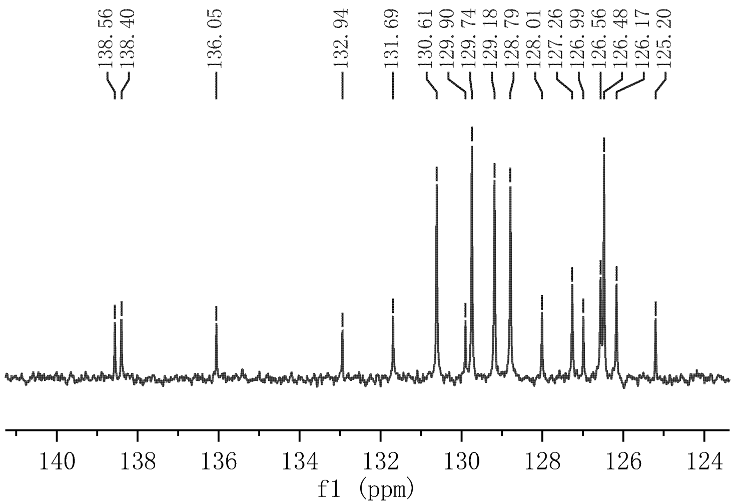

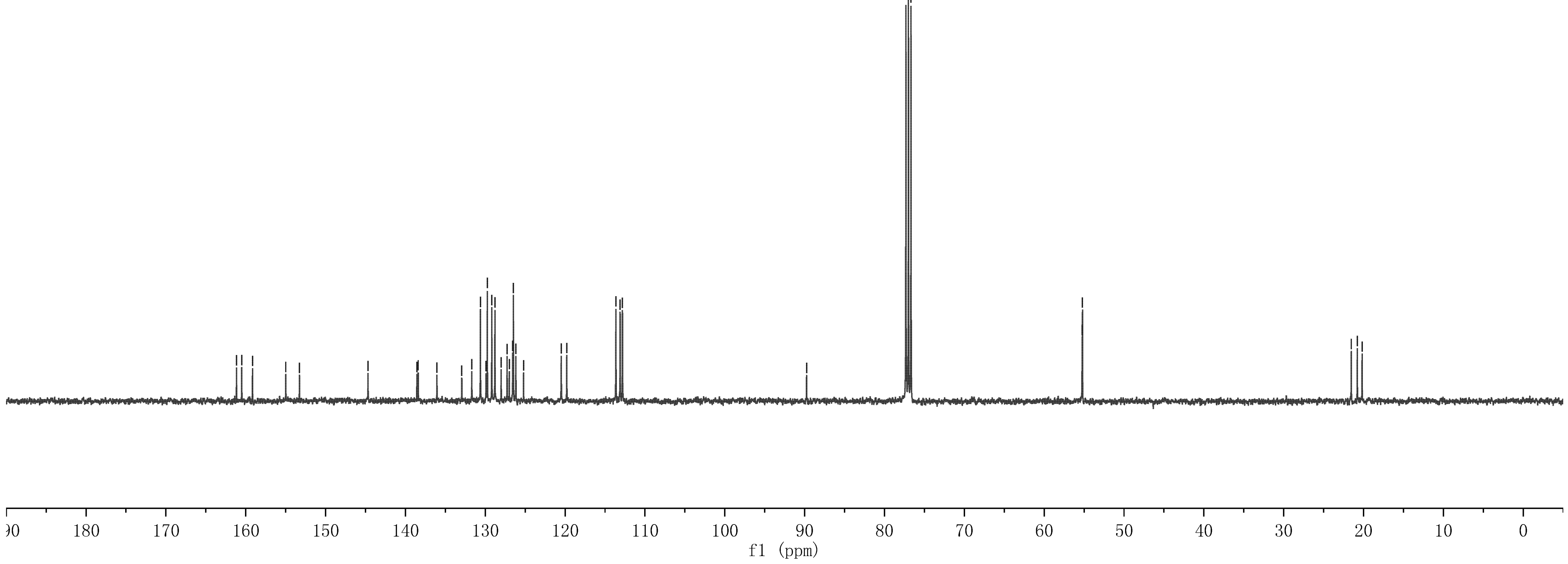

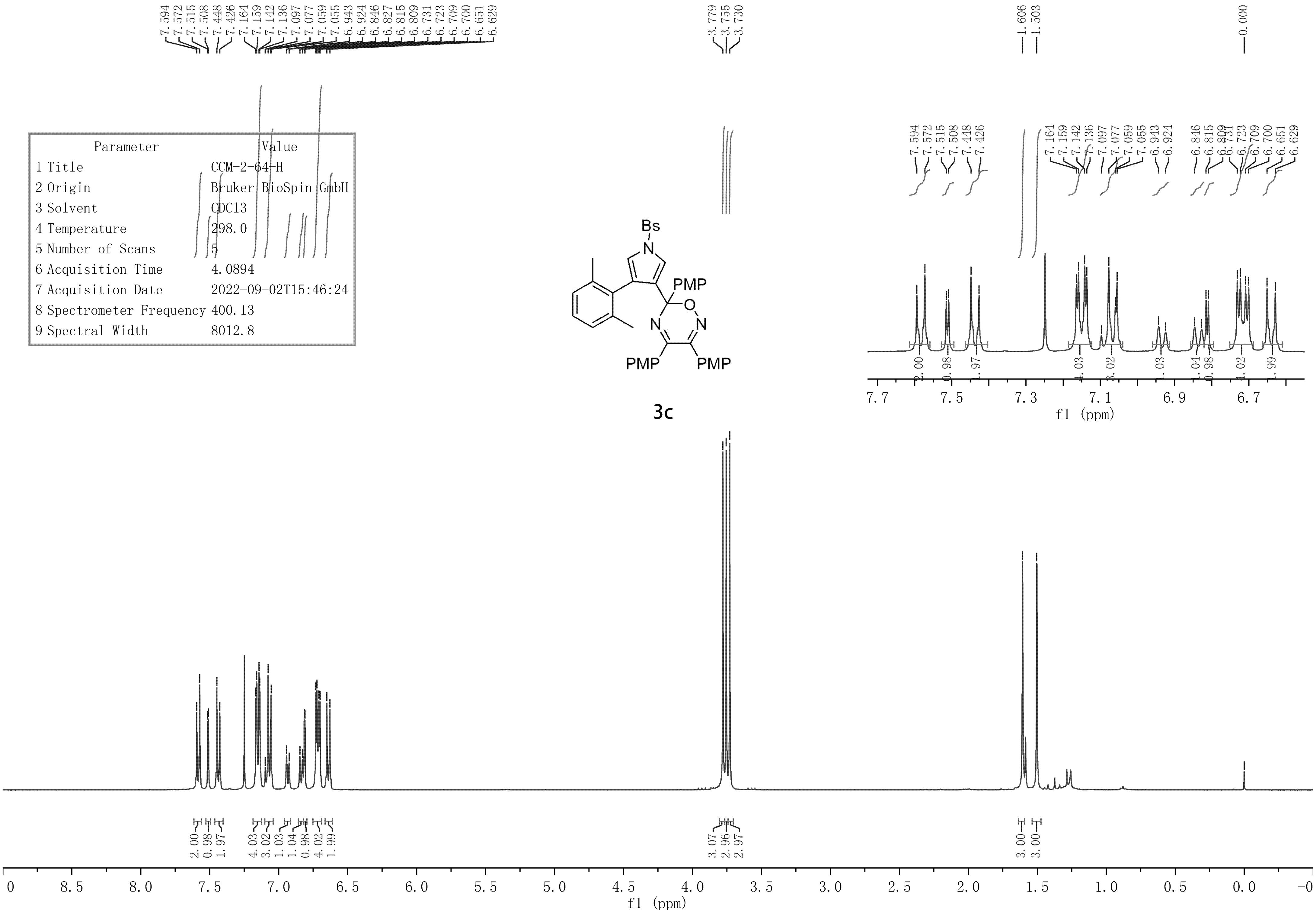

Parameter Value
1 Title CCM-2-64-H
2 Origin Bruker BioSpin GmbH
3 Solvent CDCl3
4 Temperature 298.0
5 Number of Scans 5
6 Acquisition Time 4.0894
7 Acquisition Date 2022-09-02T15:46:24
8 Spectrometer Frequency 400.13
9 Spectral Width 8012.8
Bs
N
PMP
O
N
N
PMP
PMP
3c
7.594 7.572 7.515 7.508 7.448 7.426 7.164 7.159 7.142 7.136 7.097 7.077 7.059 7.055 6.943 6.924 6.846 6.827 6.815 6.809 6.731 6.723 6.709 6.700 6.651 6.629
3.779 3.755 3.730
1.606 1.503
0.000
2.00 0.98 1.97 4.03 3.02 1.03 1.04 0.98 4.02 1.99
3.07 2.96 2.97
3.00 3.00
f1 (ppm)

Parameter	Value
1 Title	CCM-2-64-C
2 Origin	Bruker BioSpin GmbH
3 Solvent	CDCl3
4 Temperature	300.0
5 Number of Scans	89
6 Acquisition Time	1.3631
7 Acquisition Date	2022-09-02T15:48:16
8 Spectrometer Frequency	100.61
9 Spectral Width	24038.5

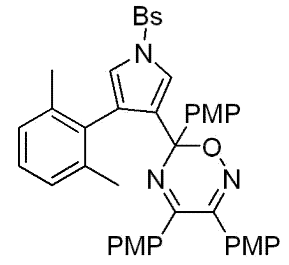

3c

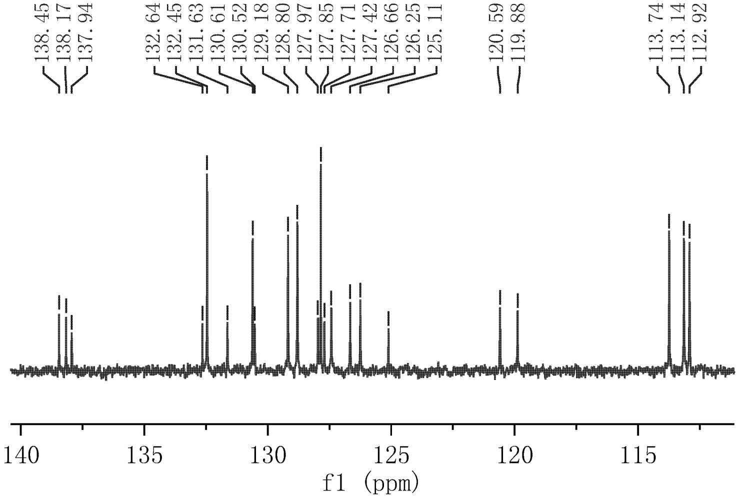

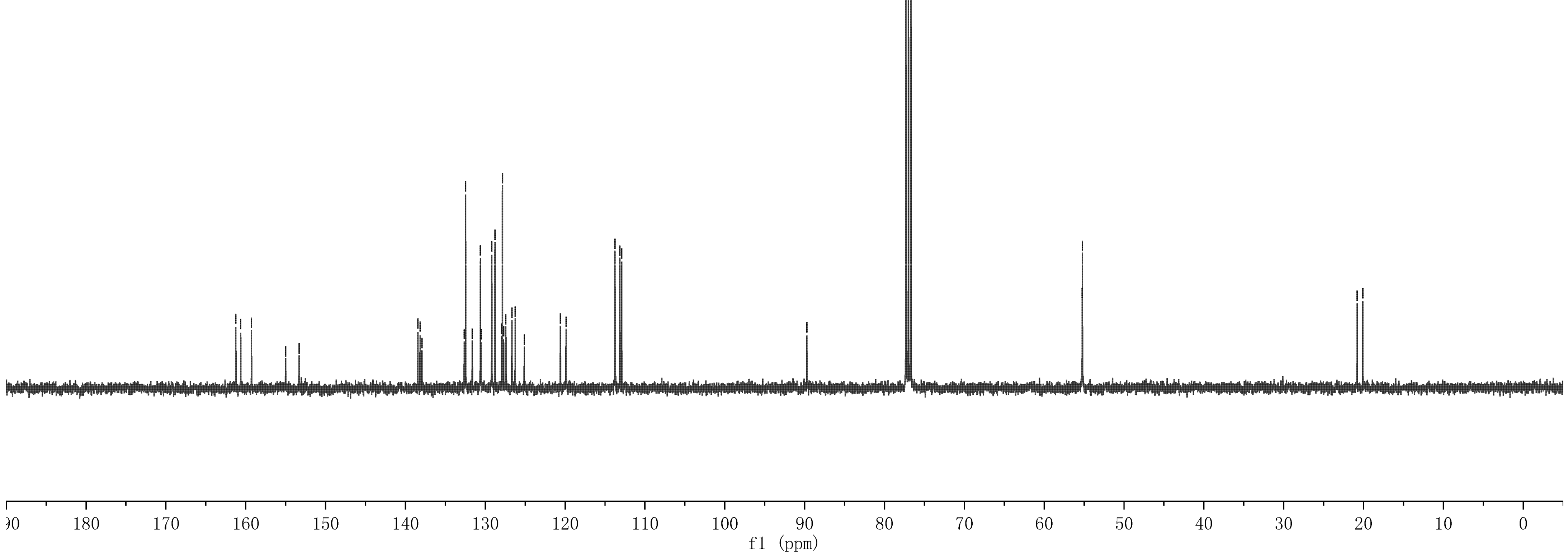

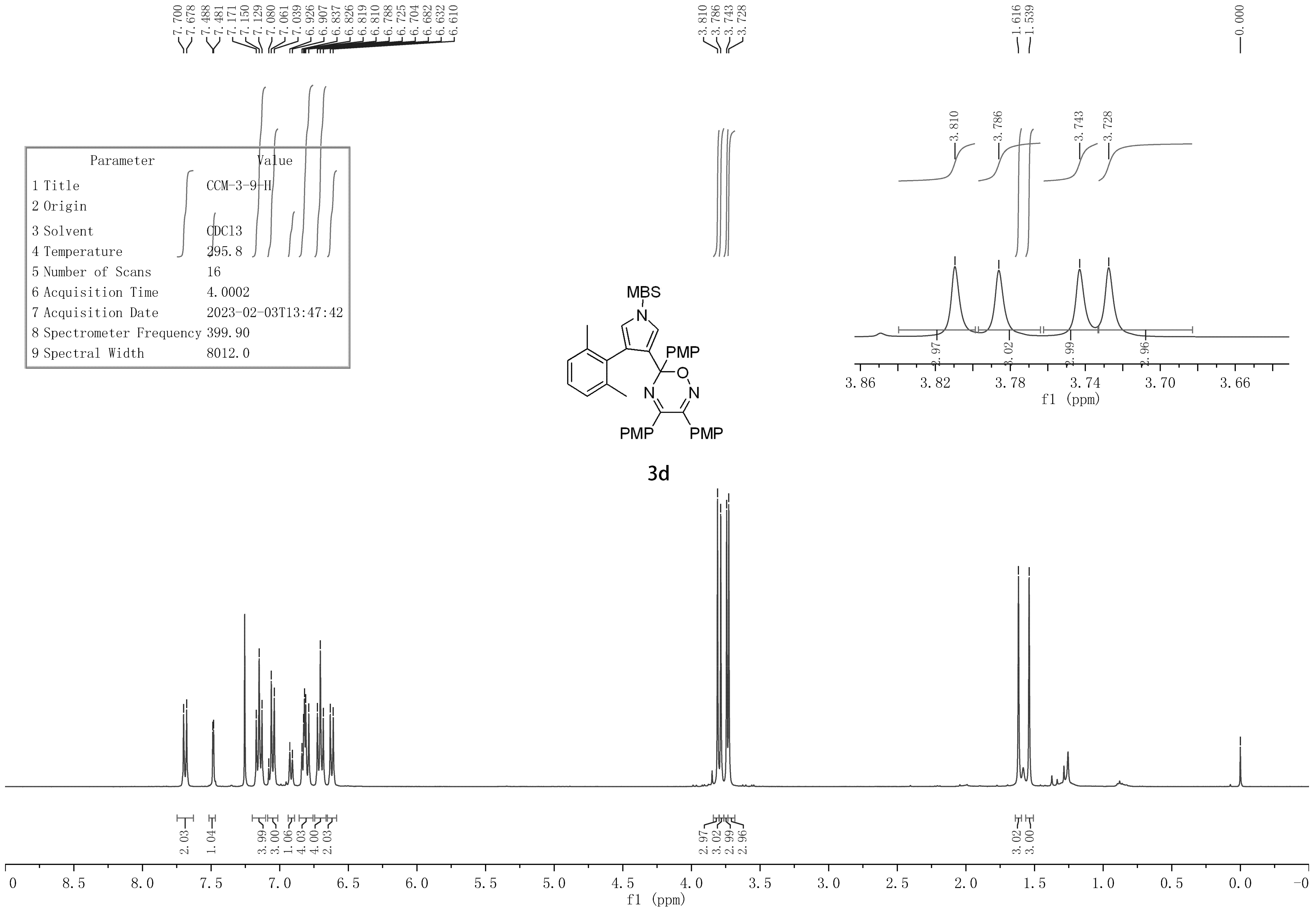

7.700 7.678 7.488 7.481 7.171 7.150 7.129 7.080 7.061 7.039 6.926 6.907 6.837 6.826 6.819 6.810 6.788 6.725 6.704 6.682 6.632 6.610
3.810 3.786 3.743 3.728
1.616 1.539
0.000
Parameter | Value
1 Title | CCM-3-9-H
2 Origin |
3 Solvent | CDCl3
4 Temperature | 295.8
5 Number of Scans | 16
6 Acquisition Time | 4.0002
7 Acquisition Date | 2023-02-03T13:47:42
8 Spectrometer Frequency | 399.90
9 Spectral Width | 8012.0
MBS
N
PMP
O
N
N
PMP
PMP
3d
3.810 3.786 3.743 3.728
2.97 3.02 2.99 2.96
3.86 3.82 3.78 3.74 3.70 3.66
f1 (ppm)
2.03 1.04 3.99 3.00 1.06 4.03 4.00 2.03
2.97 3.02 2.99 2.96
3.02 3.00
0 8.5 8.0 7.5 7.0 6.5 6.0 5.5 5.0 4.5 4.0 3.5 3.0 2.5 2.0 1.5 1.0 0.5 0.0 -0
f1 (ppm)

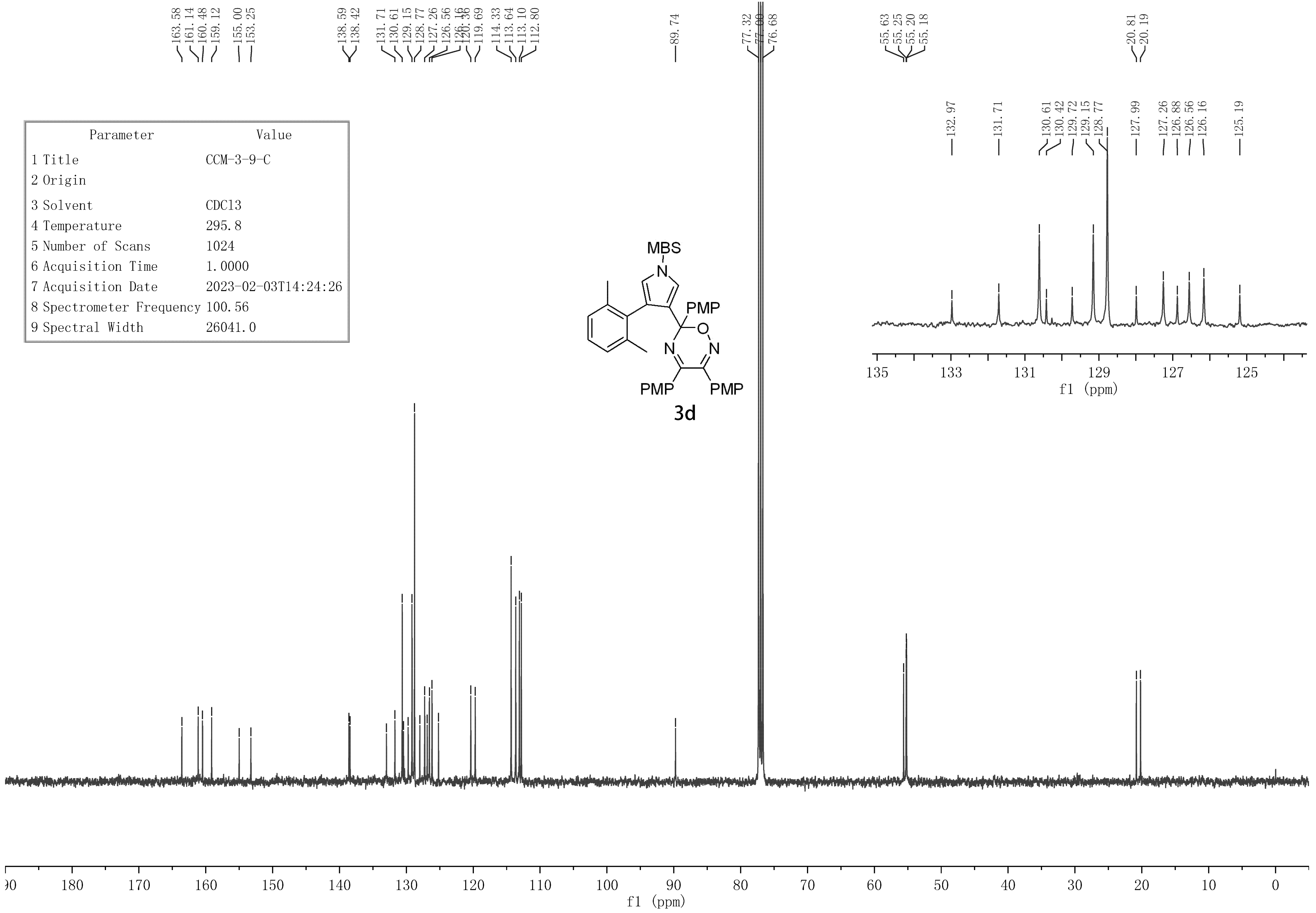

Parameter	Value
1 Title	CCM-3-9-C
2 Origin	
3 Solvent	CDCl3
4 Temperature	295.8
5 Number of Scans	1024
6 Acquisition Time	1.0000
7 Acquisition Date	2023-02-03T14:24:26
8 Spectrometer Frequency	100.56
9 Spectral Width	26041.0

3d

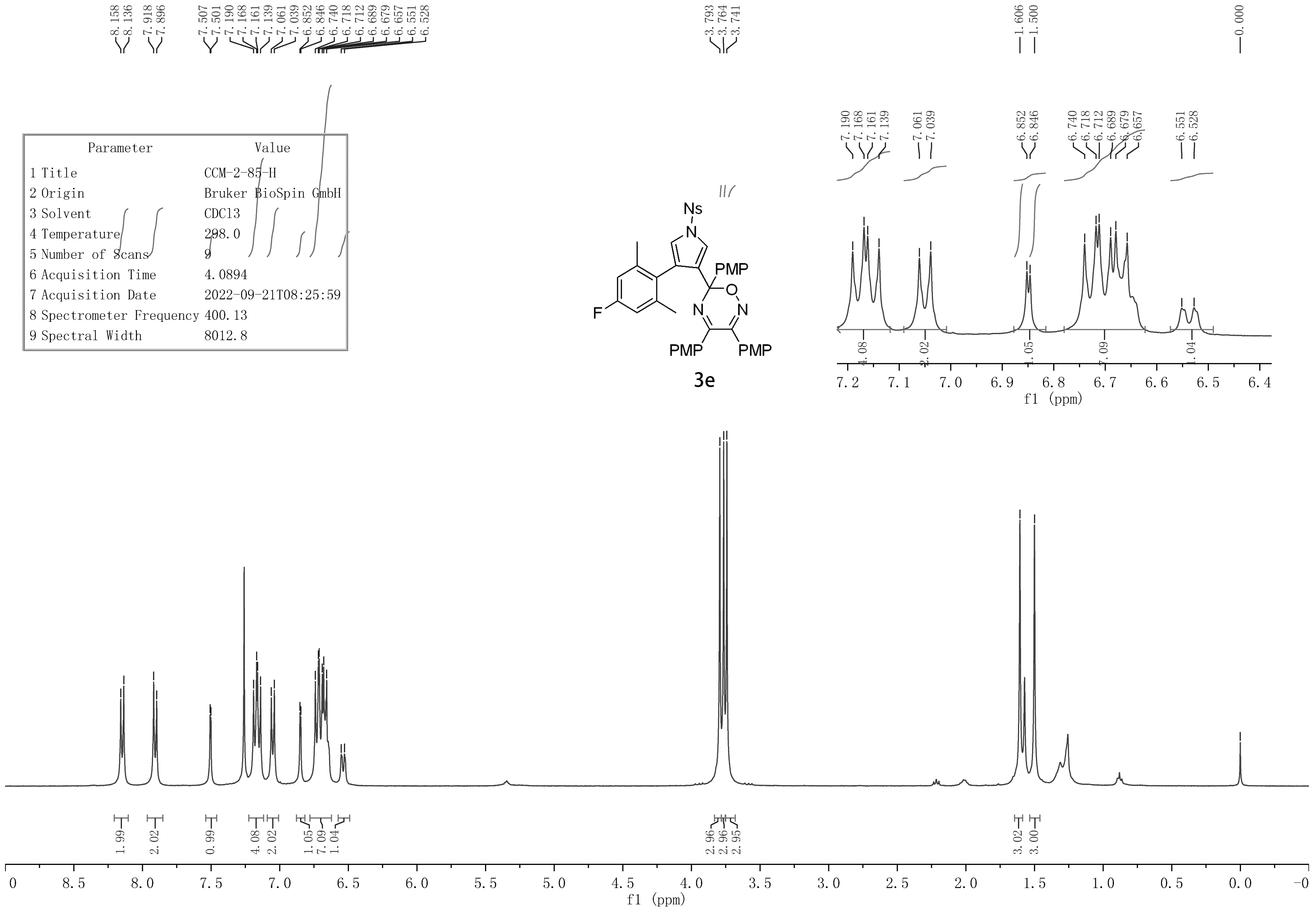

8.158
8.136
7.918
7.896
7.507
7.501
7.190
7.168
7.161
7.139
7.061
7.039
6.852
6.846
6.740
6.718
6.712
6.689
6.679
6.657
6.551
6.528
3.793
3.764
3.741
1.606
1.500
0.000
Parameter Value
1 Title CCM-2-85-H
2 Origin Bruker BioSpin GmbH
3 Solvent CDCl3
4 Temperature 298.0
5 Number of Scans 9
6 Acquisition Time 4.0894
7 Acquisition Date 2022-09-21T08:25:59
8 Spectrometer Frequency 400.13
9 Spectral Width 8012.8
Ns
N
F
PMP
O
N
N
PMP
PMP
3e
4.08
2.02
1.05
7.09
1.04
7.2 7.1 7.0 6.9 6.8 6.7 6.6 6.5 6.4
f1 (ppm)
1.99
2.02
0.99
4.08
2.02
1.05
7.09
1.04
2.96
2.96
2.95
3.02
3.00
8.5 8.0 7.5 7.0 6.5 6.0 5.5 5.0 4.5 4.0 3.5 3.0 2.5 2.0 1.5 1.0 0.5 0.0
f1 (ppm)

Parameter	Value
1 Title	ccm-2-85-c
2 Origin	
3 Solvent	CDCl3
4 Temperature	298.3
5 Number of Scans	2000
6 Acquisition Time	1.0000
7 Acquisition Date	2022-09-22T01:39:41
8 Spectrometer Frequency	100.56
9 Spectral Width	26041.0

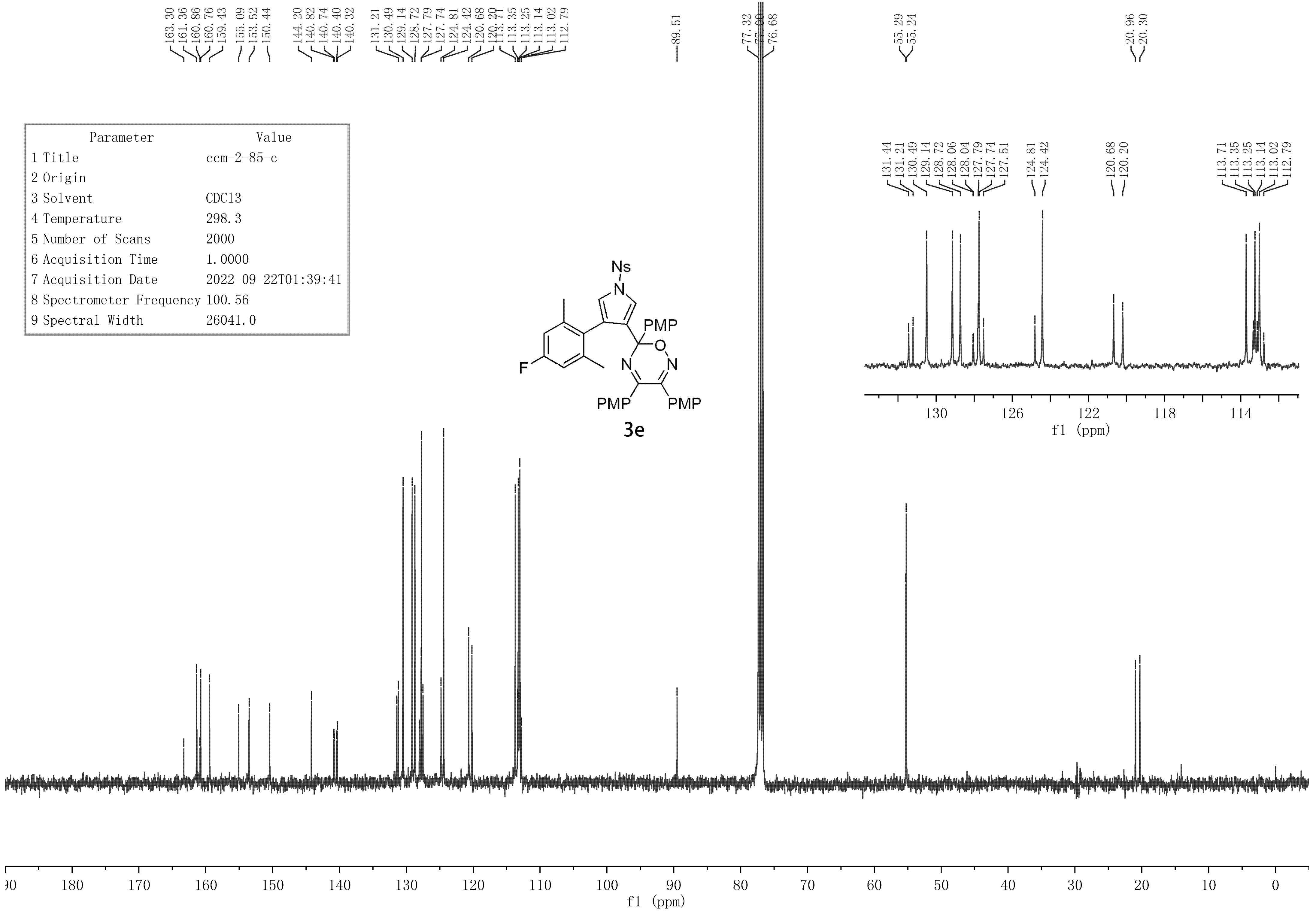

Parameter	Value
1 Title	ccm-2-85-f
2 Origin	Bruker BioSpin GmbH
3 Solvent	C6D6
4 Temperature	297.6
5 Number of Scans	16
6 Acquisition Time	0.7340
7 Acquisition Date	2022-09-20T20:28:01
8 Spectrometer Frequency	376.31
9 Spectral Width	89285.7

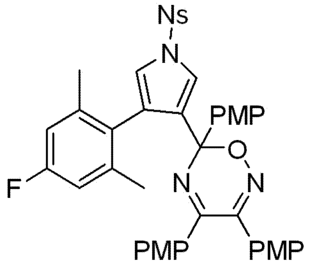

3e

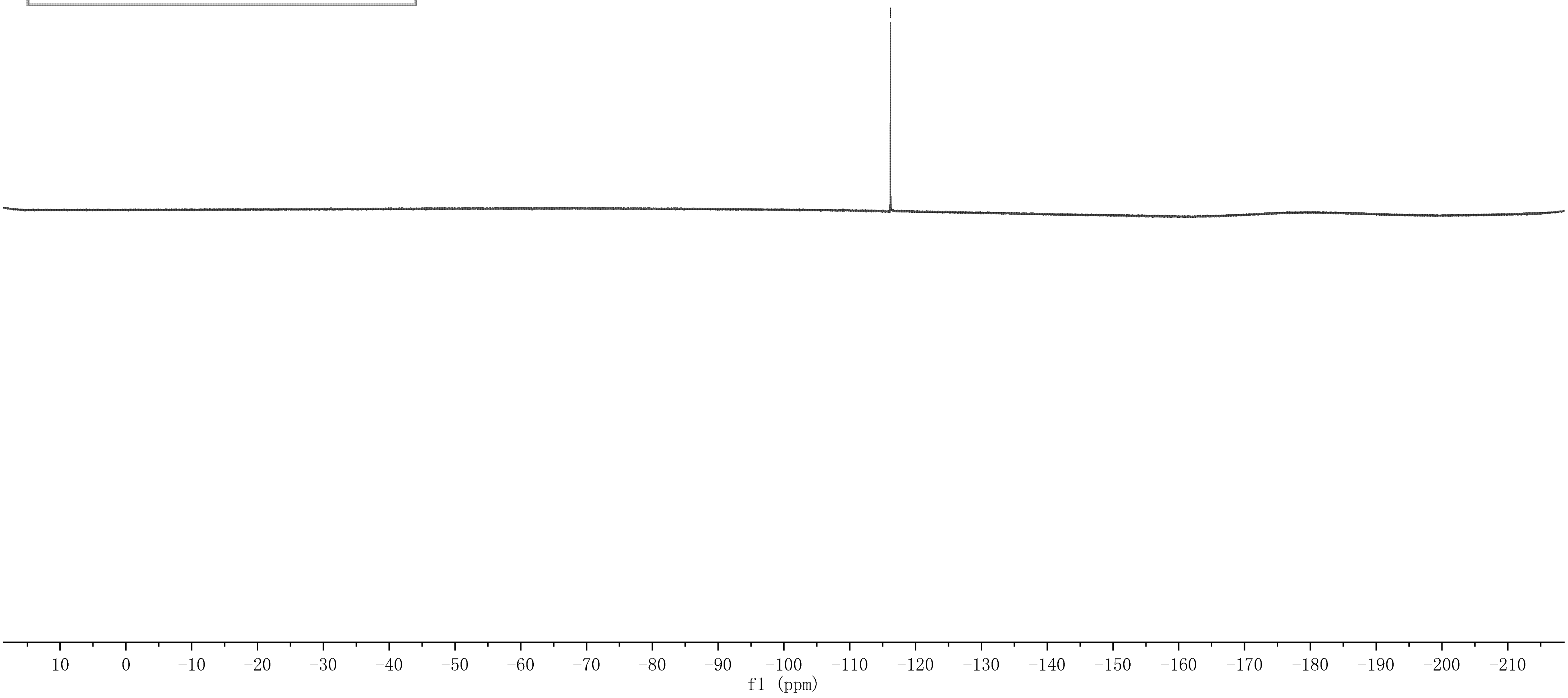

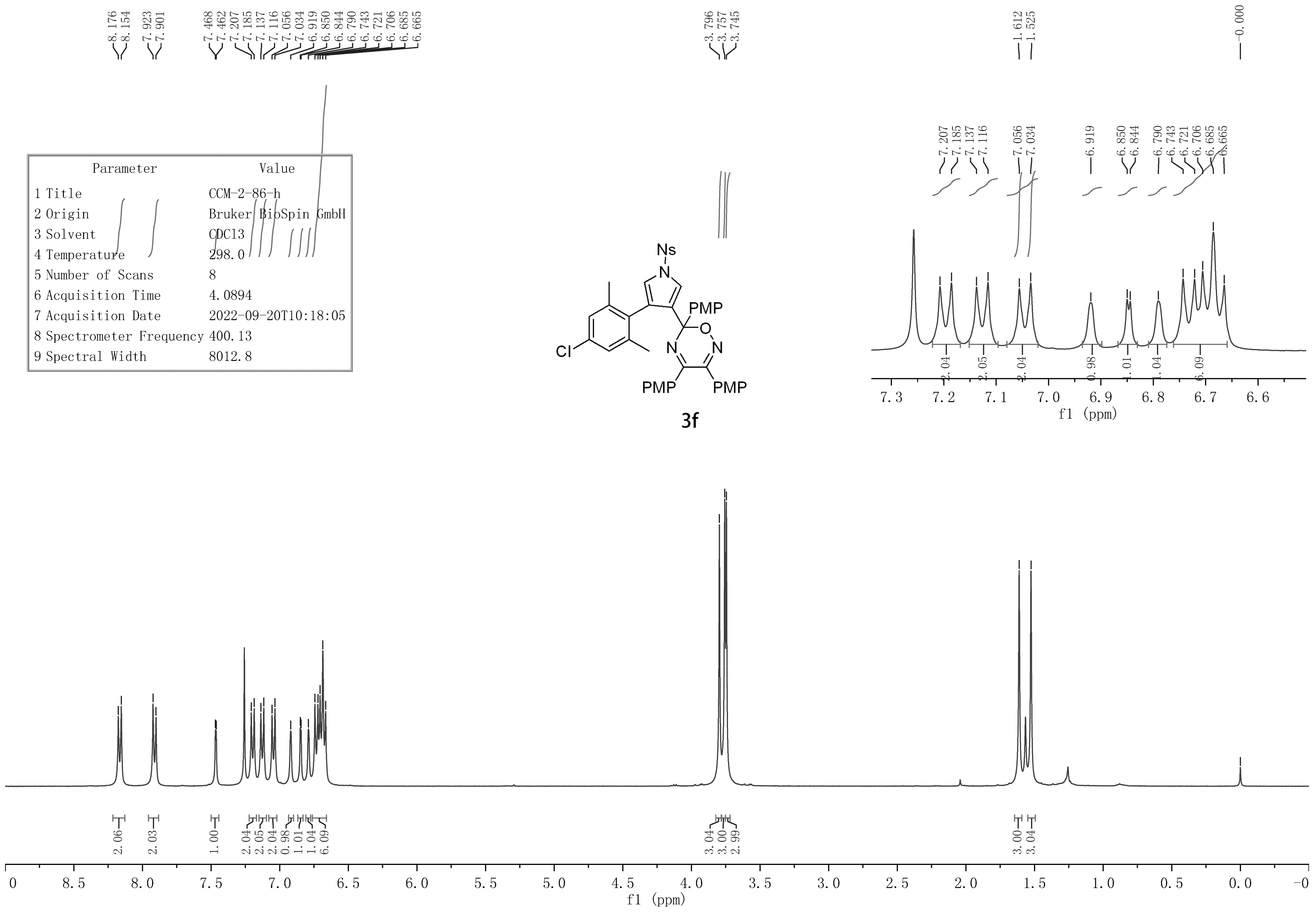
8.176
8.154
7.923
7.901
7.468
7.462
7.207
7.185
7.137
7.116
7.056
7.034
6.919
6.850
6.844
6.790
6.743
6.721
6.706
6.685
6.665
3.796
3.757
3.745
1.612
1.525
-0.000
Parameter
Value
1 Title
CCM-2-86-h
2 Origin
Bruker BioSpin GmbH
3 Solvent
CDCl3
4 Temperature
298.0
5 Number of Scans
8
6 Acquisition Time
4.0894
7 Acquisition Date
2022-09-20T10:18:05
8 Spectrometer Frequency
400.13
9 Spectral Width
8012.8
Ns
N
PMP
O
N
N
Cl
PMP
PMP
2.06
2.03
1.00
2.04
2.05
2.04
0.98
1.01
1.04
6.09
3.04
3.00
2.99
3.00
3.04
f1 (ppm)

3f

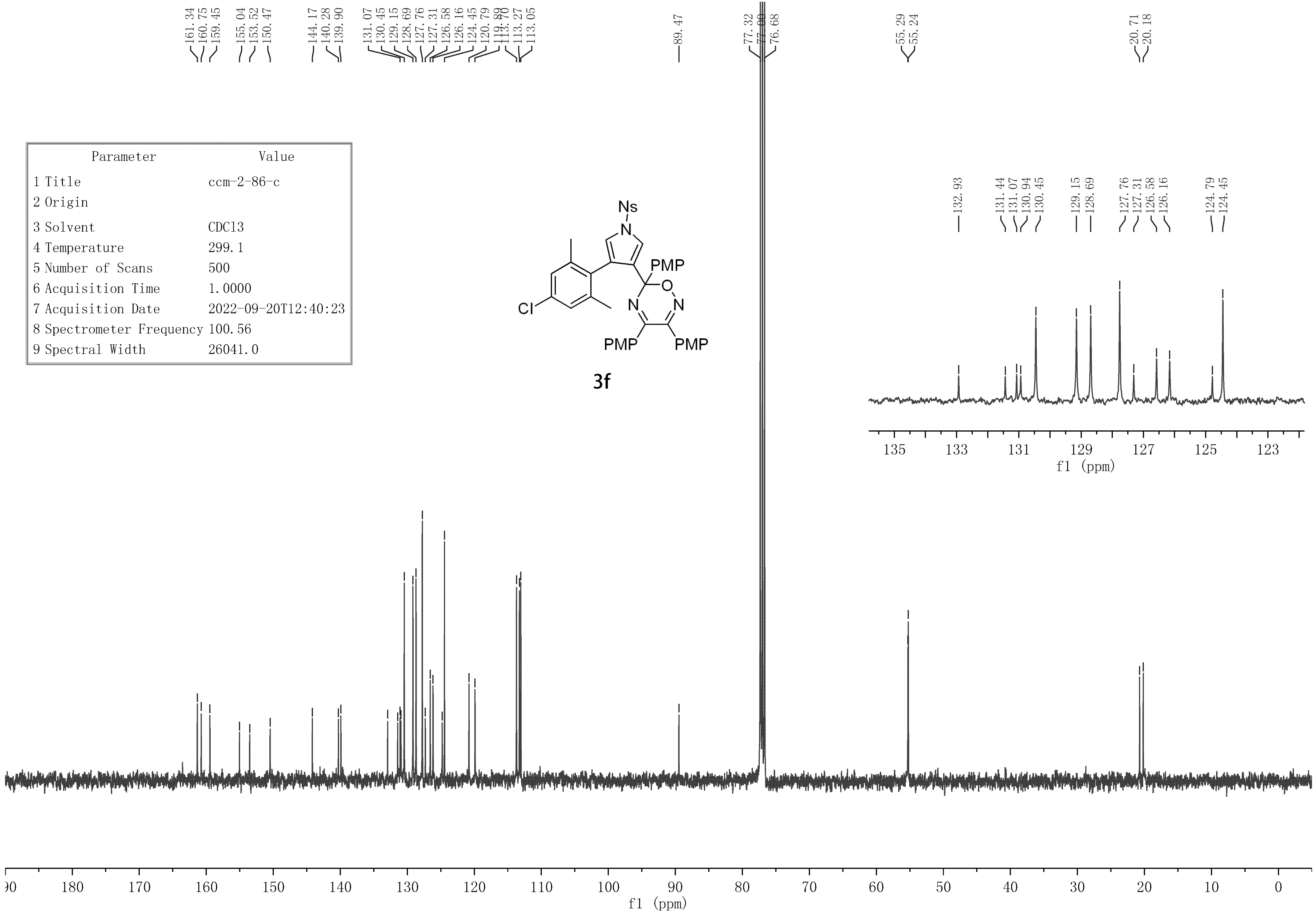

Parameter	Value
1 Title	ccm-2-86-c
2 Origin	
3 Solvent	CDCl3
4 Temperature	299.1
5 Number of Scans	500
6 Acquisition Time	1.0000
7 Acquisition Date	2022-09-20T12:40:23
8 Spectrometer Frequency	100.56
9 Spectral Width	26041.0

3f

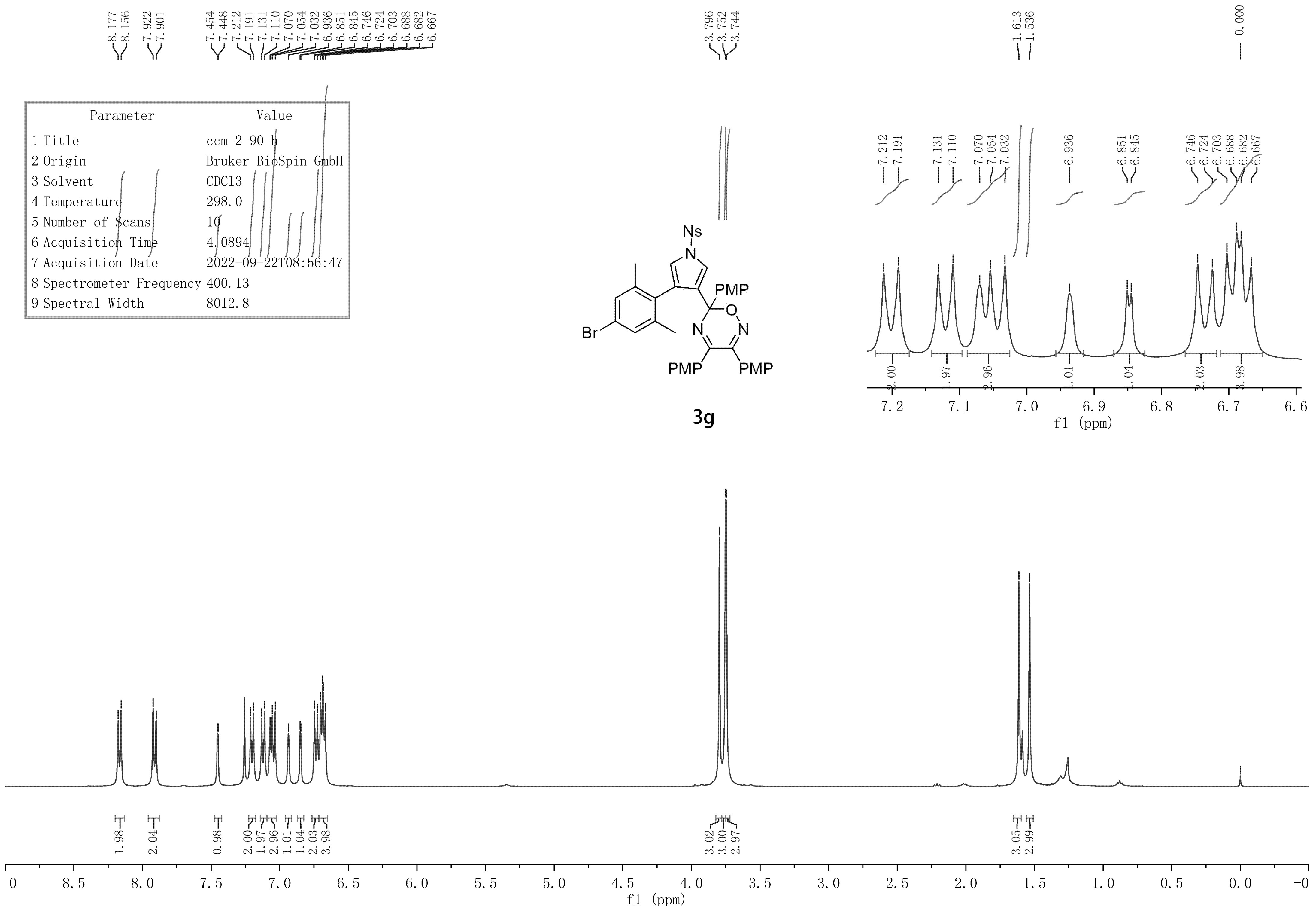

8.177
8.156
7.922
7.901
7.454
7.448
7.212
7.191
7.131
7.110
7.070
7.054
7.032
6.936
6.851
6.845
6.746
6.724
6.703
6.688
6.682
6.667
3.796
3.752
3.744
1.613
1.536
-0.000
Parameter
Value
1 Title
ccm-2-90-h
2 Origin
Bruker BioSpin GmbH
3 Solvent
CDCl3
4 Temperature
298.0
5 Number of Scans
10
6 Acquisition Time
4.0894
7 Acquisition Date
2022-09-22T08:56:47
8 Spectrometer Frequency
400.13
9 Spectral Width
8012.8
Ns
N
PMP
O
N
N
Br
PMP
PMP
3g
2.00
1.97
2.96
1.01
1.04
2.03
3.98
7.2
7.1
7.0
6.9
6.8
6.7
6.6
f1 (ppm)
1.98
2.04
0.98
3.02
3.00
2.97
3.05
2.99
8.5
8.0
7.5
7.0
6.5
6.0
5.5
5.0
4.5
4.0
3.5
3.0
2.5
2.0
1.5
1.0
0.5
0.0
f1 (ppm)

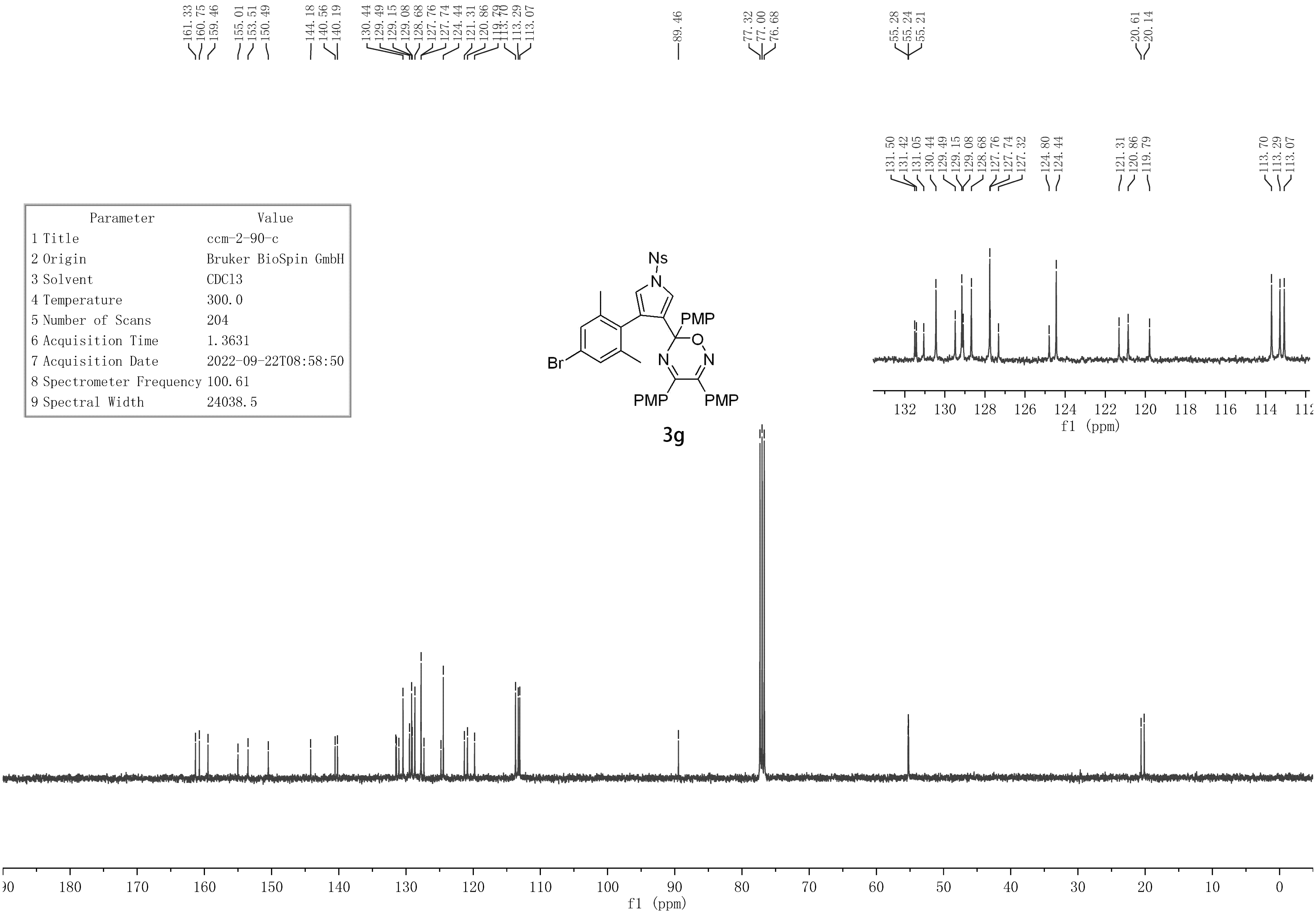

3g

Parameter	Value
1 Title	ccm-2-90-c
2 Origin	Bruker BioSpin GmbH
3 Solvent	CDCl3
4 Temperature	300.0
5 Number of Scans	204
6 Acquisition Time	1.3631
7 Acquisition Date	2022-09-22T08:58:50
8 Spectrometer Frequency	100.61
9 Spectral Width	24038.5

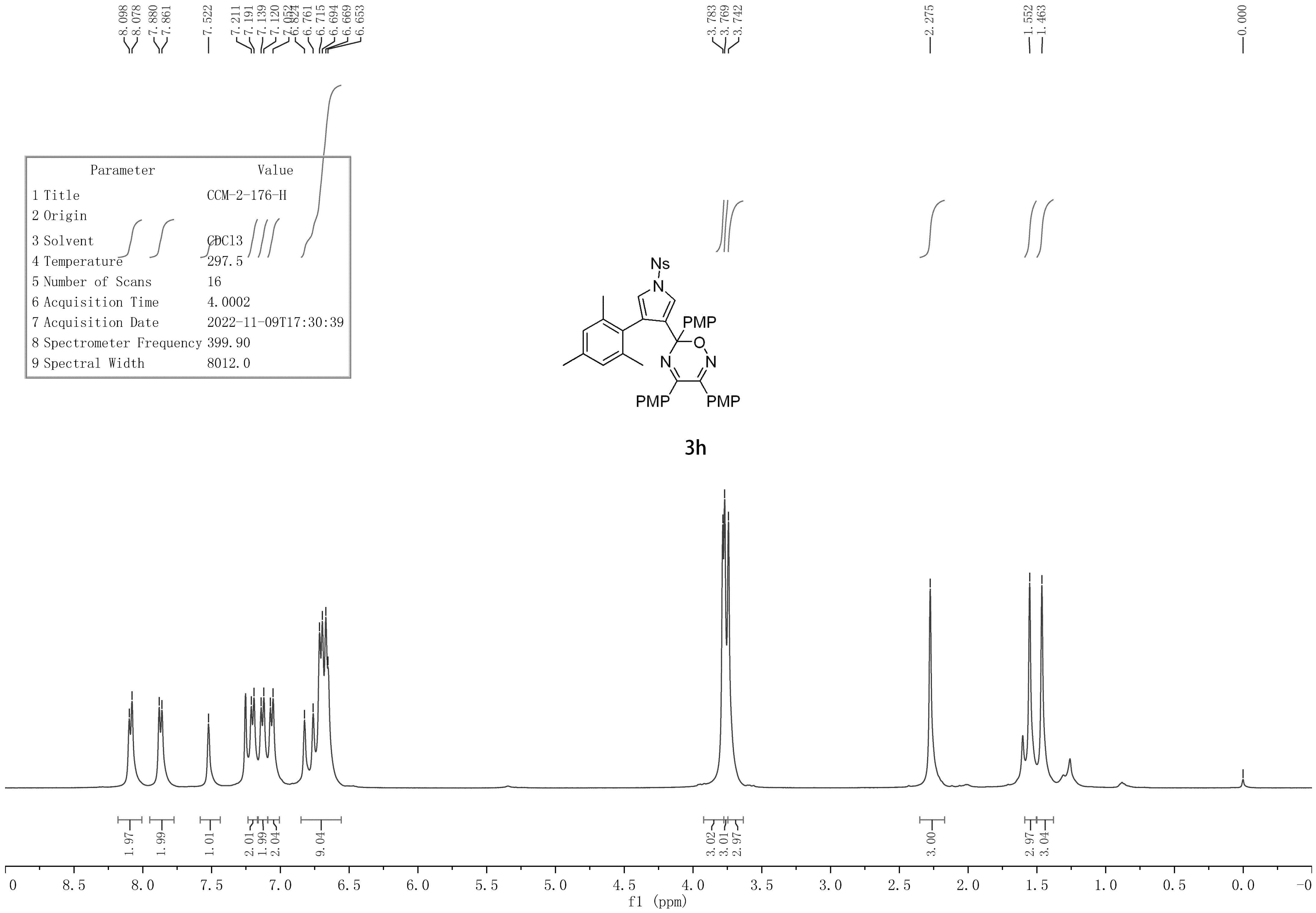
8.098
8.078
7.880
7.861
7.522
7.211
7.191
7.139
7.120
7.052
6.824
6.761
6.715
6.694
6.669
6.653
3.783
3.769
3.742
2.275
1.552
1.463
0.000
Parameter Value
1 Title CCM-2-176-H
2 Origin
3 Solvent CDCl3
4 Temperature 297.5
5 Number of Scans 16
6 Acquisition Time 4.0002
7 Acquisition Date 2022-11-09T17:30:39
8 Spectrometer Frequency 399.90
9 Spectral Width 8012.0
Ns
N
PMP
O
N
N
PMP
PMP
3h
1.97
1.99
1.01
2.01
1.99
2.04
9.04
3.02
3.01
2.97
3.00
2.97
3.04
f1 (ppm)

Parameter	Value
1 Title	CCM-2-176-C
2 Origin	
3 Solvent	CDCl3
4 Temperature	298.2
5 Number of Scans	1024
6 Acquisition Time	1.0000
7 Acquisition Date	2022-11-09T18:07:25
8 Spectrometer Frequency	100.56
9 Spectral Width	26041.0

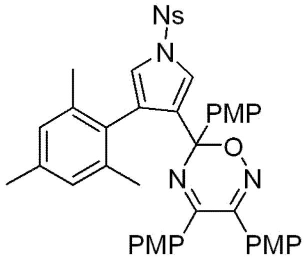

3h

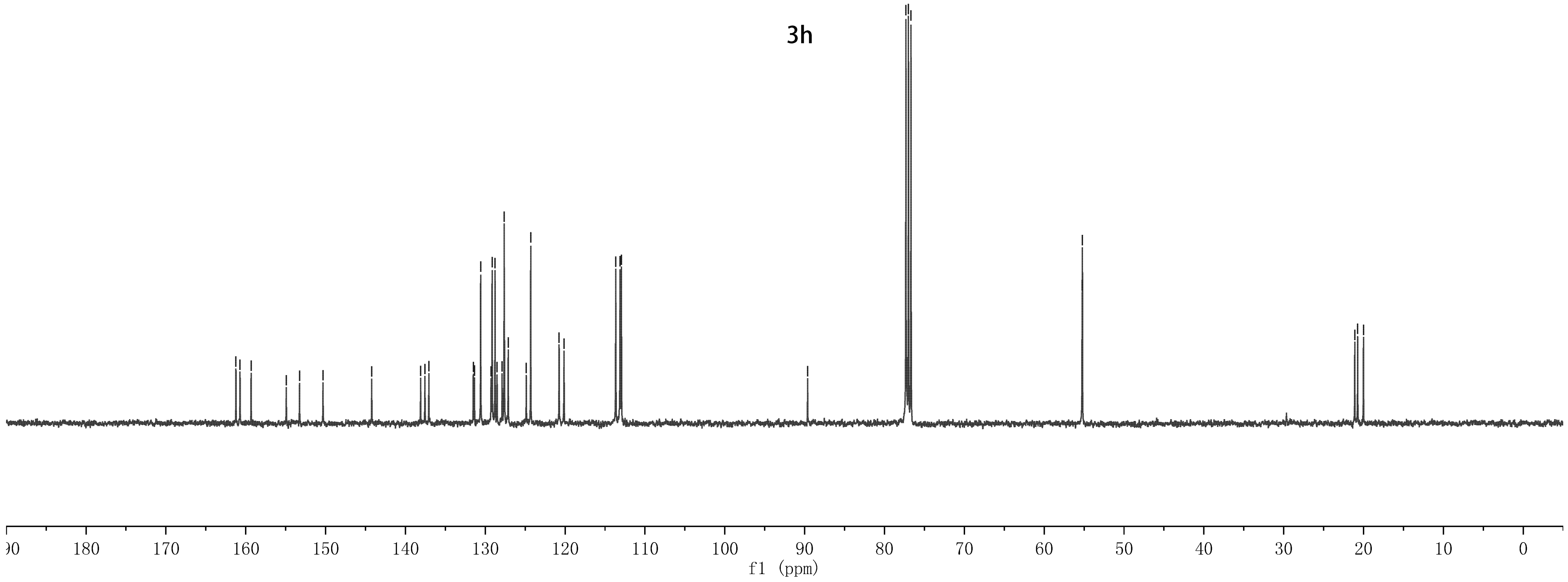

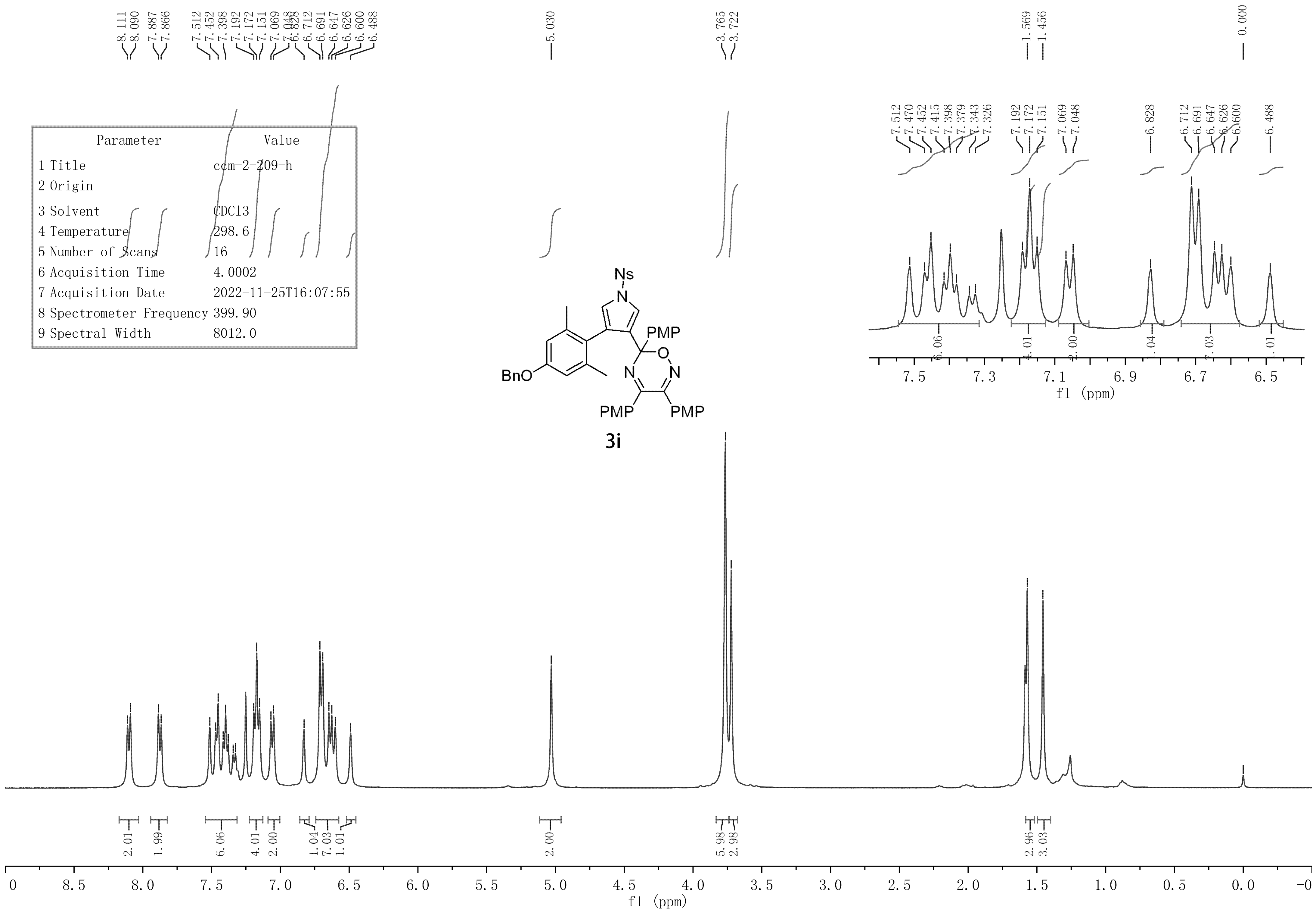

8.111
8.090
7.887
7.866
7.512
7.452
7.398
7.192
7.172
7.151
7.069
7.048
6.828
6.712
6.691
6.647
6.626
6.600
6.488
5.030
3.765
3.722
1.569
1.456
-0.000
Parameter Value
1 Title ccm-2-209-h
2 Origin
3 Solvent CDCl3
4 Temperature 298.6
5 Number of Scans 16
6 Acquisition Time 4.0002
7 Acquisition Date 2022-11-25T16:07:55
8 Spectrometer Frequency 399.90
9 Spectral Width 8012.0
Ns
N
PMP
O
N
N
BnO
PMP
PMP
3i
7.512
7.470
7.452
7.415
7.398
7.379
7.343
7.326
7.192
7.172
7.151
7.069
7.048
6.828
6.712
6.691
6.647
6.626
6.600
6.488
6.06
4.01
2.00
1.04
7.03
1.01
f1 (ppm)
2.01
1.99
6.06
4.01
2.00
1.04
7.03
1.01
2.00
5.98
2.98
2.96
3.03
f1 (ppm)

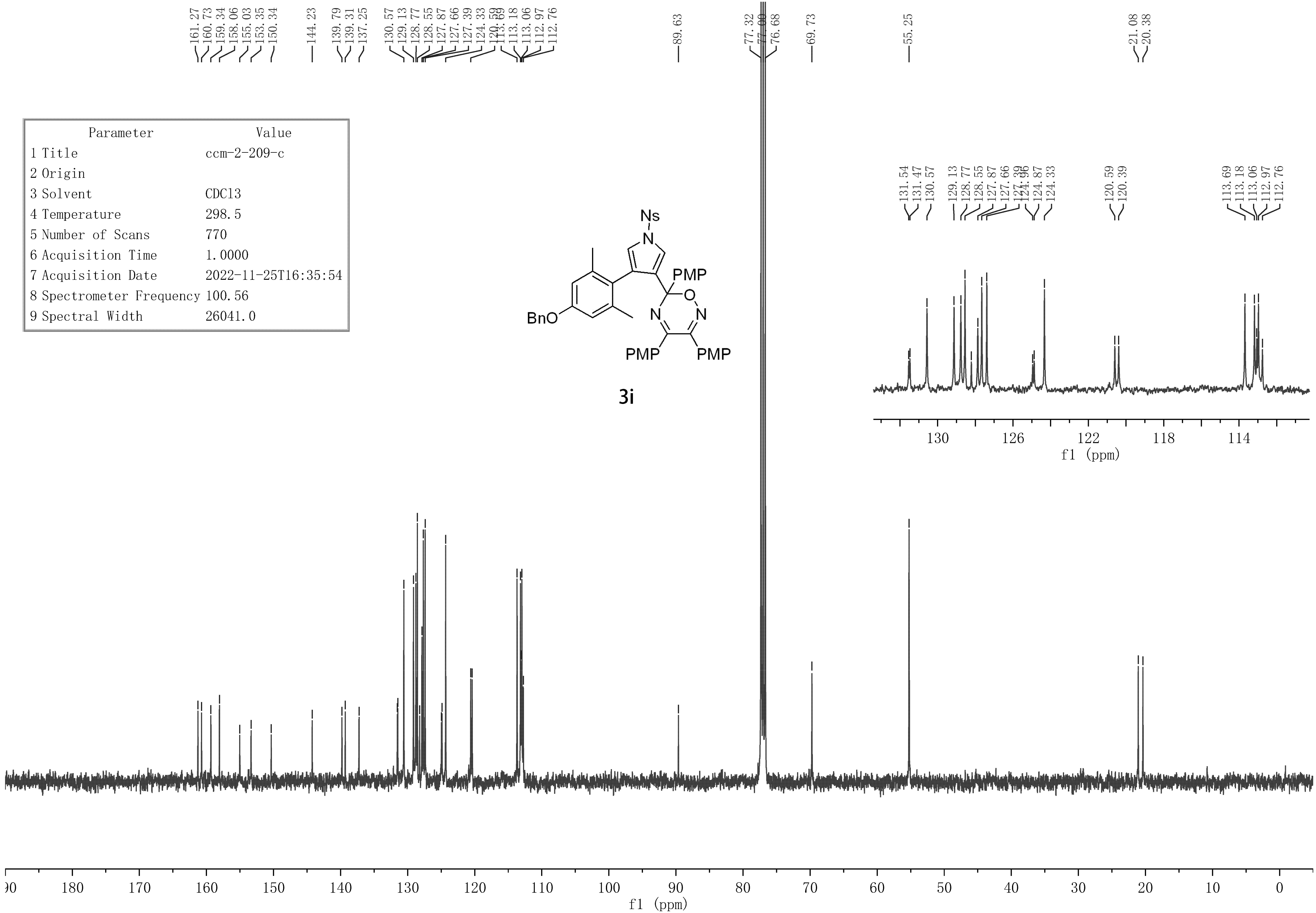

Parameter	Value
1 Title	ccm-2-209-c
2 Origin	
3 Solvent	CDCl3
4 Temperature	298.5
5 Number of Scans	770
6 Acquisition Time	1.0000
7 Acquisition Date	2022-11-25T16:35:54
8 Spectrometer Frequency	100.56
9 Spectral Width	26041.0

3i

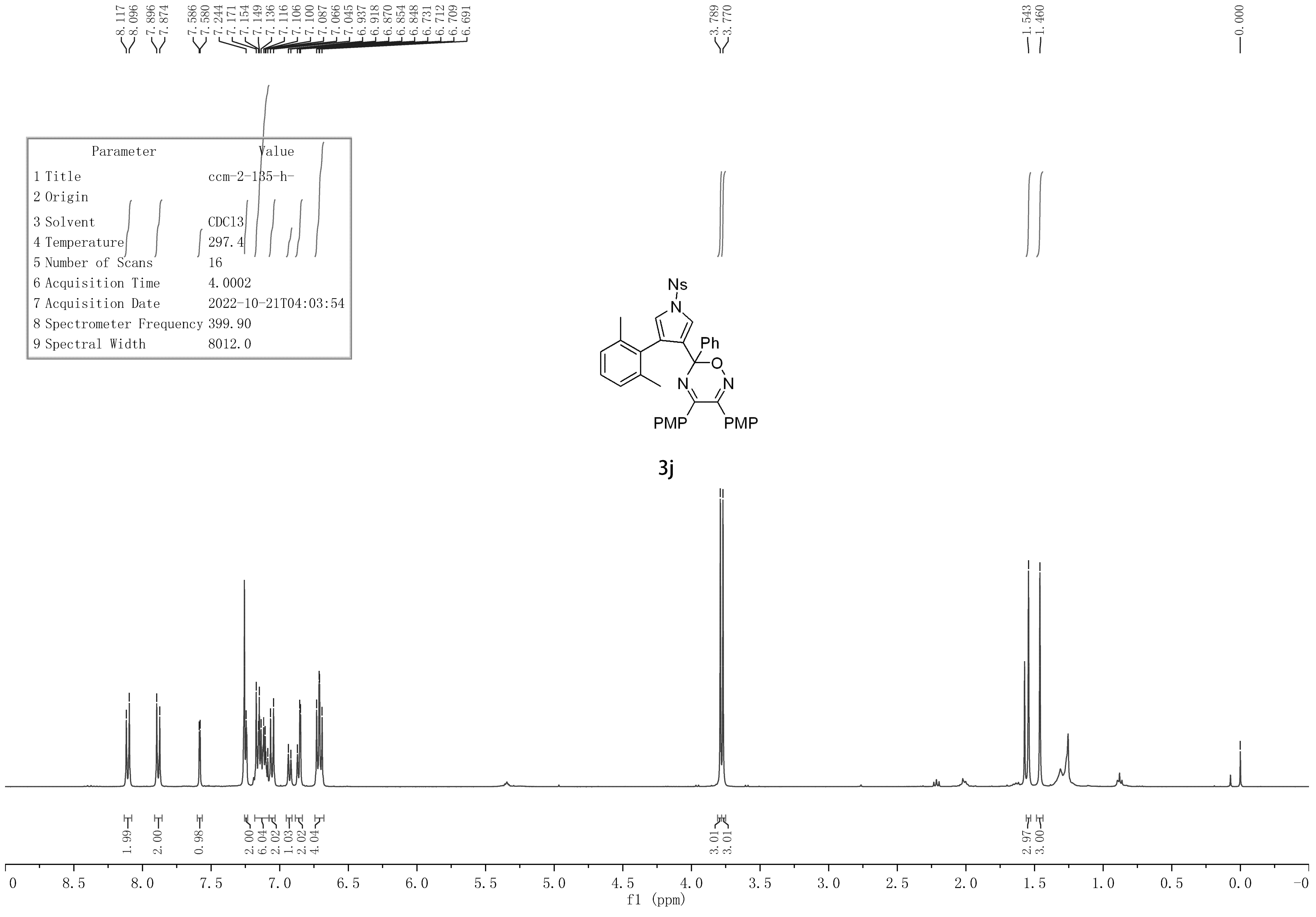
8.117
8.096
7.896
7.874
7.586
7.580
7.244
7.171
7.154
7.149
7.136
7.116
7.106
7.100
7.087
7.066
7.045
6.937
6.918
6.870
6.854
6.848
6.731
6.712
6.709
6.691
3.789
3.770
1.543
1.460
0.000
Parameter Value
1 Title ccm-2-135-h-
2 Origin
3 Solvent CDCl3
4 Temperature 297.4
5 Number of Scans 16
6 Acquisition Time 4.0002
7 Acquisition Date 2022-10-21T04:03:54
8 Spectrometer Frequency 399.90
9 Spectral Width 8012.0
Ns
N
Ph
O
N
N
PMP
PMP
3j
1.99
2.00
0.98
2.00
6.04
2.02
1.03
2.02
4.04
3.01
3.01
2.97
3.00
f1 (ppm)

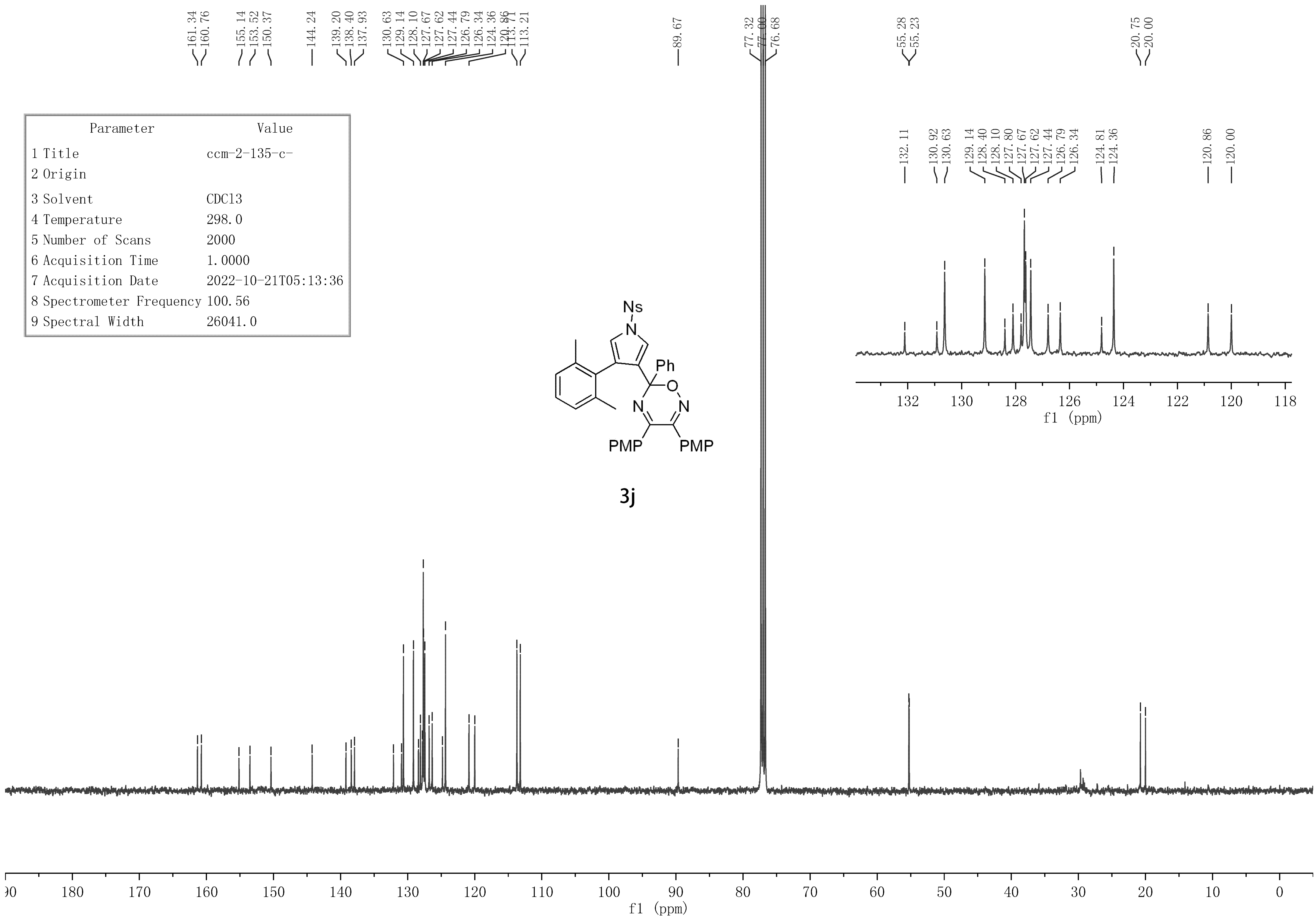
161.34
160.76
155.14
153.52
150.37
144.24
139.20
138.40
137.93
130.63
129.14
128.10
127.67
127.62
127.44
126.79
126.34
124.36
120.86
113.71
113.21
89.67
77.32
77.00
76.68
55.28
55.23
20.75
20.00
Parameter Value
1 Title ccm-2-135-c-
2 Origin
3 Solvent CDCl3
4 Temperature 298.0
5 Number of Scans 2000
6 Acquisition Time 1.0000
7 Acquisition Date 2022-10-21T05:13:36
8 Spectrometer Frequency 100.56
9 Spectral Width 26041.0
132.11
130.92
130.63
129.14
128.40
128.10
127.80
127.67
127.62
127.44
126.79
126.34
124.81
124.36
120.86
120.00
Ns
N
Ph
O
N
N
PMP
PMP
3j
f1 (ppm)

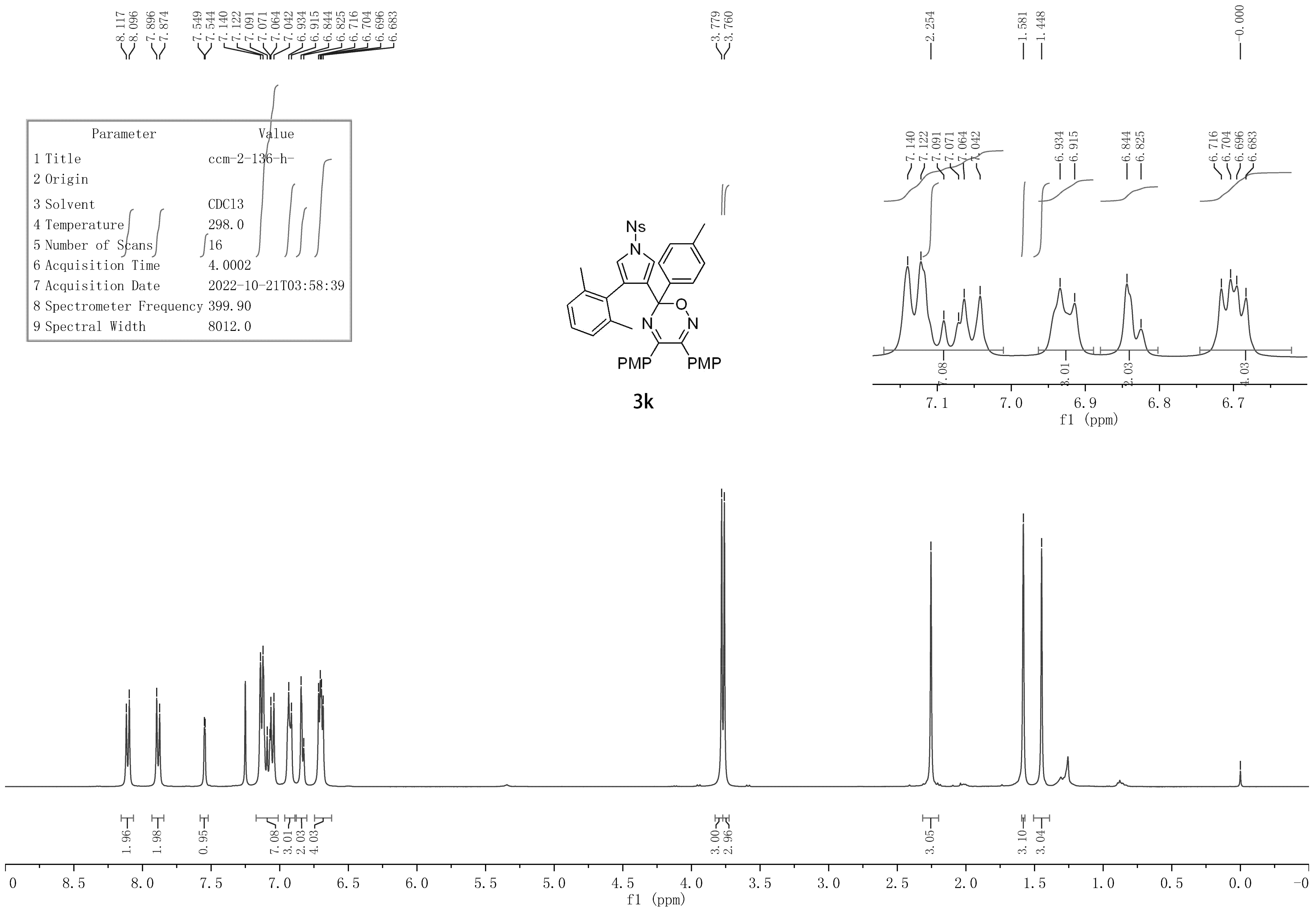

Parameter Value
1 Title ccm-2-136-h-
2 Origin
3 Solvent CDCl3
4 Temperature 298.0
5 Number of Scans 16
6 Acquisition Time 4.0002
7 Acquisition Date 2022-10-21T03:58:39
8 Spectrometer Frequency 399.90
9 Spectral Width 8012.0
Ns
N
O
N
N
PMP
PMP
3k
8.117 8.096 7.896 7.874 7.549 7.544 7.140 7.122 7.091 7.071 7.064 7.042 6.934 6.915 6.844 6.825 6.716 6.704 6.696 6.683
3.779 3.760
2.254
1.581 1.448
-0.000
1.96 1.98 0.95 7.08 3.01 2.03 4.03 3.00 2.96 3.05 3.10 3.04
f1 (ppm)

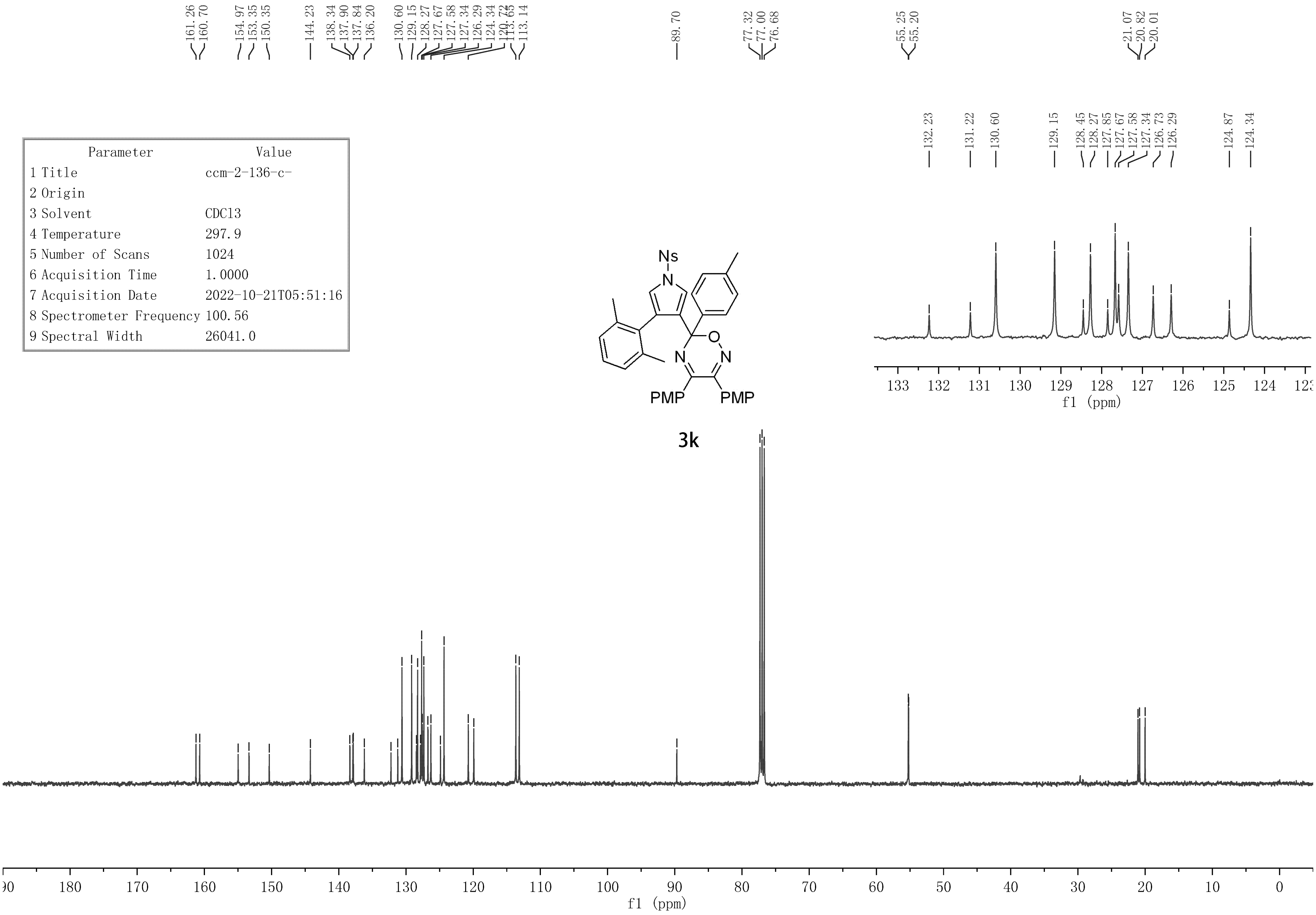

Parameter	Value
1 Title	ccm-2-136-c-
2 Origin	
3 Solvent	CDCl3
4 Temperature	297.9
5 Number of Scans	1024
6 Acquisition Time	1.0000
7 Acquisition Date	2022-10-21T05:51:16
8 Spectrometer Frequency	100.56
9 Spectral Width	26041.0

3k

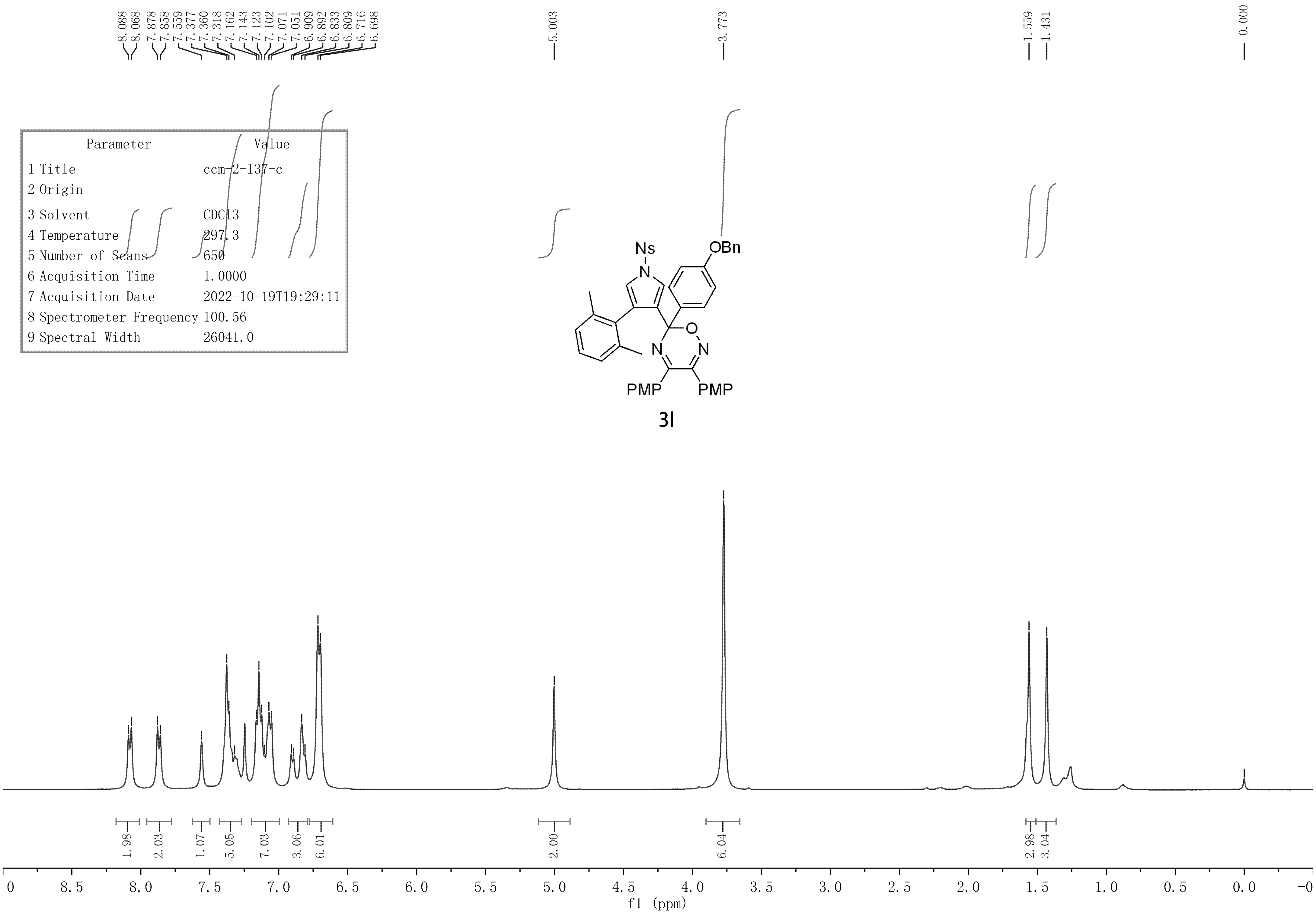

Parameter	Value
1 Title	ccm-2-137-c
2 Origin	
3 Solvent	CDCl3
4 Temperature	297.3
5 Number of Scans	650
6 Acquisition Time	1.0000
7 Acquisition Date	2022-10-19T19:29:11
8 Spectrometer Frequency	100.56
9 Spectral Width	26041.0

Parameter	Value
1 Title	ccm-2-137-c
2 Origin	
3 Solvent	CDCl3
4 Temperature	297.3
5 Number of Scans	650
6 Acquisition Time	1.0000
7 Acquisition Date	2022-10-19T19:29:11
8 Spectrometer Frequency	100.56
9 Spectral Width	26041.0

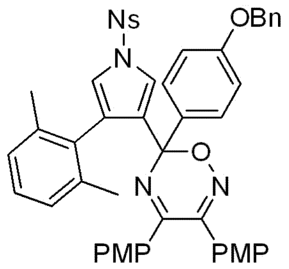

3l

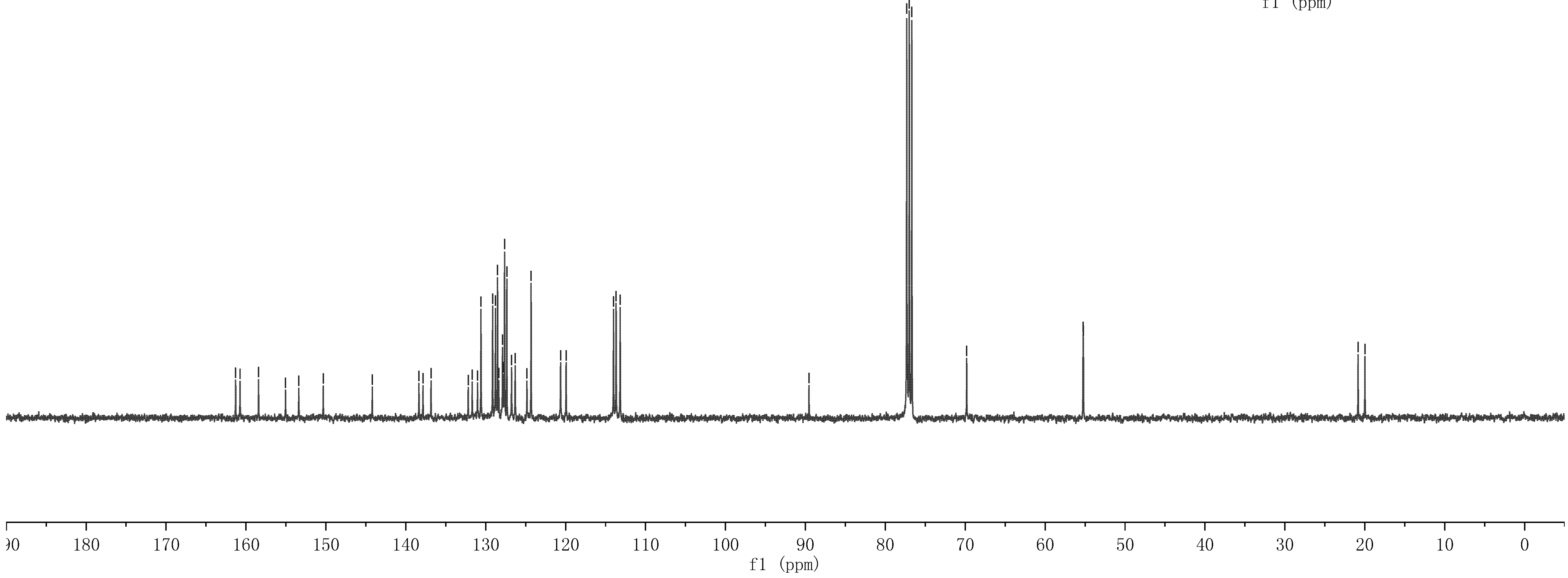

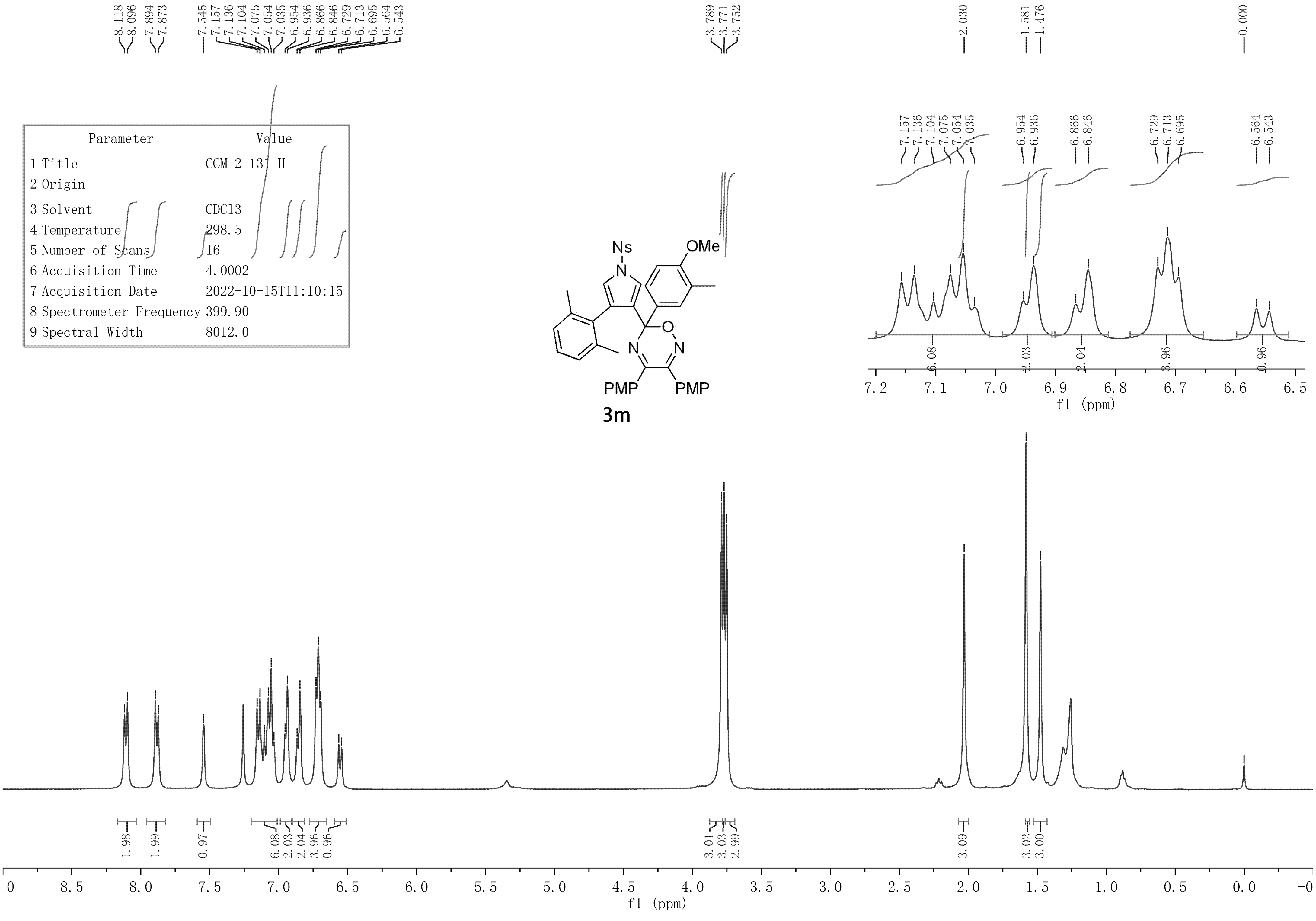

3m

Parameter	Value
1 Title	CCM-2-131-H
2 Origin	
3 Solvent	CDCl3
4 Temperature	298.5
5 Number of Scans	16
6 Acquisition Time	4.0002
7 Acquisition Date	2022-10-15T11:10:15
8 Spectrometer Frequency	399.90
9 Spectral Width	8012.0

Parameter	Value
1 Title	CCM-2-131-C
2 Origin	
3 Solvent	CDCl3
4 Temperature	298.4
5 Number of Scans	650
6 Acquisition Time	1.0000
7 Acquisition Date	2022-10-13T20:28:15
8 Spectrometer Frequency	100.56
9 Spectral Width	26041.0

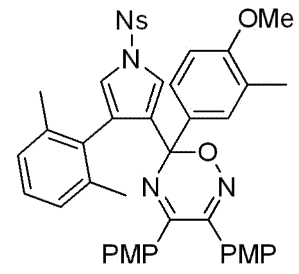

3m

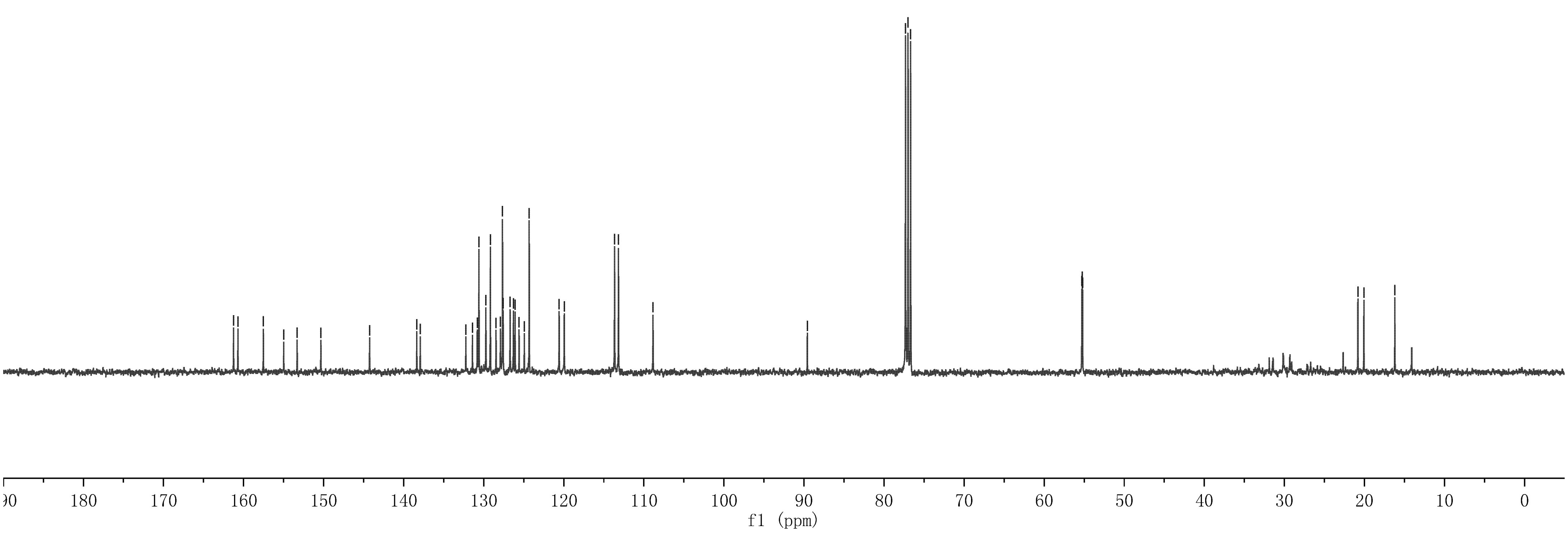

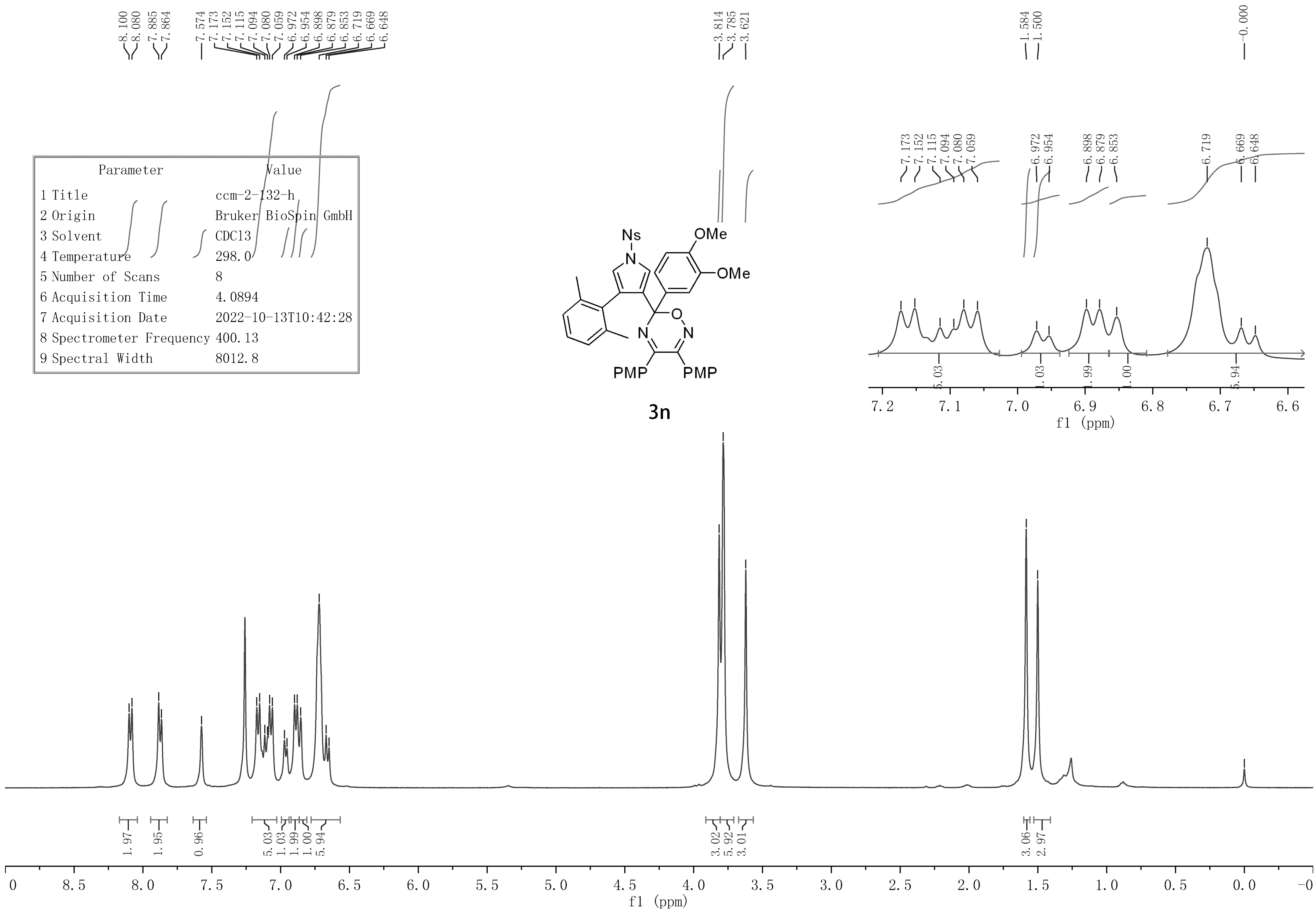
Parameter Value
1 Title ccm-2-132-h
2 Origin Bruker BioSpin GmbH
3 Solvent CDCl3
4 Temperature 298.0
5 Number of Scans 8
6 Acquisition Time 4.0894
7 Acquisition Date 2022-10-13T10:42:28
8 Spectrometer Frequency 400.13
9 Spectral Width 8012.8
8.100 8.080 7.885 7.864 7.574 7.173 7.152 7.115 7.094 7.080 7.059 6.972 6.954 6.898 6.879 6.853 6.719 6.669 6.648
3.814 3.785 3.621
1.584 1.500
-0.000
1.97 1.95 0.96 5.03 1.03 1.99 1.00 5.94 3.02 5.92 3.01 3.06 2.97
f1 (ppm)
Ns
OMe
OMe
O
N
N
PMP
PMP

3n

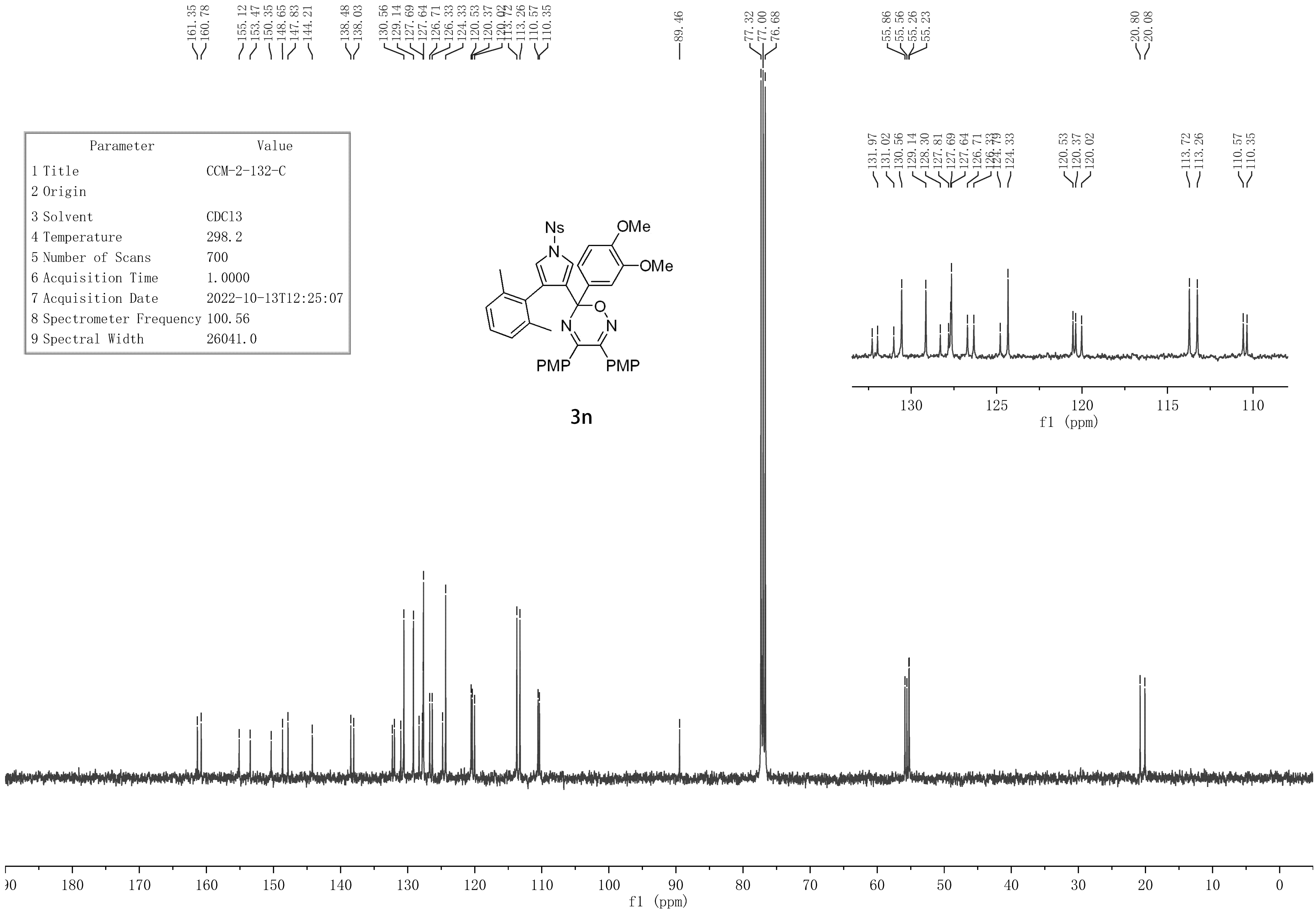
161.35
160.78
155.12
153.47
150.35
148.65
147.83
144.21
138.48
138.03
130.56
129.14
127.69
127.64
126.71
126.33
124.33
120.53
120.37
120.02
113.72
113.26
110.57
110.35
89.46
77.32
77.00
76.68
55.86
55.56
55.26
55.23
20.80
20.08
Parameter Value
1 Title CCM-2-132-C
2 Origin
3 Solvent CDCl3
4 Temperature 298.2
5 Number of Scans 700
6 Acquisition Time 1.0000
7 Acquisition Date 2022-10-13T12:25:07
8 Spectrometer Frequency 100.56
9 Spectral Width 26041.0
Ns
N
OMe
OMe
O
N
N
PMP
PMP
131.97
131.02
130.56
129.14
128.30
127.81
127.69
127.64
126.71
126.33
124.79
124.33
120.53
120.37
120.02
113.72
113.26
110.57
110.35
130
125
120
115
110
f1 (ppm)
180
170
160
150
140
130
120
110
100
90
80
70
60
50
40
30
20
10
0
f1 (ppm)

3n

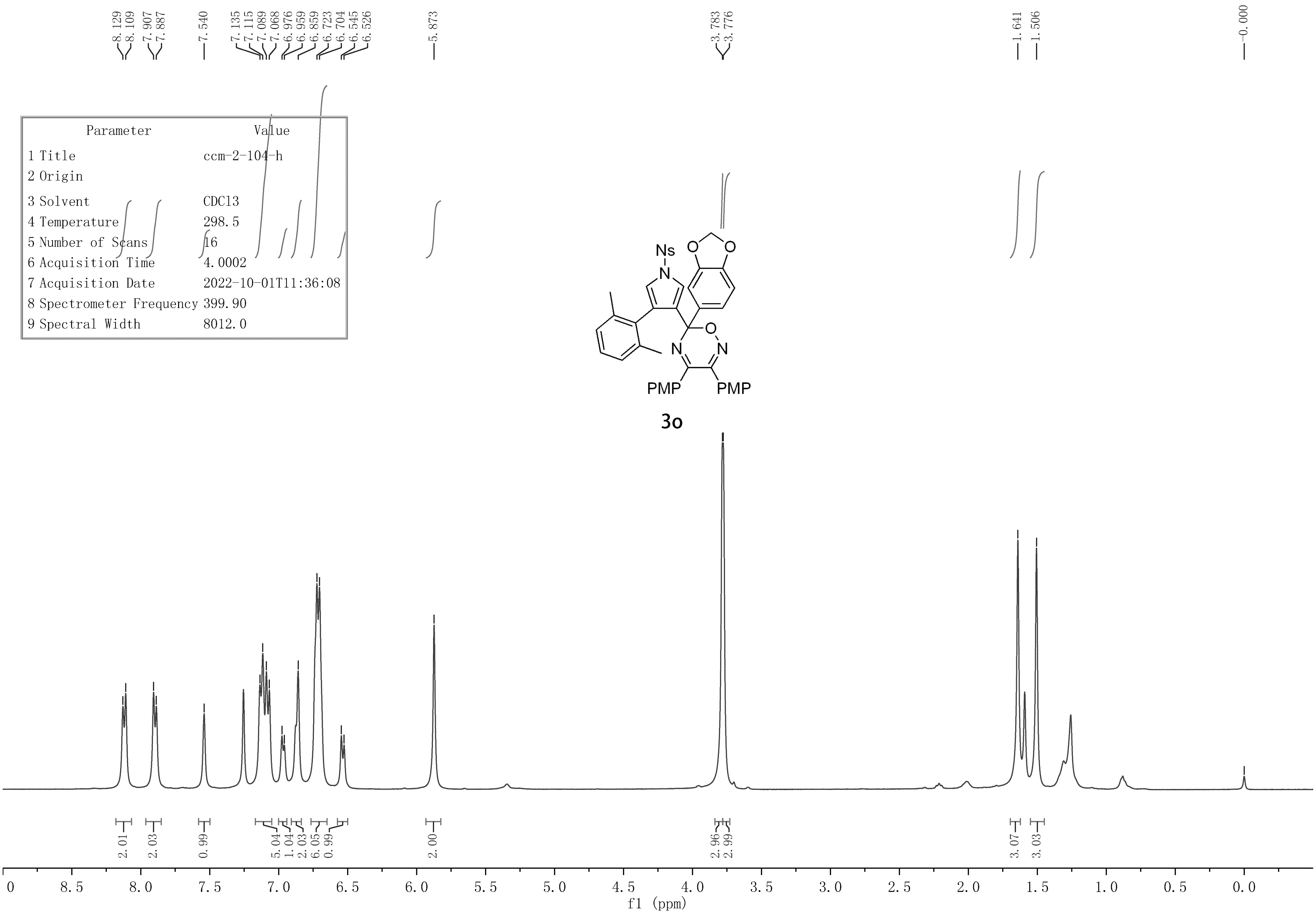
8.129
8.109
7.907
7.887
7.540
7.135
7.115
7.089
7.068
6.976
6.959
6.859
6.723
6.704
6.545
6.526
5.873
3.783
3.776
1.641
1.506
-0.000
Parameter Value
1 Title ccm-2-104-h
2 Origin
3 Solvent CDCl3
4 Temperature 298.5
5 Number of Scans 16
6 Acquisition Time 4.0002
7 Acquisition Date 2022-10-01T11:36:08
8 Spectrometer Frequency 399.90
9 Spectral Width 8012.0
Ns
N
O
O
O
N
N
PMP
PMP
2.01
2.03
0.99
5.04
1.04
2.03
6.05
0.99
2.00
2.96
2.99
3.07
3.03
f1 (ppm)

3o

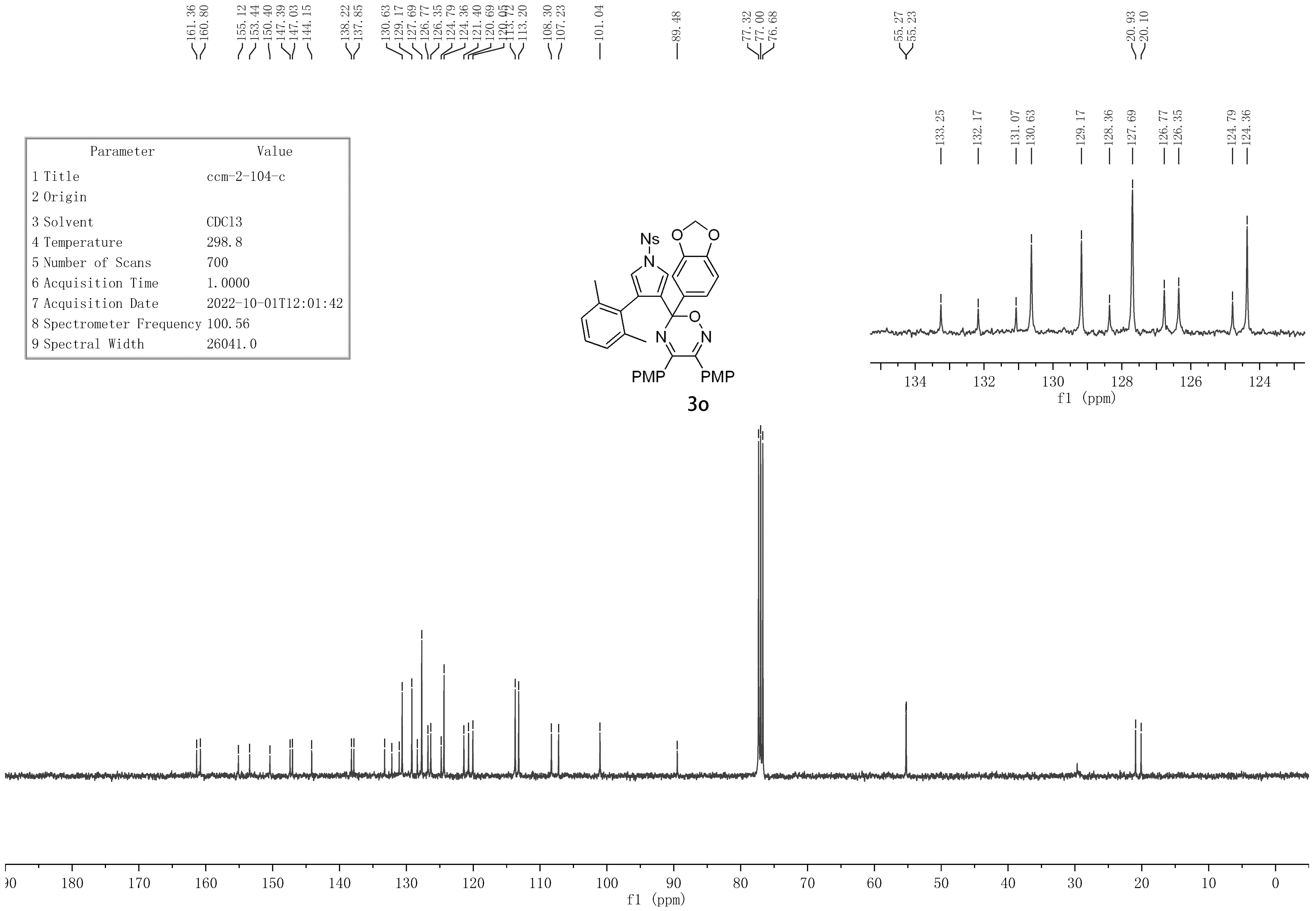
161.36
160.80
155.12
153.44
150.40
147.39
147.03
144.15
138.22
137.85
130.63
129.17
127.69
126.77
126.35
124.79
124.36
121.40
120.69
113.72
113.20
108.30
107.23
101.04
89.48
77.32
77.00
76.68
55.27
55.23
20.93
20.10
Parameter Value
1 Title ccm-2-104-c
2 Origin
3 Solvent CDCl3
4 Temperature 298.8
5 Number of Scans 700
6 Acquisition Time 1.0000
7 Acquisition Date 2022-10-01T12:01:42
8 Spectrometer Frequency 100.56
9 Spectral Width 26041.0
Ns
O
N
PMP
PMP
133.25
132.17
131.07
130.63
129.17
128.36
127.69
126.77
126.35
124.79
124.36
f1 (ppm)
f1 (ppm)

3o

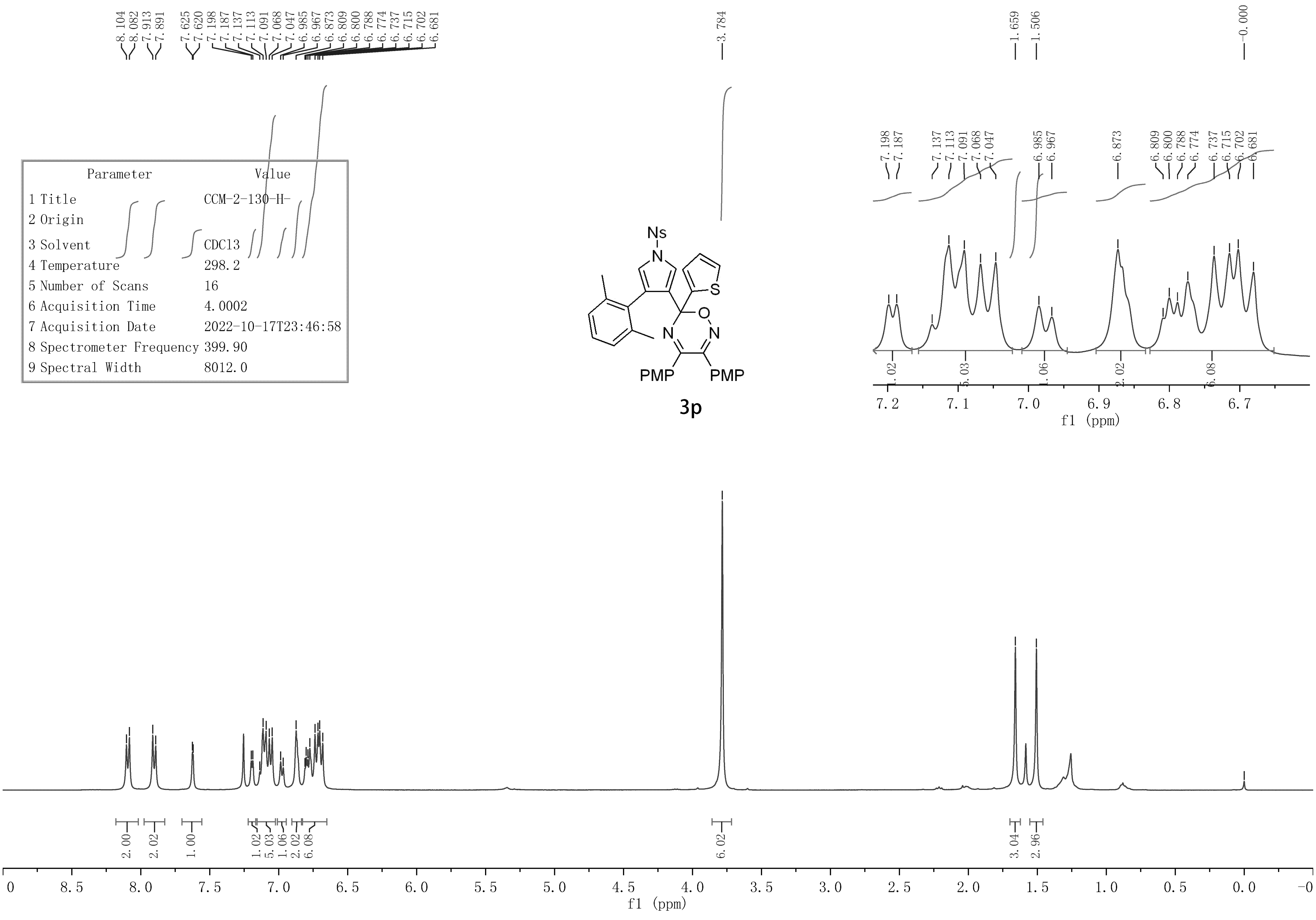

8.104
8.082
7.913
7.891
7.625
7.620
7.198
7.187
7.137
7.113
7.091
7.068
7.047
6.985
6.967
6.873
6.809
6.800
6.788
6.774
6.737
6.715
6.702
6.681
3.784
1.659
1.506
-0.000
Parameter Value
1 Title CCM-2-130-H-
2 Origin
3 Solvent CDCl3
4 Temperature 298.2
5 Number of Scans 16
6 Acquisition Time 4.0002
7 Acquisition Date 2022-10-17T23:46:58
8 Spectrometer Frequency 399.90
9 Spectral Width 8012.0
Ns
N
S
O
N
N
PMP
PMP
3p
1.02
5.03
1.06
2.02
6.08
7.2
7.1
7.0
6.9
6.8
6.7
f1 (ppm)
2.00
2.02
1.00
1.02
5.03
1.06
2.02
6.08
6.02
3.04
2.96
8.5
8.0
7.5
7.0
6.5
6.0
5.5
5.0
4.5
4.0
3.5
3.0
2.5
2.0
1.5
1.0
0.5
0.0
f1 (ppm)

Parameter	Value
1 Title	CCM-2-130-C-
2 Origin	
3 Solvent	CDCl3
4 Temperature	298.4
5 Number of Scans	1024
6 Acquisition Time	1.0000
7 Acquisition Date	2022-10-18T00:23:42
8 Spectrometer Frequency	100.56
9 Spectral Width	26041.0

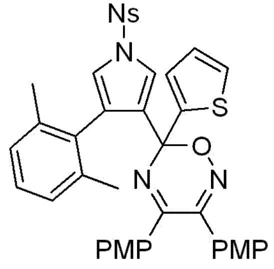

3p

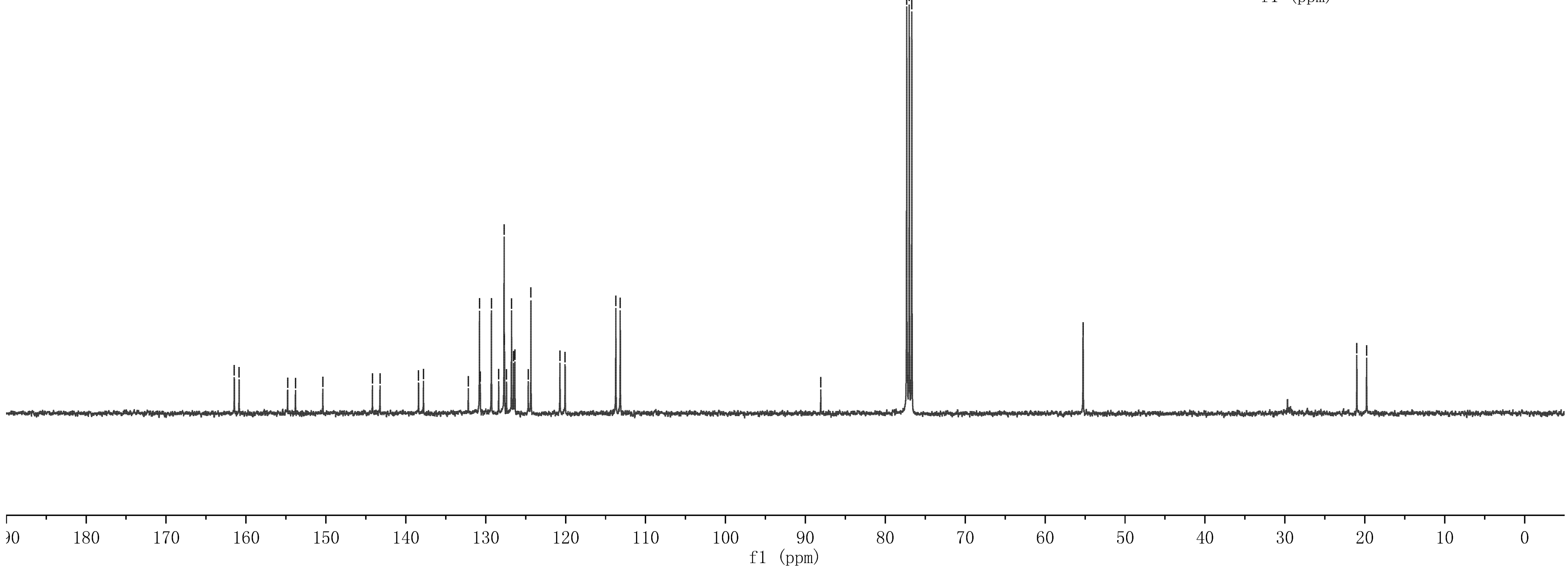

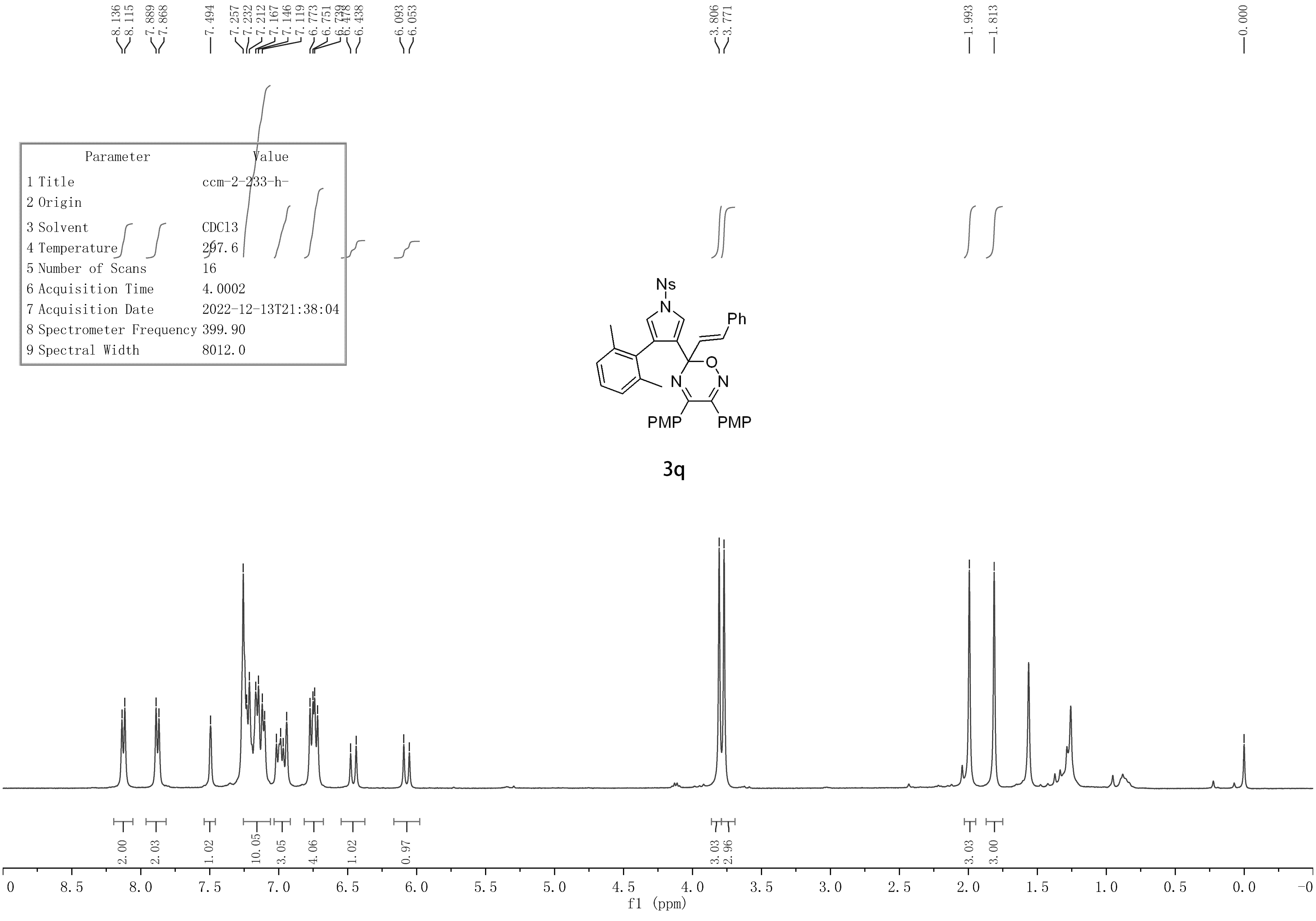
8.136
8.115
7.889
7.868
7.494
7.257
7.232
7.212
7.167
7.146
7.119
6.773
6.751
6.739
6.478
6.438
6.093
6.053
3.806
3.771
1.993
1.813
0.000
Parameter Value
1 Title ccm-2-233-h-
2 Origin
3 Solvent CDCl3
4 Temperature 297.6
5 Number of Scans 16
6 Acquisition Time 4.0002
7 Acquisition Date 2022-12-13T21:38:04
8 Spectrometer Frequency 399.90
9 Spectral Width 8012.0
Ns
N
Ph
O
N
N
PMP
PMP
3q
2.00
2.03
1.02
10.05
3.05
4.06
1.02
0.97
3.03
2.96
3.03
3.00
8.5
8.0
7.5
7.0
6.5
6.0
5.5
5.0
4.5
4.0
3.5
3.0
2.5
2.0
1.5
1.0
0.5
0.0
f1 (ppm)

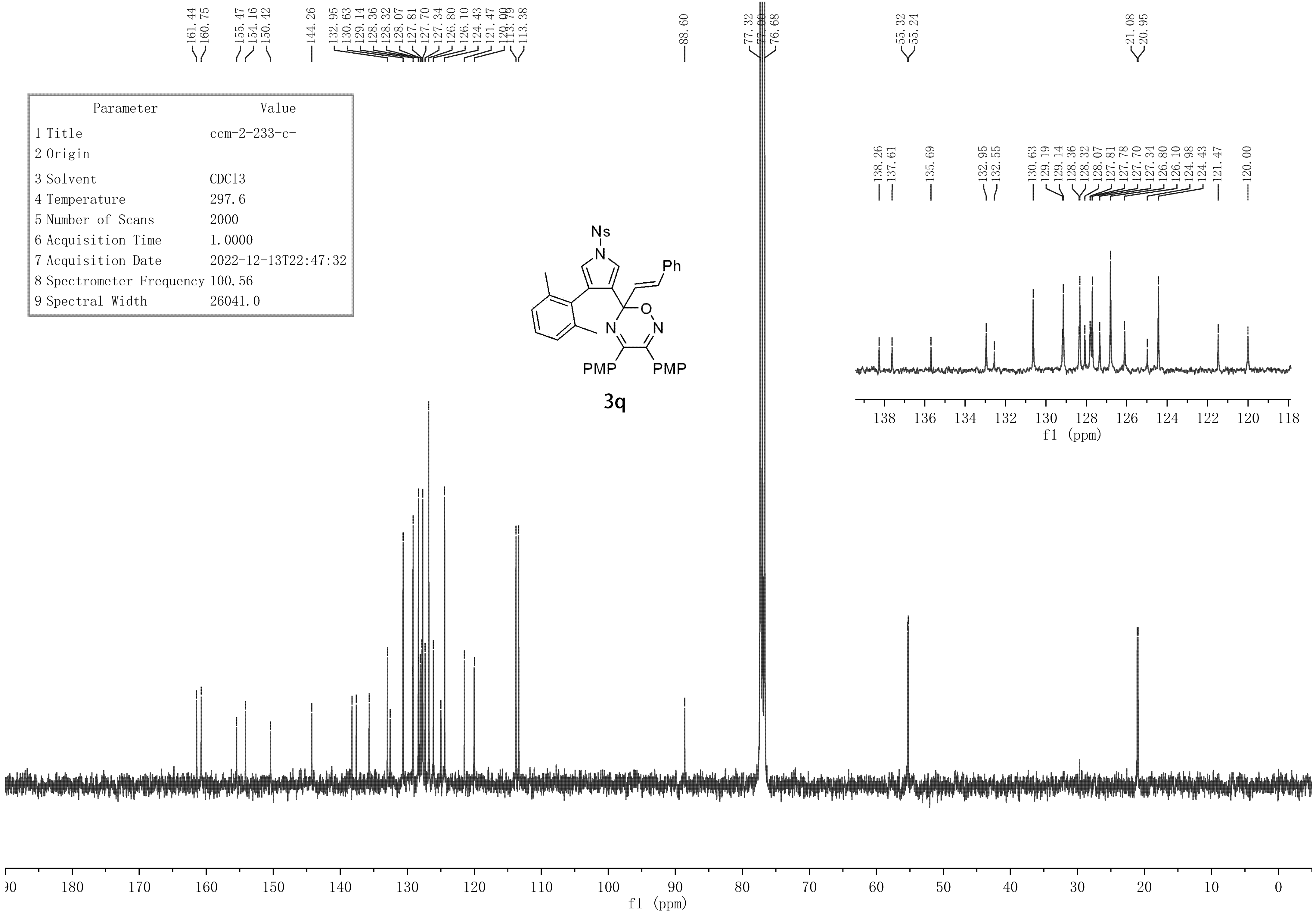

Parameter	Value
1 Title	ccm-2-233-c-
2 Origin	
3 Solvent	CDCl3
4 Temperature	297.6
5 Number of Scans	2000
6 Acquisition Time	1.0000
7 Acquisition Date	2022-12-13T22:47:32
8 Spectrometer Frequency	100.56
9 Spectral Width	26041.0

3q

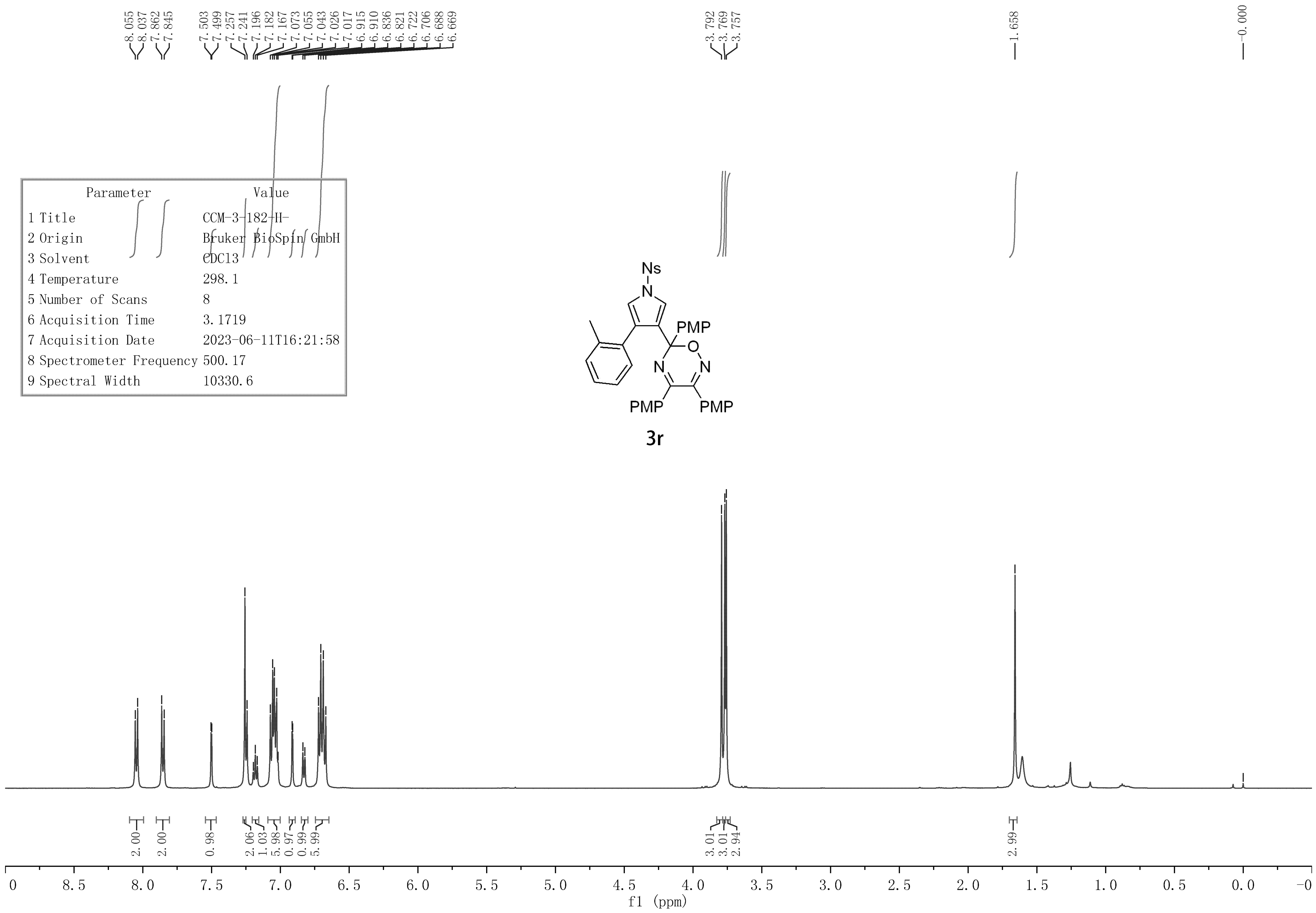
8.055
8.037
7.862
7.845
7.503
7.499
7.257
7.241
7.196
7.182
7.167
7.073
7.055
7.043
7.026
7.017
6.915
6.910
6.836
6.821
6.722
6.706
6.688
6.669
3.792
3.769
3.757
1.658
-0.000
Parameter Value
1 Title CCM-3-182-H-
2 Origin Bruker BioSpin GmbH
3 Solvent CDCl3
4 Temperature 298.1
5 Number of Scans 8
6 Acquisition Time 3.1719
7 Acquisition Date 2023-06-11T16:21:58
8 Spectrometer Frequency 500.17
9 Spectral Width 10330.6
Ns
N
PMP
O
N
N
PMP
PMP
3r
2.00
2.00
0.98
2.06
1.03
5.98
0.97
0.99
5.99
3.01
3.01
2.94
2.99
f1 (ppm)

Parameter	Value
1 Title	CCM-3-182-C-
2 Origin	Bruker BioSpin GmbH
3 Solvent	CDCl3
4 Temperature	298.4
5 Number of Scans	397
6 Acquisition Time	1.1010
7 Acquisition Date	2023-06-11T16:25:27
8 Spectrometer Frequency	125.77
9 Spectral Width	29761.9

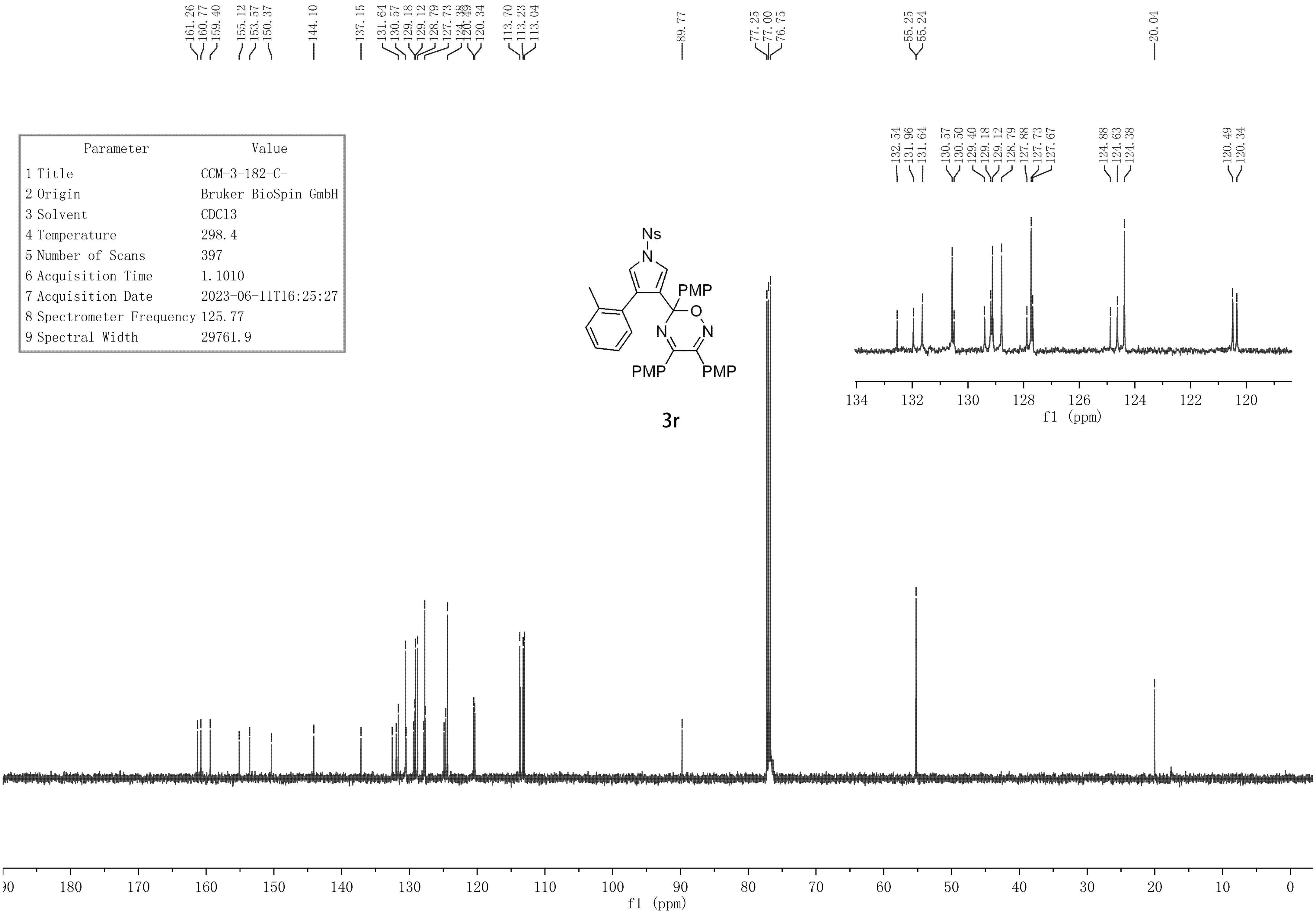

3r

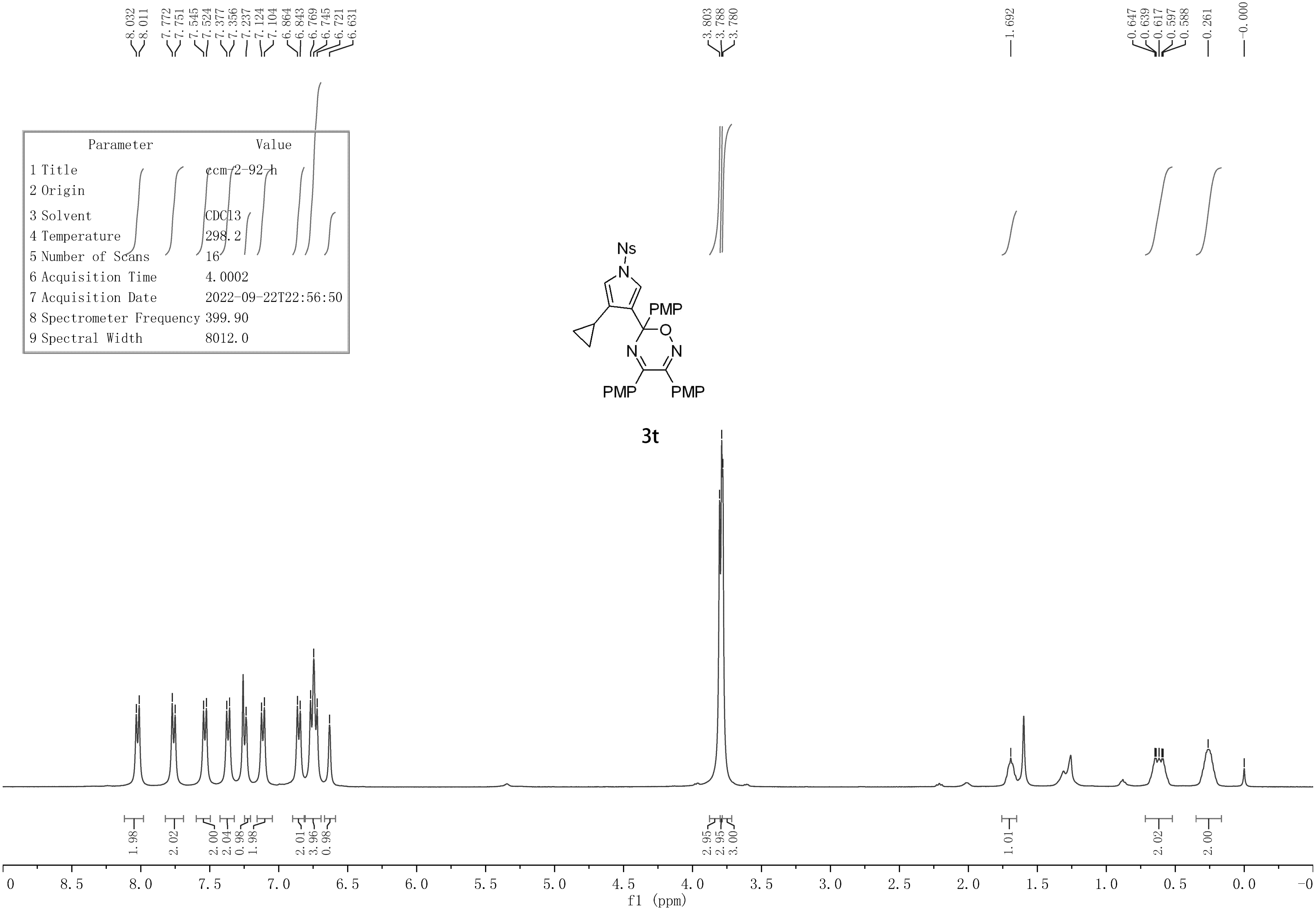
8.032
8.011
7.772
7.751
7.545
7.524
7.377
7.356
7.237
7.124
7.104
6.864
6.843
6.769
6.745
6.721
6.631
3.803
3.788
3.780
1.692
0.647
0.639
0.617
0.597
0.588
0.261
-0.000
Parameter Value
1 Title ccm-2-92-h
2 Origin
3 Solvent CDCl3
4 Temperature 298.2
5 Number of Scans 16
6 Acquisition Time 4.0002
7 Acquisition Date 2022-09-22T22:56:50
8 Spectrometer Frequency 399.90
9 Spectral Width 8012.0
Ns
N
PMP
O
N
N
PMP
PMP
3t
1.98
2.02
2.00
2.04
0.98
1.98
2.01
3.96
0.98
2.95
2.95
3.00
1.01
2.02
2.00
f1 (ppm)

Parameter	Value
1 Title	ccm-2-92-c
2 Origin	
3 Solvent	CDCl3
4 Temperature	298.8
5 Number of Scans	1024
6 Acquisition Time	1.0000
7 Acquisition Date	2022-09-22T23:33:23
8 Spectrometer Frequency	100.56
9 Spectral Width	26041.0

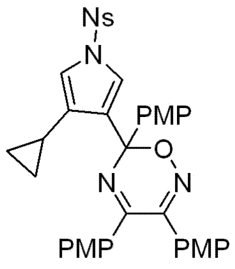

3t

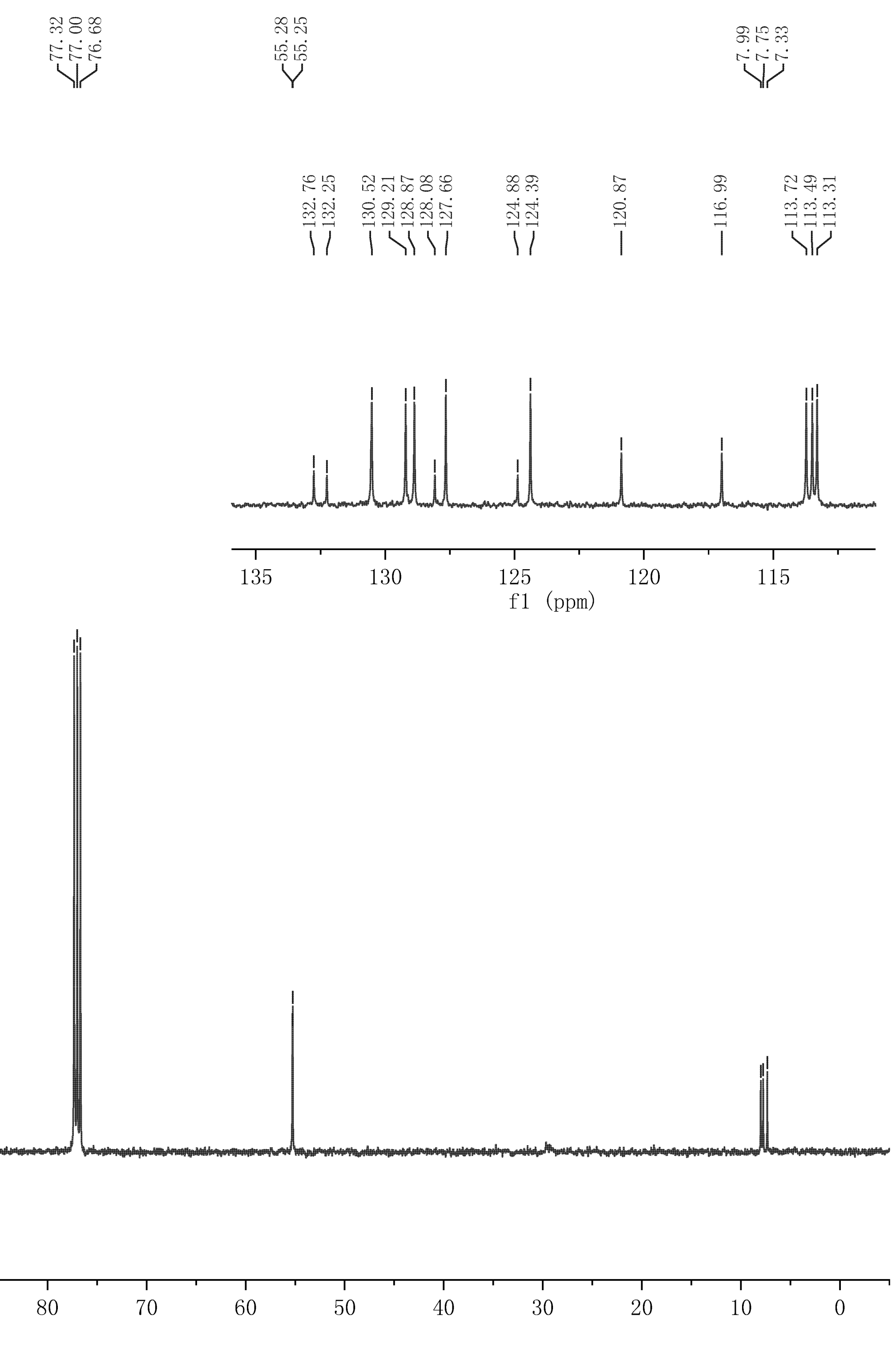

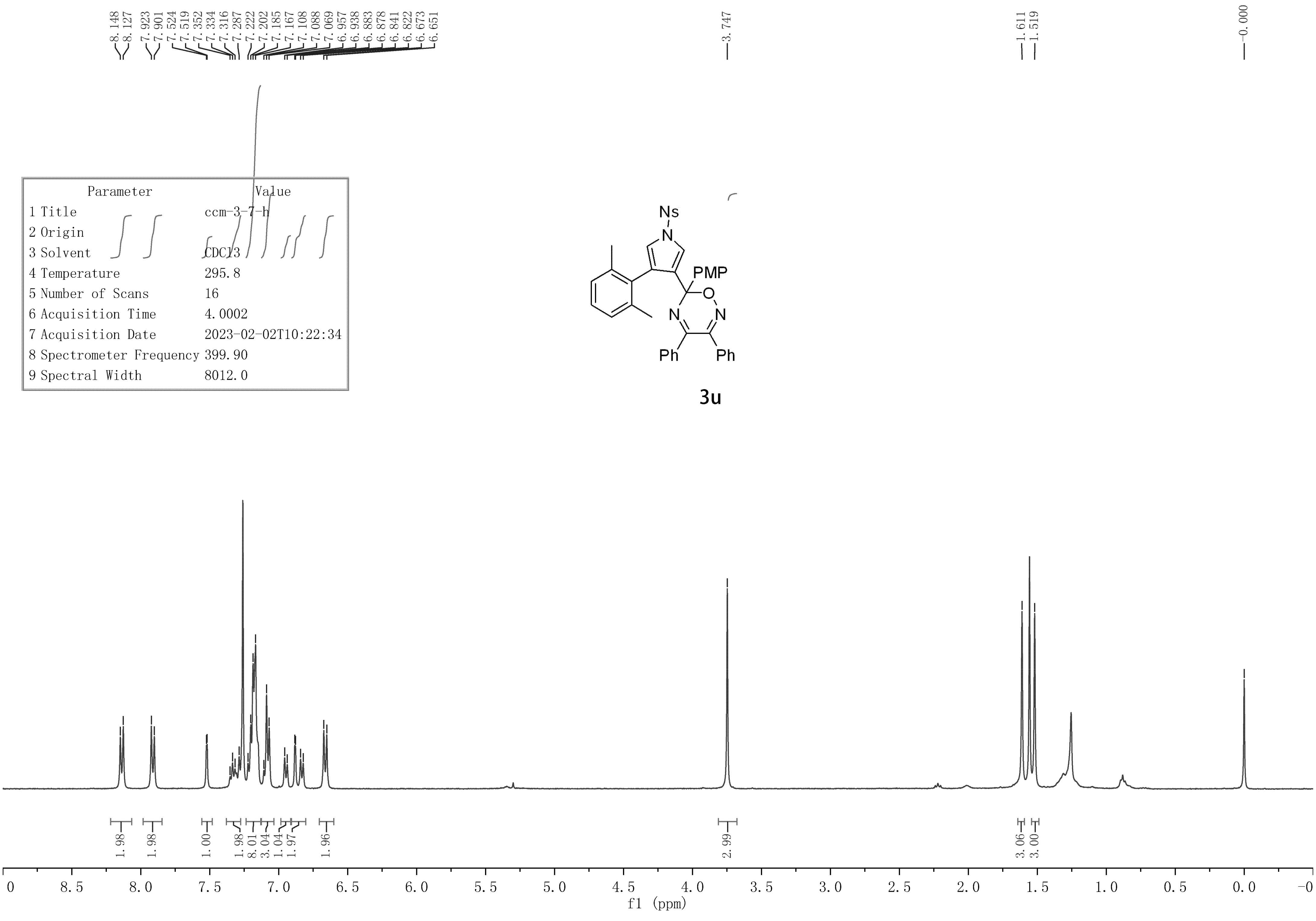

8.148
8.127
7.923
7.901
7.524
7.519
7.352
7.334
7.316
7.287
7.222
7.202
7.185
7.167
7.108
7.088
7.069
6.957
6.938
6.883
6.878
6.841
6.822
6.673
6.651
3.747
1.611
1.519
-0.000
Parameter Value
1 Title ccm-3-7-h
2 Origin
3 Solvent CDCl3
4 Temperature 295.8
5 Number of Scans 16
6 Acquisition Time 4.0002
7 Acquisition Date 2023-02-02T10:22:34
8 Spectrometer Frequency 399.90
9 Spectral Width 8012.0
Ns
N
PMP
O
N
N
Ph
Ph
3u
1.98
1.98
1.00
1.98
8.01
3.04
1.04
1.97
1.96
2.99
3.06
3.00
0
8.5
8.0
7.5
7.0
6.5
6.0
5.5
5.0
4.5
4.0
3.5
3.0
2.5
2.0
1.5
1.0
0.5
0.0
-0
f1 (ppm)

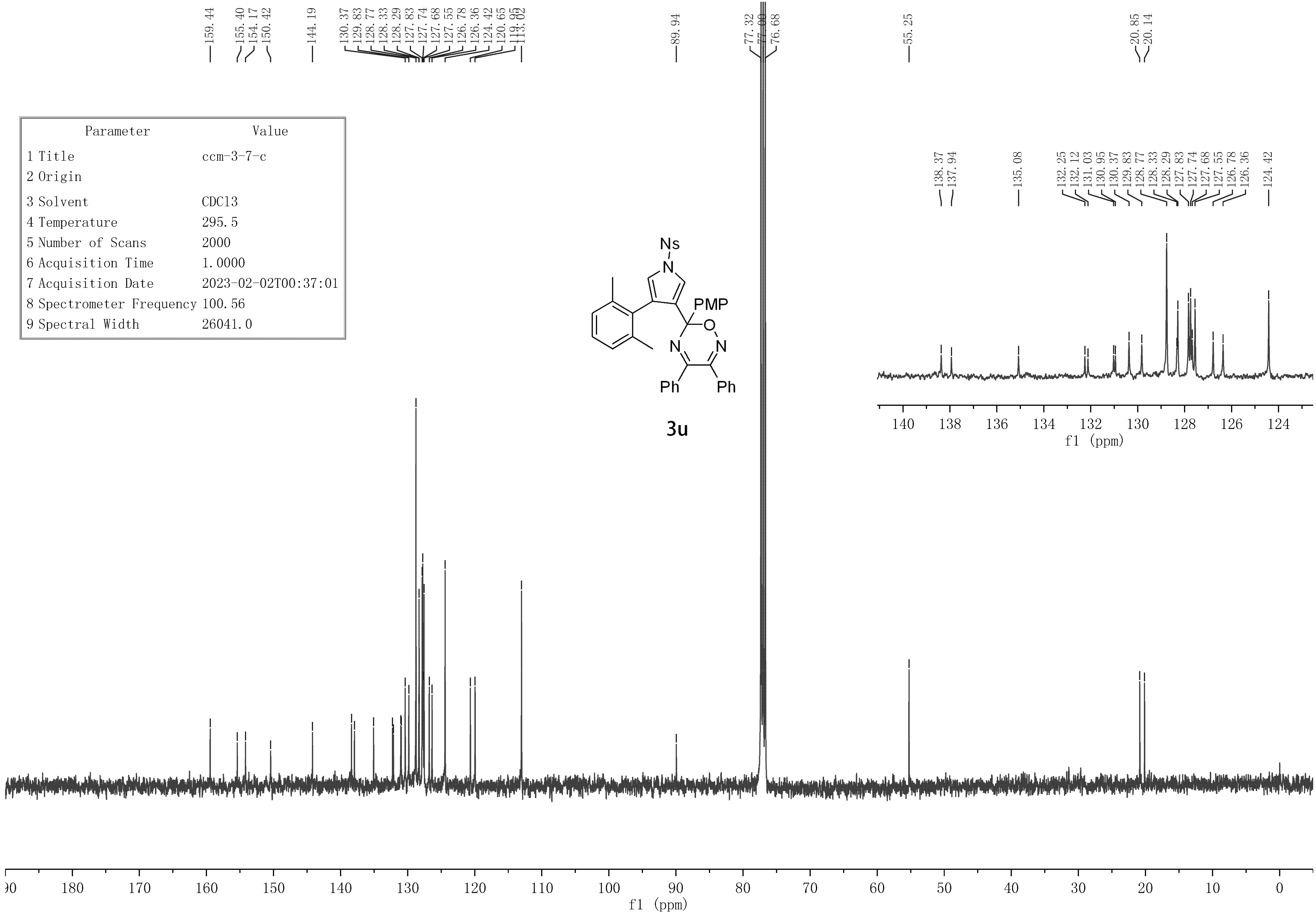

	Parameter	Value
1	Title	ccm-3-7-c
2	Origin	
3	Solvent	CDCl3
4	Temperature	295.5
5	Number of Scans	2000
6	Acquisition Time	1.0000
7	Acquisition Date	2023-02-02T00:37:01
8	Spectrometer Frequency	100.56
9	Spectral Width	26041.0

3u

Parameter	Value
1 Title	ccm-3-8-h
2 Origin	Bruker BioSpin GmbH
3 Solvent	CDCl3
4 Temperature	298.0
5 Number of Scans	9
6 Acquisition Time	4.0894
7 Acquisition Date	2023-02-02T20:05:39
8 Spectrometer Frequency	400.13
9 Spectral Width	8012.8

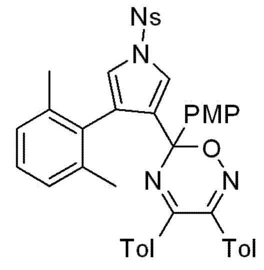

3v

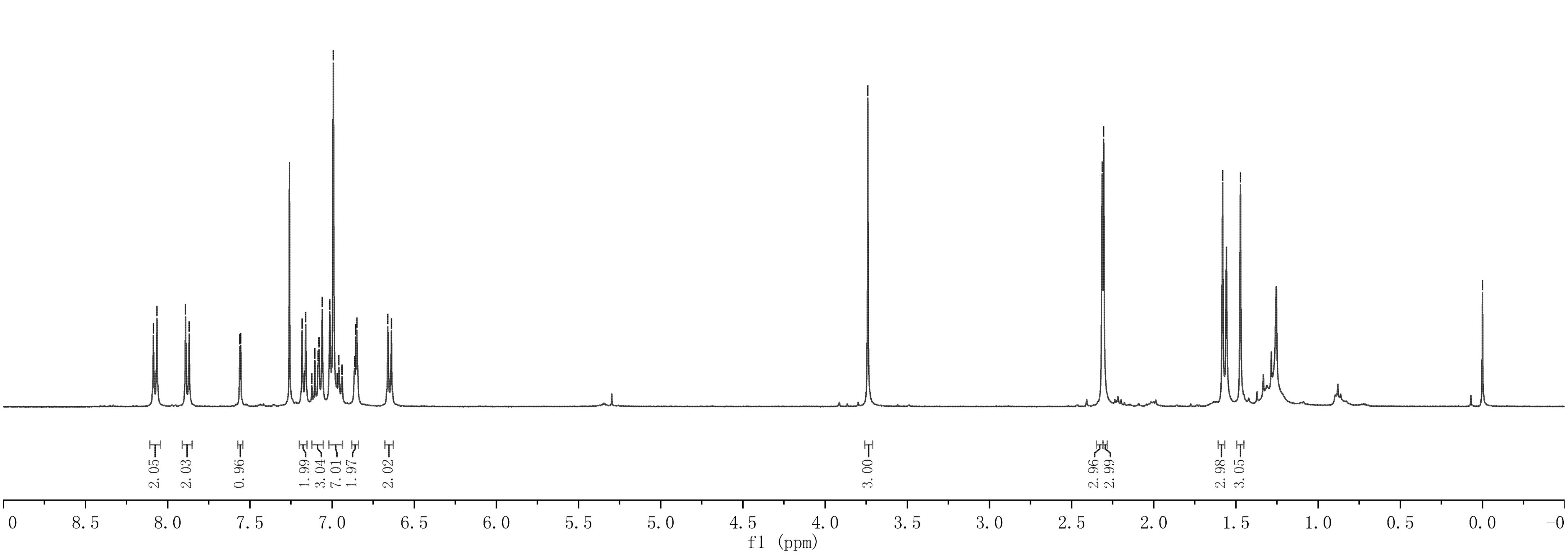

Parameter	Value
1 Title	CCM-3-8-C
2 Origin	
3 Solvent	CDCl3
4 Temperature	295.6
5 Number of Scans	1600
6 Acquisition Time	1.0000
7 Acquisition Date	2023-02-02T21:14:21
8 Spectrometer Frequency	100.56
9 Spectral Width	26041.0

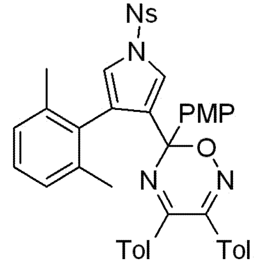

3v

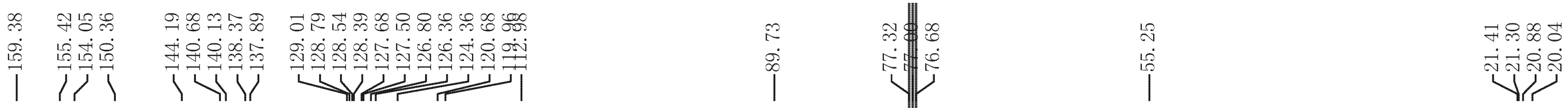

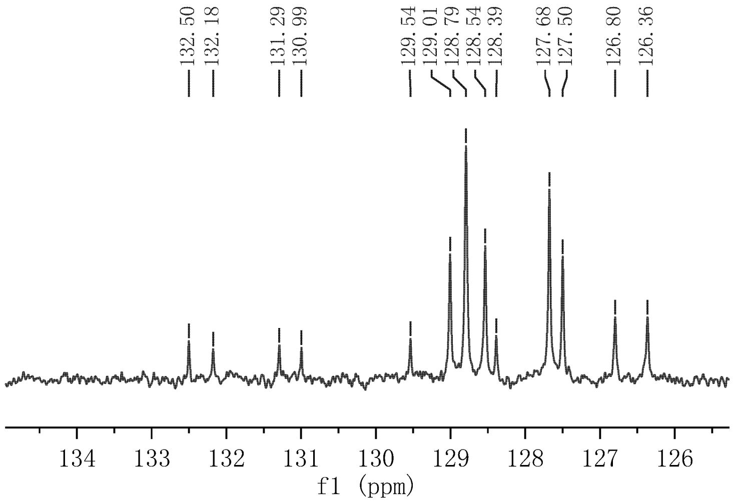

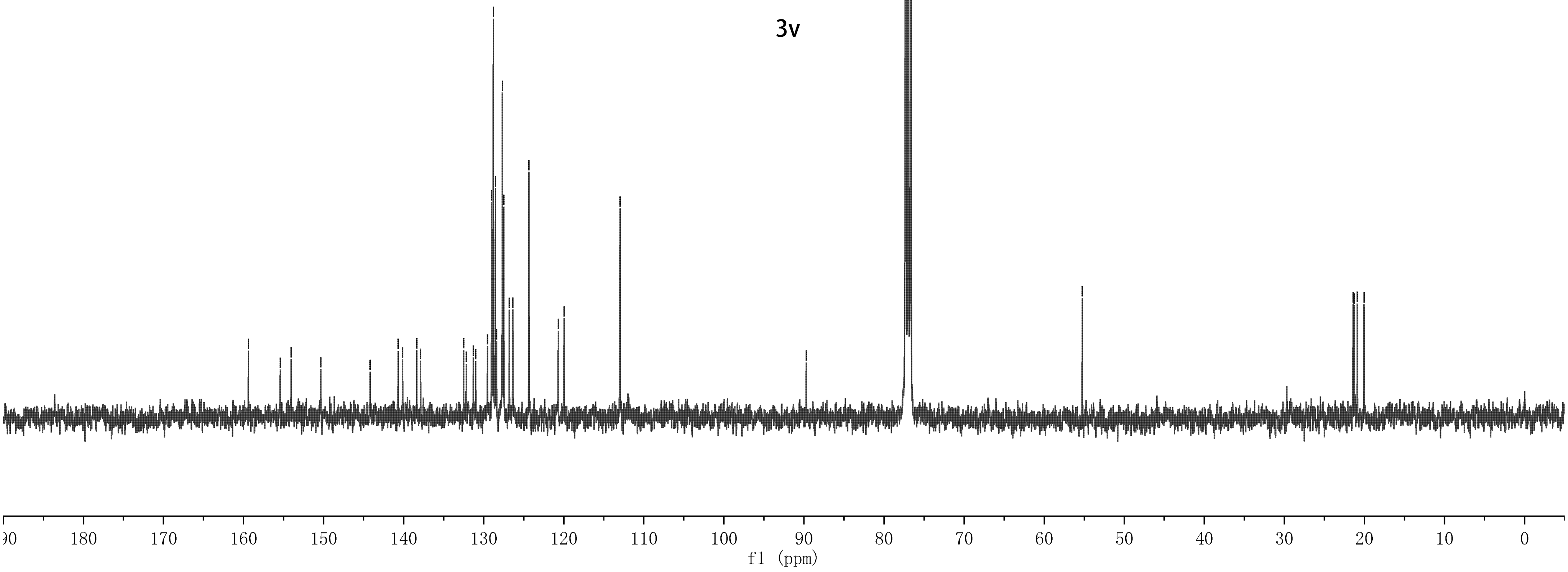

Parameter	Value
1 Title	CCM-2-65-H
2 Origin	
3 Solvent	CDCl3
4 Temperature	299.9
5 Number of Scans	16
6 Acquisition Time	4.0002
7 Acquisition Date	2022-09-03T16:30:39
8 Spectrometer Frequency	399.92
9 Spectral Width	8012.0

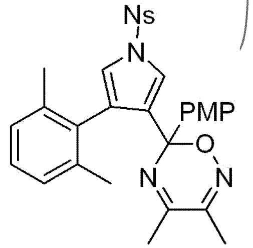

3w

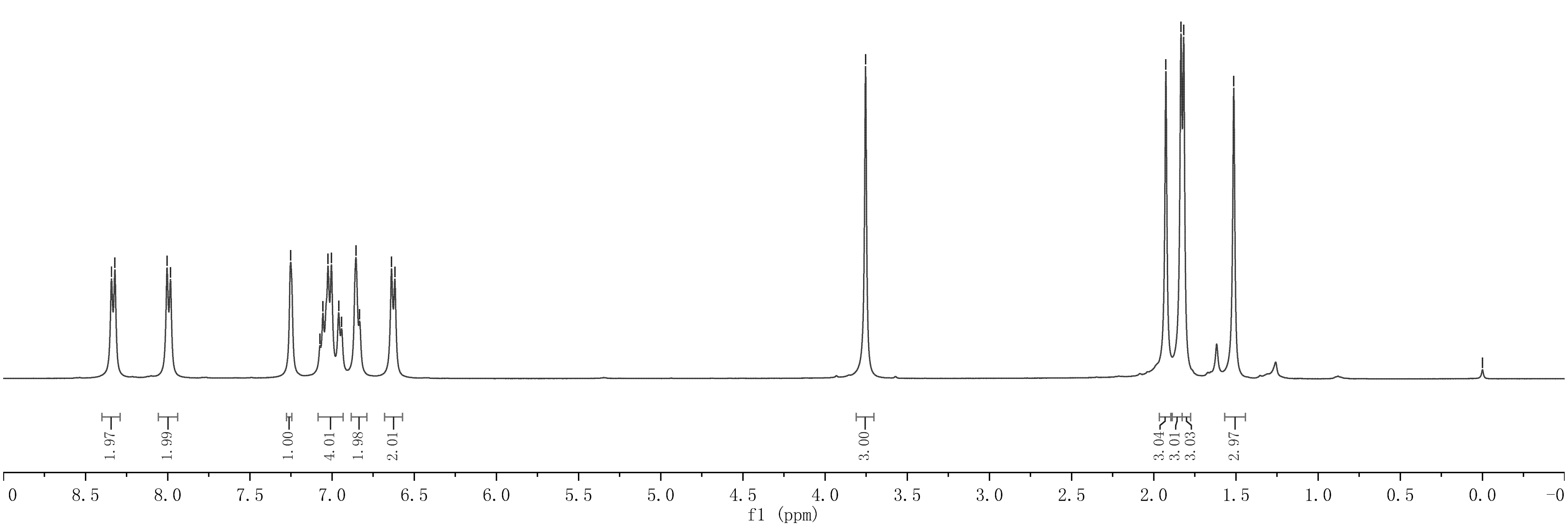

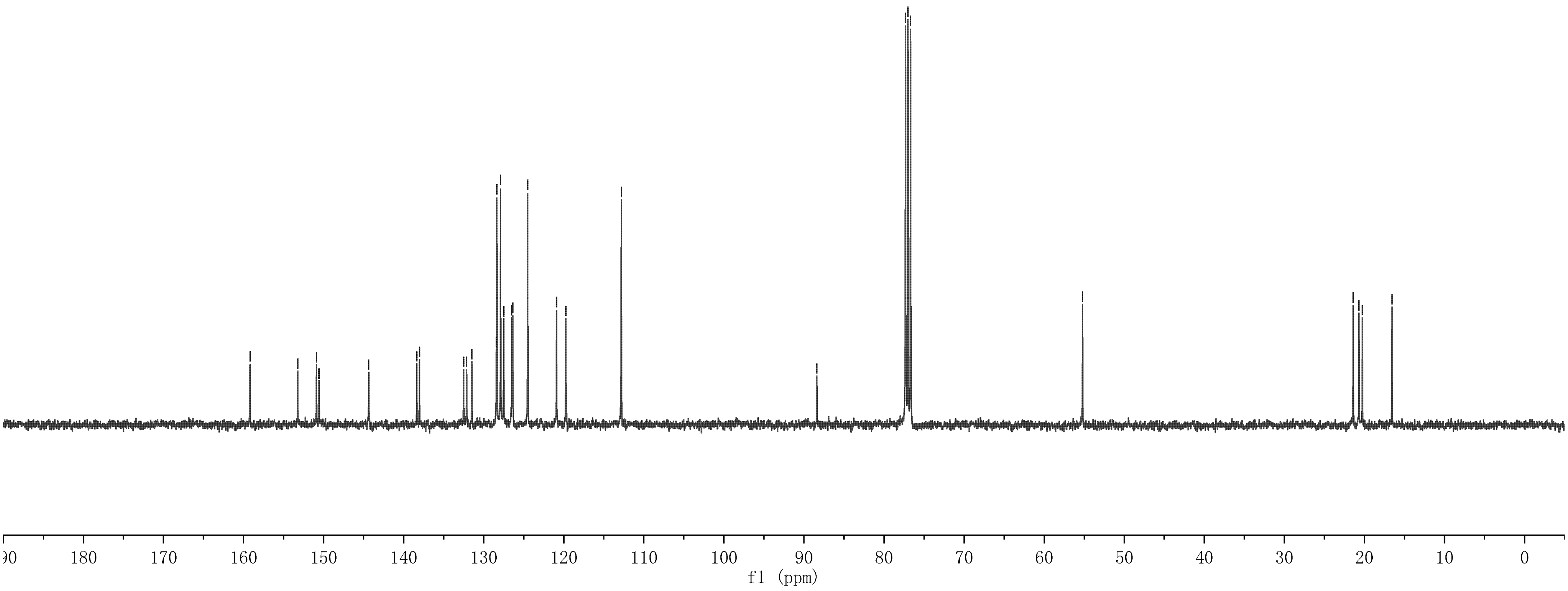

Parameter	Value
1 Title	CCM-2-65-C
2 Origin	
3 Solvent	CDCl3
4 Temperature	299.9
5 Number of Scans	400
6 Acquisition Time	1.0000
7 Acquisition Date	2022-09-03T16:46:25
8 Spectrometer Frequency	100.56
9 Spectral Width	26041.0

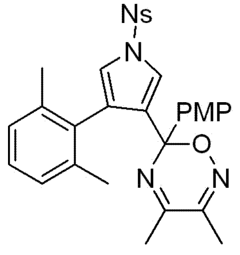

3w

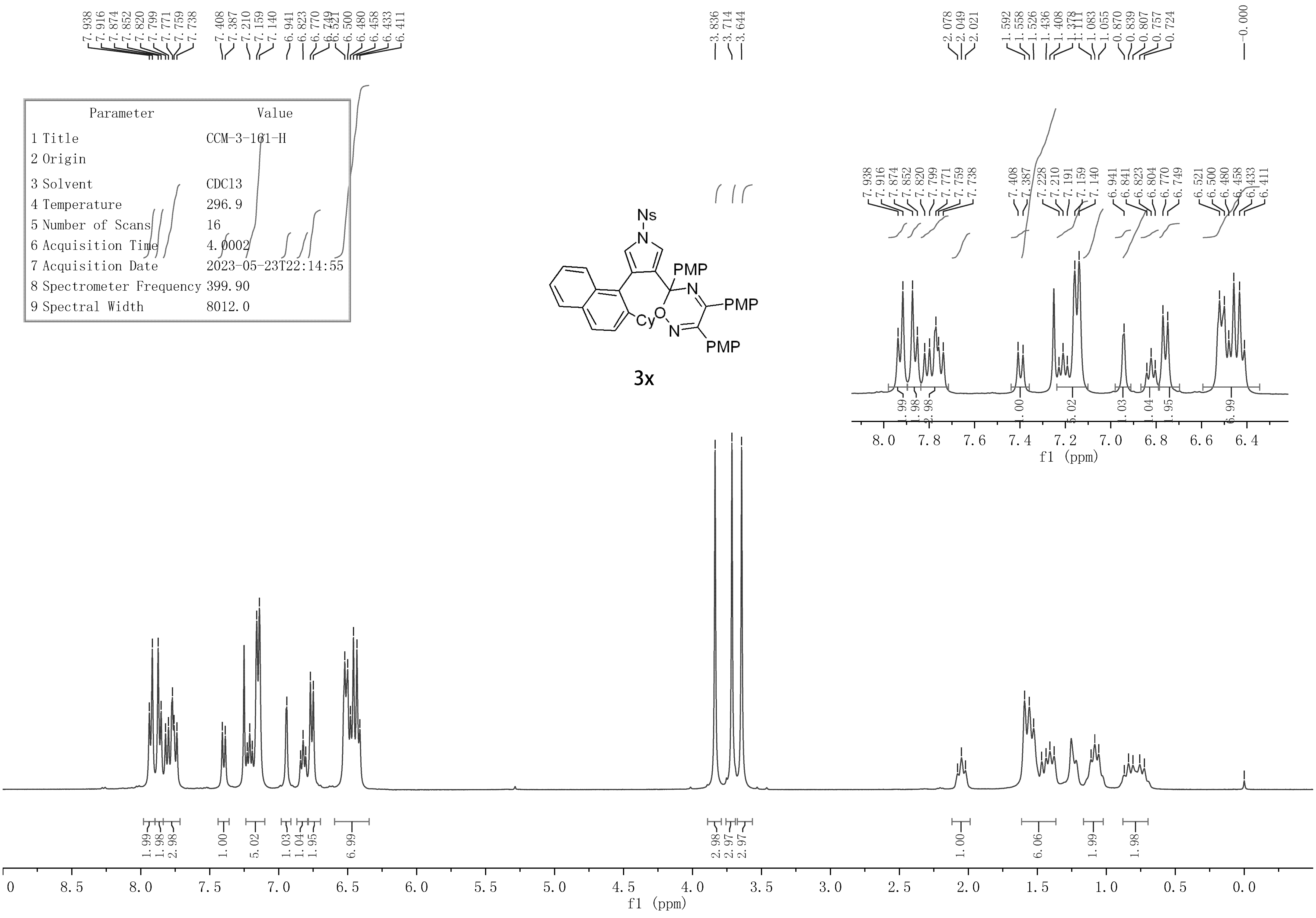

Parameter Value
1 Title CCM-3-161-H
2 Origin
3 Solvent CDCl3
4 Temperature 296.9
5 Number of Scans 16
6 Acquisition Time 4.0002
7 Acquisition Date 2023-05-23T22:14:55
8 Spectrometer Frequency 399.90
9 Spectral Width 8012.0
Ns
N
PMP
N
PMP
Cy
O
N
PMP
3x
f1 (ppm)

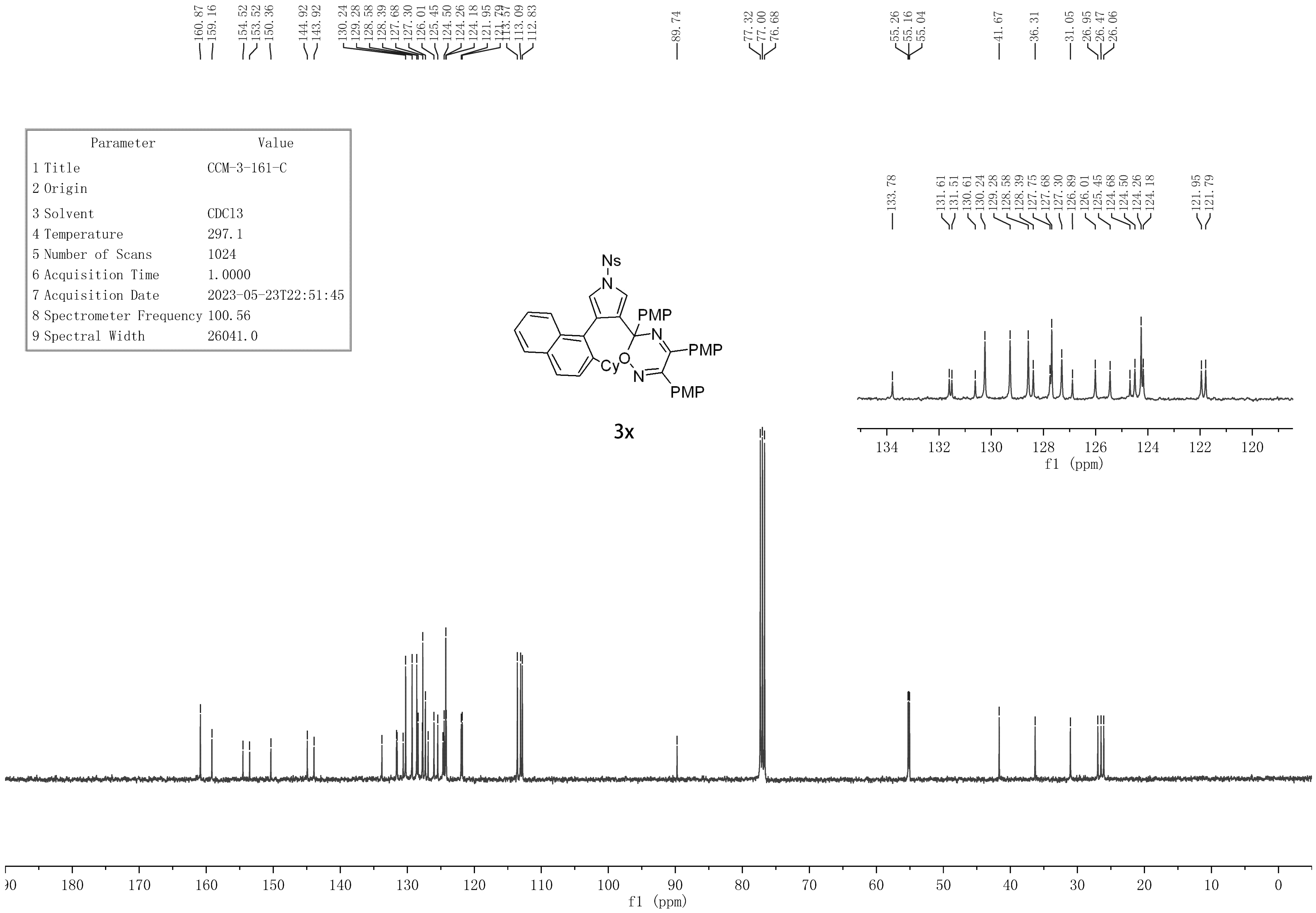

160.87
159.16
154.52
153.52
150.36
144.92
143.92
130.24
129.28
128.58
128.39
127.68
127.30
126.01
125.45
124.50
124.26
124.18
121.95
121.79
113.57
113.09
112.83
89.74
77.32
77.00
76.68
55.26
55.16
55.04
41.67
36.31
31.05
26.95
26.47
26.06
Parameter Value
1 Title CCM-3-161-C
2 Origin
3 Solvent CDCl3
4 Temperature 297.1
5 Number of Scans 1024
6 Acquisition Time 1.0000
7 Acquisition Date 2023-05-23T22:51:45
8 Spectrometer Frequency 100.56
9 Spectral Width 26041.0
Ns
N
PMP
N
PMP
Cy
O
N
PMP
3x
133.78
131.61
131.51
130.61
130.24
129.28
128.58
128.39
127.75
127.68
127.30
126.89
126.01
125.45
124.68
124.50
124.26
124.18
121.95
121.79
134 132 130 128 126 124 122 120
f1 (ppm)
180 170 160 150 140 130 120 110 100 90 80 70 60 50 40 30 20 10 0
f1 (ppm)

Parameter	Value
1 Title	CCM-3-15-H-
2 Origin	Bruker BioSpin GmbH
3 Solvent	CDCl3
4 Temperature	298.0
5 Number of Scans	5
6 Acquisition Time	4.0894
7 Acquisition Date	2023-02-13T15:34:49
8 Spectrometer Frequency	400.13
9 Spectral Width	8012.8

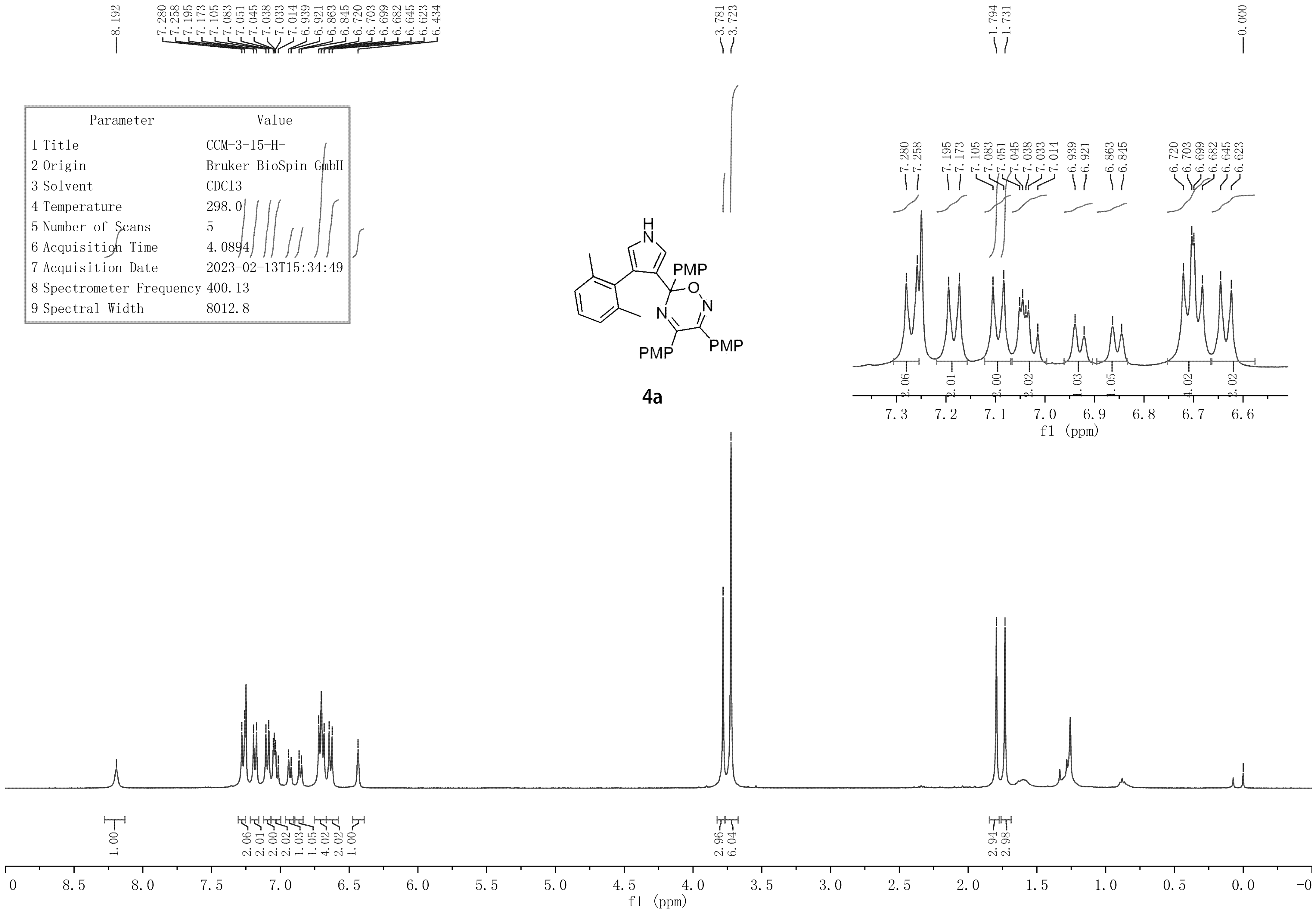

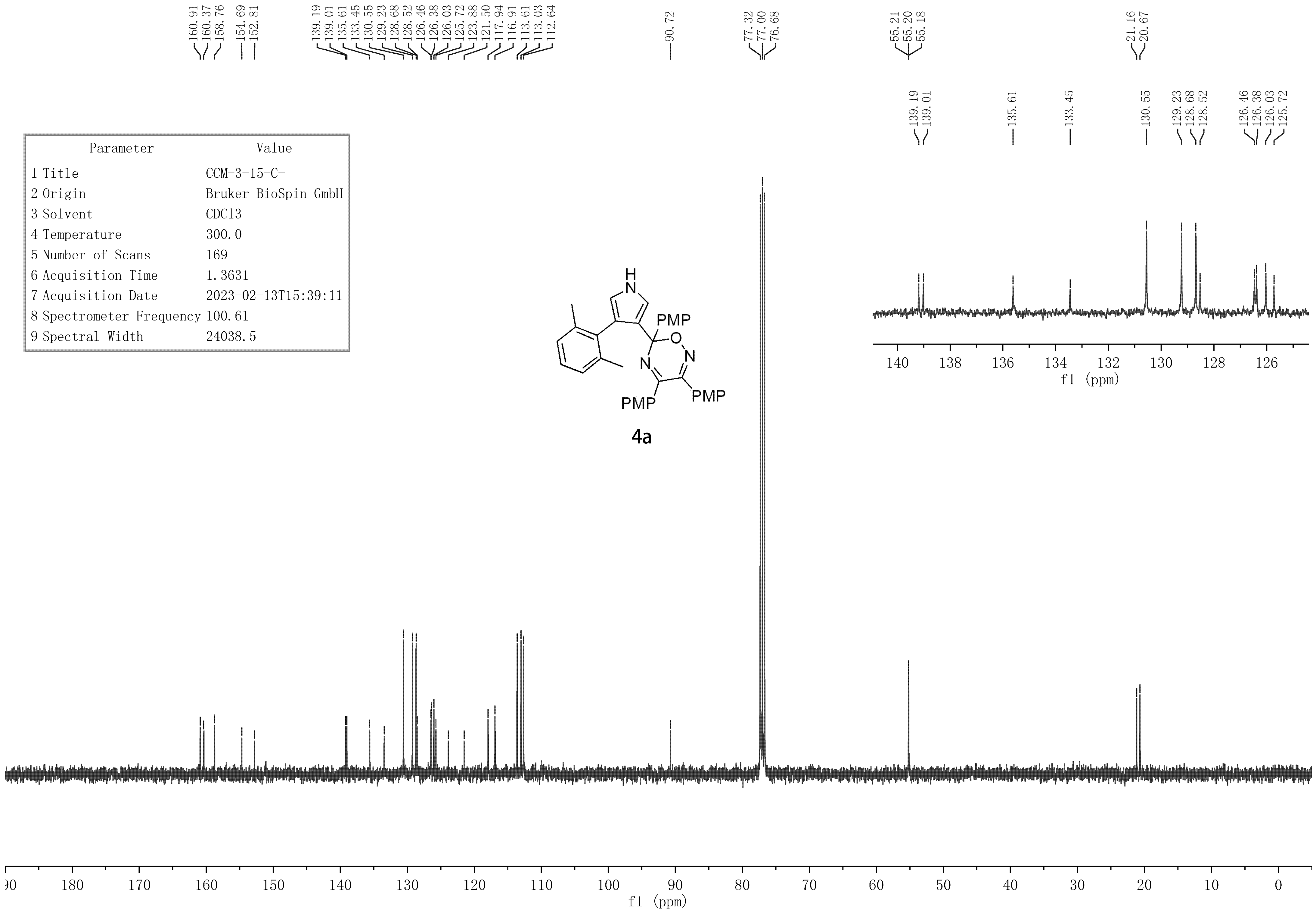

160.91
160.37
158.76
154.69
152.81
139.19
139.01
135.61
133.45
130.55
129.23
128.68
128.52
126.46
126.38
126.03
125.72
123.88
121.50
117.94
116.91
113.61
113.03
112.64
90.72
77.32
77.00
76.68
55.21
55.20
55.18
21.16
20.67
Parameter Value
1 Title CCM-3-15-C-
2 Origin Bruker BioSpin GmbH
3 Solvent CDCl3
4 Temperature 300.0
5 Number of Scans 169
6 Acquisition Time 1.3631
7 Acquisition Date 2023-02-13T15:39:11
8 Spectrometer Frequency 100.61
9 Spectral Width 24038.5
H
N
PMP
O
N
N
PMP
PMP
4a
f1 (ppm)
f1 (ppm)

Parameter	Value
1 Title	CCM-3-15-C135
2 Origin	Bruker BioSpin GmbH
3 Solvent	CDCl3
4 Temperature	300.0
5 Number of Scans	53
6 Acquisition Time	1.3631
7 Acquisition Date	2023-02-13T15:48:17
8 Spectrometer Frequency	100.61
9 Spectral Width	24038.5

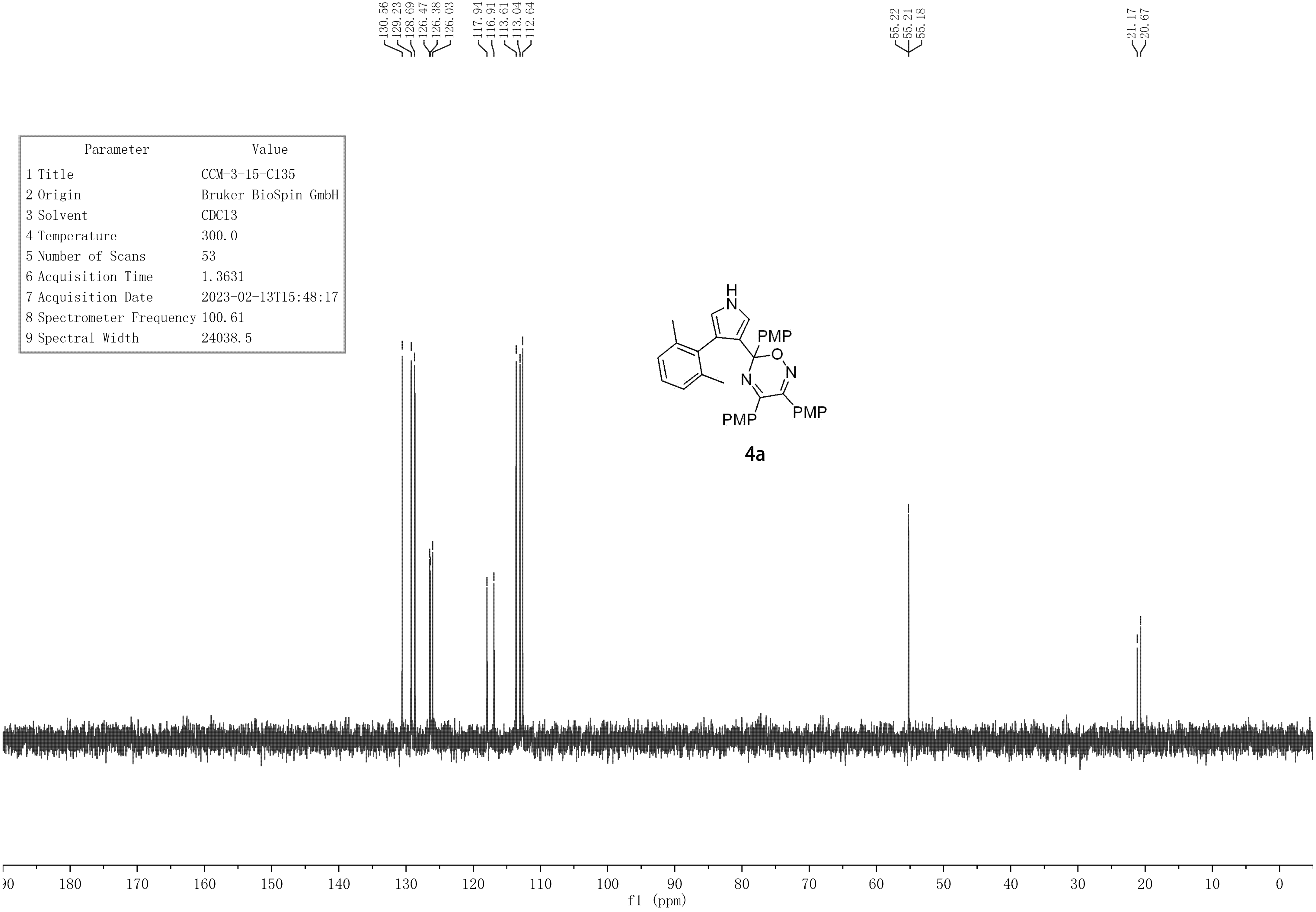

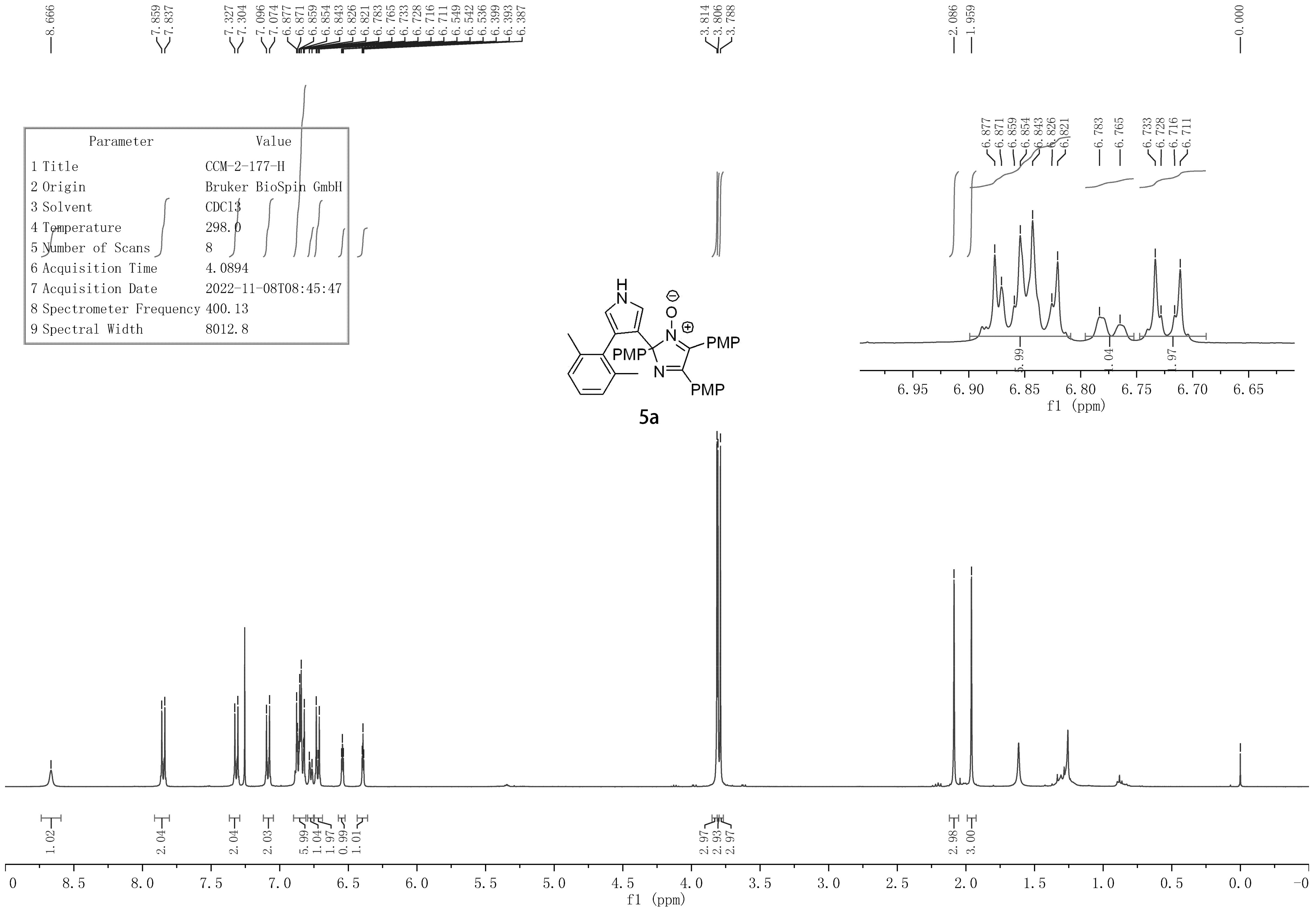
8.666
7.859
7.837
7.327
7.304
7.096
7.074
6.877
6.871
6.859
6.854
6.843
6.826
6.821
6.783
6.765
6.733
6.728
6.716
6.711
6.549
6.542
6.536
6.399
6.393
6.387
3.814
3.806
3.788
2.086
1.959
0.000
Parameter Value
1 Title CCM-2-177-H
2 Origin Bruker BioSpin GmbH
3 Solvent CDCl3
4 Temperature 298.0
5 Number of Scans 8
6 Acquisition Time 4.0894
7 Acquisition Date 2022-11-08T08:45:47
8 Spectrometer Frequency 400.13
9 Spectral Width 8012.8
H
N
O
N
PMP
PMP
N
PMP
5a
5.99
1.04
1.97
6.95 6.90 6.85 6.80 6.75 6.70 6.65
f1 (ppm)
1.02
2.04
2.04
2.03
5.99
1.04
1.97
0.99
1.01
2.97
2.93
2.97
2.98
3.00
8.5 8.0 7.5 7.0 6.5 6.0 5.5 5.0 4.5 4.0 3.5 3.0 2.5 2.0 1.5 1.0 0.5 0.0
f1 (ppm)

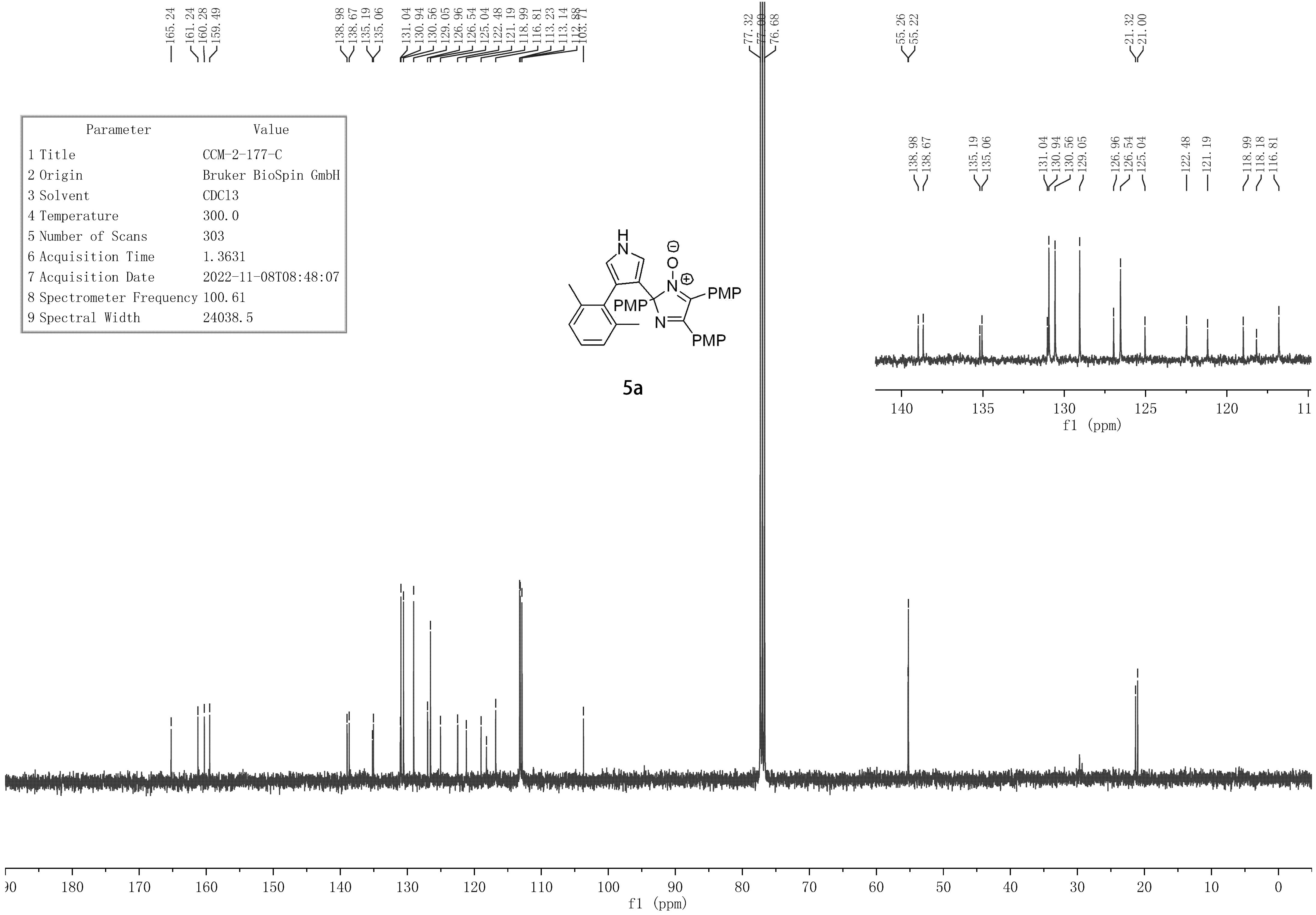

165.24
161.24
160.28
159.49
138.98
138.67
135.19
135.06
131.04
130.94
130.56
129.05
126.96
126.54
125.04
122.48
121.19
118.99
116.81
113.23
113.14
112.88
103.71
77.32
77.00
76.68
55.26
55.22
21.32
21.00
Parameter Value
1 Title CCM-2-177-C
2 Origin Bruker BioSpin GmbH
3 Solvent CDCl3
4 Temperature 300.0
5 Number of Scans 303
6 Acquisition Time 1.3631
7 Acquisition Date 2022-11-08T08:48:07
8 Spectrometer Frequency 100.61
9 Spectral Width 24038.5
H
N
O
N
PMP
PMP
N
PMP
5a
138.98
138.67
135.19
135.06
131.04
130.94
130.56
129.05
126.96
126.54
125.04
122.48
121.19
118.99
118.18
116.81
f1 (ppm)
f1 (ppm)

Parameter	Value
1 Title	CCM-2-177-C135
2 Origin	Bruker BioSpin GmbH
3 Solvent	CDCl3
4 Temperature	300.0
5 Number of Scans	67
6 Acquisition Time	1.3631
7 Acquisition Date	2022-11-08T09:05:48
8 Spectrometer Frequency	100.61
9 Spectral Width	24038.5

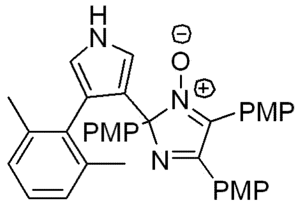

5a

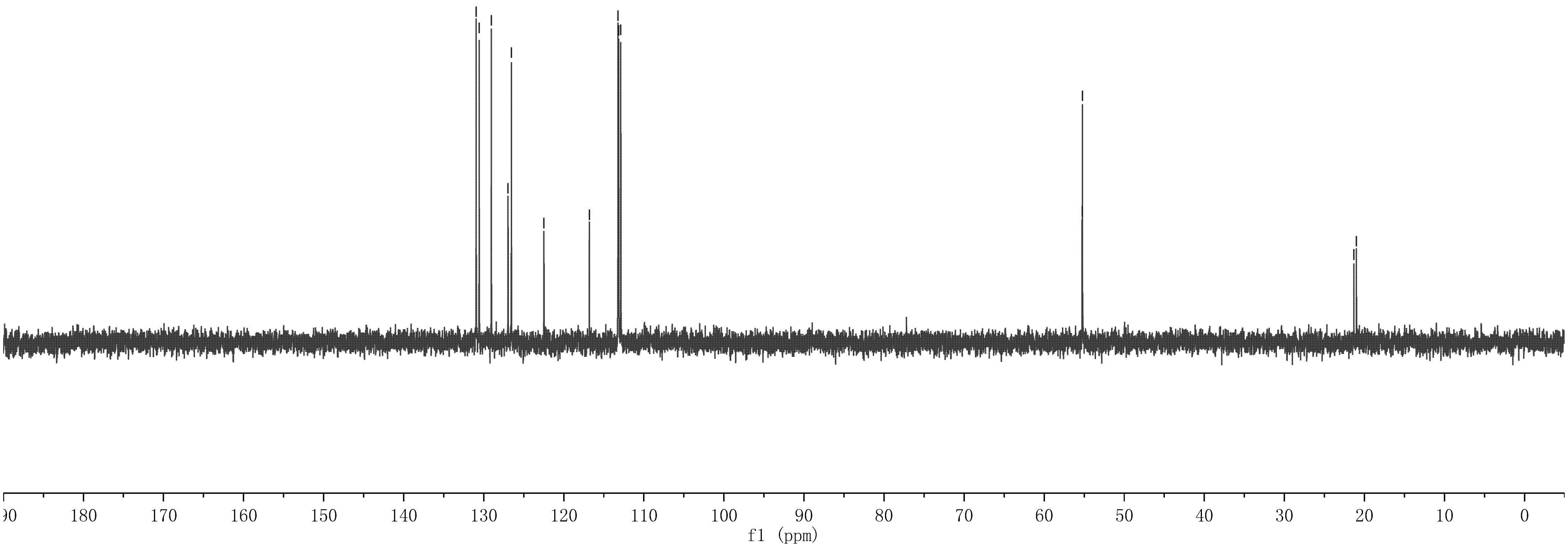

Supplement: Supplementary file 3 — Supplementary Data 1 [file 42004_2023_999_MOESM3_ESM.pdf]
